# Dysregulation of club cell biology in idiopathic pulmonary fibrosis

**DOI:** 10.1371/journal.pone.0237529

**Published:** 2020-09-17

**Authors:** Wu-Lin Zuo, Mahboubeh R. Rostami, Michelle LeBlanc, Robert J. Kaner, Sarah L. O’Beirne, Jason G. Mezey, Philip L. Leopold, Karsten Quast, Sudha Visvanathan, Jay S. Fine, Matthew J. Thomas, Ronald G. Crystal

**Affiliations:** 1 Department of Genetic Medicine, Weill Cornell Medical College, New York, New York, United States of America; 2 Department of Medicine, Weill Cornell Medical College, New York, New York, United States of America; 3 Department of Biological Statistics and Computational Biology, Cornell University, Ithaca, New York, United States of America; 4 Boehringer Ingelheim Pharma GmbH & Co. KG, Biberach an der Riss, Germany; 5 Boehringer Ingelheim Pharmaceuticals, Ridgefield, Connecticut, United States of America; University of Pittsburgh, UNITED STATES

## Abstract

Idiopathic pulmonary fibrosis (IPF) is a progressive, chronic fibrotic lung disease with an irreversible decline of lung function. “Bronchiolization”, characterized by ectopic appearance of airway epithelial cells in the alveolar regions, is one of the characteristic features in the IPF lung. Based on the knowledge that club cells are the major epithelial secretory cells in human small airways, and their major secretory product uteroglobin (SCGB1A1) is significantly increased in both serum and epithelial lining fluid of IPF lung, we hypothesize that human airway club cells contribute to the pathogenesis of IPF. By assessing the transcriptomes of the single cells from human lung of control donors and IPF patients, we identified two SCGB1A1^+^ club cell subpopulations, highly expressing MUC5B, a significant genetic risk factor strongly associated with IPF, and SCGB3A2, a marker heterogeneously expressed in the club cells, respectively. Interestingly, the cellular proportion of SCGB1A1^+^MUC5B^+^ club cells was significantly increased in IPF patients, and this club cell subpopulation highly expressed genes related to mucous production and immune cell chemotaxis. In contrast, though the cellular proportion did not change, the molecular phenotype of the SCGB1A1^+^SCGB3A2^high^ club cell subpopulation was significantly altered in IPF lung, with increased expression of mucins, cytokine and extracellular matrix genes. The single cell transcriptomic analysis reveals the cellular and molecular heterogeneity of club cells, and provide novel insights into the biological functions of club cells in the pathogenesis of IPF.

## Introduction

Club cells, characterized by the apical dome shaped morphology with dense cytoplasmic granules and short microvilli, represent approximate 20% of the epithelial cells and are the major secretory cells in human small airway epithelium (SAE) [1–3]. Secretoglobin family 1A member 1 (SCGB1A1), a secreted protein with anti-inflammatory properties, is the cell-specific marker and major secretory product for human SAE club cells [4–6]. Single cell RNA-sequencing of human SAE has identified the biological functions of club cells, including host defense, physical barriers and their potential roles in the pathogenesis of monogenetic and infectious lung disorders [3].

In asymptomatic smokers and chronic obstructive pulmonary disease (COPD), the number of club cells and the expression of club cell marker SCGB1A1 in the human SAE are decreased [1, 7], and the SCGB1A1 levels in lung epithelial lining fluid is decreased in asthma [8, 9], together suggesting that COPD and asthma are the club cell deficiency disorders. In contrast, SCGB1A1 expression is increased in both serum and epithelial lining fluid of idiopathic pulmonary fibrosis (IPF) [10]. Murine studies of club cells in the pulmonary fibrosis are mixed. Depletion of club cells by naphthalene suppress bleomycin-induced lung injury and fibrosis, while over-expression of another club cell marker SCGB3A2 in mouse lung exhibits an anti-fibrotic activity [11, 12]. Based on these observations, it is likely that the club cells play a unique role in the pathogenesis of IPF.

To assess this concept, we evaluated the club cell populations in the single-cell RNA-sequencing data from controls *vs* IPF described by Reyfman et al [13]. Analysis identified two unique club cell sub-populations, a SCGB1A1^+^ club cell sub-population highly expressing SCGB3A2, another club cell marker [14, 15], and a second SCGB1A1^+^ sub-population expressing MUC5B, a known genetic risk gene for IPF [16]. The proportion of SCGB1A1^+^MUC5B^+^ club cells was increased in IPF, with high expression of genes-related to mucins and immune cell chemoattractants. In contrast, the proportion of SCGB1A1^+^SCGB3A2^high^ club cells was similar to the controls, but the transcriptome of the SCGB1A1^+^SCGB3A2^high^ club cells was significantly dysregulated in IPF, with increased gene expression related to extracellular matrix formation, mucins and the growth factors relevant to pulmonary fibrosis. Together, these data provide novel insights into the molecular phenotypes and biological functions of club cells in the pathogenesis of IPF.

## Methods

### Source of single-cell RNA-sequencing data

The single-cell RNA-sequencing data described by Reyfman et al [13] was downloaded from a publically available database (Gene Expression Omnibus, series GSE122960). The data in the database did not contain protected health information or patient identifiers. The original publication noted that all procedures used to obtain tissue were reviewed by Institutional Review Boards as well as the funding agency and that patients involved in the study had provided written informed consent [13]. A total of 18,887 single cells from 4 control donors and 13,256 single cells from 4 IPF patient were analyzed. Detailed information of the 4 controls and the 4 IPF patients characterized by Reyfman et al [13] are summarized in S1 Table in S1 File.

### Data analysis

Processing of the single-cell RNA-sequencing data was performed using Seurat package V2 and R 3.5 [17]. Initially, gene expression matrices from the 4 controls and 4 IPF patients were combined. Cells were filtered out that have unique gene counts over 10,000 or less than 200 and the percentage of mitochondrial genes for each cell were less than 0.25. Gene expression matrix of 32,143 cells for these eight samples and total number of 18,732 genes was normalized by the total number of unique molecular identifiers (UMIs) per cell, multiplying by a scale factor (10,000) and then log transformed. The resulting 1,069 variably expressed genes were summarized by principal component analysis (PCA) and the 6 principle components further summarized using t-Distributed Stochastic Neighbor (t-SNE) dimensionally reduction method using RunTSNE function in Seurat package with perplexity parameter set to 30. The clustering algorithm, K-nearest neighbor graph based on the euclidean distance in PCA space was constructed by FindClusters function in Seurat to iteratively group cells together. Identification of clusters was guided by identifying marker genes by FindallMarkers function by Wilcox method in Seurat. Differential gene expression analysis was performed for each cluster between cells from controls *vs* IPF patients.

To evaluate the club cell populations, we re-analyzed cluster 1, which highly expressed club cell marker SCGB1A1. The most variable genes in cluster 1 cells were identified and applied dimensionally reduction PCA. The standard deviations of the PCs were plotted using a cutoff to identify which PCA is informative. The clustering algorithm, K-nearest neighbor graph based in PCA space approach implemented in FindClusters function was applied to find subclusters in cluster 1 cells. The resolution parameter was set as 0.5. This yielded 7 sub-clusters. The cell type of each sub-cluster of cluster 1 was identified by finding marker genes, and the sub-clusters visualized with t-SNE plots. Slingshot was applied for inferring cell lineage and pseudotimes from single cell RNA seq data [18]. The Slingshot algorithm uses prior knowledge for dimensional reduction and clustering and can capture multiple lineages in a dataset.

## Results

### Cellular and transcriptomic heterogeneity

**Fig 1:**
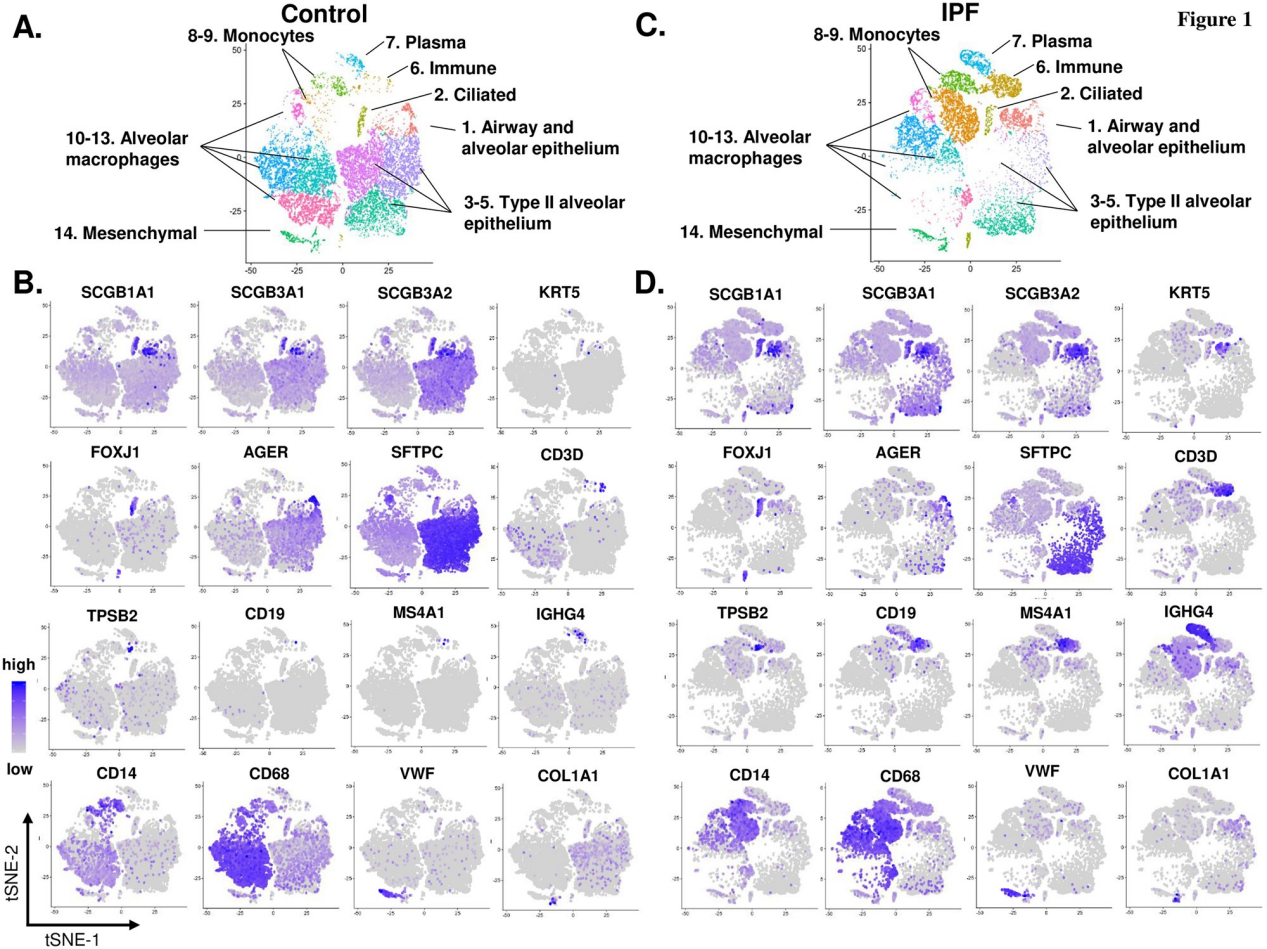
Identification of unique cell populations from the human lungs of controls *vs* IPF patients by single cell RNA-sequencing. Based on the data of Reyfman et al [13], 18,887 single cells from the lungs of 4 controls and 13,256 single cells from the lungs of 4 IPF patients (see S1 Table in S1 File for the detailed information of controls and IPF patients) were generated from the data in NCBI Gene Expression Omnibus (GEO accession number: GSE122960). **A.** Unsupervised t-distributed Stochastic Neighbor Embedding (t-SNE) clustering of the single cells from the lungs of 4 controls. A total of 14 unique cells populations were identified. **B.** Expression of the canonical markers of the different cell populations from control human lungs in the t-SNE plot shown in panel **A.** Club cell markers–SCGB1A1, SCGB3A1 and SCGB3A2; basal cell–KRT5; ciliated cell–FOXJ1; type I alveolar cells–AGER; type II alveolar cells–SFTPC; T cells–CD3D; mast cells–TPSB2; B cells–CD19 and MS4A1; plasma cells–IGHG; monocytes–CD14; alveolar macrophages–CD68; endothelial cells–VWF; and fibroblasts–COL1A1. **C.** Unsupervised t-SNE clustering of the single cells from the lung of 4 IPF patients. The same 14 cell populations were identified in IPF patients, as in the controls. **D.** Expression of the canonical markers of the different cell populations from in the IPF t-SNE plot shown in panel **C.** The markers are the same as in panel **B.** Based on signature genes (see S2 and S3 Tables in S1 File) and the expression of canonical markers of the different cell populations, the 14 cell populations identified in panels **A.** and **C.** were defined as: 1. airway (including basal and club cells) and alveolar (including type I and II alveolar cells) epithelial cells; 2. ciliated; 3–5. type II alveolar cells; 6. immune cells (including T cells, mast cells and B cells); 7. plasma cells; 8–9. monocytes; 10–13. alveolar macrophages; and 14. mesenchymal cells (including endothelial cells and fibroblasts).

To assess the cellular and transcriptomic heterogeneities of cell populations in the control donors and IPF patients, 18,887 single cells from 4 control donors and 13,256 single cells from 4 IPF patients were analyzed. The initial unsupervised t-SNE clustering of these single cells from human lung identified a total of 14 unique cell populations in both control donors (Fig 1A) and IPF patients (Fig 1C). Based on signature genes for each cell population and the expression of the canonical markers for the cell populations, the 14 cell populations were assigned as: 1 –airway and alveolar epithelial cells, highly expressing club cell markers SCGB1A1 and SCGB3A2, basal cell (BC) marker KRT5 and KRT15, and type I alveolar cell marker AGER and HOPX; 2 –ciliated cells, highly expressing FOXJ1 and DNAI1; 3~5 –type II alveolar cells, highly expressing SFTPC and ABCA3; 6 –immune cells, highly expressing T cell marker CD3D and CD8A, mast cell marker TPSB2 and KIT, and B cell marker CD19 and MS4A1; 7 –plasma cells, highly expressing IGHG4 and CD27; 8~9 –monocytes, highly expressing CD14 and ITGAM; 10~13 –alveolar macrophages, highly expressing CD68 and PPARG; 14 –mesenchymal cells, highly expressing endothelial cell marker VWF and PECAM1, and fibroblast marker COL1A1 and COL14A1 (Fig 1B and 1D; S1 Fig and S2 Table in S1 File).

### Club cell sub-populations in human lung of controls *vs* IPF

**Fig 2:**
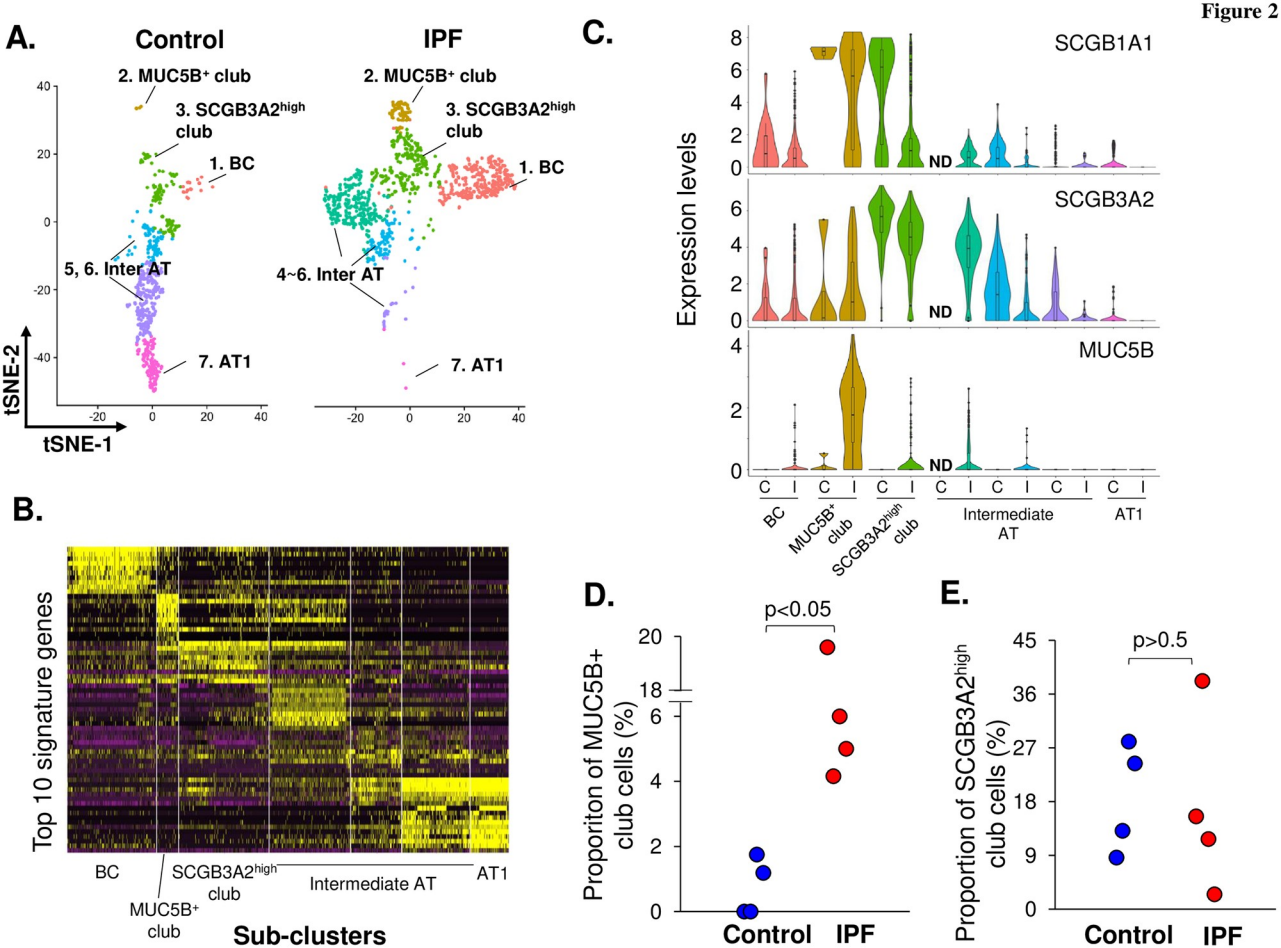
Identification of club cell sub-populations from the human lung of controls *vs* IPF patients by single cell RNA-sequencing. **A.** Unsupervised t-SNE clustering of the single cells from airway epithelial and alveolar cell cluster (cluster 1) in Fig 1A and 1C. A total of 6 unique cell populations were identified in controls (*left*) and 7 unique cell populations in IPF patients (*right*). Based on the signature genes for each cell population (see S3 Table and S3A Fig in S1 File), these cell populations were defined as: (1) basal cells–highly expressing KRT5; (2 and 3) club cells–highly expressing SCGB1A1; (4–6) intermediate alveolar cells–expressing type I alveolar cell markers AGER and HOPX, and type II alveolar cell marker SFTPC and ABCA3; (7) type I alveolar cells–highly expressing both AGER and HOPX. **B.** Heatmap plot in the expression of top 10 signature genes from the 7 unique cell populations identified in panel **A. C.** Violin plots showing the expression of club cell genes SCGB1A1 *(top)*, SCGB3A2 *(middle)*, and IPF genetic risk factor gene MUC5B *(bottom)* in the cell populations from control (C) and IPF (I) donors identified in panel **A**. Based on the violin plots, the sub-cluster 2 and 3 were defined as SCGB1A1^+^MUC5B^+^ and SCGB1A1^+^SCGB3A2^high^ club cells, respectively. Note that sub-cluster 4 was not detected (ND) in control cell populations. **D.** Fraction of the SCGB1A1^+^MUC5B^+^ club cell sub-population in controls *vs* IPF patients. **E.** Fraction of the SCGB1A1^+^SCGB3A2^high^ club cell sub-population in controls *vs* IPF. In panels **D**-**E.**, each dot represents one individual. *Blue dots*–controls, *red dots*–IPF patients. p values are shown.

The airway epithelium are associated with the pathogenesis of IPF [19–24]: (1) there is small airway pathology and dysfunction in IPF [19, 20] (2) the numbers of epithelial BC are increased in IPF [20, 22], and (3) BC genes are associated with the mortality in IPF [22]. To further study the role of airway epithelial cells in the IPF pathogenesis, the 569 single cells from control donors and 1,013 single cell from IPF patients in the airway and alveolar epithelial cell cluster (cluster 1 in Fig 1A and 1C) were re-analyzed. The unsupervised t-SNE clustering identified 6 and 7 cell populations in the control donors and IPF patients, respectively (Fig 2A, S2 Fig in S1 File). Using the signature genes for each cluster and the known markers for the epithelial cell populations, we assigned these cell populations as: 1 –BC, highly expressing KRT5; 2~3 –club cells, highly expressing SCGB1A1; 4~6 –intermediate alveolar cells, expressing both type II (SFTPC, ABCA3) and I (AGER, HOPX) alveolar cell markers; 7 –type I alveolar cells, highly expressing AGER and HOPX (Fig 2B and 2C, S3A Fig and S3 Table in S1 File).

Consistent with the literature that the airway and alveolar epithelial cells are reorganized in the IPF lung [19–26], the proportions of airway BC and alveolar cells were increased and decreased, respectively (S3B Fig in S1 File). Two club cell subpopulations, both highly expressing the club cell marker SCGB1A1, were identified in the single-cell data. One club cell subpopulation highly expressed MUC5B (MUC5B^+^ club cells), a known genetic risk gene for IPF [16], while the other club cell subpopulation highly expressed SCGB3A2 (SCGB3A2^high^ club cells), a marker heterogeneously expressed in the club cells [14, 15] (Fig 2C). Both club cell populations were present in both control and IPF datasets (Fig 2C). While there were only a few MUC5B^+^ club cells in the control donors, the fraction of MUC5B^+^ club cells was significantly increased in the IPF patients (Fig 2D). In contrast, the proportion of SCGB3A2^high^ club cells did not change in control donors *vs* IPF patients (Fig 2E).

### Transcriptomic differences between MUC5B^+^ and SCGB3A2^high^ club cells

**Fig 3:**
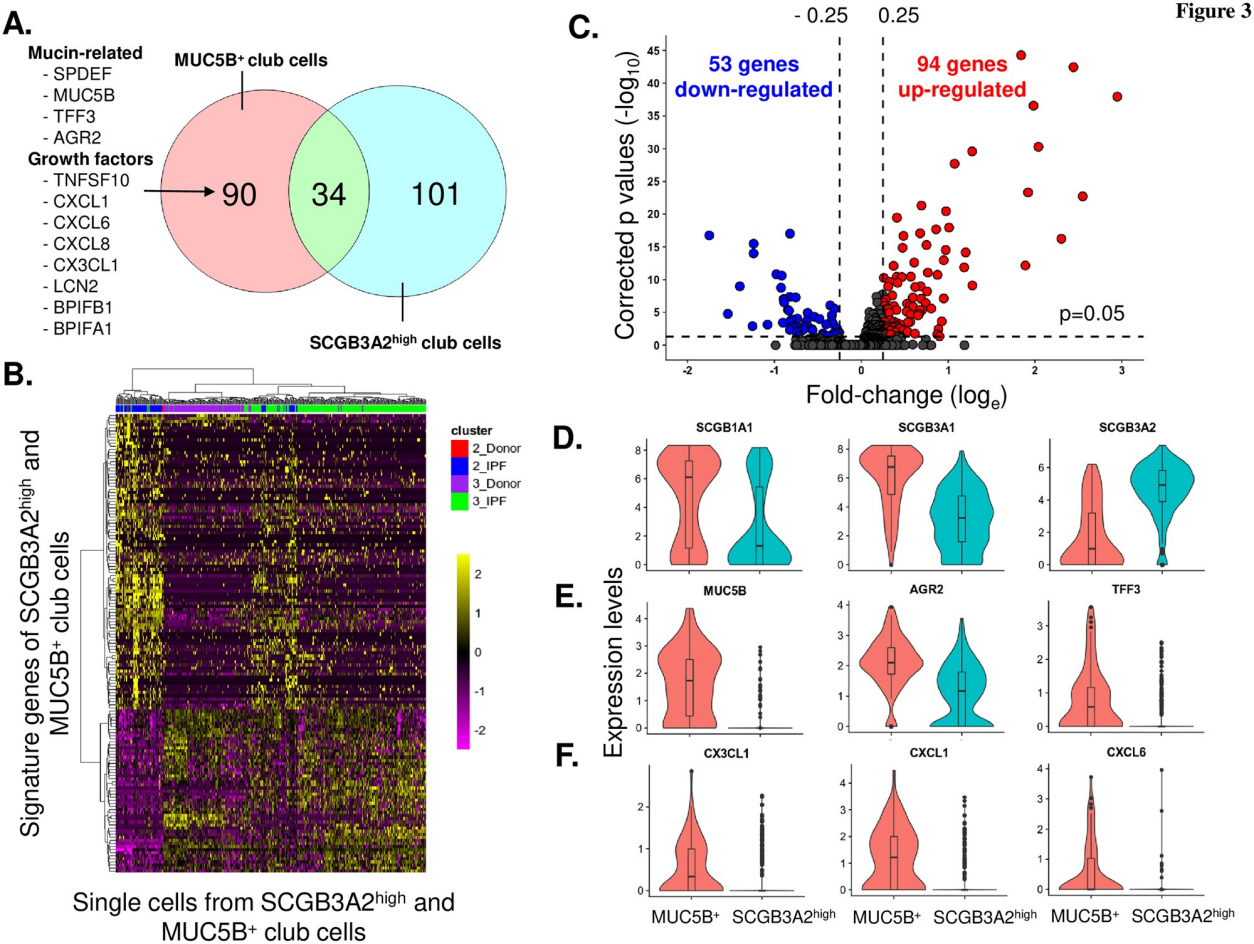
Transcriptomic differences between the club cell sub-populations. **A.** Venn diagram of the signature genes from club cell sub-populations. *Left*–signature genes of SCGB1A1^+^MUC5B^+^ club cells (n = 124), *right*–signature genes of SCGB1A1^+^SCGB3A2^high^ club cells (n = 135), and overlap signature genes (n = 34). The SCGB1A1^+^MUC5B^+^ club cell unique signature genes related to mucin and growth factors are highlighted on the left of the Venn diagram. **B.** Heat map plot showing the expression of the signature genes in SCGB1A1^+^SCGB3A2^high^ and SCGB1A1^+^MUC5B^+^ club cells. *X-axis*–signature genes, *y-axis*–single cells. *Y-axis*, *red*–control of SCGB1A1^+^MUC5B^+^ club cells; *blue—*IPF SCGB1A1^+^MUC5B^+^ club cells; *purple*–control SCGB1A1^+^SCGB3A2^high^ club cells; and *green—*IPF SCGB1A1^+^SCGB3A2^high^ club cells. **C.** Volcano plots of significantly down-regulated (53 genes, *blue*) and up-regulated (94 genes, *red*) genes in SCGB1A1^+^MUC5B^+^ club cells *vs* SCGB1A1^+^SCGB3A2^high^ club cells. *Y-axis*–p values (-log_10_), *x-axis*–the fold-change (log_e_). Genes selected for analysis differed between populations by a fold-change (log_e_) >0.25 as the up-regulated genes or <-0.25 as the down-regulated genes (vertical dotted lines), and p<0.05 with Bonferroni correction (horizontal line at y = 0.5). **D.** Violin plots of the expression of club cell genes (SCGB1A1, SCGB3A1 and SCGB3A2) in SCGB1A1^+^MUC5B^+^ and SCGB1A1^+^SCGB3A2^high^ club cells. **E.** Violin plots in the expression of mucin-related genes (MUC5B, AGR2 and TFF3) in SCGB1A1^+^MUC5B^+^ and SCGB1A1^+^SCGB3A2^high^ club cells. **F.** Violin plots in the expression of growth factor-related genes (CX3CL1, CXCL1, CXCL6 and CXCL8) in SCGB1A1^+^MUC5B^+^ and SCGB1A1^+^SCGB3A2^high^ club cells. In **D.**-**F.**, *left–*SCGB1A1^+^MUC5B^+^, *right*–SCGB1A1^+^SCGB3A2^high^ club cells.

To assess the differences between MUC5B^+^ and SCGB3A2^high^ club cells, we first compared the signature genes of these 2 club cell subpopulations. There were total 124 and 135 signature genes for the MUC5B^+^ and SCGB3A2^high^ club cells, respectively. Only 34 of these signature genes, including the club cell genes SCGB1A1, SLPI and WFDC2, were overlapping (Fig 3A), suggesting differing roles for these club cell populations in the pathogenesis of IPF. Compared to the SCGB3A2^high^ club cells, the IPF-induced MUC5B^+^ club cells had a unique signature gene set, which included genes related to mucous cell differentiation (SPDEF, MUC5B, TFF3 and AGR2), growth factors related to host defense (LCN2, BPIFB1 and BPIFA1) and immune cell attractants (CXCL1, CXCL6, CXCL8 and CX3CL1) (Fig 3A). The heatmap plot of the signature genes also showed that MUC5B^+^ and SCGB3A2^high^ club cells were clearly different, while a subset of the MUC5B^+^ club cells in IPF patients co-expressed the SCGB3A2^high^ club cell signature genes (Fig 3B).

Comparison of the transcriptomes of the MUC5B^+^ and SCGB3A2^high^ club cell subpopulations identified 53 genes significantly down-regulated and 94 genes significantly up-regulated in the MUC5B^+^ club cells. In this MUC5B^+^ subpopulation, the club cell markers SCGB1A1 and SCGB3A1 were up-regulated, while SCGB3A2 was down-regulated (Fig 3D). Consistent with the signature gene analysis, expression of the mucin-related genes (AGR2 and TFF3), and the immune cell chemoattractant-related genes were all significantly increased in the MUC5B^+^ club cells (Fig 3E and 3F).

### Transcriptomic dysregulation of the SCGB3A2^high^ club cells in IPF

**Fig 4:**
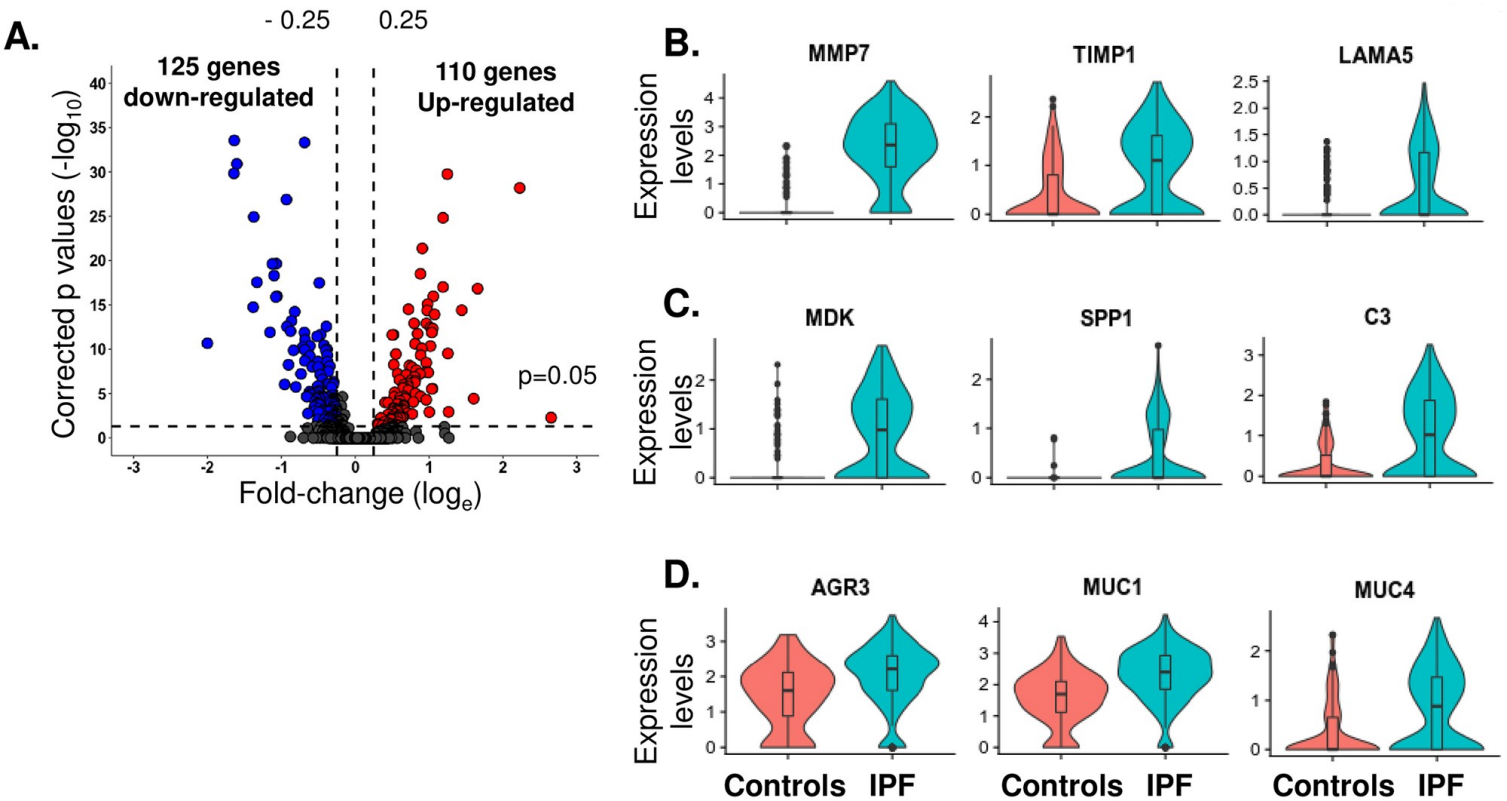
Transcriptomic dysregulation of SCGB3A2^high^ club cells in IPF. **A.** Volcano plots of significantly down-regulated (125 genes, *blue*) and up-regulated (110 genes, *red*) genes in SCGB1A1^+^SCGB3A2^high^ club cells from IPF. *Y-axis*–p values (-log_10_), *x-axis*–the fold-change (log_e_). Cutoff: shown as dotted lines, fold-change (log_e_) > 0.25 as the up-regulated genes or <-0.25 as the down-regulated genes, p value<0.05 with Bonferroni correction. **B.** Violin plots of the expression of extracellular matrix-related genes (MMP7, TIMP1 and LAMA5) in SCGB1A1^+^SCGB3A2^high^ club cells of controls *vs* IPF. **C.** Violin plots in the expression of growth factor-related genes (MDK, SPP1 and C3) in SCGB1A1^+^SCGB3A2^high^ club cells from controls *vs* IPF. **D.** Violin plots of the expression of mucin-related genes (AGR3, MUC1 and MUC4) in SCGB1A1^+^SCGB3A2^high^ club cells of controls *vs* IPF. In **B.**-**D.**, *left–*controls, *right*–IPF.

Though the proportion of the SCGB3A2^high^ fraction club cells did not change, the transcriptome of the SCGB3A2^high^ club cells was significantly altered in IPF. A total of 125 genes were significantly down-regulated, while 110 genes were up-regulated in the IPF SCGB3A2^high^ club cells (Fig 4A). Gene ontology analysis identified that the down-regulated genes in SCGB3A2^high^ club cells in IPF were mainly associated with translation, nuclear-transcribed mRNA catabolic process, viral transcription, response to hormone stimulus and cAMP (S4A Fig in S1 File). In contrast, the up-regulated genes in SCGB3A2^high^ club cells in IPF were related to immune response, antigen processing and presentation, interferon-gamma signaling pathway, cellular protein metabolic process, and T cell co-stimulation (S4B Fig in S1 File), suggesting that the SCGB3A2^high^ club cells of IPF patients were highly associated with immune cell maturation. In addition, expression of many genes related to the functions of extracellular matrix formation (MMP7, TIMP1 and LAMA5) and growth factors (MDK, SPP1 and C3) were significantly up-regulated in the SCGB3A2^high^ club cells in IPF (Fig 4B and 4C). The SCGB3A2^high^ club cells in IPF also showed a mucous cell-like phenotype, which had increased expression of AGR3, MUC1 and MUC4 (Fig 4D).

### SCGB3A2^+^ alveolar cells in IPF

**Fig 5:**
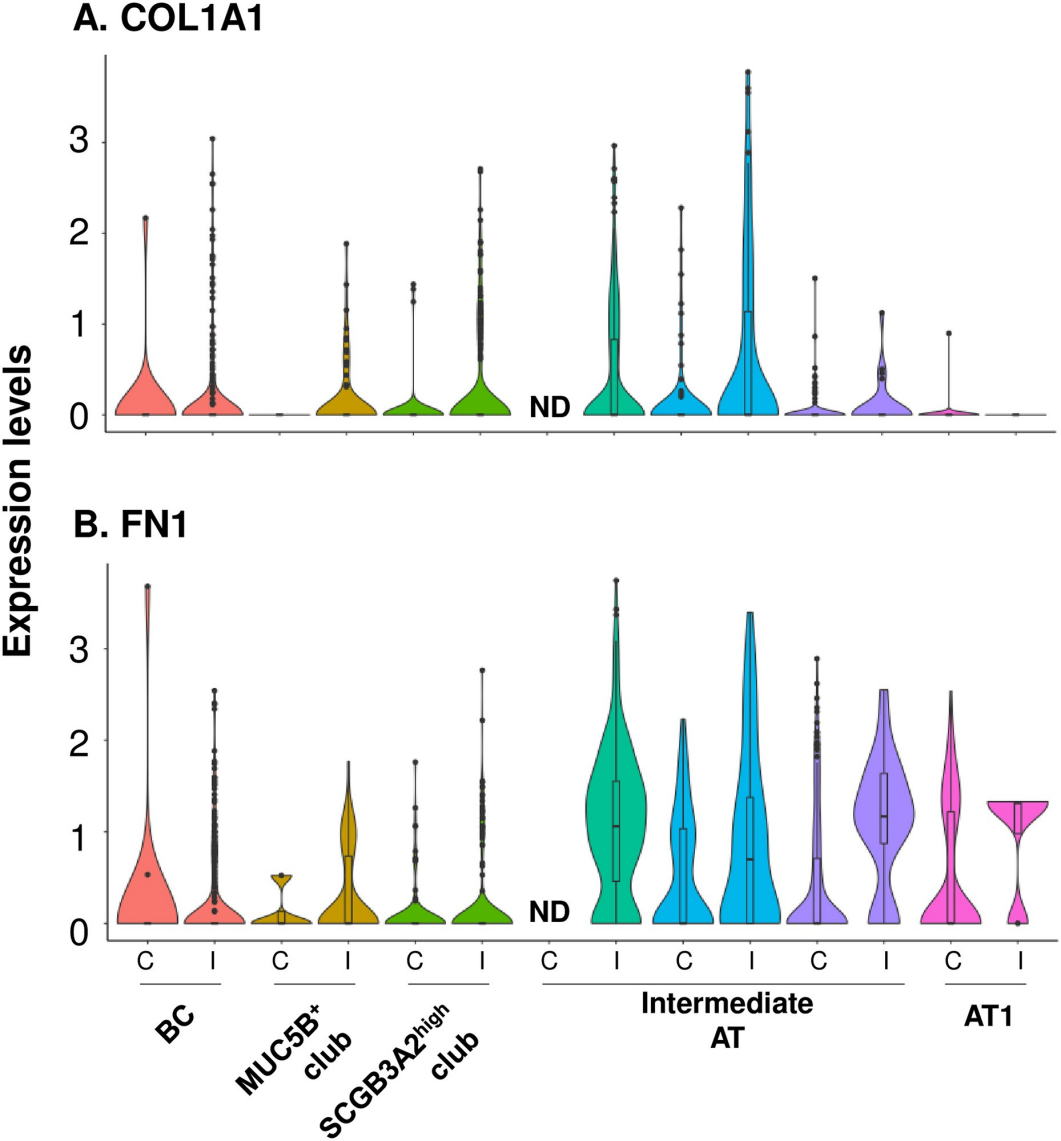
Expression of extracellular matrix genes in SCGB3A2^+^ alveolar cells in populations of lung epithelial cells. Violin plots showing the expression of extracellular matrix genes SCGB1A1 *(top)*, SCGB3A2 *(middle)*, and IPF genetic risk factor gene MUC5B *(bottom)* in the cell populations from control (C) and IPF (I) donors identified in Fig 2A. **A**. SCGB1A1. **B.** FN1. Note that sub-cluster 4 was not detected (ND) in control cell populations.

Lastly, we identified a subpopulation of alveolar cells mainly contributed by one IPF individual, only presented in IPF and not in the control donors (Fig 2A, S2 Fig in S1 File). This alveolar cell population expressed mild level of type I alveolar cell markers AGER and HOPX and high level of type II alveolar cell markers SFTPC and ABCA3 (S3A Fig in S1 File), and also had some characteristics of club cells, by highly expressing SCGB3A2 (Fig 2B and 2C). Interestingly, the SCGB3A2^+^ alveolar cells highly expressed collagen (COL1A1) and fibronectin (FN1) genes (Fig 5A and 5B), suggesting that, in addition to myofibroblasts, the SCGB3A2^+^ hyperplastic alveolar cells also contributed to the extracellular matrix deposition in IPF.

### Cell lineage of the club cells in IPF

To identify the ontology of the club cell populations in IPF patients, we applied a cell lineage pseudotime analysis on the IPF single cells in Fig 2. As BC are the stem/progenitor cells for human club cells [3], two different lineages were identified. A single lineage started from BC and gave rise to SCGB3A2^high^ club cells. At that point, the path bifurcated leading to either MUC5B+ club cells or SCGB3A2^+^ alveolar cells leading to intermediate alveolar cells and type I alveolar cells as the destination (S5 Fig in S1 File).

## Discussion

IPF is a progressive, chronic fibrotic lung disorder of unknown cause and a median survival of 2 to 4 years since diagnosis [27, 28]. To determine the roles of club cell populations, the major secretory cells in human small airways, in the pathogenesis of IPF, we assessed the single cell transcriptome data from human lung of control donors and IPF patients. Two unique club cell subpopulations were identified in the human lung, highly expressing SCGB1A1 and MUC5B, and SCGB1A1 and SCGB3A2, respectively. The SCGB1A1^+^ MUC5B^+^ club cell is an IPF-dependent cell population, highly expresses genes related to mucin and chemoattractant cytokines to immune cells. In contrast, the SCGB1A1^+^ SCGB3A2^high^ club cell subpopulation presents in equal proportion in IPF and controls. The transcriptome of this club cell subpopulation is significantly altered in IPF patients, with increased expression of mucins, cytokines and extracellular matrix genes.

The pathogenesis of IPF is associated with loss of alveolar architecture, airway epithelial remodeling, activation of fibroblasts, accumulation of extracellular matrix and chronic inflammation [19–28]. Originally observed 4 decades ago [19], there is increased evidence suggesting that “bronchiolization”, characterized by ectopic appearance of airway epithelial cells in the alveolar space, is a characteristic feature in the IPF lung, likely playing a crucial role in IPF progression [19–24]. Basal cells (BC), the stem/progenitor cells in the human airway epithelium, are normally absent in the normal human alveoli. In contrast, an increased number of BC are present in the remodeled alveolar epithelium of IPF patients [20, 22]. A group of BC expressed genes is associated with the clinical outcome and mortality of IPF patients, consistent with an important role of airway basal cells in IPF [22]. Metaplasia of goblet cells, a differentiated product of BC, also plays a role in “bronchiolization”. Immuno-stainings and RNA-sequencing have identified extensive goblet cell metaplasia in IPF, and many of the goblet cells co-express alveolar type I and II cell markers [21, 23, 24].

There are several lines of evidence that indicate a potential role of club cells in the pathogenesis of IPF. Club cells, another differentiation product of BC, are the major secretory and host defense cell population in human small airway epithelium. In contrast to the decrease of club cell numbers in COPD and asthma, SCGB1A1, the major secretory product of club cells, is increased in both serum and bronchoalveolar lavage (BAL) fluid of IPF patients [10]. In mouse, depletion of club cells by naphthalene attenuates bleomycin-induced lung injury and fibrosis [11, 12]. In contrast, over-expression of SCGB3A2, a club cell marker with heterogeneous expression [14, 15], in mouse lung accelerates resolution of bleomycin-induced pulmonary fibrosis [11, 12].

By assessing the transcriptomes of the single cells from human lung, we were able to evaluate the cellular and transcriptomic heterogeneities of club cells in IPF and controls from the study of Reyfman et al [13]. First, we identified a SCGB1A1^+^ club cell sub-population highly expressing MUC5B. This is of significant interest in the context that MUC5B promoter polymorphism is a significant genetic risk factor strongly associated with IPF [16]. Overexpression of MUC5B induces impaired mucociliary clearance and enhances lung fibrosis in mice [16]. The single cell data analysis showed that the proportions of SCGB1A1^+^ MUC5B^+^ club cells was significantly increased in the IPF lung, consistent with observation that the MUC5B expression is increased in IPF [29]. The single cell analysis permitted identification of the molecular phenotype and function of the SCGB1A1^+^ MUC5B^+^ club cells in IPF. We found that these MUC5B^+^ club cells expressed high levels of mucin-related genes such as SPDEF, TFF3 and AGR2, suggesting the MUC5B^+^ club cells are a goblet cell-like subpopulation [30, 31]. Moreover, the SCGB1A1^+^ MUC5B^+^ club cells expressed a number of chemoattractant cytokines, including the neutrophil chemoattractant CXCL1, 6 and 8 [32–34], and CX3CL1, a potent chemoattractant for T cells and monocytes [35], suggesting that this club cell subpopulation could interact with immune cells.

In regard to the ontology of the MUC5B^+^ club cells migrating to the alveoli in IPF, our previous study demonstrated that MUC5B is highly expressed in normal human small airway club cells [3], i.e., SCGB1A1^+^ MUC5B^+^ club cells in broncholarization of the IPF lung might be the result of migration of club cells from human small airway epithelium [20, 36]. Another possibility might be the small airway BC, since: (1) human small airway BC can differentiate into SCGB1A1^+^ MUC5B^+^ club cells [3]; and (2) there are increased numbers of BC in the lower respiratory tract of the IPF lung [20, 22]. The cell lineage analysis support this hypothesis that the BC generate the SCGB3A2^high^ club cells first, to further differentiated into MUC5B+ club cells.

SCGB3A2 is a another marker with heterogeneous expression in club cells [14, 15]. There is a SCGB3A2^high^ club cell subpopulation in the murine airways that highly express UPK3A, but reduced expression of SCGB1A1 [14, 15]. This cell population contributes to the generation of club and ciliated cells in the adult lung [14, 15]. We also identified a club cell sub-population in human lung that highly express SCGB3A2, with less expression of SCGB1A1 compared to the MUC5B^+^ club cells. Although the proportion of the SCGB1A1^+^ SCGB3A2^high^ club cells is not different, the molecular functions of this club cell population is significantly altered in the IPF lung. In IPF, the SCGB1A1^+^ SCGB3A2^high^ club cells may directly contribute to the extracellular matrix (ECM) accumulation by increased expression of matrix metalloproteinase (MMP) and laminin. The high expression of inhibitor of MMP in the SCGB3A2^high^ club cells may contribute to the complex regulation of ECM in IPF. In addition, SCGB3A2^high^ club cells in IPF expressed many fibrotic growth factors, such as C3, MDK and SPP1 [37–39]. Interestingly, like the MUC5B^+^ club cells, the SCGB3A2^high^ club cells in the IPF lung also express many mucin-related genes, but with a different repertoire, suggesting the heterogeneity of goblet cell-like club cells in the IPF.

In a recent preprint paper from Haberman et al. [40], single-cell data from nonfibrotic controls and pulmonary fibrosis samples, including IPF, chronic hypersensitivity pneumonitis, nonspecific interstitial pneumonia, sarcoidosis and unclassifiable interstitial lung disease, were evaluated to identify four secretory cell phenotypes: SCGB1A1^+^MUC5B^+^MUC5AC^+^ cells; SCGB1A1^+^MUC5B^+^ cells; SCGB1A1^+^SCGB3A2^+^ cells and SCGB3A2^+^ only cells. The proportion of SCGB3A2^+^ only cells was increased and widespread in the cystic and fibrotic lesions of pulmonary fibrosis tissue sections. In our current study which only focused on IPF, we found that the SCGB1A1^+^SCGB3A2^+^ cells were presented in both control and IPF, and SCGB1A1^+^MUC5B^+^ cells were mostly contributed by the IPF patients. However, the SCGB1A1^+^MUC5B^+^MUC5AC^+^ and SCGB3A2^+^ only cells were not detected in our study, suggesting these two secretory cell populations might occur in other types of non-IPF pulmonary fibrosis than IPF. In addition, consistent with the findings by Haberman et al [40], we also identified an alveolar cell population highly expressing SCGB3A2. These cells likely contribute to the pathogenesis of IPF by high expression of extracellular matrix-related genes. In conclusion, the single cell transcriptomic analysis reveals the cellular and molecular heterogeneity of club cells, and provide novel insights into the biological functions of club cells in the pathogenesis of IPF.

## Supporting information

S1 File (PDF)
