# Supplementary material for: Dysregulation of club cell biology in idiopathic pulmonary fibrosis
Source: PLoS One. 2020 Sep 17;15(9):e0237529. doi: 10.1371/journal.pone.0237529 (PMC7498242; doi:10.1371/journal.pone.0237529)

Supplemental Table I. Demographics of control donors and IPF patients[1]

Donors	Age	Sex	Race	Smoking Status	Cause of Death
	63	Female	African american	Never	Stroke
	55	Male	Asian	Former	Intracranial hemorrhage
	29	Female	African american	Never	Anoxic brain injury
	57	Female	African american	Never	Anoxic brain injury
**IPF**	**Age**	**Sex**	**History of smoking**	**Supplemental oxygen use prior to transplant**	
	66	Male	Yes	Yes	
	60	Male	No	Yes	
	68	Male	Yes	Yes	
	72	Female	No	Yes	

[1]Described by Reyfman et al.

Supplemental Table II. Signature genes for the cell populations in human lung of control donors and IPF patients

Cluster 1 (airway and alveolar epithelial cells)		Cluster 2 (ciliated cells)		Cluster 3 (type II alveolar cells-1)		Cluster 4 (type II alveolar cells-2)		Cluster 5 (type II alveolar cells-3)		Cluster 6 (immune cells)		Cluster 7 (plasma cells)	
Gene symbol	Adjusted p values	Gene symbol	Adjusted p values	Gene symbol	Adjusted p values	Gene symbol	Adjusted p values	Gene symbol	Adjusted p values	Gene symbol	Adjusted p values	Gene symbol	Adjusted p values
SCGB3A1	0	CAPS	0	SFTPD	0	SFTPC	0	SFTPA2	0	TPSAB1	0	IGKC	0
SCGB1A1	0	TPPP3	0	SFTA2	0	SLPI	0	SFTPA1	0	CCL5	0	IGLC2	0
SCGB3A2	0	C9orf24	0	PGC	0	CXCL2	0	FGG	0	LTB	0	IGHG3	0
CEACAM6	0	TMEM190	0	SFTPC	0	TM4SF1	0	PGC	0	IL32	0	IGHG4	0
S100A2	0	FAM183A	0	SLPI	0	CLDN18	0	LRRK2	0	TRAC	0	IGHG1	0
AGER	0	C1orf194	0	HOPX	0	SFTA2	0	C4BPA	0	CORO1A	0	IGLC3	0
CAV1	0	C20orf85	0	RNASE1	0	SFTPB	0	PIGR	0	CD2	0	IGHA1	0
KRT7	0	LRRIQ1	0	CYB5A	0	PEBP4	0	HHIP	0	CD3D	0	JCHAIN	0
EMP2	0	SCGB1A1	0	NAPSA	0	NAPSA	0	SFTPB	0	MS4A1	0	IGHM	0
KRT19	0	DNAAF1	0	PLA2G1B	0	SFTPA2	0	LAMP3	0	TRBC2	0	IGHGP	0
GPRC5A	0	RSPH1	0	FGGY	0	HOPX	0	NAPSA	0	CD7	0	MZB1	0
C19orf33	0	CETN2	0	SFTPB	0	PEG10	0	SFTPC	0	CD79A	0	IGHG2	0
MMP7	0	TSPAN1	0	MGST1	0	GKN2	0	DUSP6	0	CD79B	0	SSR4	0
TACSTD2	0	C5orf49	0	AK1	0	SFTPA1	0	CXCL17	0	TRBC1	0	HERPUD1	0
ADIRF	0	SNTN	0	SLC34A2	0	MUC1	0	TFPI	0	RAC2	0	FKBP11	0
WFDC2	0	CCDC146	0	SFTA3	0	MGST1	0	TACC2	0	LIMD2	0	XBP1	0
KRT17	0	CD24	0	SEPP1	0	SFTPD	0	DHCR24	0	CXCR4	0	DERL3	0
KRT15	0	MS4A8	0	S100A14	0	SLC34A2	0	LPCAT1	0	ARHGDIB	0	CD79A	0
CCND1	0	CCDC170	0	PEBP4	0	CTSH	0	C8orf4	0	CD3E	0	PIM2	0
FXYD3	0	C11orf88	0	CTSH	0	SDR16C5	0	ADGRF5	0	ISG20	0	PRDX4	0
SDC1	0	PIFO	0	ABCA3	0	DSTN	0	TTN	0	CD37	0	PRDM1	0
LMO7	0	MORN2	0	DBI	0	PLA2G1B	0	WIF1	0	CD48	0	ITM2C	0
CAV2	0	DNAH12	0	C8orf4	0	KRT8	0	SLC39A8	0	CD3G	0	SEC11C	0
CD55	0	C9orf116	0	MUC1	0	TTN	0	SLPI	0	RPL13A	0	FCRL5	0
CTSE	0	DYNLRB2	0	SFTPA1	0	AGR3	0	SLC34A2	0	ETS1	0	CPNE5	0
SCEL	0	OMG	0	TACC2	0	KRT18	0	PARM1	0	ACAP1	0	POU2AF1	0
CD24	0	RP11-356K	0	CXCL17	0	LMO7	0	PLA2G1B	0	CD27	0	TNFRSF17	0
CYP4B1	0	ERICH3	0	SPTSSA	0	C4BPA	0	MACROD2	0	RPL3	0	JSRP1	0
LCN2	0	DNAH5	0	RPS4Y1	0	CXCL17	0	MALL	0	RPS18	0	HSP90B1	0
TSPAN13	0	CEP126	0	SFTPA2	0	EMP2	0	PEBP4	0	CLEC2D	0	IGHA2	1.49E-302
FMO2	0	C12orf75	0	PMM1	0	NGFRAP1	0	MGST1	0	RPS3	0	SPAG4	1.08E-295
SOX4	0	TUBB4B	0	TSTD1	0	GPRC5A	0	XIST	0	RPL10	0	RGS1	9.36E-287
KRT18	0	SMIM22	0	ID1	0	LAMP3	0	MFSD2A	0	RPSA	0	SELK	4.18E-269
MYL9	0	FOXJ1	0	TMEM97	0	LDLR	0	SDC4	0	RPL13	0	CD27	1.18E-265
BCAM	0	CCDC173	0	CACNA2D	0	RNASE1	0	RNF145	0	RPL18A	0	IGKV1-12	1.92E-265
PERP	0	CDHR3	0	MALL	0	ELF3	0	MTUS1	0	TRAF3IP3	0	SLAMF7	4.9E-265
SFN	0	FAM229B	0	LPCAT1	0	DHCR24	0	MSMO1	0	LCK	0	DNAJB9	1.07E-258
KRT8	0	BBOF1	0	RP11-259K	0	TACC2	0	CADM1	0	BTG1	0	FKBP2	3.43E-248
ZFP36L1	0	ENKUR	0	DCXR	0	SFTA3	0	ST3GAL5	0	RPS27	0	ISG20	8.02E-245
CLIC5	0	AC013264.	0	FASN	0	AGR2	0	CTSH	0	RHOH	0	MEI1	5.48E-238
CAPN2	0	RP11-295M	0	NGFRAP1	0	EGR1	0	HMGCS1	0	PIK3IP1	0	SPCS2	4.98E-224
SEPW1	0	TMC5	0	SDR16C5	0	BMP2	0	MUC1	0	RPS27A	0	PDK1	2.79E-222
LAMB3	0	MRPS31	0	LAMP3	0	FASN	0	SFTA2	0	RPS2	0	VIMP	3.53E-204
PTPRF	0	CFAP53	0	C16orf89	0	LPCAT1	0	DRAM1	0	HNRNPA1	0	IGLV3-1	8.62E-201
ANXA3	0	EFHC1	0	GKN2	0	C16orf89	0	PCDH9	0	ICAM3	0	ST6GAL1	9.43E-183
TM4SF1	0	SPAG17	0	DUOX1	0	HSD17B6	0	SFTPD	0	RCSD1	0	SDF2L1	2.92E-180
FOLR1	0	ODF3B	0	FAM177A1	0	ICAM1	0	MALAT1	0	RPLP2	0	PNOC	8.13E-174
ITGA3	0	CFAP43	0	LL22NC03-	0	PGC	0	SCD	0	RPL23A	0	ERLEC1	1.73E-173
SDPR	0	STK33	0	LRRK2	0	RGS16	0	ZNF385B	0	VPREB3	0	KIAA0125	3.8E-166
NEDD4L	0	FAM216B	0	CLDN18	0	CEBPD	0	WSB1	0	RPS29	0	FAM46C	7.35E-166
CDH1	0	ODF2L	0	ZNF385B	0	NFKBIA	0	SDR16C5	0	IKZF3	0	TXNDC5	2.65E-165
NKX2-1	0	CFAP157	0	AZGP1	0	STEAP4	0	ETV1	0	RPS6	0	CD79B	1.09E-160
EPCAM	0	SLC44A4	0	SELENBP1	0	HMGCS1	0	GOLGA4	0	RPS14	0	MANF	3.82E-159
AGRN	0	EFCAB1	0	PRDX5	0	CADM1	0	MAL2	0	EEF1D	0	IGLV6-57	4.7E-159
MDK	0	IGFBP7	0	PEG10	0	RNF145	0	SOD3	0	RPL32	0	IGKV4-1	1.99E-156
CRIP2	0	AGR3	0	F3	0	C8orf4	0	CEBPD	0	RPS15A	0	PLPP5	3.81E-155
SCNN1B	0	NEK5	0	C14orf1	0	FGGY	0	FASN	0	RPLP1	0	RP11-16E1	7.32E-149
ITGA2	0	HYDIN	0	BEX2	0	NPC2	0	PDE4D	0	RPL10A	0	IGKV3-11	6.48E-148
GAS6	0	C2orf40	0	MFSD2A	0	SELENBP1	0	IRX3	0	RPL27A	0	CRELD2	4.6E-147
KLK11	0	DYDC2	0	NPC2	0	MACROD2	0	MID1IP1	0	RPL18	0	RRBP1	2.24E-143
CP	0	TUBA1A	0	ALOX15B	0	WIF1	0	SECISBP2I	0	PTPN7	0	SEL1L	1.25E-138
EPS8L2	0	GSTA1	0	MAOA	0	SEPP1	0	AQP3	0	RPL19	0	IGKV1-39	5.01E-136
S100A16	0	LDLRAD1	0	RPL24	0	AREG	0	ITGB6	0	SEPT1	0	SPCS3	4.71E-134
FLRT3	0	ZMYND10	0	MBIP	0	SDC4	0	LDLR	0	RPS15	0	HIST1H2B	3.33E-132
EHD2	0	KIF9	0	DRAM1	0	MSMO1	0	ARFGEF3	0	RPL30	0	RAB30	3.71E-131
DSP	0	WDR66	0	SFTA1P	0	LRRK2	0	DLC1	0	RPS25	0	TSC22D3	8.83E-130
SEMA3B	0	CCDC78	0	SDC4	0	MALL	0	ATP1B1	0	RPL11	0	TXNDC15	8.59E-128
RBMS3	0	PRDX5	0	WFDC2	0	DLC1	0	SELENBP1	0	RPL4	0	ZBP1	4.82E-127
LAMA5	0	ELF3	0	LMO3	0	KRT19	0	ATP11A	0	RPS5	0	LAX1	1.54E-126
AQP5	0	DYNLL1	0	FDPS	0	FAM177A1	0	NTN4	0	RPS7	0	NUCB2	7.94E-122
JUP	0	SPATA18	0	CKB	0	SCD	0	MAOA	0	GNB2L1	0	IGKV3-15	9.65E-121
ERBB2	0	TCTEX1D2	0	WIF1	0	ADGRF5	0	YAP1	0	RPS3A	0	IGKV3-20	9.91E-121
SYNPO	0	TSPAN19	0	HHIP	0	WBP5	0	ACSL4	0	RPL15	0	SEL1L3	5.15E-120
SCD5	0	CHST9	0	NNMT	0	FDPS	0	MT-ATP8	0	RPL29	0	IGLC7	1.28E-116
TSPAN1	0	CLU	0	SOD3	0	C11orf96	0	ATP1A1	0	RPL21	0	IGHV3-23	1.59E-116
CDH3	0	METTL7A	0	MACROD2	0	ZFP36	0	NUP58	0	RPS4X	0	CYBA	4.02E-114
AQP4	0	RP1	0	PIGR	0	SLC39A8	0	MLPH	0	RPL36	0	CD38	2.42E-110

Cluster 8 (monocytes-1)		Cluster 9 (monocytes-2)		Cluster 10 (alveolar macrophages-1)		Cluster 11 (alveolar macrophages-2)		Cluster 12 (alveolar macrophages-3)		Cluster 13 (alveolar macrophages-4)		Cluster 14 (mesenchymal cells)	
Gene symbol	Adjusted p values	Gene symbol	Adjusted p values	Gene symbol	Adjusted p values	Gene symbol	Adjusted p values	Gene symbol	Adjusted p values	Gene symbol	Adjusted p values	Gene symbol	Adjusted p values
S100A8	0	SPP1	0	VIM	2.39E-121	INHBA	0	INHBA	0	C1QA	0	SPARCL1	0
S100A9	0	CCL2	0	HNRNPK	3.085E-86	THBS1	0	FABP4	0	C1QB	0	MGP	0
FCN1	0	CTSB	0	RHOA	1.635E-80	MS4A7	0	C1QB	0	C1QC	0	IGFBP7	0
S100A12	0	CTSL	0	OSBPL8	3.239E-78	OLR1	0	CD52	0	OLR1	0	TIMP3	0
VCAN	0	C15orf48	0	CIR1	2.331E-77	MSR1	0	SNX10	0	RETN	0	CLDN5	0
LST1	0	APOE	0	NBEAL1	2.247E-75	ALOX5AP	0	DEFB1	0	HLA-DQA1	0	TAGLN	0
AIF1	0	CHI3L1	0	GNAS	1.183E-74	RP11-1143	0	AKR1C3	0	HLA-DRB1	0	A2M	0
CORO1A	0	CTSZ	0	PABPC1	1.845E-74	MRC1	0	ALOX5AP	0	GCHFR	0	VWF	0
RGS2	0	GPNMB	0	YWHAB	2.658E-74	SNX10	0	SERPING1	0	MARCO	0	ACKR1	0
FGL2	0	LILRB4	0	LYZ	1.435E-73	MCEMP1	0	LYAR	0	APOC1	0	MT1M	0
MS4A6A	0	TGFBI	0	AP1S2	1.52E-71	FOLR3	0	CD163	0	TREM1	0	MT2A	0
LYZ	0	PSAP	0	PCBP1	2.8E-71	PLIN2	0	MS4A7	0	TYROBP	0	CALD1	0
SRGN	0	LGMN	0	C5AR1	5.034E-71	CSTA	0	MSR1	0	FBP1	0	CLU	0
TIMP1	0	LGALS1	0	RP11-1143	4.951E-69	ALOX5	0	CXCL3	0	ALOX5AP	0	MT1X	0
MNDA	0	LIPA	0	CMTM6	8.598E-69	FABP4	0	PLIN2	0	CD52	0	CTGF	0
COTL1	0	TIMP1	0	GLIPR1	8.836E-69	FCGR2A	0	FN1	0	VSIG4	0	POSTN	0
CD14	0	CYBB	0	GABARAP	9.098E-68	PSMA7	0	C1QA	0	MCEMP1	0	GNG11	0
RNASE6	0	IL1RN	0	STK17B	2.503E-67	EVI2B	0	IFI6	0	S100A4	0	EPAS1	0
FAM26F	0	CCL18	0	NAMPT	5.661E-67	LYAR	0	HLA-DRB5	0	HLA-DQB1	0	MYL9	0
TYMP	0	EMP1	0	DAZAP2	2.773E-66	GLUL	0	CCL18	0	CD68	0	ADIRF	0
LGALS2	0	CD14	0	MCL1	9.547E-63	RASGEF1E	0	RND3	0	FCGRT	0	TCF4	0
C10orf54	0	TYMP	0	MS4A6A	6.097E-62	PPARG	0	PSMA7	0	ACP5	0	GPX3	0
ACTB	0	PLA2G7	0	PRPF38B	6.555E-62	LGALS3	0	VSIG4	0	FTH1	0	FAM107A	0
GAPDH	0	CHIT1	0	PRRC2C	2.736E-61	ARL4A	0	ISG15	0	HLA-DRB5	0	EGFL7	0
CST3	0	MAFB	0	TRA2B	5.444E-61	CCDC88A	0	ACP5	0	CTSD	0	CALCRL	0
LSP1	0	MMP9	0	TMEM167	9.967E-61	CXCR4	0	MRC1	0	CTSC	0	SPARC	0
FYB	0	CD84	0	ARF6	1.801E-59	APOC1	0	OLR1	0	HLA-DMA	0	ADAMTS1	0
CPVL	0	ITGB2	0	TNFRSF1B	1.989E-59	MARCO	0	CTSC	0	HLA-C	0	CAV1	0
AP1S2	0	SH3BGRL3	0	ZEB2	6.427E-59	HLA-DRA	0	CSTA	0	GSTO1	0	PRSS23	0
CFP	0	FN1	0	SGK1	3.381E-58	FTH1	0	APOC1	0	TSPO	0	IFITM3	0
ARHGDIB	0	FPR3	0	SAMHD1	3.523E-58	VSIG4	0	HLA-DQB1	0	FTL	0	C7	0
CD48	0	CTSD	0	GPR183	4.884E-58	SLC11A1	0	GLUL	0	PYCARD	0	C10orf10	0
GLIPR1	0	TREM2	0	SSR1	1.77E-57	CTSC	0	STXBP2	0	S100A11	0	PTRF	0
ITGB2	0	SRGN	0	RTN4	2.028E-57	BCL2A1	0	GLDN	0	GRN	0	ID3	0
PTPRC	0	SGK1	0	CDC42	8.928E-57	CD163	0	HLA-DRA	0	C1orf162	0	RAMP2	0
MPEG1	0	CCL3	0	GDI2	1.461E-56	SDCBP	0	GRN	0	HLA-DPB1	0	MMRN1	0
LGALS1	0	SDC2	0	C20orf24	4.177E-56	C1QB	0	MCEMP1	0	HLA-DPA1	0	CRIP2	0
CYBA	0	MARCKS	0	RPL17	4.549E-56	ACP5	0	FCER1G	0	FABP3	0	PTPRB	0
SH3BGRL3	0	PLEK	0	IQGAP1	5.687E-56	FCER1G	0	HLA-DRB1	0	CFD	0	PECAM1	0
TMSB10	0	CSTB	0	CEBPB	8.876E-56	VIM	0	TREM1	0	SLC11A1	0	IFITM2	0
TYROBP	0	EMP3	0	RNF130	1.016E-55	FTL	0	BHLHE41	0	HLA-DRA	0	TPM1	0
RGS18	0	VIM	0	CD164	1.162E-55	TYROBP	0	GCHFR	0	FCER1G	0	SDPR	0
H3F3A	0	ACTB	0	HCLS1	8.368E-55	HPGD	0	THBS1	0	HLA-DMB	0	CLEC14A	0
RPS24	0	CTSS	0	OGFRL1	8.622E-55	LYZ	0	C1QC	0	RP11-598F	0	PCAT19	0
RPL10	0	CAPG	0	RALA	7.594E-54	RNF149	0	HPGD	0	EVI2B	0	CD93	0
RPL28	0	IGSF6	0	IL10RB	1.98E-53	C1QA	0	MARCO	0	CAPG	0	SLCO2A1	0
UBA52	0	CTSK	0	BTG1	2.704E-53	LY96	0	SDCBP	0	GLRX	0	IGFBP4	0
RPL26	0	GM2A	0	PGK1	2.353E-52	ALDH2	0	RGCC	0	PLIN2	0	HSPG2	0
LIMD2	0	CD68	0	MFSD1	4.609E-52	CD52	0	FTH1	0	TMSB4X	0	LDB2	0
GPR183	0	ANXA2	0	CHMP1B	5.17E-52	TXNIP	0	PPARG	0	LYZ	0	NPDC1	0
HCLS1	0	RP11-1143	0	PTPRE	2.08E-51	TCF7L2	0	ALDH2	0	RBP4	0	ENG	0
RPL18A	2.37E-303	CCR1	0	TPP1	6.804E-51	MTPN	0	LGALS3	0	SERPING1	0	RAMP3	0
RPL13A	1.68E-301	ABCA1	0	FGL2	8.954E-51	IRS2	0	CES1	0	CD74	0	PCDH17	0
CLEC10A	2.58E-298	SOD2	0	HMOX1	5.038E-50	TSPO	0	SLC11A1	0	CES1	0	HYAL2	0
RPS3A	4.55E-298	AIF1	0	CD36	9.866E-50	ATP5EP2	0	HLA-DQA1	0	CXCL16	0	SLC9A3R2	0
RGS10	1.18E-297	FYB	0	ARPC5	3.417E-49	HLA-DPA1	0	TSPO	0	LGALS3	0	ADAMTS9	0
TMSB4X	1.88E-297	TMSB10	0	FAM133B	4.767E-48	CORO1C	0	CD74	0	GPX1	0	COX7A1	0
SERPINB9	1.75E-293	PLAUR	0	FYB	9.766E-48	ZFAS1	0	LPL	0	ITM2B	0	CRIM1	0
PFN1	4.37E-290	HCST	0	GRB2	1.602E-47	LTA4H	0	BCL2A1	0	CYP27A1	0	VAMP5	0
IL1R2	3.74E-289	APOC1	0	LGALS3	2.866E-47	CD74	0	RASGEF1E	0	LRPAP1	0	IL33	0
CYBB	1.29E-288	COTL1	0	ETS2	4.632E-47	SERPING1	0	LY96	0	CXCL3	0	SNCG	0
CSF1R	1.8E-287	MS4A6A	0	LCP1	4.742E-47	ARL6IP1	0	ALOX5	0	AKR1B1	0	ADGRL4	0
IFI30	1.07E-282	AP2S1	0	VMP1	7.9E-47	ADAM17	0	FTL	0	CYBA	0	NFIB	0
LILRB2	1.41E-281	CFL1	0	ARPC1B	1.447E-46	DAB2	0	S100A11	0	ATP6V1F	0	ARHGAP2	0
RPL32	1.11E-280	LAPTM5	0	DDX3X	1.71E-46	MPHOSPH	0	CCDC88A	0	STXBP2	0	PALMD	0
CFL1	2.56E-280	FGL2	0	IFNGR1	1.871E-46	UBB	0	ARL4A	0	SERPINA1	0	PLAC9	0
LCP1	2.46E-279	FTL	0	C6orf62	5.131E-46	LRRFIP1	0	FBP1	0	UBB	0	S1PR1	0
PRELID1	3.94E-276	GSN	0	ATP13A3	7.757E-46	HLA-DPB1	0	S100A4	0	ALDH2	0	CCDC85B	0
CD93	1.01E-269	CALM3	0	ATP5EP2	9.594E-46	SNHG12	0	UBB	0	CSTA	0	NUAK1	0
PKIB	2.67E-266	RGS1	0	ZFP36L2	1.471E-45	CTSS	0	HLA-DPB1	0	AIF1	0	EMCN	0
RPL13	1.81E-263	LCP1	0	YWHAH	1.941E-45	GCHFR	0	GPNMB	0	GMFG	0	SRPX	0
TNFRSF1B	4.76E-262	LAP3	0	MAFB	2.039E-45	ITM2B	0	CTSD	0	CD37	0	PLVAP	0
RPS15A	1.62E-260	LYZ	0	CD44	2.668E-45	CTNNB1	0	APOE	0	DOK2	0	TINAGL1	0
CD1E	2.08E-260	FABP5	0	WIPF1	2.884E-45	LAPTM5	0	ITM2B	0	HN1	0	MMRN2	0
VIM	2.74E-256	TPM4	0	RGS2	2.911E-45	RPL17	0	ADAM17	0	LAPTM5	0	MCAM	0
GMFG	3.7E-255	TPM3	0	CLEC7A	7.069E-45	HLA-DQB1	0	EVI2B	0	LY6E	0	CYYR1	0
RPS7	3.38E-254	ZEB2	0	PSMA7	2.235E-44	CD164	0	RP11-598F	0	ALDOA	0	CD34	0

Cluster 1 (airway and alveolar epithelial cells)		Cluster 2 (ciliated cells)		Cluster 3 (type II alveolar cells-1)		Cluster 4 (type II alveolar cells-2)		Cluster 5 (type II alveolar cells-3)		Cluster 6 (immune cells)		Cluster 7 (plasma cells)	
Gene symbol	Adjusted p values	Gene symbol	Adjusted p values	Gene symbol	Adjusted p values	Gene symbol	Adjusted p values	Gene symbol	Adjusted p values	Gene symbol	Adjusted p values	Gene symbol	Adjusted p values
IRX2	0	ROPN1L	0	TMEM245	0	SOCS3	0	ALPL	0	RPL14	0	SPCS1	3.12E-110
CLIC6	0	AKAP9	0	CPB2	0	MT1E	0	CXADR	0	RPS23	0	IGLL5	6.39E-109
PAM	0	DYNC2H1	0	RPS12	0	LIMCH1	0	NFIC	0	SIT1	0	HSH2D	3.55E-108
DDR1	0	FAM92B	0	ATP1A1	0	ATP1A1	0	PTPN13	0	RPL7	0	TMEM156	5.47E-107
ST6GALN	0	RABL2B	0	TFPI	0	MBIP	0	RHOBTB2	0	RPS19	0	UBE2J1	5.49E-107
SCNN1A	0	HMGN3	0	PTPN13	0	FOSB	0	CRTAC1	0	RPL35A	0	TPD52	1.65E-106
ELF3	0	CAPSL	0	DLC1	0	CYB5A	0	SYNE1	0	RPL35	0	SUB1	9.53E-105
PLXNB2	0	IGFBP5	0	CSF3R	0	S100A14	0	SNX25	0	RPS8	0	DDIT4	1.54E-104
C1orf116	0	IGFBP2	0	RHOBTB2	0	DUOX1	0	ARRDC3	0	RPL41	0	AC006129.	4E-102
SYNE2	2.52E-308	MNS1	0	IDI1	0	ZNF385B	0	MECOM	0	HLA-A	0	SPATS2	1.45E-99
ANOS1	3.94E-307	DZIP3	0	NDUFA4	0	MAL2	0	HOPX	0	SEPT6	0	IL16	9.15E-99
PLPP2	3E-306	SAXO2	0	ID4	0	F3	0	NPC2	0	DDIT4	0	MEF2C	3.17E-96
TRIM2	8.4E-306	TMEM231	0	RPS3	0	C19orf33	0	KCNJ15	0	RPS21	0	CREB3L2	1.545E-95
SFTA1P	1.14E-305	CCDC17	0	DUOXA1	0	EZR	0	INSIG1	0	FAU	6.68E-307	FBXW7	4.867E-92
TMC5	1.51E-303	PSENEN	0	TMEM125	0	PCDH9	0	AHCYL2	0	HIST1H4C	4.08E-304	LMAN1	1.476E-91
TMEM98	3.03E-297	CDHR4	0	GSTA4	0	MLPH	0	CCDC141	0	RPL26	4.34E-303	TP53INP1	3.835E-91
PLS3	6.95E-297	C11orf97	0	NRGN	0	ATP1B1	0	COBLL1	0	TPSB2	7.67E-302	ANKRD28	7.332E-90
KLF5	7.29E-297	ALDH3B1	0	AGR2	0	SFN	0	FNIP2	0	CD247	2.45E-297	EAF2	8.258E-88
ECE1	4.36E-295	DRC3	0	DHCR24	0	CEACAM6	0	SNX30	0	HLA-E	8.75E-295	CTA-292E1	3.33E-87
ITGB4	5.47E-295	POLR2I	0	SH3YL1	0	EBP	0	JUND	0	LAT	3.2E-293	GNG7	2.04E-85
ERBB3	2.44E-294	RFX3	0	C1orf21	0	SYNE1	0	GLS	0	PTPRC	3.58E-292	RALGPS2	1.212E-84
FHL2	3.05E-292	KIF21A	0	ADI1	0	DRAM1	0	C1orf21	0	TBC1D10C	1.27E-287	LINC00152	2.028E-84
NEDD9	1.7E-290	CATSPERI	0	FMO5	0	CLDN4	0	MBIP	0	SELL	1.59E-285	AL928768.	1.145E-78
CLDN1	5.13E-287	MORN5	0	AKAP13	0	SOD3	0	FHDC1	0	PSMB9	3.02E-284	LINC01480	2.133E-74
RTKN2	7.86E-287	SPEF2	0	SLC25A5	0	FLRT3	0	IL6ST	0	GZMM	4.97E-275	CPEB4	3.325E-74
FAT1	9.29E-286	CDS1	0	HHIP-AS1	0	AK1	0	IDI1	0	BIRC3	8.68E-272	DNAJC1	7.701E-74
CLU	1.7E-285	RP4-666F2	0	LIMCH1	0	MID1IP1	0	LGALSL	0	BANK1	3.83E-271	ICAM3	4.382E-70
LMO3	5.46E-285	FHAD1	0	SLCO4C1	0	MAOA	0	DSTN	0	RPL28	1.48E-270	POU2F2	6.015E-69
DSTN	8.93E-284	DNAH7	0	COX6C	0	MFSD2A	0	NEAT1	0	RPL34	2.34E-269	SDC1	5.369E-68
KRT5	9.59E-284	ZBBX	0	POLR2L	0	JUND	0	SGMS2	0	CST7	6.9E-268	CHPF	2.351E-65
EZR	6.24E-283	IQCG	0	GSTP1	0	EPCAM	0	GPR160	0	EEF1B2	4.31E-267	SEC14L1	1.142E-63
MICAL2	7.99E-283	CFAP70	0	PEBP1	0	FXYD3	0	PTP4A1	0	RPL8	3.16E-266	TBCEL	1.02E-62
IL32	1.09E-281	WDR60	0	SECISBP2I	0	IFT57	0	BTG3	0	IL2RG	3.53E-266	CTA-384D	3.087E-59
MAL2	1.95E-281	CLIC6	0	VEPH1	0	ABCA3	0	TSPAN3	0	EIF1AY	7.1E-266	SSR3	3.617E-59
GJA1	5.39E-281	CFAP126	0	CEBPD	0	PARM1	0	PRKCI	0	GLTSCR2	3.33E-265	IRF4	6.113E-59
MYH14	1.41E-278	SPAG6	0	COX14	0	NKX2-1	0	PXMP4	0	NKG7	7.37E-265	TXNDC11	3.931E-54
SFTA3	2.54E-277	PFN2	0	CHI3L2	0	TFPI	0	KIAA1324I	0	KLRB1	1.31E-262	NXPE3	1.388E-50
NDNF	3.37E-277	DNALI1	0	MYLK	0	MTUS1	0	CERS2	0	CTSW	3.6E-260	LARP1B	9.779E-50
LAMC2	5.69E-277	SPAG16	0	PLA2G16	0	CYBRD1	0	CAT	0	B2M	2.74E-259	FUT8	4.71E-49
DHCR24	7.53E-277	SPA17	0	HSD17B6	0	SCGB3A2	0	ANK3	0	TSC22D3	2.97E-258	RABAC1	7.428E-49
DMKN	9.36E-277	CP	0	C2	0	BTG3	0	C2	0	ANXA6	3.43E-258	PABPC4	3.404E-47
CLDN4	2.58E-276	C9orf135	0	ELF3	0	C1orf21	0	THOC2	0	IL7R	1.35E-257	DUSP5	2.918E-44
TMEM63B	4.23E-276	EZR	0	RPL18	0	SUSD2	0	SPINT2	0	AC092580.	3.69E-257	PDIA6	9.53E-44
FSTL1	4.78E-275	C21orf58	0	MRPL14	0	MSLN	0	RDH11	0	HIST1H1D	5.55E-257	IFNAR2	1.919E-43
CX3CL1	3.03E-274	ARMC3	0	HMGCS1	0	TSC22D1	0	AKAP13	0	ACTB	1.99E-254	SRPRB	2.209E-43
TIMP3	1.39E-273	FAM227A	0	CA2	0	CLDN7	0	IRF2BP2	0	FXYD5	1.16E-251	TMEM258	8.903E-43
PTK7	2.68E-273	PRR29	0	PLA2G10	0	KRT7	0	NFIX	0	PYHIN1	1.41E-248	CCDC167	7.65E-42
EFNA1	1.54E-272	DTHD1	0	MAL2	0	SNX25	0	MYO6	0	SKAP1	1.43E-247	SYVN1	2.141E-41
SUSD2	4.79E-271	RP11-128M	0	TMEM141	0	SQLE	0	FAM177A1	0	CD96	2.82E-246	ITGA6	5.761E-40
CTTN	7.19E-271	HSP90AA1	0	ETV1	0	ITGB6	0	ASPH	0	GZMA	2.98E-246	MANEA	5.911E-39
MUC4	3.3E-269	PPIL6	0	TTN	0	WFDC2	0	PRKAR1A	0	RP11-796E	2.38E-244	BTG2	1.458E-38
NFIB	4.87E-268	TEKT1	0	SLC25A4	0	AQP4	0	TMEM87A	0	RGS13	5.24E-244	HM13	2.734E-38
ICAM1	6.78E-268	IK	0	PCBD1	0	TSPAN13	0	CYB5A	0	KIAA1551	2.05E-243	PTP4A3	5.688E-36
MS4A15	9.82E-268	RP11-620J	0	SNX30	0	YAP1	0	TOP1	0	RPL37	2.62E-241	SELM	1.821E-35
C1R	1.16E-267	CCDC153	0	CKS2	0	FOLR1	0	SPAG9	0	STK4	1.93E-240	RP11-138A	2.181E-34
HMCN1	4.12E-266	TUBA4B	0	RPS6	0	AKAP13	0	ZNF106	0	RPS4Y1	4.08E-240	PDIA4	2.485E-34
CD151	4.67E-266	UFC1	0	DDT	0	NEAT1	0	WWC3	0	CD69	3.31E-238	MALAT1	1.946E-33
EGFR	1.88E-265	ATPIF1	0	ADGRF5	0	TMEM97	0	CFLAR	0	STK17A	3.97E-238	TMED9	2.099E-33
AQP3	2.09E-265	TNFAIP8L	0	EBP	0	ATP11A	0	TMEM243	0	SPOCK2	8.37E-238	GLCCI1	2.693E-33
PAWR	3.27E-265	FAM81B	0	IRX3	0	ACAT2	0	HSD17B12	0	BATF	2.78E-237	HSPA13	1.497E-32
APP	9.48E-265	IFT27	0	RNF145	0	SCNN1A	0	GLO1	0	RPL6	1.16E-234	HIST1H1C	1.07E-31
SPOCK2	3.47E-263	CFAP73	0	FOLR1	0	SPTSSA	0	TACC1	0	HMGB2	2.59E-230	GNL3	5.955E-31
HOPX	1.06E-262	CCDC113	0	PFKFB2	0	KLF5	0	AHCYL1	0	ITM2A	5.38E-230	SEC61B	1.713E-30
TNC	1.56E-262	DNAJA4	0	CRTAC1	0	TMEM245	0	TTC3	0	ARHGAP1	3.35E-229	H1FX	2.123E-30
ABLIM1	2.07E-262	MAP3K19	0	RPLP0	0	CXADR	0	TCEAL8	0	RPL31	1.65E-228	GMPPB	3.964E-30
RNF145	8.11E-262	CFAP221	0	NECAB1	0	MYO1B	0	LDHB	0	RPS10	2.41E-225	VOPP1	8.629E-30
CNN3	9.64E-262	LRRC23	0	PTP4A1	0	ARFGEF3	0	MAP3K13	0	SUB1	4.48E-225	BCL2L11	3.011E-29
TNNC1	2.53E-261	CSPP1	0	EPCAM	0	CCDC141	0	SMARCA5	0	GPR171	4.55E-225	SIL1	4.999E-29
DSG2	7.45E-261	WDR78	0	ALPL	0	DCXR	0	PTP4A2	0	RPS17	1.64E-224	HSPA5	1.08E-26
WFS1	1.13E-260	WDR38	0	NKX2-1	0	WSB1	0	EI24	0	TIGIT	3.24E-223	CMB9-22P	3.119E-25
PTPRZ1	9.69E-260	DYNLT1	0	ACADL	0	CD59	0	PPA1	0	GAPDH	6.36E-222	TCF4	4.291E-24
SOX2	6.54E-259	LRRC46	0	BLVRB	0	BEX2	0	ERLEC1	0	RPL39	1.87E-221	SSR2	1.053E-23
MYO1B	7.56E-259	CCDC39	0	HSBP1L1	0	IDI1	0	DHRS7	0	ITM2C	5.07E-221	MT1F	1.108E-23
COL17A1	3.5E-254	CIB1	0	NEDD4L	0	TSTD1	0	AIDA	0	CCR7	5.14E-221	TMED2	3.846E-23
CXCL14	1.57E-253	CCDC30	0	SMIM22	0	GLS	0	CPNE3	0	C1orf186	1.02E-220	RP11-386I	3.058E-22
RAB11FIP1	1.86E-253	HSBP1	0	MLPH	0	FHDC1	0	GSPT1	0	RPL7A	1.98E-219	ERN1	7.647E-22
LBH	1.19E-252	IFT22	0	KIF22	0	TMEM41B	0	CD46	0	TNFAIP8	2.03E-219	TRAM1	1.954E-21
NCKAP1	2.88E-252	CFAP100	0	SMIM4	0	SLC25A4	0	GNAQ	0	SYTL3	4.55E-218	DERL1	2.83E-20

Cluster 8 (monocytes-1)		Cluster 9 (monocytes-2)		Cluster 10 (alveolar macrophages-1)		Cluster 11 (alveolar macrophages-2)		Cluster 12 (alveolar macrophages-3)		Cluster 13 (alveolar macrophages-4)		Cluster 14 (mesenchymal cells)	
Gene symbol	Adjusted p values	Gene symbol	Adjusted p values	Gene symbol	Adjusted p values	Gene symbol	Adjusted p values	Gene symbol	Adjusted p values	Gene symbol	Adjusted p values	Gene symbol	Adjusted p values
LAPTM5	1.05E-253	LITAF	0	FOS	5.332E-44	RPS27L	0	CTSS	0	ATP6V0B	0	CDH5	0
FPR1	2.25E-253	LIMS1	0	PPT1	7.574E-44	SH3BGRL	0	CORO1C	0	MS4A7	0	JAM2	0
CD300A	3.24E-253	MS4A4A	0	LRRFIP1	9.521E-44	RAP1B	0	AKR1C2	0	MRC1	0	SELP	0
RPS23	6.34E-253	TNFSF13B	0	SRGAP2B	1.525E-43	S100A11	0	TYROBP	0	CST3	0	SOX18	0
RPLP2	8.5E-253	PRDX1	0	CHD1	1.639E-43	HNMT	0	VIM	0	GLDN	0	LRRC32	0
RPS9	4.16E-252	FCER1G	0	MEF2C	5.182E-43	AZI2	0	CYP27A1	0	TFRC	0	NRN1	0
MYO1F	9.84E-249	TFRC	0	BASP1	6.066E-43	HLA-DQA1	0	GLRX	0	LST1	0	ERG	0
FAM49B	2.23E-248	TYROBP	0	ATP6V1B2	9.053E-43	S100A10	0	KLF4	0	VIM	0	ROBO4	0
RPS27A	1.41E-245	CYBA	0	CPPED1	1.007E-42	HLA-DRB5	0	FCGR2A	0	JAML	0	MYCT1	0
FCER1G	1.5E-242	S100A9	0	SLC31A2	2.081E-42	YBX1	0	S100A10	0	ARPC3	0	HLA-E	0
RPL19	1.76E-242	CD99	0	RGS10	2.719E-42	NUPR1	0	B2M	0	SLC7A7	0	MYH9	0
RPS2	1.85E-242	PKM	0	ATP1B3	2.925E-42	GRINA	0	ATP5EP2	0	VASP	0	CCL14	0
RASSF2	1.01E-240	ITGAX	0	ACTR2	4.197E-42	H2AFY	0	CD68	0	IGSF6	0	APP	0
RNASET2	8.99E-236	ATOX1	0	ZMIZ1	2.259E-41	TAF1D	0	HEXB	0	CLIC1	0	TPM2	0
S100A4	5.19E-235	LHFPL2	0	VCAN	3.319E-41	ARPC1B	0	PAG1	0	HPGD	0	AQP1	0
PRKCB	1.8E-234	STAT1	0	AHNAK	4.163E-41	S100A4	0	LY6E	0	ABCG1	0	LCN6	0
RPS14	3.19E-233	HLA-A	0	LILRB3	4.618E-41	LARP7	0	DAB2	0	APOE	0	TSPAN7	0
C1orf162	1.27E-231	MNDA	0	UBE2R2	5.646E-41	CBX3	0	RPS27L	0	GLIPR2	0	TMTC1	8.3E-308
FAU	3.1E-229	AQP9	0	TPM3	5.872E-41	PRPF38B	0	LINC01272	0	MGST3	0	CXorf36	4.55E-307
CD1C	6.96E-229	PLTP	0	SMAP2	7.807E-41	B2M	0	PCOLCE2	0	HEXB	0	EMP1	4.2E-300
RPS18	7.6E-228	LSP1	0	COTL1	1.206E-40	C6orf48	0	LRRFIP1	0	HSPA1A	0	COL4A1	3.54E-299
RPS6	2.84E-226	RNF130	0	WAC	1.382E-40	ARID5B	0	ANXA1	0	NCF2	0	HEG1	6.42E-299
GPSM3	1.9E-224	UCP2	0	NCOR1	1.477E-40	TGOLN2	0	SPI1	0	PCOLCE2	0	PIK3R3	1.2E-298
RNASE2	2.81E-224	CALR	0	PTEN	2.647E-40	NOP10	0	HLA-DPA1	0	PNPLA6	0	ID1	2E-297
HCST	7.16E-224	TUBB	0	SRGN	7.373E-40	SNRPB2	0	VAT1	0	SPI1	0	COL6A2	1.9E-294
SELL	5.41E-222	ITGAM	0	RBPJ	1.245E-39	TBCA	0	TCF7L2	0	AGRP	0	RASIP1	3.38E-294
RPS15	1.13E-220	S100A10	0	MARCKS	1.541E-39	NBEAL1	0	FHL1	0	FCGR3A	0	TIE1	4.56E-294
TNFSF13B	2.97E-218	NPL	0	MT-ATP6	2.288E-39	RTN4	0	RMDN3	0	CCL18	0	ACTA2	6.34E-293
RPL15	4.49E-217	SLC16A3	0	ZFAND5	3.574E-39	DDX21	0	HLA-B	0	GLUL	0	RUNX1T1	6.6E-292
ARPC3	5.98E-217	HLA-B	0	MT-ATP8	1.497E-38	EML4	0	MTPN	0	VAMP8	0	NES	5.35E-287
LILRA5	6.02E-217	TUBA1B	0	SKAP2	2.436E-38	SEC11A	0	ARL6IP1	0	OAZ1	0	SPRY1	1.48E-286
ENTPD1	8.86E-216	MFSD1	0	DUSP1	3.446E-38	SYF2	0	FAM89A	0	RNASET2	0	CYR61	4.07E-286
HMGB2	1.09E-215	PPT1	0	DPYD	8.554E-38	CST3	0	ANXA5	0	NOP10	0	TMEM204	1.73E-285
JAML	2.37E-215	CD44	0	PTPRC	3.042E-37	SARAF	0	C5AR1	0	PFDN5	0	MSRB3	1.5E-283
GPX1	5E-215	GPX1	0	LMO4	3.109E-37	POMP	0	MGST3	0	ALOX5	0	IL6ST	2.6E-281
HLA-B	3.88E-214	SDS	0	FKBP5	3.878E-37	ARPC3	0	TXN	0	MS4A4A	0	LEPR	1.51E-279
C19orf38	5.49E-214	AHNAK	0	QKI	3.899E-37	PRR13	0	LYZ	0	MYL6	0	RHOJ	1.86E-279
SMAP2	7.12E-209	AP1S2	0	CSF2RA	1.588E-36	TCEB2	0	C1orf162	0	UPP1	0	CD59	4.1E-279
CLEC4A	1.83E-205	KCTD12	0	HLA-DPB1	2.282E-36	ZFAND5	0	RP11-1143	0	C9orf16	0	TNXB	6.68E-279
S100B	1.35E-203	CTSA	0	SLA	3.811E-36	HMGN2	0	CD81	0	PFN1	0	FLT1	3.84E-277
TGFBI	1.67E-203	ATP6AP2	0	CAP1	6.961E-36	MT-ND2	0	SH3BGRL	0	TMSB10	0	BGN	1.7E-276
UCP2	4.21E-203	C5AR1	0	HCK	2.445E-35	ANP32B	0	TXNIP	0	MIR3945H	0	AEBP1	4.15E-276
AOAH	8.67E-202	SAMHD1	0	CLEC2B	3.603E-35	RSRC2	0	RP5-839B4	0	GYPC	0	PODXL	1E-275
NAIP	8.94E-199	FTH1	0	CCR1	4.843E-35	DYNC1I2	0	LAPTM5	0	TCEB2	0	LMCD1	1.55E-275
HCK	1.2E-198	TTYH3	0	SDC2	8.491E-35	DNAJA1	0	HSP90AA1	0	CD81	0	COL18A1	7E-275
MARCH1	1.25E-197	OTOA	0	CASP1	1.365E-34	TAX1BP1	0	NOP10	0	SPN	0	NR2F2	9.51E-275
SPI1	3.17E-197	GAPDH	0	HERPUD1	7.573E-34	CTSD	0	H2AFY	0	MYL12A	0	TM4SF18	9.74E-274
GAPT	1.4E-196	CSF1R	0	C1orf56	1.132E-33	PTPMT1	0	CEBPB	0	ZNF706	0	SOX7	1.99E-272
FAM65B	1.75E-196	CREG1	0	PABPC4	3.866E-33	EIF1	0	CD164	0	GRINA	0	TIMP1	2.25E-272
PTMA	3.83E-194	S100A8	0	IQGAP2	6.285E-33	RWDD1	0	CST3	0	SLCO2B1	0	KLF2	5.05E-271
RPL37	1.28E-193	ATP6V1F	0	MTHFD2	1.172E-32	CSTB	0	HLA-DMA	0	ANXA5	0	TGFBR3	3.17E-269
MAFB	2.62E-192	GBP1	0	SLC8A1	1.345E-32	GPX4	0	YBX1	0	HLA-B	0	PLSCR4	1.02E-267
ADGRE2	1.56E-191	FCGR3A	0	FGR	3.604E-32	UQCRB	0	PSAP	0	BSG	0	ZEB1	1.14E-264
PYCARD	6.08E-190	GLIPR1	0	CLEC4E	6.06E-32	CAPZA2	0	CAPG	0	HNMT	0	PTGIS	1.26E-260
H3F3B	2.79E-189	RALA	0	FAM129A	1.117E-31	RPL39	0	TCEB2	0	CD300LF	0	TEK	2.96E-260
FCER1A	1.56E-187	LILRB2	0	RAP2B	1.338E-31	PPIG	0	ZEB2	0	GPD1	0	RHOC	8.45E-260
CD86	4.41E-187	ATP6V1B2	0	CPVL	1.501E-31	EIF3E	0	CSTB	0	LGALS1	0	LAMA4	6.26E-259
RPL29	3.13E-186	PFN1	0	PLSCR1	2.1E-31	UBA52	0	CTSZ	0	AKR1C3	0	LIFR	1.18E-258
ARPC1B	3.32E-185	HIF1A	0	ZFHX3	2.574E-31	SRRM1	0	LGALS1	0	MSR1	0	COL4A2	2.32E-258
CTSS	2.1E-182	NR1H3	0	NR4A2	2.636E-31	EIF1B	0	CYBA	0	RND3	0	DCN	4.55E-258
RPL11	2.35E-182	CCDC88A	0	CCDC88A	3.415E-31	UQCRH	0	SERPINA1	0	PPARG	0	MT1E	3.43E-255
RPLP1	9.31E-182	ACTR3	0	SLC2A3	5.048E-31	TKT	0	ARPC3	0	PLBD1	0	ZNF385D	1.36E-254
SAMHD1	1.8E-181	CD63	0	TMEM38B	1.192E-30	GPX1	0	AIF1	0	APIP	0	AKAP12	2.54E-253
CD53	1.79E-179	ARPC5	0	MARCH1	2.085E-30	USMG5	0	ANXA2	0	LGALS3BI	0	TMEM100	3.64E-253
EMP3	2.44E-179	CYFIP1	0	MT-ND3	2.154E-30	TMSB10	0	GPX1	0	NUPR1	0	ELN	1.39E-252
GIMAP7	8.68E-179	ACTR2	0	MAP3K1	3.399E-30	ATP5G2	0	TMSB10	0	SH3BGRL3	0	SPTBN1	1.6E-250
POU2F2	5.87E-177	ACTG1	0	CECR1	1.128E-29	YWHAB	0	MT-ND2	0	TBXAS1	0	TM4SF1	5.16E-246
TPM3	2.31E-176	PEA15	0	LILRB4	1.606E-29	PSAP	0	SRGN	0	COMT	0	TGM2	4.29E-245
RGS1	3.17E-176	CECR1	0	LITAF	5.954E-29	PFDN5	0	XIST	0	SH3BGRL	0	ABI3BP	1.02E-242
PAK1	2.63E-175	A2M	0	RASSF3	9.748E-29	HLA-B	0	CTSB	0	RP5-839B4	0	GRASP	9.01E-241
ARPC4	7.02E-175	FCGR2B	0	CKLF	1.836E-28	PCBP1	0	SH3BGRL3	0	MCOLN1	0	RAPGEF4	2.76E-240
RPL27A	3.92E-173	BASP1	0	PLAUR	7E-28	VDAC2	0	ASAH1	0	CD163	0	NOTCH4	7.57E-240
RPL39	2.67E-172	TPP1	0	ANXA1	1.126E-27	ST13	0	NCF2	0	S100A13	0	S100A16	9.38E-240
CKLF	3.19E-172	GRB2	0	CMIP	1.488E-27	UBE2D3	0	ACOT7	0	GS1-600G8	0	LTBP1	3.73E-237
OAZ1	6.48E-172	CD53	0	RNF144B	4.668E-27	MORF4L1	0	LRPAP1	0	GAA	0	BMPR2	4.52E-237
CLEC7A	8.9E-172	TIMP2	0	RGS1	7.027E-27	NCOR1	0	CTSL	2.42E-307	C6orf48	0	SELM	8.13E-236
HLA-DRB1	3.53E-170	CAPZB	0	CTSS	1.119E-26	GABARAP	0	IFI44L	4.03E-307	CRIP1	0	COL1A2	8.86E-235
ARPC5	6.99E-170	CCL7	0	RNASE6	2.776E-26	COX17	0	SOAT1	1.58E-304	TREM2	0	COL6A1	4.08E-232

Cluster 1 (airway and alveolar epithelial cells)		Cluster 2 (ciliated cells)		Cluster 3 (type II alveolar cells-1)		Cluster 4 (type II alveolar cells-2)		Cluster 5 (type II alveolar cells-3)		Cluster 6 (immune cells)		Cluster 7 (plasma cells)		Cluster 8 (monocytes-1)		Cluster 9 (monocytes-2)		Cluster 10 (alveolar macrophages-1)		Cluster 11 (alveolar macrophages-2)		Cluster 12 (alveolar macrophages-3)		Cluster 13 (alveolar macrophages-4)		Cluster 14 (mesenchymal cells)	
Gene symbol	Adjusted p values	Gene symbol	Adjusted p values	Gene symbol	Adjusted p values	Gene symbol	Adjusted p values	Gene symbol	Adjusted p values	Gene symbol	Adjusted p values	Gene symbol	Adjusted p values	Gene symbol	Adjusted p values	Gene symbol	Adjusted p values	Gene symbol	Adjusted p values	Gene symbol	Adjusted p values	Gene symbol	Adjusted p values	Gene symbol	Adjusted p values	Gene symbol	Adjusted p values
SPTBN1	5.46E-252	SRI	0	RPS28	0	ZFP36L1	0	LINC00657	0	GPSM3	8.35E-216	UFM1	1.334E-18	PLD4	1.18E-168	RGS10	0	CLEC12A	3.969E-26	C20orf24	0	RBP4	4.08E-304	LPL	0	FAM167B	6.62E-231
PPL	2.8E-250	WDR54	0	MMAB	0	C14orf1	0	TRAM1	0	CD99	6.05E-214	RPL22L1	6.862E-18	FXYD5	6.53E-168	RNASE6	0	CTSB	4.331E-26	UGP2	0	CRIP1	3.87E-302	FCGR2A	0	NNMT	3.5E-228
RNASE1	3.97E-250	FAM174A	0	TFCP2L1	0	RHOBTB2	0	NBEAL1	0	CPA3	4.42E-213	GADD45A	2.815E-17	RPL30	1.54E-167	CLEC5A	0	FOSL2	7.898E-26	RGCC	0	TFRC	1.11E-301	LINC01272	0	IFI27	5.14E-227
SYT8	2.95E-249	OSBPL6	0	PRDX2	0	XIST	0	SAR1A	0	FAM118A	3.04E-212	LMO4	1.383E-15	CSF2RA	1.88E-166	GNAI2	0	PDK4	7.959E-26	TXN	0	SEPT11	1.78E-299	S100A10	0	FSTL1	1.01E-226
ATP1B1	3.84E-248	NPHP1	0	TM7SF2	0	CYP4B1	0	CHP1	0	CD8A	8.92E-210	SVIP	6.768E-15	SLA	1.99E-166	CAP1	0	LST1	8.792E-26	ATP6V1E1	0	LPXN	3.92E-299	TKT	0	PPP1R14A	4.65E-226
RBPMS	9.34E-248	DNAH11	0	TSC22D1	0	PTP4A1	0	AMD1	0	PARP1	1.58E-209	BIRC3	4.026E-05	LRRC25	3.38E-166	HLA-DMB	0	AIF1	3.9E-25	MAP1LC3I	0	RNF149	9.39E-298	EIF3K	0	ITM2A	4.61E-225
EXPH5	9.67E-248	DNAL1	0	PDE4D	0	PPA1	0	SEC62	0	POU2F2	1.53E-208			HLA-DPB1	5.4E-166	MAF	0	SAMSN1	6.078E-25	ATP6V1F	0	IRS2	1.91E-297	B2M	0	DPYSL3	1.84E-224
PROM2	4.15E-247	CFAP54	0	TMEM87A	0	CRTAC1	0	FRMD4B	0	RPS28	1.3E-206			ADM	4.99E-165	FAM49B	0	CORO1C	6.805E-25	UBXN1	0	MYADM	2.03E-297	AKIRIN2	0	CNN3	6.24E-224
RAB27B	2.73E-246	TMEM67	0	BMP1	0	RAB27A	0	DDX17	0	HIST1H1E	4.05E-201			RPS13	1.49E-164	ARHGAP1	0	MS4A7	1.252E-24	ABHD5	0	GSTO1	3.86E-297	IFI6	0	RARRES2	6.17E-223
SLC34A2	5.77E-246	MAATS1	0	ITGB6	0	CAV1	0	RPN2	0	GRAP2	8.94E-201			KCTD12	5.62E-164	WARS	0	CDC42SE1	1.875E-24	NCF2	0	NUPR1	1.17E-294	SEC11A	0	FAM43A	1.3E-220
MECOM	3.66E-245	AK9	0	MSMO1	0	ALPL	0	ITSN2	0	CD52	5.71E-200			RPL7	2.42E-163	DAB2	0	LGALS1	4.998E-24	RPL38	0	CBX3	3.58E-294	TNNI2	0	IGFBP5	3.03E-220
GPC1	5.9E-245	MLF1	0	WBP5	0	VEPH1	0	ATRX	0	CD82	8.88E-200			SIGLEC10	5.43E-163	GPR183	0	ITGB2	5.336E-24	AIMP1	0	POMP	2.57E-293	BAX	0	MFAP4	1.13E-216
SPINT1	1.25E-244	FAM166B	0	TMEM41B	0	C3	0	WNK1	0	FYN	1.35E-198			RHOG	5.47E-163	C3AR1	0	CYBB	4.384E-23	RP5-839B4	0	CD53	5.33E-291	PSAP	0	PLAT	2.13E-212
FAM129B	6.28E-244	CALM1	0	GEM	0	PEBP1	0	UQCRQ	0	FNBP1	4.63E-198			ABI3	5.48E-163	GNB4	0	KCTD12	2.788E-22	EIF5B	0	MIR3945H	5.85E-289	OLR1	0	LHFP	1.95E-211
NAV2	2.9E-243	SOX2	0	C12orf57	0	NEDD4L	0	IPO7	0	TMSB4X	2.33E-197			VAMP5	1.39E-162	S100A11	0	MXD1	3.005E-21	EVI2A	2.7E-307	ATP6V1F	1.29E-284	TXNIP	0	FOXF1	7.67E-211
EFEMP1	5.4E-243	RSPH4A	0	MRPS36	0	FOXA2	0	TMED2	0	ITGA4	5.54E-196			ADAM28	1.16E-160	FKBP1A	0	PLPP3	1.162E-20	SNX3	1.99E-306	AGRP	4.62E-284	CAMP	0	CXCL12	1.67E-210
MYO1C	5.39E-242	DNAH9	0	SNHG7	0	IRX2	0	USP8	0	GZMB	6.62E-196			CD1D	3.41E-160	LGALS9	0	EMP3	1.648E-20	FAM89A	4.5E-306	TIMP2	1.18E-279	PTPN6	0	PTGDS	2.59E-210
CYR61	1.41E-241	CASC1	0	CHP1	0	PDE4D	0	SEC63	0	HLA-C	2.29E-195			HRH2	3.93E-160	FBP1	0	IGSF6	3.537E-20	CD68	1.32E-305	PLXDC2	3.49E-279	RMDN3	0	GPR146	4.56E-209
COL12A1	1.55E-241	RIIAD1	0	NPNT	0	POLR2L	0	MBNL1	0	APOBEC3	3.22E-195			SERPINB1	2.21E-159	PMP22	0	THBS1	3.73E-20	MTIF3	7.08E-303	PTPRC	4.36E-279	SNX10	0	CDH11	1.28E-208
ASS1	2.84E-241	SSBP4	0	RPS4X	0	ALOX15B	0	SARS	0	BLK	1.76E-192			HNRNPA1	3.27E-158	GRN	0	FCGR2A	8.537E-20	DPH3	1.79E-302	SLC7A7	3.62E-275	PLAUR	0	PDE2A	7.41E-207
NCKAP5	3.86E-241	NME5	0	COBLL1	0	CACNA2D	0	TMEM30A	0	AL928768.	2.37E-192			MEF2C	1.44E-157	VAMP5	0	XIST	6.174E-19	RP11-598F	2.85E-302	SLCO2B1	2.97E-273	FPR2	0	PPFIBP1	1.16E-206
OCIAD2	4.24E-241	CCDC181	0	CRNDE	0	TSPAN3	0	NUCKS1	0	GNG2	2.4E-192			WAS	4.9E-157	PTAFR	0	NHSL2	8.612E-19	GPNMB	2.02E-301	SECTM1	4.89E-271	UBE2L6	0	ENPP2	2.35E-206
HSPG2	5.76E-241	FANK1	0	RPS6KA2	0	CYP51A1	0	SLC25A3	0	LSP1	3.04E-192			IGSF6	4.08E-156	ANXA5	0	IRS2	1.029E-18	SPN	6.63E-301	HNMT	6.55E-271	GPX4	0	CAV2	1.31E-204
NXN	1.01E-239	FABP6	0	RPL6	0	GSTP1	0	KTN1	0	STAG3	5.5E-192			HLA-C	5.16E-156	ACP2	0	HLA-A	1.104E-18	SVIL	1.06E-300	XAF1	1.35E-267	LY86	0	EDN1	1.97E-204
SPINT2	2.12E-239	NEK10	0	EEF2	0	KCNJ15	0	ARL5A	0	UCP2	1.27E-191			HLA-DPA1	1.7E-155	MFSD12	0	TNFAIP3	2.796E-18	ARF6	2.75E-299	GRINA	1.47E-267	MT-CO1	0	GATA2	2.2E-204
MAGI3	1.37E-238	PLPP2	0	RPSA	0	LDHB	0	YWHAE	0	ZAP70	3E-191			RPS16	8.3E-154	PLXDC2	0	HLA-DQA	2.041E-17	APOE	1.58E-298	FGR	2.09E-267	BLOC1S1	0	SOCS3	1.93E-201
NGFRAP1	1.49E-238	CFAP44	0	POLR3H	0	AHCYL2	0	ZFP36	0	TUBA4A	8.62E-191			PFDN5	8.51E-154	BCAP31	0	SDCBP	3.668E-17	VPS29	5.36E-298	GABARAP	1.49E-265	STMN1	0	LIMS2	9.31E-201
ALDH3A1	3.86E-238	STMND1	0	AGPAT2	0	PRDX5	0	GFPT1	0	HMGN1	1.3E-190			F13A1	1.13E-153	PTPRC	0	B3GNT5	1.2E-16	LINC01272	1.44E-297	OAZ1	2.25E-263	SNX2	0	NBL1	2.61E-199
MUC1	1.14E-237	C9orf9	0	RARRES3	0	PTPN13	0	FDFT1	0	GIMAP7	9.51E-190			RPL35A	1.44E-153	PGK1	0	RNF149	1.617E-16	SH3GLB1	9.08E-297	MPHOSPH	2.5E-263	HBEGF	0	ADCY4	1.67E-197
RHOBTB3	2.16E-237	RSPH9	0	BTG3	0	SPTBN1	0	UTRN	0	RHOF	2.59E-189			RPL6	7.32E-153	MPP1	0	EMP1	1.569E-15	SFT2D1	2.78E-296	LEPROTL1	4.5E-262	CD63	0	FBN1	3.22E-197
IGFBP7	1.18E-236	CFAP45	0	GPR160	0	LMO3	0	EPHX1	0	LBH	1.05E-188			ACTG1	1.31E-152	RHOA	0	RHOB	5.074E-15	SEPT11	2.54E-295	VMO1	4.77E-262	BHLHE41	0	ECE1	1.08E-196
SLC1A1	5.85E-236	C6orf118	0	ATPIF1	0	MECOM	0	DMBT1	0	FCMR	1.54E-188			RPS19	1.31E-152	B2M	0	PLIN2	2.42E-14	LEPROTL1	7.94E-295	EMP3	1.03E-260	ADAMTSL	0	INMT	2.62E-196
COL8A1	6.51E-236	RP4-539M	0	RPS5	0	ETV1	0	AKAP1	0	SEPT7	5.32E-188			RCSD1	6.39E-152	RBPJ	0	CD163	2.368E-13	RSL1D1	4.58E-294	CXCR4	2.68E-260	ZFAS1	0	GIMAP7	9.65E-196
TPBG	1.26E-235	SPEF1	0	RPS9	0	TACSTD2	0	TMBIM6	0	FCRLA	1.95E-187			ZEB2	8.84E-152	NAGK	0	KLF4	9.449E-13	RTN3	1.01E-293	CXCL16	2.79E-260	MMP19	0	SLCO4A1	3.73E-195
MMP1	1.44E-234	VWA3B	0	NEBL	0	DDAH1	0	RAB27A	0	RP1-34B20	2.47E-186			LY86	9.74E-152	HLA-DRB1	0	AREG	4.366E-12	GLDN	1.19E-293	LCP1	1.1E-257	VMO1	0	ITGB1	3.73E-193
TJP1	2.72E-234	LRTOMT	0	FXYD3	0	PARD6B	0	CPB2	0	SMCHD1	2.5E-185			RPS8	1.41E-151	CPVL	0	GPNMB	1.603E-11	FBP1	3.32E-293	LGALS9	4.93E-257	SLC15A3	0	NDRG1	8.65E-193
ADGRG1	7.93E-234	DPCD	0	CYBRD1	0	PIGR	0	ABCA3	0	MS4A2	4.11E-184			LILRA1	1.87E-151	H2AFY	0	ZFP36L1	4.985E-11	GRN	5.05E-293	RPL17	4.26E-256	BLOC1S2	0	F8	1.72E-192
CH17-360D	1.75E-233	PROM1	0	SNX25	0	TMEM125	0	C16orf89	0	NCF1	5.61E-183			CSTA	1.07E-149	TMSB4X	0	THBD	8.789E-11	MCL1	9.96E-293	GLIPR2	5.69E-256	SMCO4	0	PLPP1	2.29E-191
C1S	1.18E-232	CFAP52	0	MPST	0	OCLN	0	HACD3	0	OXNAD1	1.97E-182			SAMSN1	2.32E-148	SPI1	0	LIPA	5.23E-10	ZC3H13	1.76E-292	ABCG1	4.62E-252	ARL4A	0	UACA	2.32E-191
BACE2	4.95E-232	UBXN10	0	CRB3	0	MALAT1	0	CYP51A1	0	CD22	1.2E-181			PTPRE	8.19E-148	ARHGDIB	0	S100A8	2.156E-09	RSL24D1	5.6E-290	PLSCR1	3.04E-249	NPM1	0	SOX17	2.52E-190
ADGRF1	5.82E-232	TSGA10	0	RPS27	0	CNN3	0	CPM	0	P2RX5	1.94E-181			FKBP5	1.87E-147	GSTO1	0	RASGEF1E	7.356E-09	BHLHE41	1.26E-289	LITAF	2.05E-248	LAMTOR1	0	MARCKSL	2.57E-189
PLEKHA1	8.13E-231	VSTM2L	0	CLDN7	0	TNNC1	0	SLCO4C1	0	INPP5D	1.17E-179			HLA-A	5.62E-146	EMILIN2	0	EREG	5.926E-08	LPL	8.25E-289	ACER3	1.63E-247	RNF149	0	GUK1	2.71E-187
ADGRF5	1.24E-228	TMEM232	0	COX7A2	0	CRNDE	0	RCN2	0	P2RY10	4.71E-179			CD300E	6.27E-146	ZYX	0	IL1RN	1.801E-07	CDC42	8.49E-289	MYL6	2.51E-247	GPSM3	0	IGFBP3	3.5E-187
OSMR	1.88E-227	RGS22	0	EPDR1	0	CCND1	0	NFIA	0	LRMP	1.28E-176			RPL4	4.16E-144	MGAT1	0	OLR1	9.506E-07	ASAH1	2.39E-288	SPATS2L	6.54E-247	NDUFS5	0	PDLIM1	6.47E-185
IFI27	2.45E-227	MOK	0	DSTN	0	SMIM22	0	ATP2C1	0	BCL11B	1.38E-176			NACA	2.22E-142	PGAM1	0	C15orf48	1.409E-06	SLC7A7	7.25E-288	LGALS3BI	3.52E-246	CSTB	0	CSRP1	2.23E-184
MAP2	4.27E-227	SPAG8	0	SPAG9	0	IRX3	0	TMEM41B	0	TNFRSF13	3.44E-176			RPL27	3.04E-142	FERMT3	0			SNX2	4.3E-285	FCGR3A	7.77E-246	AVPI1	0	COL3A1	3.29E-184
ARHGEF2	1.67E-226	EFCAB10	0	MTUS1	0	ANK3	0	C6orf62	0	PTMA	1.19E-175			FCGR2B	3.45E-142	HAVCR2	0			GLRX	1.64E-283	CTNNB1	1.99E-245	DDAH2	0	SEMA6A	2.94E-182
INADL	8.44E-226	MUC4	0	C11orf96	0	MET	0	FAM46A	0	EMB	1.24E-175			RP11-1143	5.64E-142	CD86	0			CXCL16	4.26E-283	CXCL5	6.47E-243	EVI2A	0	DNAJB4	5.23E-181
SFTPB	4.03E-224	TUSC3	0	MAP1LC3	0	AQP1	0	DESI2	0	RPL5	1.3E-175			EVI2B	1.22E-141	GLUL	0			ZBTB8OS	1.11E-282	TBCA	9.47E-243	SLC31A2	0	ARHGEF1	1.28E-180
PRSS8	1.06E-221	FGF14	0	ASPH	0	EFEMP1	0	ATP2B4	0	SNRPD2	5.25E-175			RUNX3	9.6E-140	TLN1	0			CAPZA1	3.44E-281	LAMP2	1.86E-242	GCA	0	TMEM47	1.52E-180
ALDH3A2	1.94E-221	TCTEX1D1	0	CLDN4	0	CYR61	0	NR4A1	2.96E-307	SH2D1A	4.86E-174			NAAA	1.95E-139	NCEH1	0			SPG21	6.06E-279	PABPC4	1.3E-241	MNDA	0	HIF3A	8.12E-179
VEGFA	4.49E-221	CCDC74A	0	NEAT1	0	TMC5	0	FOXP1	3.4E-307	TNFRSF18	6.93E-172			APOBEC3	1.53E-138	MTHFD2	0			MIS18BP1	3.81E-275	AMIGO2	3.6E-241	UBXN1	0	CTNNAL1	8.02E-178
FLNB	4.66E-221	CFAP46	0	C7orf55	0	IFITM3	0	FILIP1	2.02E-306	AC006129.	1.27E-171			PLAC8	7E-138	FCGR1A	0			C1QC	4.05E-275	SARAF	3E-240	FHL1	0	MKL2	2.19E-177
TMEM45A	6E-221	ADGB	0	TOB1	0	HHIP	0	LMO3	8.12E-306	GABPB1	2.31E-170			RP5-1171I	2.68E-137	LAIR1	0			DNASE2	7.38E-275	CD37	9.96E-238	PHLDA3	0	FAM171A1	4.09E-177
HES1	2.97E-220	MAPK15	0	RPL41	0	ADIRF	0	SPRY4	2.6E-305	HIST1H2B	1.13E-169			HLA-DRA	1.58E-136	RAB31	0			RND3	3.55E-274	RAP1B	5.29E-237	CARD16	0	RGS5	1.69E-176
MT1E	1.68E-219	C10orf107	0	NFIX	0	AGER	0	TGFBR2	2.8E-305	PAX5	1.31E-167			STK17B	2.56E-136	TNFRSF1B	0			ANKRD12	1.21E-273	TRIM22	3.28E-236	ATP5G3	0	YBX3	2.31E-176
KITLG	5.01E-219	C22orf15	0	SMAGP	0	S100A6	0	RASEF	4.08E-305	IL16	1.65E-167			CLIC1	1.28E-135	TNS3	0			ARRDC4	4.13E-272	PLBD1	1.32E-235	S100A6	0	MYLK	2.71E-176
ID1	4.61E-218	CD164L2	0	POLR2C	0	EPDR1	0	ACSL1	3.44E-303	GTF3A	4.95E-167			P2RY13	4.06E-134	SLC1A3	0			ACER3	1.62E-271	PHLDA3	9.65E-234	B3GNT5	0	KIAA0355	5.64E-176
CYB5A	1.1E-217	MAP1B	0	BNIP3	0	SGMS2	0	GNAS	2.19E-301	HCST	1.03E-166			GIMAP4	4.86E-134	GLIPR2	0			PTPN12	1.74E-271	CARD16	2.47E-231	RPL28	0	C1QTNF1	7.21E-176
MYO5C	1.11E-217	FAM179A	0	SQLE	0	GJA1	0	MET	2.78E-301	MZT2A	2.13E-166			ACTR2	6.31E-134	DMXL2	0			ANKRD28	1.61E-270	CFD	6.34E-230	OSCAR	0	DACH1	1.84E-174
PDZK1IP1	2.35E-217	PLEKHS1	0	STARD10	0	TRIB1	0	EBP	1.95E-300	RP11-693J	2.33E-166			LILRB4	5.2E-133	SDCBP	0			CHCHD10	2.14E-270	MME	1.2E-227	KCNAB2	0	F2R	1.5E-173
LAMA3	7.05E-217	WDR63	0	COX7C	0	CDS1	0	PABPC1	8.05E-300	CD19	3.55E-166			CST7	9.69E-133	IQGAP2	0			LAMP2	2.31E-270	FKBP5	6.42E-227	FGR	0	KIAA1462	1.89E-173
PMEPA1	8.21E-217	LRRC10B	0	PARM1	0	GPR160	0	RUFY3	1.3E-298	HPGDS	1.31E-165			RPS11	1.46E-131	RAP2B	0			YWHAZ	3.74E-269	SNTB1	1.18E-226	BST2	0	KDR	5.29E-173
MISP	2.12E-216	C6	0	LDLR	0	GSTA4	0	FLRT3	1.12E-297	LINC00926	3.8E-165			CLEC5A	2.38E-131	LINC01272	0			CEBPB	1.77E-267	ABHD5	6.37E-222	TXN	0	FKBP1A	3.44E-172
PKP3	2.23E-215	KIAA1211	0	BFAR	0	JUN	0	GOLPH3	1.36E-297	TRAF5	4.81E-164			ICAM3	4.55E-131	CMTM6	0			VDAC1	2.57E-266	CLEC12A	3.03E-218	RNH1	0	THBD	6.37E-172
VWA1	7.97E-215	NWD1	0	HYI	0	SOCS2	2.51E-308	LIMCH1	1.56E-297	TMEM156	7.11E-164			PLXNC1	8.69E-131	M6PR	0			GPCPD1	3.38E-266	OPN3	7.04E-217	MFGE8	0	KLF9	7.6E-172
PLXNB1	1.05E-214	C11orf70	0	PPA1	0	DUOXA1	3.33E-304	PURA	1.05E-296	PSMB8-AS	1.65E-162			STAB1	2.3E-130	WIPF1	0			CHURC1	6.73E-266	YWHAG	1.06E-216	INHBA	0	CSRP2	3E-171
AGR3	2.05E-214	SRGAP3-A	0	DPM3	0	PRKAR1A	1.43E-303	PON2	3.91E-296	ANKRD44	1.8E-162			HLA-E	2.68E-130	FAM20C	0			NSRP1	3.35E-264	SLFN11	2.86E-216	FAU	0	PRKCDBP	8.79E-171
KIAA1522	8.71E-214	RABL2A	0	RGS16	0	ID1	3.78E-303	RB1CC1	5.24E-295	PSMB8	3.09E-162			DBNL	2.71E-130	EFHD2	0			GCA	3.6E-264	CLEC4E	5.46E-214	NCF4	0	CASKIN2	1.04E-170
PTGS2	4.91E-213	APOBEC4	0	GOLT1A	0	PIGA	2.84E-302	RFC1	1.64E-294	HIST1H2B	1.86E-160			IFI16	4.86E-130	FLNA	0			RAC1	1.05E-263	RTN4	9.88E-214	ARHGAP1	0	FBLN2	4.84E-170
ZDHHC9	9.27E-212	C7orf57	0	C3	0	RPLP0	5.14E-302	PPP2CB	3.24E-294	RGS1	2.62E-160			AP2S1	9.18E-130	LST1	0			DECR1	1.52E-263	NCEH1	5.02E-212	CD53	0	ESAM	1.49E-169
TP63	3.05E-211	DNAI2	0	FILIP1	0	PLA2G4F	9.21E-300	UBE3A	1.52E-292	TNFRSF13	1.25E-159			PSAP	1.13E-129	SLAMF8	0			PCOLCE2	4.35E-263	SNX2	1.76E-211	AXL	0	ADH1B	2.3E-169
PDPN	3.51E-211	DNAH10	0	DMKN	0	COBLL1	2.51E-299	SLC6A14	4.61E-292	ITK	3.67E-158			CNPY3	3.71E-128	LGALS3	0			ANXA5	6.63E-263	MMP19	2.75E-211	LAIR1	0	CD99	2.84E-167
VSIG2	4.75E-211	DNAH6	0	ZNF106	0	UQCRQ	1.01E-298	EPDR1	1.04E-291	EIF3F	1.7E-157			RGS19	7.88E-128	CD4	0			CFDP1	8.74E-263	FPR2	7.87E-211	RP3-460G2	0	NID1	6.77E-167
CXADR	2.32E-210	TEKT2	0	PLA2G4F	0	SLCO4C1	1.3E-298	PEBP1	2.12E-291	C12orf75	2.19E-157			CLEC4E	1.94E-127	NABP1	0			PAG1	5.58E-261	MS4A4A	3.35E-208	TFPT	0	NOSTRIN	1.62E-165
RAB25	5.4E-209	CFAP65	0	CD59	0	NCOA7	1.49E-297	MEGF9	4.72E-291	PSME1	1.93E-156			PGLS	4.3E-127	PLEKHO1	0			ROCK1	2.31E-259	MYL12A	8.58E-207	GPNMB	0	GALNT15	9.04E-164
GOLGA8B	6.2E-209	DRC1	0	DESI2	0	NTN4	3.9E-297	MPZL2	6.93E-291	EVL	8.53E-156			GNAI2	2E-126	YWHAZ	0			PABPC4	3.72E-259	VPS29	2.7E-205	ARRB2	0	GPIHBP1	5.88E-163
IGFBP2	2.63E-208	ALOX15	0	RPL8	0	TMEM87A	1.5E-296	TRA2A	9.31E-291	TNFRSF4	9.84E-155			EEF1D	2.15E-126	SIRPA	0			MGST3	3.83E-259	ATP5E	5.1E-205	FPR1	0	GIMAP8	1.54E-162

Cluster 1 (airway and alveolar epithelial cells)		Cluster 2 (ciliated cells)		Cluster 3 (type II alveolar cells-1)		Cluster 4 (type II alveolar cells-2)		Cluster 5 (type II alveolar cells-3)		Cluster 6 (immune cells)		Cluster 7 (plasma cells)		Cluster 8 (monocytes-1)		Cluster 9 (monocytes-2)		Cluster 10 (alveolar macrophages-1)		Cluster 11 (alveolar macrophages-2)		Cluster 12 (alveolar macrophages-3)		Cluster 13 (alveolar macrophages-4)		Cluster 14 (mesenchymal cells)	
**Gene symbol**	**Adjusted p values**	**Gene symbol**	**Adjusted p values**	**Gene symbol**	**Adjusted p values**	**Gene symbol**	**Adjusted p values**	**Gene symbol**	**Adjusted p values**	**Gene symbol**	**Adjusted p values**	**Gene symbol**	**Adjusted p values**	**Gene symbol**	**Adjusted p values**	**Gene symbol**	**Adjusted p values**	**Gene symbol**	**Adjusted p values**	**Gene symbol**	**Adjusted p values**	**Gene symbol**	**Adjusted p values**	**Gene symbol**	**Adjusted p values**	**Gene symbol**	**Adjusted p values**
DST	1.3E-207	TTC26	0	MCCC1	0	ATP8A1	2.21E-296	NFKBIA	9.11E-290	SMIM14	2.94E-154			RP6-159A1	6.12E-126	YBX1	0			PLSCR1	1.36E-257	OAS1	3.02E-204	ATP6V0D1	0	TSPAN18	3.61E-162
MAGED1	2.16E-207	DOC2A	0	KCNJ8	0	SPINT2	2.38E-296	TMPRSS2	2.38E-289	CORO1B	1.05E-153			RAC2	1.32E-124	FAM198B	0			RBPJ	2.01E-256	SPN	1.71E-202	PRSS21	0	CLEC3B	4.04E-161
PCYOX1	2.19E-207	RP11-275I1	0	NAA38	0	SNX30	3.09E-296	KIDINS220	2.61E-289	KIT	3.23E-151			BTF3	2.09E-124	OAZ1	0			PSMG2	5.14E-256	GPATCH11	3.66E-202	NMB	0	FCN3	9.24E-161
ENAH	4.91E-207	STOML3	0	MET	0	PLS3	8.74E-296	NCOA7	7.84E-289	BIN1	2.8E-150			ITGA4	2.46E-123	MATK	0			YWHAG	7.83E-256	ATP6AP1	1.71E-199	DECR1	0	FZD4	7.37E-159
S100A14	1.09E-206	NEK11	0	RPL23	0	RPS6KA2	4.49E-295	ZFP36L1	8.15E-289	RPS9	5.92E-150			GCA	7.82E-123	C1orf54	0			GPATCH11	3.21E-255	RNF13	2.19E-198	PILRA	0	ACVRL1	1.97E-158
AMOTL2	5.21E-206	MAP1A	0	KIAA1324I	0	ST3GAL5	4.04E-293	EPCAM	8.45E-289	SP140	8.33E-150			SLC2A3	8.64E-122	CD48	0			GRB2	1.43E-254	PYCARD	8.82E-198	LTA4H	0	SMAD6	2.52E-158
MYRF	1.6E-205	C11orf16	0	LANCL1-A	0	SYT15	4.65E-290	EBAG9	1.65E-288	HLA-B	4.22E-149			SUB1	2.06E-121	FOLR2	0			RAB13	2.17E-253	UPP1	1.41E-197	LAMTOR2	0	NR2F1	5.47E-158
DKK3	7.15E-203	TEX9	0	C5orf38	0	SFTA1P	4.3E-288	ATP8A1	2.21E-288	RUNX3	6.52E-148			PSMB9	2.82E-121	ACP5	0			MDM2	2.59E-253	AZI2	3.71E-197	TNFAIP2	0	MMP2	7.75E-158
SYTL1	8.02E-203	DLEC1	0	RPL7	0	EFNA1	2.49E-287	PLA2G4F	1.37E-287	RPL12	8.32E-148			GDI2	3.59E-121	FUOM	0			TRIM22	9.57E-253	SAMHD1	3.85E-197	REEP5	0	MYH11	1.57E-157
GGTLC1	8.55E-203	AKAP14	0	CCDC57	0	C2	1.89E-285	TANC2	1.66E-285	KLRD1	2.49E-147			LILRA2	1.33E-120	HEXB	0			RNF13	1.26E-252	KCTD12	5E-197	CFL1	0	HSPA12B	3E-157
CADM1	1.07E-202	CAPS2	0	RPL5	0	EPS8	2.26E-284	CXCL2	5.98E-284	RPS12	2.73E-147			DDIT4	1.55E-120	BMP2K	0			TSC22D3	8.55E-252	FMNL2	8.64E-197	PGD	0	TFPI	1.42E-156
SMIM22	1.14E-202	PPP1R42	0	HRSP12	0	NPNT	1.37E-283	ETV5	2.76E-282	CYBA	3.96E-147			ADAM8	3.32E-120	ADGRE2	0			SSB	5.27E-251	RAC1	3.92E-196	VDAC2	0	KCTD12	1.71E-155
SHROOM3	2.42E-202	CTXN1	0	MID1IP1	0	RASEF	3.23E-282	CLDN18	1.59E-281	LINC00152	3.68E-145			SLC8A1	1.27E-119	MRC1	0			SLC31A2	1.11E-250	FPR1	2.01E-193	PPIC	0	RBMS3	9.17E-155
FOXA2	2.82E-202	OSCP1	0	AQP4	0	CLDN3	1.33E-281	DCBLD2	3.27E-280	ANXA2R	4.21E-145			PARVG	1.45E-118	ASAP1	0			SLCO2B1	1.9E-250	SELPLG	1.59E-192	LY96	0	GFOD1	6.76E-154
LSR	2.87E-202	VWA3A	0	SCNN1A	0	NFKBIZ	1.56E-280	ETS2	3.84E-280	ZNF90	6.79E-145			TMEM176I	2.43E-118	ADAM9	0			BTF3L4	3.21E-250	MNDA	3.53E-192	BCL2A1	0	ADAMTS4	1.47E-153
PTPN14	1.67E-201	ANKUB1	0	SUSD2	0	MAGI1	1.24E-278	MAST4	4.82E-280	PFN1	4.47E-144			HMHA1	3.12E-118	MMP14	0			SPATS2L	9.06E-250	PDCL	1.17E-191	TGM2	0	FOSL2	3.4E-153
KIAA1217	3.53E-201	C4orf47	0	RPS20	0	PON3	7.51E-276	FMO5	1.42E-279	C9orf142	5.2E-144			PPP1R18	3.18E-118	CMTM3	0			LRPAP1	5.07E-249	UBE2L6	2.31E-189	UBA52	0	LUM	8.87E-153
MPZL2	5.19E-201	CCDC114	0	RAB25	0	KLF6	2.26E-275	MACF1	2.01E-279	SPIB	5.87E-144			RPS17	1.22E-117	C4orf48	0			SAMHD1	6.18E-246	YWHAH	2.45E-189	ATP6AP1	0	C1S	2.04E-152
FNBP1L	2.44E-200	ARMC4	0	SNRPF	0	DMKN	3.83E-274	ARMC9	2.65E-279	TESPA1	9.34E-144			CAPZB	2.76E-117	CLEC7A	0			STOM	2.61E-245	FABP3	1.55E-188	LGALS9	0	RPGR	2.84E-152
GOLGA8A	1.42E-199	SCGB2A1	0	ALDH6A1	0	FADS1	7.8E-274	C11orf96	8.81E-279	CCND3	8.81E-143			ATP5G2	4.42E-117	ADAP2	0			SPI1	4.45E-245	DST	3.5E-188	H2AFZ	0	ETS1	1.62E-151
C9orf3	1.02E-198	LRRC6	0	ANK3	0	NECAB1	8.3E-274	BAZ1B	3.15E-278	PRF1	2.35E-142			TBXAS1	1.58E-116	HLA-DPA1	0			SMCO4	9.69E-245	ATP6V0B	5.33E-188	EIF4EBP1	0	HOXA5	3.86E-151
CFH	9.73E-198	RP11-101E	0	NDUFC2	0	TGFBR2	1.04E-272	MGLL	2.74E-277	MEF2C	3.76E-142			RPL10A	2.41E-116	HLA-E	0			LRP1	1.42E-244	LAIR1	1.4E-186	CTSS	0	HSPB1	9.64E-151
DAB2IP	1.77E-197	RAB36	0	ARMC9	0	SLC6A14	6.31E-270	SLC25A36	3.71E-277	ARGLU1	1.27E-141			MARCKSL	3.52E-116	BCL2A1	0			CD81	2.2E-244	FRMD4A	2.23E-186	NDUFB5	0	NPR3	3E-148
MBOAT2	3.14E-197	ABCA13	0	PRSS8	0	CDH1	7.35E-265	TMEM125	1.7E-276	APRT	2.3E-141			SLC25A6	7.7E-116	HLA-DRA	0			AMIGO2	2.55E-244	EVI2A	2.57E-186	CKLF	0	JUNB	4.75E-148
PTRF	5.46E-196	SLFN13	0	RPS14	0	BMP1	1.48E-264	PON3	4.52E-276	EIF3D	7.56E-141			LPAR6	4.32E-115	GALM	0			ATP6AP1	2.61E-244	HN1	3.85E-186	HLA-A	0	PKIG	8.36E-148
TINAGL1	2.83E-195	IQCK	0	RPL10	0	PDLIM3	7.52E-264	CLDN7	5.04E-276	RP5-1171I1	9.16E-140			CAP1	1.23E-114	GPR137B	0			POR	3.24E-244	PECAM1	4.23E-186	ANXA2	0	VCAM1	1.46E-147
VCL	1.22E-194	TTC25	0	RPL13	0	PON2	7.52E-263	STEAP4	2.05E-274	UBA52	3.58E-139			CD37	4.12E-114	SERF2	0			VAT1	8.54E-244	IGSF6	8.19E-186	HCST	0	BCL6B	1.55E-145
NFIX	2.33E-194	MDH1B	0	SCD	0	SPRY4	1.51E-260	DDAH1	7.24E-274	SMAP2	4.13E-139			SLC16A3	4.61E-113	KIAA0930	0			CASP4	1.85E-243	RHOB	2.12E-184	THEMIS2	0	TSHZ2	2.85E-145
TNFRSF12	2.94E-194	DNAAF3	0	YAP1	0	IL6ST	4.44E-260	PPP2R5A	8.86E-274	STK17B	5.04E-139			PKM	5.7E-113	LILRB1	0			NHSL2	1.86E-243	DOCK11	5.7E-182	CPVL	0	MT1A	4.64E-144
RBFOX2	3.75E-194	DNAH2	0	CDKL2	0	SLC38A1	7.55E-260	SLC38A1	1.73E-273	PRDM1	5.16E-139			NLRP3	5.96E-113	CAPZA1	0			KLF4	4.52E-242	TFEC	7.51E-181	ANXA11	0	SH2D3C	6.06E-144
FSTL3	2.13E-192	TCTEX1D	0	ARFGEF3	0	PIP5K1B	2.85E-258	FDPS	1.93E-273	HIST1H1C	7.02E-139			EFHD2	1.05E-112	FXYD5	0			PGD	4.92E-242	CITED2	9.33E-181	RPS4Y1	0	TBX3	6.79E-144
SH2D3A	9.56E-192	PTPRT	0	RPL10A	0	ADGRD1	6.78E-258	ITPR3	4.45E-273	AKNA	4.12E-137			RPL18	8.66E-112	SMIM3	0			MDH1	2.43E-241	B3GNT5	1.35E-180	NACA	0	NDRG4	7.77E-143
PON2	2.54E-190	AK7	0	AGR3	0	DHCR7	1.72E-257	EPS8	4.63E-273	CCDC167	6.29E-137			GNB2L1	2.89E-110	NCF2	0			APOBR	2.72E-241	OASL	4.04E-178	H2AFY	0	IL6	1.2E-142
FAM83H	3.24E-189	EFHB	0	AC007405.	0	PFKFB2	1.09E-256	CRNDE	1.92E-272	AIM2	9.82E-137			CD4	4.85E-110	SKAP2	0			TPP1	1.42E-240	LARP7	4.84E-178	ABHD5	0	PLA1A	1.47E-142
PRKCZ	3.91E-189	PRSS12	0	ETV5	0	LSS	5.14E-256	PLA2G12A	8.97E-272	LSM7	1.34E-136			EVI2A	1.01E-109	ADAMDEC	0			HBEGF	6.28E-240	FCGRT	1.15E-176	SLA	0	ADAM15	1.5E-142
ATOH8	2.61E-188	GPR162	0	KLF13	0	FAM129B	2.54E-255	KLF5	8.98E-272	PPM1K	1.59E-136			EIF3K	1.46E-109	ARPC1B	0			HSD17B11	7.45E-240	TPP1	2.22E-174	RNF13	0	FOXC1	2.9E-142
NFIA	2.77E-188	SPATA17	0	FHDC1	0	WWC3	1.12E-252	FAM210B	6.09E-271	CPNE5	2.92E-136			MYO1G	2.24E-109	CORO1C	0			MCTS1	1.9E-239	SNRPB2	2.8E-174	GSN	0	ZNF521	8E-142
SERPINH1	3.73E-188	TMEM212	0	EFNA1	0	CGNL1	1.78E-252	SH3YL1	7.75E-271	PBXIP1	3.1E-136			PREX1	7.95E-109	PIK3AP1	0			OPN3	8.64E-239	GPX3	1.27E-172	SEPT11	0	DKK3	4.79E-141
TMPRSS2	4.7E-188	DCDC1	0	EPS8	0	NFIX	2.59E-252	RAB5B	5.81E-270	CD6	3.57E-136			SULF2	4.04E-108	LILRB3	0			HLA-DMA	4.34E-238	HLA-DMB	1.98E-172	ATP1B3	0	SMTN	7.29E-141
WTIP	2.3E-187	KIF19	0	ATP8A1	0	NUP58	8.58E-252	DCXR	6.04E-270	RANBP1	5.64E-136			IL10RA	6.22E-107	HAMP	0			AAK1	3.32E-237	LSAMP	6.99E-172	SERF2	0	TNS2	2.5E-140
IFT57	1.33E-186	LINC01571	0	SLC39A8	0	HMGCR	4.13E-251	MTRR	6.59E-270	CFL1	5.85E-136			CMTM6	6.46E-107	HSD3B7	0			HLA-DQA	3.37E-237	THEMIS2	1.86E-171	THBD	0	FBLN1	1.21E-139
LIPH	1.34E-185	SLITRK6	0	RP11-532F	0	LAPTM4B	2.84E-250	PACSIN2	2.81E-269	MAP4K1	1.92E-135			CMTM3	6.99E-107	HS3ST2	0			GNAS	1.03E-236	PLAUR	5.65E-170	LRP1	0	PRELP	4.04E-139
GIPC1	1.47E-185	CCDC40	0	RMDN2	0	ITPR3	3.48E-250	SCNN1A	1E-268	RPL9	8.48E-135			RPL35	4.16E-106	ENG	0			CAP1	5.83E-236	RUNX1	1.16E-169	ADAM17	0	ADGRL2	1.11E-138
CHD3	5.64E-185	ANKRD66	0	AC090616.	0	PRDX2	4.37E-250	TMEM245	5.71E-268	RP11-160E	6.18E-133			TPI1	4.41E-106	MRAS	0			CYP27A1	2.3E-235	LTA4H	4.01E-169	TRPV2	0	DNASE1L3	1.53E-138
TEAD1	1.88E-184	DNAI1	0	MVD	0	CDK2AP2	4.56E-250	CYBRD1	2.83E-267	RPS16	2.31E-130			RBM3	5.39E-106	PLXNC1	0			OSBPL11	3.83E-234	IFIT3	4.34E-169	CTSL	0	MEIS2	3.76E-138
ARHGEF12	2.33E-184	BAIAP3	0	ENSA	0	SLC22A31	2.53E-249	WBP5	3.17E-267	RPL27	1.87E-129			KYNU	5.59E-106	LRP1	0			RALBP1	1.22E-233	SSB	8.5E-169	COX4I1	0	COL1A1	4.31E-138
CKB	1.06E-183	CFAP69	0	RPL35A	0	EGFR	1.65E-248	NKX2-1	3.26E-267	ATM	5.49E-129			ARHGAP3	9.17E-106	MARCO	0			OSTF1	2E-233	OAS2	1.57E-168	NDUFV2	0	CARHSP1	4.91E-138
PPP4R1	1.56E-183	C1orf168	0	SNHG19	0	PPP1R9A	1.77E-247	SQLE	5.21E-267	IL1RL1	9.25E-129			DRAP1	4.67E-105	IL4I1	0			ANXA1	2.3E-233	HERC5	4.4E-168	C20orf27	0	TACC1	6.26E-138
CTNND1	1.89E-183	PACRG	0	HMGN3	0	ANXA3	4.56E-247	TMEM163	2.33E-266	ARHGAP2	1.8E-128			LAT2	7.62E-105	TM4SF19	0			MOB3B	2.4E-233	AKR1B1	3.78E-167	IFI30	0	PEAR1	8.56E-137
PLXNA1	2.99E-183	CFAP74	0	SLC22A31	0	ETV5	1.92E-246	SLC25A4	2.37E-266	IFI16	4.27E-128			RPL8	1.74E-104	HLA-DQA	0			PAK2	1.6E-231	GMFG	5.71E-167	TALDO1	0	GPR4	2.37E-135
COL4A3	1.34E-182	CCDC189	0	ANAPC16	0	FAM213A	4.98E-244	TSC22D1	5.65E-266	RPL24	5.21E-128			IFITM2	1.91E-104	ZNF385A	0			TREM1	1.74E-229	SNX3	1.35E-166	RHBDD2	0	SHANK3	2E-134
TES	1.7E-182	TTLL10	0	SPINT2	0	USP13	8.45E-244	PPP3CA	1.7E-264	CIRBP	5.63E-128			FKBP1A	3.24E-104	ATP5E	0			CARD16	2.42E-229	ADTRP	3.02E-166	RGCC	0	CRISPLD2	3.37E-133
ITGB6	3.61E-182	CASC2	0	PIP5K1B	0	AFF3	2.67E-243	SCN1A	7.03E-263	POU2AF1	1.62E-127			CDA	5.63E-104	CD163	0			CCL18	3.19E-229	MDH1	8.95E-166	LEPROTL1	0	FKBP10	4.22E-132
ATP1A1	5.1E-182	LINC01513	0	RPL3	0	SLC12A2	8.47E-243	ALOX15B	2.18E-262	ELF1	2.5E-126			ARRB2	1.77E-103	MSR1	0			PSMC1	4.74E-229	DUSP1	9.99E-165	HDDC2	0	SEMA3F	5.05E-132
GTF2I	4.45E-181	FRMPD2	0	CREB3L1	0	RBPMS	2.43E-242	HHIP-AS1	1.97E-261	RSRP1	2.76E-126			FCGRT	1.08E-102	PLBD2	0			UBASH3B	7.53E-229	PTGER4	4.43E-164	EVL	0	CLIC4	2.74E-131
SERPIND1	4.74E-181	DAW1	0	RPL9	0	TC2N	7.22E-240	PPP1R9A	2.22E-261	GSTK1	3.91E-126			RPS4Y1	1.31E-101	IL10RA	0			RAB31	1.88E-227	EML4	1.35E-163	AP2S1	0	HES1	6.25E-131
CCND2	1.84E-180	MORN3	0	SEC61G	0	MRPL14	1.18E-238	RASGRF1	1.9E-260	MALAT1	4.24E-126			C15orf48	1.58E-101	CXCL16	0			MFSD14B	3.49E-227	PGD	1.45E-163	CLEC2B	0	FLNA	7.84E-130
NT5DC2	1.99E-180	C1orf87	0	RPL19	0	ACADL	2.44E-238	DUOX1	2.97E-259	CHMP4A	9.79E-126			PLEK	1.96E-101	HCK	0			SLC3A2	6.09E-227	AKIRIN2	1.75E-163	MAP3K8	0	ELK3	1.19E-129
TNKS1BP1	2.13E-180	CCDC65	0	TMEM164	0	CA2	3.42E-237	ERRFI1	3.62E-259	EEF2	1.69E-125			LILRB3	7.49E-101	MT-CO2	0			ZBTB7A	7.24E-227	SEC11A	6.65E-161	HIGD2A	0	PLCB4	1.39E-129
CTGF	2.65E-180	SIX1	0	H1F0	0	NCKAP1	9.11E-236	AREG	7.54E-259	TAF7	2.25E-125			ARPC2	7.95E-101	CD74	0			RUNX1	2.08E-226	PELI1	3.97E-159	TIMP2	0	COL15A1	1.69E-129
F11R	4.77E-180	PIH1D2	0	P3H2	0	RAI14	1.27E-235	RBPMS	6.58E-258	HLA-F	2.26E-125			NUDT16	1.13E-100	PRKCB	0			ZEB2	1.22E-225	SLC35E3	4.78E-159	LSM6	0	ARGLU1	1.95E-129
MET	8.66E-180	C2orf73	0	WWC3	0	NFIC	2.05E-234	RNF141	4.81E-257	GIMAP4	4.86E-125			PSTPIP2	2.24E-100	SNCA	0			IAH1	1.7E-225	DOK2	1E-158	FUOM	0	CCL23	1.03E-128
FAM174B	3.41E-179	IQCD	0	KRT18	0	FADS2	3.02E-234	MKNK2	3.81E-256	SNRPB	9.51E-125			INPP5D	6.09E-100	LY96	0			STX7	1.25E-224	MAP2K1	1.09E-157	TCF7L2	0	COL6A3	2.41E-128
MALL	8.23E-179	C1orf158	0	EPHX1	0	FZD5	6.11E-232	OCLN	1.28E-255	NPM1	1.34E-124			FES	6.59E-100	HK3	0			BNIP2	2.42E-224	DYNLL1	3.54E-157	BCAP31	0	GSN	3.33E-127
TMEM54	1.69E-178	MRLN	0	NTN4	0	PRKCI	6.75E-231	CDS1	1.47E-255	SSR2	1.92E-124			NDRG2	1.1E-99	DFNA5	0			STXBP2	1.17E-223	TOMM40	3.81E-157	RHOG	0	KANK3	5.87E-127
CLSTN1	2.15E-178	CROCC2	0	SYNE1	0	INSIG1	8.02E-231	GALNT10	2.96E-255	BCL2	1.73E-122			ADAM19	2.09E-99	HLA-DPB1	0			CD44	1.34E-223	ACOT2	7.88E-157	TNFSF12	0	PXDN	2.6E-126
PDGFA	2.58E-178	DNAH3	0	RPL11	0	PTPRG	1.14E-230	ROS1	3.23E-255	PNISR	5.77E-122			WIPF1	3.75E-99	CMKLR1	0			RABGGTB	6.31E-223	KLHL6	1.92E-156	RPS19	0	SHE	3.4E-126
LAMB2	3.07E-178	DCDC2B	0	CLIC3	0	SHROOM3	2.36E-230	SPTSSA	8.38E-255	RPA2	6.33E-122			FAM49A	4.28E-99	HLA-C	0			TMBIM1	6.75E-223	RNF130	5.59E-156	C19orf70	0	APOLD1	5.71E-126
HOOK2	4.11E-178	PPP1R32	0	TOMM7	0	LL22NC03-	4.26E-230	RAI14	3.43E-254	GABPB1-A	7.91E-122			FERMT3	4.6E-99	ADM	0			UBE2E2	8.6E-223	FCGR1A	3.47E-154	DNASE2	0	GIMAP6	4.96E-125
ATHL1	1.24E-177	MARCH10	0	ADIRF	0	SH3YL1	5.2E-230	DNAJC21	9.15E-254	FKBP8	1.21E-121			EEF1B2	4.95E-99	SMPDL3A	0			LPXN	1.03E-222	CRTAM	6.84E-154	MME	0	LTBP4	5.05E-125
PHLDB2	1.38E-177	LRRC71	0	PXMP4	0	TEAD1	5.65E-230	SC5D	1.19E-253	BCAS4	3.5E-121			PLEKHO1	1.029E-98	RASSF4	0			DOCK11	1.84E-222	GPX4	5.81E-153	SARAF	0	DKK2	6.22E-125
LIMCH1	9.95E-177	RIBC1	0	EEF1A1	0	TOP1	1.51E-229	LAPTM4B	4.67E-253	PPP1CA	3.72E-121			RIN3	3.825E-97	LACTB	0			ARPC5	1.61E-221	MARCH1	2.93E-152	PTPMT1	0	PDLIM3	7.79E-125
CST6	1.17E-176	CFAP77	0	STEAP4	0	LIPH	2.03E-229	PFKFB2	9.96E-253	PRKCB	1.91E-120			PSMB8	8.335E-97	SLC8A1	0			EPB41L4A	1.37E-220	EGR2	1.96E-151	BLVRA	0	ICAM2	1.3E-124
SLC15A2	1.63E-176	CFAP57	0	QDPR	0	AQP3	5.29E-229	SEPP1	6.8E-252	UBE2I	4.36E-120			LILRB1	1.918E-96	NRP1	0			CMC2	2.45E-220	YWHAZ	7.29E-151	RPL15	0	HRCT1	2.05E-124
AES	2.02E-176	CCDC74B	0	BAD	0	EVA1A	4.01E-227	BEX2	1.99E-251	HNRNPA0	1.74E-119			FMNL1	2.424E-96	FAM96A	0			TRPV2	2.51E-218	USMG5	2.32E-150	SAP18	0	EFEMP1	2.38E-124
HDAC7	9.6E-176	EFHC2	0	RPL29	0	TM7SF2	5.24E-227	HEBP2	4.34E-251	CDC42SE2	2.62E-119			CASP1	6.37E-96	ARRB2	0			PABPC1	3.71E-218	MX1	2.33E-150	CIB1	0	LAMB2	4.01E-124
FZD6	3.95E-175	C4orf22	0	C11orf54	0	ERLEC1	5.63E-227	LRP2	7.87E-251	C17orf62	2.7E-119			ITGAM	1.093E-95	EPB41L3	0			CLEC12A	2.09E-215	GPRIN3	4.53E-150	BID	0	AFAP1L1	1.16E-123
AGR2	8.64E-175	WI2-1959D	0	PID1	0	PRSS8	3.53E-226	CDK2AP2	1.72E-250	ARHGEF1	4.74E-119			HHEX	3.465E-95	SH3BGRL	0			THEMIS2	2.11E-215	ARF6	8E-150	FAM89A	0	PTPN14	4.59E-123

Cluster 1 (airway and alveolar epithelial cells)		Cluster 2 (ciliated cells)		Cluster 3 (type II alveolar cells-1)		Cluster 4 (type II alveolar cells-2)		Cluster 5 (type II alveolar cells-3)		Cluster 6 (immune cells)		Cluster 7 (plasma cells)		Cluster 8 (monocytes-1)		Cluster 9 (monocytes-2)		Cluster 10 (alveolar macrophages-1)		Cluster 11 (alveolar macrophages-2)		Cluster 12 (alveolar macrophages-3)		Cluster 13 (alveolar macrophages-4)		Cluster 14 (mesenchymal cells)	
Gene symbol	Adjusted p values	Gene symbol	Adjusted p values	Gene symbol	Adjusted p values	Gene symbol	Adjusted p values	Gene symbol	Adjusted p values	Gene symbol	Adjusted p values	Gene symbol	Adjusted p values	Gene symbol	Adjusted p values	Gene symbol	Adjusted p values	Gene symbol	Adjusted p values	Gene symbol	Adjusted p values	Gene symbol	Adjusted p values	Gene symbol	Adjusted p values	Gene symbol	Adjusted p values
KLK10	2.01E-174	PIH1D3	0	KCNJ15	0	SPAG9	5.1E-225	ZDHHC21	4.33E-250	HLA-DOB	3.55E-118			RNF130	7.698E-95	MOB1A	0			ADAMTSL	2.89E-214	UBASH3B	1.77E-149	HEXA	0	TNFSF10	9.02E-123
MAGI1	2.03E-174	GRIN3B	0	RAI14	0	CAV2	1.16E-223	AGR3	5.16E-250	GMFG	5.32E-118			EMB	9.521E-95	ATP6V0B	0			APOL6	2.16E-213	FKBP15	2.89E-149	PRR13	0	EFNB2	1.66E-122
PALLD	2.47E-174	RIBC2	0	ATP11A	0	CREB3L1	1.33E-223	NCKAP1	6.55E-250	TMEM154	1.18E-117			TNFAIP8	4.933E-94	ARPC4	0			PDE4DIP	3.54E-213	GPCPD1	2.78E-148	SLC16A3	0	LUZP1	1.87E-122
TNS1	2.52E-174	RP11-347J	0	IRX2	0	GALNT3	4.84E-223	MRC2	9.89E-250	SMARCB1	1.23E-117			GIMAP1	5.392E-94	CYTH4	0			SP100	1.6E-212	LILRA6	3.5E-148	AZI2	0	APOD	2.96E-122
TAX1BP3	4.37E-174	RP11-193M	0	RPL7A	0	RP11-259K	5.7E-223	FAM213A	2.3E-249	FUS	2.66E-117			NAMPT	1.172E-93	CDC42	6.44E-308			TIMP2	2.89E-212	FILIP1L	4.5E-148	IL1B	0	PALM	3.42E-122
TMC4	8.29E-174	TTC29	0	LGI3	0	NEDD9	4.5E-222	RAP1GAP	4.36E-249	DUT	3.39E-117			ACTR3	1.436E-92	ARPC3	7.24E-307			IFI6	3.99E-211	OTUD1	4.51E-147	HCK	0	LYVE1	7.92E-122
CAPN8	1.39E-173	CFAP206	0	LAPTM4B	0	ASPH	5.24E-222	STXBP1	1.2E-248	RP11-347P	6.99E-117			FYN	1.963E-92	DSE	8.35E-306			PLXDC2	5.3E-211	PRDX1	1.33E-146	AKR1C2	0	DOCK9	4.6E-121
GALNT5	3.85E-173	CLUAP1	0	C14orf132	0	FOS	7.56E-222	STARD10	2.61E-248	WIPF1	1.47E-116			LINC00152	2E-92	SLC38A6	3.48E-304			DOCK8	3.77E-210	ATP10A	1.49E-146	CLEC7A	0	GJA5	1.35E-120
ABCC3	4.76E-173	CC2D2A	0	IVD	0	LRP6	5E-220	TRIP11	2.95E-248	SUSD3	9.13E-116			CDC42	2.104E-92	IL17RA	7.16E-304			GLIPR2	2.37E-209	PTPMT1	2.53E-146	RNF130	0	PREX2	2.28E-120
IRX3	1.24E-172	LRRC34	0	RPL14	0	LNX2	1.75E-219	ABCC4	4.58E-248	IKZF1	4.09E-115			CFD	3.435E-92	BCAT1	2.34E-303			RMDN3	3.46E-209	LAMP1	2.56E-146	H2AFJ	0	CPE	3.9E-120
LAPTM4B	1.31E-172	FXYD3	0	CTNNBIP1	0	MYO5C	2.5E-219	TC2N	1.67E-247	PLP2	9.28E-115			FNBP1	4.142E-92	ATP13A3	2.27E-302			YWHAH	5.17E-209	SLC31A2	2.8E-145	VPS29	0	DOC2B	3.17E-119
CLIC3	2.07E-172	TTLL9	0	CEBPA	0	ERRFI1	3.47E-216	CA2	8.42E-247	FBL	9.63E-115			DSE	8.323E-92	NRP2	4.97E-301			COA6	3.24E-208	SIGLEC1	4.13E-144	CSNK2B	0	PALLD	4.62E-119
GRAMD3	2.44E-172	RRAD	0	TMEM243	0	RAB25	8.42E-216	TRIB1	1.27E-246	ARPC4	2.57E-113			CAMK1	1.302E-91	GPR65	6.84E-299			POLD4	2.6E-207	LCP2	9.96E-144	YBX1	0	WWTR1	9.61E-119
CGN	2.89E-172	MAP6	0	MECOM	0	DUSP6	1.69E-214	ACSS2	2.2E-246	NACA	1.6E-112			SRGAP1	4.333E-91	MERTK	7.36E-298			FRG1	1.63E-206	DNAJA1	1.52E-143	LAMP2	0	BMX	2.12E-118
LGALS3BP	3.35E-172	RP11-263K	0	CCDC141	0	SECISBP2L	1.76E-214	RHOB	4.34E-246	CYTIP	3.15E-112			IFNGR2	1.425E-90	SLA	2.9E-296			MAN1A1	3.86E-206	ZBTB8OS	2.02E-143	KLF4	0	DST	2.62E-118
COL4A2	5.93E-172	DMKN	0	FAM213A	0	VEGFA	7.89E-213	ARHGEF1	1.08E-245	COPE	1.3E-111			MARCKS	3.695E-90	RIN2	3.12E-295			OSCAR	1.29E-205	MT2A	2.58E-143	FABP5	0	ADGRG6	1.6E-117
IGFBP4	1.04E-171	TFF3	0	BMP2	0	FILIP1	2.36E-212	EGR1	7.42E-245	MARCKSL	4.13E-111			IFNGR1	5.703E-90	ARPC2	3.63E-295			CITED2	1.85E-205	SH3GLB1	3.8E-143	PECAM1	0	CCDC3	7.07E-117
ODF2L	5.29E-171	SYT8	0	RPL23A	0	THOC2	2.46E-212	CCDC14	9.18E-245	ADAM28	1.03E-110			CLECL1	2.535E-89	SLC29A3	5.3E-293			ADGRE5	2.38E-205	GNAI2	4.43E-142	PSME2	0	FLT4	1.23E-116
ARHGAP3	6.05E-171	MAP9	0	PRKAR1A	0	NR4A1	3.98E-212	C14orf1	3.06E-244	EIF3G	1.02E-109			PSMB8-AS	2.973E-87	NTAN1	4.47E-292			SMS	7.42E-205	SAT1	1.26E-139	FRMD4A	0	SYNPO	2.99E-116
PDLIM2	1.35E-170	ST6GALN	0	ACAT1	0	DESI2	1.31E-211	ABCD3	1.1E-243	SRSF2	3.05E-109			CSK	3.361E-87	PAPSS1	3.44E-291			GPRIN3	1.04E-204	CD83	2.95E-139	COX5B	0	TJP1	6.11E-116
NTM	3.66E-170	LCA5	0	MRPL33	0	PMM1	9.4E-210	PIP5K1B	2.42E-243	TSEN54	4.64E-109			BASP1	3.646E-87	CHST11	2.76E-290			FKBP5	2.53E-204	GYPC	5.84E-139	RAB13	0	AIF1L	1.22E-115
RARRES3	4.14E-170	FTO	3.61E-308	SELM	0	HHIP-AS1	6.81E-209	MED13	2.79E-243	CCDC85B	5.29E-109			BID	4.872E-87	GNS	1.52E-287			MAP2K1	2.82E-204	MFSD1	6.23E-139	AMIGO2	0	PLCXD3	1.25E-115
RARRES2	6.29E-170	PERP	2.93E-307	KRT8	0	DBI	7.85E-209	TCEA3	9.4E-242	PCSK7	6.65E-109			NAGA	1.458E-86	ACTN1	2.01E-286			PNPLA6	1.42E-203	UGP2	9.64E-139	PRDX1	0	FBLIM1	3.12E-115
MYO6	2.28E-169	SPAG1	6.14E-307	PON2	0	FNIP2	9.15E-209	PPP4R1	1E-241	ANP32E	4.36E-108			LAMTOR4	1.492E-86	ITGA5	3E-285			IFNGR1	5.27E-203	ATP1B3	1.8E-138	OASL	0	TPM4	6.76E-115
TRAM1	8.27E-169	TCTN1	7.08E-307	AARD	0	TMPRSS2	1.43E-208	TM4SF1	1.3E-241	RP11-596C	5.34E-108			HLA-DMB	1.885E-86	SPARC	1.93E-283			ATP1B3	1.97E-202	FRG1	1.1E-137	RPL36AL	0	TGFBR2	1.93E-114
HMGB3	9.97E-169	RFX2	1.22E-306	EVA1A	0	AKAP1	7.35E-208	ACACA	2.03E-241	SEPT9	8.4E-108			RPL36A	2.415E-86	ACE	6.76E-281			ACOT7	8.98E-202	PDLIM1	1.19E-137	C19orf60	0	MATN2	1.16E-113
YAP1	1.01E-167	WDR93	1.83E-306	RPL31	0	EPHX1	1.66E-207	RNASE1	3.38E-241	EEF1A1	2.82E-107			MXD1	4.607E-86	PLD3	2.21E-280			MMP19	1.28E-201	COA6	1.35E-137	BTF3	0	FSCN1	2.46E-113
IGFBP6	1.65E-167	NELL2	1.16E-304	USP53	0	C1orf116	1.7E-207	CAMK2D	4.84E-241	C19orf43	1.69E-106			HIF1A	1.118E-85	RNF13	6.39E-280			HEXB	9.46E-200	CLIC1	3.64E-137	CD83	0	GRK5	8.9E-113
ANKRD1	1.92E-166	AGBL2	2.06E-304	RPL18A	0	MAP3K13	4.91E-207	C3	1.31E-240	TCF7	4.39E-106			LCP2	1.635E-85	RGS2	1.31E-278			GABARAP	2.81E-199	RAB11FIP1	2.42E-136	CTSZ	0	IGF2	1.18E-112
FGFR3	3.84E-166	ECT2L	2.11E-302	SPSB2	0	NEBL	1.96E-206	FOS	6.48E-240	VWA5A	1.39E-105			ARHGAP4	2.273E-85	DHRS9	1.46E-278			FCF1	5.51E-199	IL1RAP	2.47E-136	UBASH3B	0	ARHGAP2	1.5E-112
ABCA7	3.95E-166	PALMD	1.37E-300	RPL36	0	TJP1	5.81E-206	HMGCR	8.41E-240	RALGPS2	4.24E-105			SRGAP2C	4.184E-85	IFI30	1.17E-277			CGGBP1	8.54E-199	SAMSN1	4.25E-136	TMEM14C	0	BTNL9	2.34E-112
CRLF1	3.24E-165	CCDC81	3.27E-300	PNKD	0	GDF15	6.5E-206	CHI3L2	1.49E-239	ITGB7	5.89E-105			PSME2	7.071E-85	ADAM10	1.38E-276			IL1RAP	2.93E-198	ANPEP	5.97E-136	PSMB3	0	SYNPO2	2.38E-112
SLC4A4	7.06E-165	C17orf97	4.93E-300	TMPRSS2	0	MAGI3	1.98E-204	KIF1C	9.3E-239	UXT	8.18E-105			CYTH4	1.62E-84	CSF2RB	2.81E-276			SLC31A1	3.45E-198	CECR1	1.54E-135	GPCPD1	0	GPRC5B	5.6E-112
PVRL4	9.04E-165	LCA5L	2.45E-299	RPS25	0	CD47	2.99E-203	MAPK13	6.4E-238	HMHA1	1.05E-104			SRGAP2B	2.444E-84	LPXN	8.82E-276			FPR2	2.2E-197	CPE	1.75E-135	CASP1	0	SULF2	6.65E-112
MSLN	9.89E-165	IFT172	9.42E-299	RAB17	0	CTTN	3.4E-203	IRX2	7.11E-238	APOLD1	1.82E-104			TWF2	2.487E-84	FGR	1.87E-275			MMD	5.84E-197	CAPZA1	5.7E-135	MTPN	0	CCDC80	9.89E-112
GGT1	1.07E-164	SRD5A2	2.2E-298	CMTM8	0	FOXP1	1.69E-201	SMC4	8.5E-238	SH3KBP1	4.76E-104			PGAM1	2.618E-84	KYNU	1.18E-274			EVL	1.36E-196	C3AR1	5.91E-134	RGS10	0	PDK4	1.09E-111
EDN1	1.25E-164	CCDC180	8.9E-298	TCEA3	0	CPNE3	8.61E-201	NFATC3	2.42E-237	FAM65B	5.31E-104			SPTLC2	5.59E-84	ATP6V1A	2.47E-273			TFEC	6.1E-196	GAS2L3	6.34E-134	NUCB1	0	CSGALNA	1.14E-111
ACTN4	5.8E-163	NUDC	1.08E-297	OCLN	0	PXMP4	2.18E-200	PDZD2	5.28E-236	TXNIP	5.45E-104			BST2	5.913E-84	ZMIZ1	1.35E-272			AGRP	8.73E-196	DDX21	7.44E-134	GABARAP	0	PDGFRB	4.1E-111
TMEM125	6.99E-163	B9D1	3.95E-297	CXADR	0	CPM	2.19E-200	EGFR	2.39E-235	UBE2D2	2.21E-103			RP11-796E	9.587E-84	FCGR2A	9.02E-272			SLC35E3	1.4E-195	STAC	2.27E-132	BRK1	0	ITGA1	4.65E-111
ST6GAL1	7.53E-163	LRRC4	2.39E-296	ATP1B1	0	CDKL2	9.39E-200	RPS6KA2	1.61E-234	IFITM2	6.11E-103			RAB31	1.027E-83	VAT1	2.27E-270			SOAT1	1.07E-194	FFAR4	3.74E-131	DNASE2B	0	MYH10	4.93E-111
TSC22D1	9.14E-163	TTC21A	3.06E-296	RPS11	0	SDPR	9.99E-198	SFTA3	2.08E-234	ARID4B	7.83E-103			APBB1IP	4.391E-83	ME2	5.05E-269			ACTR2	2.46E-194	UQCRH	1.08E-130	RENBP	0	SASH1	5.37E-111
MLLT4	1.16E-162	LRRC74B	6.72E-294	PRR15L	0	MAST4	1.52E-197	MYO1B	4.35E-234	ID2	1.98E-102			GRB2	4.106E-82	NCKAP1L	6.04E-269			ATP6V1B2	4.26E-193	SRP14	4.35E-130	RPL18A	0	CRYAB	6.7E-111
LLGL2	1.58E-161	TSPAN6	1.98E-293	GALE	0	MYH14	7.78E-197	XBP1	9.87E-234	BIN2	2.89E-102			PSMB10	5.761E-82	CXCL9	6.21E-268			PLBD1	1.1E-192	EREG	6.29E-130	ETHE1	0	CNRIP1	7.56E-111
FGFR2	1.94E-161	RP11-122M	2.02E-292	RPS17	0	CDKL5	2.5E-195	RAB3IP	1.08E-233	TMA7	5.89E-102			METTL9	9.122E-82	SOAT1	5.66E-267			USP15	1.33E-191	PILRA	6.6E-129	ISG15	0	ANGPT1	1.92E-110
AQP1	2E-161	MUC15	8.14E-292	EHF	0	GPD1L	1.12E-193	AC013461.	1.1E-233	HNRNPM	7.8E-102			PPP1CA	2.359E-81	HSD11B1	2.9E-266			FGR	7.12E-191	ACSL1	9.61E-129	HCLS1	0	PLK2	4.74E-110
ABHD2	9.2E-161	NHLRC4	1.06E-291	PON3	0	IER2	1.55E-193	ARHGAP5	2.87E-233	RPL38	1.37E-101			THEMIS2	1.596E-80	KCNN4	5.69E-266			ZNF706	1.73E-189	ZFAS1	1.04E-128	CTBS	0	CAMK2N1	9.56E-110
BTG3	1.67E-160	EFCAB12	1.09E-291	GPD1L	0	TCEA3	2.56E-192	ESYT3	4.44E-233	SRSF5	1.81E-101			STK4	3.926E-80	PPP1R18	6.45E-266			PFDN4	4.57E-189	CAP1	1.15E-128	ARHGDIB	0	DDR2	1.06E-109
LRRC8A	1.74E-160	TRAF3IP1	8.21E-291	CCDC69	0	CERS2	2.67E-192	TIPARP	5.31E-233	HMGB1	1.96E-101			CARD16	2.422E-79	IL7R	6.17E-265			ENPP4	3.35E-188	DNASE2	1.21E-128	CYCS	0	EBF1	3.83E-109
FARP1	2.24E-160	TEKT4	1.3E-290	RPS3A	0	MPZL2	2.81E-191	CD59	5.89E-233	MYCBP2	2.86E-101			LIMS1	2.918E-79	SNX9	6.75E-264			QKI	4.74E-188	ZFP36L2	1.4E-128	ERP44	0	HEY1	2.34E-108
C3	4.07E-160	GAS2L2	2.45E-290	UQCRQ	0	SLC25A23	3.57E-191	PTPRG	7.29E-233	RP11-51J9.	6.53E-101			ZYX	5.088E-79	NAGA	6.89E-264			NUP214	1.23E-187	SAMD9L	5.03E-128	KAT8	0	PMP22	3.36E-107
IL1R1	7.19E-160	TP73	3.53E-290	PLS3	0	STXBP1	1.09E-190	CEP70	8.2E-232	BUB3	6.74E-101			NCF1	5.021E-78	POMP	2.37E-261			NCEH1	1.83E-187	STOM	1.89E-127	RP11-356N	0	THSD7A	4.06E-107
SLC25A23	8.97E-160	DNAJB13	5.69E-290	KRTCAP3	0	STARD10	8.71E-190	FZD5	1.85E-231	SAMSN1	1.73E-100			C4orf48	5.501E-78	ENO1	2.88E-261			RP3-460G2	2.78E-187	BLVRA	2.05E-127	SFT2D1	0	TSPAN9	6.26E-107
CCDC64B	6.41E-159	CFAP47	1.59E-289	DHCR7	0	TACC1	1.67E-188	SLC22A31	3.67E-231	PNN	1.94E-100			PLIN3	1.056E-77	IFI16	3.16E-261			FRMD4A	3.99E-187	QKI	2.83E-125	SELPLG	0	MACF1	8.35E-107
TBL1XR1	7.18E-159	RPGRIP1L	3.46E-287	RPL27A	0	PTPRF	3.91E-188	SNHG25	1.17E-230	TRABD	5.5E-100			FGR	1.908E-77	HM13	3.43E-259			NCKAP1L	3.99E-187	PTPN12	5.29E-125	TGFB1	0	NFIA	1.25E-106
NFATC3	1.73E-158	KCNRG	1.07E-286	RAB3IP	0	HACD3	2.08E-187	SLC44A1	3.6E-230	OCIAD2	1.88E-99			ZNF385A	2.553E-77	MT-CO3	3.24E-258			MYADM	1.04E-185	SCCPDH	1.01E-124	PHLDA1	0	C2CD4B	9.89E-106
OCLN	2.28E-158	IFT57	4.63E-285	MAP3K13	0	AKR1C1	2.95E-187	PID1	6.31E-230	MBP	3.18E-99			WASF2	5.005E-77	HLA-DMA	3.91E-257			DRAM2	1.57E-185	OSCAR	5.53E-124	COX6B1	0	CTHRC1	1.1E-105
SERPINF1	5.74E-158	C14orf142	7.49E-285	RPS16	0	AMD1	5.86E-186	IL20RA	8.74E-230	PSTPIP1	3.74E-99			MOB1A	1.194E-76	PRCP	6.03E-257			PHLDA3	4.67E-184	KCNAB2	3.69E-123	EFHD2	0	FGF2	1.4E-105
ARHGAP2	7.88E-158	TNFRSF19	2.06E-284	CGNL1	0	CAT	4.43E-184	SLC5A3	1.15E-229	SNRNP70	4.985E-98			LGALS9	7.105E-76	TMEM51	9.83E-256			GSTO1	6.13E-184	TGOLN2	1.29E-121	LMNA	0	INPP1	1.68E-105
EHF	1.43E-157	LKAAEAR	1.22E-283	PMF1	0	CRB3	5.37E-184	MYO9A	5.4E-228	NOSIP	5.738E-98			CCR1	1.208E-75	AKR1A1	1.27E-254			UPP1	1.41E-183	HSPA1A	3.43E-121	CHCHD2	0	CD200	3.48E-105
SEMA5A	2.84E-157	CCDC60	8.3E-283	HACD1	0	UNC13B	2.47E-183	GALNT3	6.73E-228	TMC8	1.479E-97			NUP214	1.534E-75	SLAMF7	1.73E-254			GPD1	6.84E-183	HCAR2	3.92E-121	RGS19	0	SNRK	7.51E-105
MYO1D	4.64E-157	CCDC191	6.78E-282	RPLP1	0	ID4	2.17E-182	SLC22A3	6.86E-228	SP100	2.58E-97			ASGR1	3.172E-75	VDAC1	2.69E-254			SCCPDH	1.63E-182	ARRDC4	7.1E-121	MYDGF	0	IL1R1	8.68E-105
CXCL17	8.24E-157	WDR49	6.29E-281	SLC38A1	0	HACD1	4.07E-182	PLXND1	1.58E-227	IGLC3	3.495E-97			C3AR1	4.168E-75	MPEG1	1.41E-253			FCGR1A	5.01E-182	TMEM50A	1.54E-120	RPL27A	0	MAP1B	1.42E-104
WDR34	1.35E-156	PLAC8	4.06E-280	KIAA1109	0	MVD	9.57E-182	TMEM41A	1.92E-226	WDR74	1.347E-96			NAP1L1	4.482E-75	PLIN3	8.24E-252			MME	5.09E-182	SEMA3C	6.48E-120	IRF8	0	FXYD1	1.78E-104
PTPRK	1.82E-156	HIPK1	7.57E-280	AC079630.	0	SLC25A5	9.69E-182	FOSB	4.84E-225	SIGIRR	1.427E-96			TALDO1	4.613E-75	PLAU	1.38E-251			CKLF	2.44E-180	MAN1A1	8.81E-120	PLA2G16	0	EPHB4	5.11E-104
TANC1	3.95E-156	CACNG6	1.21E-279	UNC13B	0	MYO6	1.12E-181	SLC46A2	8.5E-225	SYTL1	4.053E-96			COX4I1	5.98E-75	CD83	1.65E-251			FHL1	8.23E-180	TEX2	1.44E-119	GNAI2	0	FERMT2	5.43E-104
CUX1	4.59E-156	FBXO15	4.96E-279	RP4-569M	0	C12orf49	1.44E-180	PCYOX1	2.11E-224	PPP1R18	7.926E-96			ADAP2	1.184E-74	SDC3	3.5E-249			BACH1	9.7E-180	GPD1	1.96E-119	CLTA	0	EHD2	7.54E-104
UTRN	6.1E-156	CCDC34	6.98E-279	ITPR3	0	C14orf132	4.96E-180	KANK1	6.46E-224	CUTA	9.683E-96			BIN2	2.275E-74	C1orf122	1.85E-248			CXCL5	1.6E-179	ANKRD28	1.34E-118	ACOT7	0	PLEKHG1	1.23E-103
TMEM30B	9.45E-156	EPPIN	1.33E-277	ANP32E	0	GLO1	1.81E-177	C12orf49	6.82E-224	TBCB	1.241E-95			ALDOA	2.291E-74	PLEKHO2	3.52E-248			PILRA	2.53E-179	VDAC1	1.43E-118	TUBA1B	0	CCDC50	2.31E-103
POLR2L	1.11E-155	SPATA33	2.64E-277	RPL37A	0	FAM184A	2.58E-177	SYT15	7.33E-224	HIST1H2B	3.399E-95			RP11-160E	1.359E-73	WDR1	1.17E-246			IFRD1	6E-179	PKP2	5.05E-118	LSM4	0	C1orf115	4.34E-103
FAM20A	1.37E-155	CCL15	3.7E-277	MUC15	0	CLIC3	7.55E-177	CDKL2	1.33E-223	SS18L2	4.991E-95			RASSF4	1.861E-73	P2RX4	3.73E-245			PSME4	2.13E-177	FOS	9.27E-118	SIRPA	0	ITGA6	2.1E-102
C1orf198	1.77E-155	WRB	4.98E-277	RPS26	0	CAPN2	1.6E-176	AUTS2	4.02E-223	FGD3	6.827E-95			SH3KBP1	1.977E-73	MARCH1	1.12E-244			ZFP36L2	4.87E-177	ARPC5	1.17E-117	GPRIN3	0	C16orf45	2.3E-102
ABI2	5.36E-155	CYP4X1	2.59E-276	MTRR	0	CYSTM1	2.09E-175	IMPA2	5.72E-223	RPL36A	9.041E-95			CD74	4.888E-73	LILRB5	1.27E-244			CREBRF	7.95E-177	CYTIP	1.75E-117	ARL6IP1	0	FAT4	1.38E-101
TNFSF10	7.07E-155	STOX1	7.9E-276	NUP58	0	CA12	2.85E-175	IL1R1	6.26E-223	POLR3GL	1.317E-94			NCKAP1L	1.191E-72	HMOX1	1.78E-244			SLU7	1.31E-176	HNRNPC	1.09E-116	SCCPDH	0	SYNE2	1.95E-101
ST14	7.47E-155	SRGAP3	3.72E-275	SNHG9	0	MEGF9	4.59E-175	TFCP2L1	8.31E-223	CD53	1.447E-94			PLP2	2.286E-72	ADAM8	2.59E-244			ZCCHC2	1.72E-176	ITGB8	1.73E-116	UQCRH	0	ARAP3	2.19E-101
DAG1	3.14E-154	MAK	1.22E-274	RPL27	0	AHCYL1	6.83E-175	DPP4	3.25E-221	FNBP4	1.936E-94			LTB	3.024E-72	LASP1	4.61E-244			IRAK3	2.76E-176	PPIA	2.22E-116	NDUFB2	0	GIPC2	3.84E-101
GSTP1	5E-154	CCP110	1.05E-272	CA12	0	RND1	2.49E-174	CREB3L1	3.3E-221	JAK1	2.181E-93			SHKBP1	3.306E-72	EIF5A	2.52E-243			SLFN11	3.82E-175	ZCCHC2	2.67E-116	APOC2	0	ITGB4	4.03E-101
ANKRD29	5.13E-154	DNPH1	3.64E-272	EBPL	0	FGFR2	1.3E-173	P3H2	3.43E-221	NMT2	2.971E-93			RILPL2	4.872E-72	FAM26F	1.21E-242			HCK	1.11E-174	OSBPL11	4.75E-116	TBC1D10C	0	GRB10	1.7E-100
TM7SF3	1.52E-153	MSI2	9.91E-272	B3GNT8	0	TIPARP	1.01E-172	USP13	6.48E-221	BTF3	1.623E-92			GSTK1	5.116E-72	ZFP36L2	3.87E-242			CYBA	1.62E-174	RAB31	6.72E-116	PEPD	0	C1R	3.3E-100

Cluster 1 (airway and alveolar epithelial cells)		Cluster 2 (ciliated cells)		Cluster 3 (type II alveolar cells-1)		Cluster 4 (type II alveolar cells-2)		Cluster 5 (type II alveolar cells-3)		Cluster 6 (immune cells)		Cluster 7 (plasma cells)		Cluster 8 (monocytes-1)		Cluster 9 (monocytes-2)		Cluster 10 (alveolar macrophages-1)		Cluster 11 (alveolar macrophages-2)		Cluster 12 (alveolar macrophages-3)		Cluster 13 (alveolar macrophages-4)		Cluster 14 (mesenchymal cells)	
Gene symbol	Adjusted p values	Gene symbol	Adjusted p values	Gene symbol	Adjusted p values	Gene symbol	Adjusted p values	Gene symbol	Adjusted p values	Gene symbol	Adjusted p values	Gene symbol	Adjusted p values	Gene symbol	Adjusted p values	Gene symbol	Adjusted p values	Gene symbol	Adjusted p values	Gene symbol	Adjusted p values	Gene symbol	Adjusted p values	Gene symbol	Adjusted p values	Gene symbol	Adjusted p values
NFIC	1.93E-153	DYX1C1	2.76E-271	DAPK2	0	KCNQ1OT	2.13E-172	CACNA2D	9.69E-221	HDAC1	2.232E-92			MTSS1	1.058E-71	TCIRG1	9.38E-240			SKAP2	3.54E-174	PDE4DIP	8.55E-116	SLC25A6	0	IFI16	4.03E-100
MSI2	1.8E-152	PCSK1N	3.1E-271	HAGLR	0	KANK1	3.1E-172	MPP7	2.72E-220	PIM2	2.635E-92			NCF2	1.089E-71	MREG	9.55E-239			PKP2	2E-173	PPIC	9.7E-116	ARPC1B	0	MYC	2.63E-99
NPNT	2.19E-152	IL5RA	4.97E-271	PTPRG	0	ARHGEF1	4.72E-172	PDZD8	7.79E-220	CYLD	2.853E-92			SEPT6	1.735E-71	PLEKHA2	2.47E-237			ATP10A	6.38E-173	ATP6V1E1	1.17E-115	ID2	0	SERPINH1	2.388E-98
NBEAL2	9.15E-152	RP11-368I7	2.03E-270	IFT57	0	RAB3IP	2.75E-171	FAM20A	1.31E-219	ARL6IP4	4.809E-92			JARID2	2.23E-71	PILRA	2.96E-237			HSP90AA1	7.05E-173	DPH3	2.01E-115	CNBP	0	STOM	3.948E-98
BAIAP2	1.34E-151	PKIG	2.38E-269	ARMT1	0	TUBB4B	6.35E-171	HAGLR	1.6E-219	DGKA	1.099E-91			MX2	4.853E-71	IFNGR2	4.83E-237			HCAR2	8.36E-173	AAK1	4.69E-115	PGLS	0	SERPINE2	5.922E-97
DDAH1	3.84E-151	DSP	7.85E-269	VKORC1L	0	SNHG9	5.39E-170	RPLP0	5.59E-219	ARHGAP3	1.398E-91			SEC14L1	5.849E-71	FCGRT	5.11E-236			PELI1	3.54E-172	TMEM38B	5.07E-114	RPL13A	0	TNFAIP1	1.167E-96
CNN2	6.44E-150	UNC119B	8.51E-268	RPL37	0	GADD45B	1.14E-169	PTPRF	5.65E-219	HSH2D	2.11E-91			CAPZA1	7.32E-71	TWF2	9.55E-236			LAIR1	4.34E-172	MAP1LC3	1.05E-113	SSB	0	FAM65A	1.449E-96
KIAA1324	6.68E-150	METRN	1.24E-267	FGFR2	0	CSRNP1	3.34E-169	AFF3	1.39E-218	RAN	6.356E-91			AGTRAP	7.969E-71	RAB10	1.46E-234			TUBA1B	1.37E-171	ADAMTSL	1.66E-113	SLC19A3	0	NEURL1B	1.804E-96
WWC1	6.99E-150	DNER	5.42E-267	FAM184A	0	PCYOX1	1.71E-168	ZNF281	4.58E-218	ABRACL	7.892E-91			LDHA	1.047E-70	MT-ND4	5.55E-234			ACTN1	1.72E-170	JAML	1.67E-113	EIF3G	0	RAB11A	2.391E-95
MXRA7	1.37E-148	CRISP2	1.27E-266	NR1D1	0	FDFT1	8.1E-168	WLS	6.85E-218	PSMB10	2.255E-90			TMEM176	1.906E-70	RAC2	1.28E-232			MCOLN1	8.2E-170	RETN	3.29E-113	LITAF	0	GJA1	4.731E-95
BIRC3	2.99E-148	CENPM	2.67E-263	LDHB	0	TMEM41A	1.03E-167	ADGRD1	1.96E-217	ATF6B	4.197E-90			KCNK6	2.261E-70	LRRC25	5.14E-232			HN1	1.77E-169	DENND4C	4.8E-113	EML4	0	TSC22D1	3.58E-94
ITPR3	6E-148	ENDOG	3.02E-263	ARL5A	0	FAM84B	1.32E-167	DSG2	2.06E-217	GPBP1	9.191E-90			PTPN6	2.774E-70	LAMP1	7.87E-231			FKBP15	4.25E-168	C20orf24	5.65E-113	PDK4	0	ITGA5	8.076E-94
LTBP4	1.56E-147	ERICH6-A	1.17E-262	GNB2L1	4.64E-307	WWC2	1.39E-167	ODC1	1.47E-216	LAPTM5	9.486E-90			CREB5	3.353E-70	PLXND1	7.89E-231			CYTIP	6.97E-168	RALBP1	1.58E-112	VAT1	0	CD151	1.034E-93
CTNNA1	1.91E-147	IQCA1	1.52E-262	EGR1	2.39E-303	BLVRB	3.61E-166	ACAT2	2.15E-216	RARRES3	1.571E-89			IER3	5.686E-70	ATP1B3	1.94E-230			PDLIM5	9.26E-168	APOBR	1.83E-112	NAA20	0	CD320	1.297E-93
DAPK2	9.69E-147	C2orf81	4.95E-262	SMDT1	3.59E-300	HACD2	4.4E-166	FAM129B	4.84E-216	RPS24	7.754E-89			PPT1	1.911E-69	SPPL2A	7.43E-230			DENND5A	6.03E-167	NDUFB1	3.96E-112	UBE2L3	0	TGFB1I1	4.297E-93
PEG10	1.25E-146	IFT88	1.52E-261	GLTSCR2	3.65E-298	WWC1	1.65E-165	NFAT5	1.57E-215	CLECL1	9.026E-89			UPP1	2.03E-69	CCL4	2.78E-229			SERPINA1	4.8E-166	IL1B	5.78E-112	TMEM219	0	BST2	7.764E-93
UBE2H	4.52E-146	SMIM5	4.82E-261	ASL	5.72E-298	TNFRSF12	1.2E-164	FOXA2	2.04E-214	ORAI2	9.185E-89			CXCR4	6.034E-69	GNA13	1.36E-228			PHACTR1	1.04E-165	COMT	6.43E-112	PFDN2	0	RPL3	2.595E-92
SLC40A1	6.1E-146	CHST6	5.4E-261	XAB2	6.6E-298	TFCP2L1	7.85E-163	CSF3R	2.49E-214	C9orf78	9.275E-89			FGD2	6.37E-69	YWHAH	1.77E-228			C1orf56	1.33E-165	TAX1BP1	7.59E-112	NDUFB7	0	SEC14L1	3.858E-92
RAI14	1.01E-145	P4HTM	5.6E-257	SIVA1	1.56E-296	MMAB	4.03E-162	CREBRF	1.16E-213	TNFRSF14	1E-88			ARF5	1.077E-68	HCLS1	4.82E-228			AKR1B1	4.1E-165	CAPZA2	1.01E-111	AP2M1	0	ZFP36	6.626E-92
GNG12	1.91E-145	IQUB	1.06E-256	TMEM256	6.39E-294	PRKCZ	5.1E-162	KCNJ8	1.76E-213	DENND2D	1.181E-88			REL	1.148E-68	TUBA1A	1.74E-227			STAC	3.4E-164	MIS18BP1	4.3E-111	COLEC12	0	PRCP	8.391E-92
TSPAN8	2.92E-145	ZNF440	2.69E-256	UQCR10	1.61E-292	FAM174B	5.38E-162	BMP1	2.57E-213	SRSF3	2.084E-88			PPP4C	1.585E-68	CST3	4.75E-227			UBE2L6	2.01E-163	C4orf48	8.27E-111	NDUFA1	0	A4GALT	1.878E-91
ST5	2.99E-145	PTPRN2	7.18E-256	FABP5	7.38E-291	MGLL	1.38E-161	FGFR2	3.12E-213	RPL22L1	2.214E-88			HLA-DQA	3.331E-68	CFD	1.45E-225			C1orf162	4.28E-163	DYNC1I2	9.84E-111	ASAH1	0	SHC1	2.833E-91
PARD6B	7.51E-145	RP11-395E	8.31E-256	ARPC1A	1.11E-289	SC5D	4.13E-161	ID4	4.15E-212	SRSF7	3.316E-88			VASP	9.399E-68	LINC00152	1.95E-225			B3GNT5	4.63E-162	PRR13	2.14E-110	SPOCD1	0	CSRNP1	4.162E-91
AUTS2	9.71E-145	IFT81	8.64E-254	SLC25A3	1.88E-289	GOLGA4	1.87E-160	NFKBIZ	4.9E-212	CAPZB	4.319E-88			RP11-386I	1.229E-67	SCARB2	1.12E-224			IER5	6.57E-162	RAB7A	2.97E-110	HCAR2	0	PIK3C2A	5.379E-91
NDFIP2	9.75E-144	RP11-1008	1.48E-253	PHPT1	2.48E-289	ADI1	2.26E-160	PAM	1.08E-211	DEF6	4.683E-88			VMP1	1.733E-67	MYO1F	1.18E-224			LITAF	6.22E-161	CASP1	5.78E-110	AKR1A1	0	RAPGEF5	9.951E-91
SLFN5	1.07E-143	CFAP58	7.42E-252	CIRBP	4.09E-287	CFLAR	7.71E-160	CA12	2.06E-211	HNRNPA3	5.9E-88			ATG3	8.908E-67	RBM3	3.92E-224			LACTB	1.41E-160	VASP	6.24E-110	LSAMP	0	FXYD6	1.626E-90
CBX6	1.39E-143	KCNN3	2.64E-251	DPM2	3.87E-281	CKB	2.16E-159	NEDD9	2.66E-211	PDCD4	7.485E-88			CCDC109	3.793E-66	COLGALT	6.92E-224			C4orf48	3.49E-160	CLEC7A	6.72E-110	TUBB6	0	STXBP6	1.817E-90
ATP13A4	1.83E-143	SYNE2	4.18E-251	IER2	1.41E-279	SLC27A3	9.9E-159	TMEM164	4.4E-211	RASGRP2	2.239E-87			PEA15	1.423E-65	HMGA1	1.04E-223			CD37	5.4E-159	VAMP8	8.26E-110	DNAJC5B	0	CNTNAP3	3.849E-90
CD2AP	4.23E-143	SPATA4	4.76E-251	ATP6V0E1	4.78E-279	CCDC14	2.35E-158	PARD6B	9.42E-210	AC090498.	2.7E-87			HERPUD1	2.936E-65	MYL6	4.39E-222			PYCARD	8.77E-159	CHMP5	9.47E-110	FAM162A	0	EID1	5.018E-90
MTUS1	1.09E-142	TSNAXIP1	2.09E-250	FADS1	7.14E-278	STAT3	3.69E-158	TMEM97	1.66E-209	HNRNPR	5.01E-87			SCIMP	4.755E-65	RHOQ	5.97E-222			ADTRP	1.87E-158	STX7	9.83E-110	FCGR1A	0	LAMC1	9.993E-90
MRPL14	1.29E-142	KIAA2012	4.73E-250	NDUFB11	1.1E-277	DHRS7	1.72E-157	TAPT1	3.14E-209	HNRNPA2	5.671E-87			OGFRL1	5.268E-65	SLC43A3	9.11E-222			CXCL3	9.07E-158	UBE2E2	1.12E-109	CD33	0	TNFRSF1A	2.551E-89
RP11-532F	1.9E-142	C16orf71	5.19E-250	MYLIP	1.7E-277	CHP1	2.08E-157	ESRRA	4.04E-209	VAMP2	9.143E-87			NABP1	1.578E-64	LPCAT2	2.64E-221			SLA	1.43E-157	PAK2	1.22E-109	ENPP4	0	EVA1C	8.311E-89
NCOR2	2.31E-142	COL21A1	1.12E-248	MRPS6	4.25E-277	RDH11	8.49E-157	DUOXA1	6.53E-209	SH3BGRL	2.63E-86			HACD4	1.888E-64	TFEC	1.02E-220			PDCL	1.59E-157	C9orf16	1.38E-108	OSTF1	0	MEIS1	2.672E-88
LPP	3.2E-142	ENAH	1.7E-248	ACAT2	3.76E-276	EHD2	4.42E-156	CGNL1	8.78E-209	STMN1	3.201E-86			HAVCR2	2.127E-64	IFI27L2	1.21E-220			TGM2	3.15E-157	LRP1	1.54E-108	C5AR1	0	SPTAN1	1.473E-87
ORMDL3	5.72E-142	PTPRF	1.73E-248	NDUFC1	1.22E-270	TCEAL8	5.45E-156	CNN3	1.78E-208	NUP210	5.251E-86			CPPED1	3.269E-64	RHOG	3.61E-220			EFHD2	1.83E-156	CDC42	7.63E-108	EPB41L4A	0	SWAP70	2.065E-87
COL1A1	3.55E-141	COLCA1	1.8E-248	MIEN1	3.18E-269	YBX3	8.02E-156	FGGY	3.57E-207	CKLF	5.509E-86			IL17RA	5.935E-64	IL10RB	1.5E-218			XAF1	3.69E-156	SLC19A3	7.82E-108	PLXDC2	0	PLPP3	2.15E-86
YPEL3	3.68E-141	HN1L	9.48E-248	PRDX6	3.58E-265	MACF1	8.63E-156	WWC1	1.46E-206	NKTR	9.351E-86			OSBPL8	7.283E-64	FKBP15	4.68E-217			MFSD1	5.7E-156	TDRD3	2.08E-107	HTATIP2	0	CNKSR3	4.248E-86
RERE	4.98E-141	CLDN3	1.41E-247	PTRHD1	3.65E-264	GEM	4.61E-155	LNX2	2.19E-206	RNPS1	1.105E-85			DOCK10	1.658E-63	CLIC1	7.3E-217			GYPC	1.21E-155	GABARAP	2.09E-107	RPL14	0	CHSY1	4.982E-86
CCDC186	1.7E-140	SERTAD4-	7.31E-247	STRA13	1.25E-263	POLR2C	1.39E-154	KCNQ1OT	1.33E-205	SPCS1	1.248E-85			COPE	3.159E-63	GLA	1.43E-216			ATP5L2	3.4E-155	ACTR2	2.47E-107	NUP214	0	PDLIM5	1.268E-85
SDC4	2.55E-140	ALDH1A1	4.88E-246	ALKBH7	2.35E-261	SPINK5	1.61E-152	SNHG9	1.83E-205	KMT2E	1.762E-85			LYN	3.175E-63	ATP6AP1	1.38E-215			LSAMP	2.07E-153	RWDD1	7.09E-107	CTSA	0	SNTB2	2.907E-85
NET1	2.89E-140	C21orf59	2.93E-245	ERP29	8.61E-260	RAB27B	8.45E-152	BMP2	5.11E-205	HCLS1	2.802E-85			PRKAG2	3.435E-63	QPCT	1.76E-214			TMEM38B	3.36E-153	ROCK1	1.67E-106	GAPLINC	0	FRMD6	3.892E-85
TPM1	9.47E-140	TRIP13	4.89E-245	OST4	4.53E-252	NFATC3	1.3E-149	EZR	1.82E-203	UBE2N	3.628E-85			SLC25A37	3.62E-63	GALNT1	2.05E-214			SELPLG	2.56E-152	SKAP2	2.55E-106	ANP32B	0	PBX1	7.483E-85
PARVA	6.1E-139	BCO2	3.24E-244	APRT	1.57E-247	F11R	1.37E-149	GUCY1A3	2.03E-203	ARHGAP9	5.114E-85			RPSA	5.568E-63	CXCL10	3.8E-214			DOK2	2.6E-152	PCBP1	6.98E-106	EGR2	0	GIMAP4	1.468E-84
TNFRSF21	9.75E-139	C6orf132	1.57E-243	NDUFA2	8.07E-246	EI24	5.28E-149	NECAB1	7.12E-202	RNF213	7.636E-85			RHOA	6.042E-63	GK	7.61E-214			CSGALNA	3.54E-152	COX17	1.02E-105	SQRDL	0	VIPR1	1.515E-84
F3	1.61E-138	CCDC80	4.27E-243	SEC61B	1.37E-230	MKNK2	3.24E-148	KRT8	1.6E-201	WAS	1.533E-84			VNN1	1.692E-62	TMEM176	9.53E-214			RP11-356N	9.18E-152	EPSTI1	1.79E-104	ZBTB8OS	0	AHNAK	2.019E-84
EI24	1.71E-138	SLC27A2	8.59E-242	NFKBIA	1.26E-218	CCNL1	3.48E-147	SPTBN1	3.99E-201	NUDT1	2.106E-84			TUBA1A	3.192E-62	AP1B1	1.1E-213			SECTM1	2.89E-151	BLOC1S2	6.52E-104	DOCK8	0	FYN	2.81E-84
TJP2	3.58E-138	MYCBPAP	5.55E-241	MRPL54	8.75E-218	KIF1C	3.68E-146	GOLGA2	9.08E-201	DGUOK	2.146E-84			ANKRD44	3.231E-62	SLC31A1	4.3E-213			ABCG1	4.09E-151	IRAK3	9.64E-104	EIF1	5.36E-277	PTPRG	3.848E-84
TSPAN12	6.89E-138	MPDZ	8.24E-241	ZDHHC3	5.88E-209	HSD17B12	2.84E-145	CMTM8	4.58E-199	CLNS1A	2.73E-84			IL16	4.195E-62	SMS	7.06E-213			FILIP1L	4.8E-150	MCTS1	1.84E-103	HSPA1B	5.88E-261	CYTL1	7.825E-84
BCR	1.2E-137	CYSTM1	5.62E-240	TXNDC17	6.81E-209	SQSTM1	4.07E-144	SLC25A23	2.05E-198	EIF4A2	1.007E-83			SKAP2	5.328E-62	DOCK10	7.76E-213			LAT2	6.5E-150	IFI16	2.58E-103	PPDPF	3.28E-237	AKT3	3.431E-83
ATXN2	1.2E-137	DZANK1	1.17E-239	ID3	1.73E-208	PTP4A2	9.41E-143	RRM2B	4.26E-198	PLCG2	1.493E-83			RHOC	6.184E-62	CALM1	1.85E-212			GNAI2	7.36E-150	PSMC1	2.64E-103	IGF1	1.14E-197	TMEM255	9.532E-83
NRP2	1.22E-137	KLHDC9	1.33E-239	SCGB3A2	7.71E-208	RFTN1	3.24E-142	FAM184A	1.36E-197	SNRPN	4.293E-83			JUNB	7.729E-62	LCP2	2.32E-212			CDC42EP3	1.66E-149	PSME1	3.13E-103	CXCL8	2.45E-116	AHNAK2	1.439E-82
NDST1	4.14E-137	CERKL	3.07E-239	CXCL2	2.31E-203	SAR1A	6.67E-141	LRP6	1.62E-197	ATP2A3	4.558E-83			GBP2	1.184E-61	RAB32	8.91E-212			SPRY1	7.61E-149	SPRY1	1.11E-102	IFI27	2.493E-34	SEPW1	2.301E-82
SERINC2	5.93E-137	NBEA	1.78E-238	MT-ND4L	1.65E-196	MYL12B	8.29E-140	INSR	6.78E-197	CREM	5.248E-83			C1orf54	2.253E-61	STAB1	1.47E-211			FMNL2	5.51E-148	HSPB1	1.89E-102			NEDD9	5.331E-82
TRIM56	1.15E-136	PCM1	3.96E-238	JUN	3.24E-195	CKS2	2.91E-139	ARHGAP3	8.32E-197	DRAP1	8.246E-83			TLN1	3.616E-61	METRNL	1.67E-210			VRK2	2.44E-147	LMNA	4.16E-102			CFD	1.384E-81
EIF4G1	6.3E-136	RP4-625H1	4.82E-238			CD55	4.63E-138	ESRP1	1.11E-196	PTTG1	1.411E-82			IKZF1	4.098E-61	GBA	1.5E-209			PTPN6	2.68E-147	MMD	5.92E-102			FLI1	2.181E-81
HEBP2	7.16E-136	VNN3	9.38E-238			STX3	2.8E-137	C9orf152	2E-196	APOBEC3	3.248E-82			UBE2D1	4.609E-61	EPB41L2	2.8E-209			CLEC7A	3.58E-147	ATP5L2	8.36E-102			ENTPD1	4.119E-81
LINC00657	1.52E-135	MYB	1.36E-237			PPP2CB	1.09E-136	ZDHHC9	9.99E-195	PIK3R1	4.395E-82			MSRB1	7.564E-61	PDXK	5.58E-209			PDK4	1.03E-146	APOL6	1.28E-101			LAMA5	1.26E-80
GPC4	2.45E-134	CLDN8	1.39E-237			ACSS2	3.69E-136	PIGA	3.82E-193	PRMT1	7.411E-82			RNF144B	3.197E-60	PHLDA1	1.27E-208			KMO	1.22E-146	SLC3A2	1.65E-101			FBLN5	2.797E-80
VEZT	3.23E-134	TRIM2	1.64E-237			GSPT1	6.11E-136	LSS	2.03E-192	PDE7A	9.131E-82			ATP6AP2	3.432E-60	PDIA6	1.61E-208			VASP	6.22E-146	CSGALNA	1.79E-101			ADARB1	3.155E-80
IFITM3	7.99E-134	C16orf46	4.26E-237			ODC1	1.94E-129	TMEM30B	4E-192	DDX39A	9.786E-82			SRSF2	5.553E-60	ZFYVE16	1.7E-208			STX11	1.56E-144	CD300LF	1.89E-101			MPDZ	4.676E-80
DGCR2	2.33E-133	ANKRD45	7.68E-237			KIDINS220	3.44E-128	GSTA4	2.26E-191	RASSF5	1.233E-81			ZFP36L2	7.389E-60	IFI6	3.29E-206			LCP1	1.3E-142	SVIL	2.9E-101			MCC	4.935E-80
STEAP4	1.64E-132	FAM161A	1.15E-236			RFC1	4.93E-128	PTPRK	3.3E-191	SMC3	1.326E-81			IL13RA1	1.029E-59	PARVB	3.38E-206			NCF4	1.54E-142	KIF16B	4.86E-101			SULF1	7.095E-80
ZMAT3	1.81E-132	HHLA2	1.03E-235			ENO1	1.26E-127	NDNF	8.12E-191	MYO1G	2.275E-81			CNTRL	1.473E-59	CD276	3.22E-205			RHOG	1.62E-142	ACTN1	7.42E-101			FOXO1	2.008E-78
COL4A4	2.98E-132	CCDC151	2.57E-235			HSPB1	2.19E-127	SHISA2	8.21E-190	UBXN1	2.29E-81			TCIRG1	2.152E-59	MYO5A	4.01E-205			CD53	3.05E-142	PSMA4	7.75E-101			PTMA	3.996E-78
PKP4	4.36E-132	CCDC96	3.29E-235			NAA38	5.71E-125	VEPH1	8.95E-190	TSPYL2	6.814E-81			DAPP1	2.343E-59	CALU	6.87E-205			ZYX	8.58E-141	ALDH1A1	3.95E-100			LIMA1	4.386E-78
AHR	6.48E-132	RP11-60L3	9.44E-235			GADD45G	1.49E-123	AQP4	1.83E-188	TNRC6B	6.828E-81			PLAUR	7.358E-59	ANPEP	8.19E-205			ACOT2	1.76E-139	AXL	8.29E-100			FLNB	5.606E-78
NFAT5	1.52E-131	BICC1	2.44E-233			CTNNA1	2.75E-123	TEAD1	6.78E-188	CH17-189	9.092E-81			OSTF1	8.385E-59	ARF6	1.55E-204			NT5C3A	5.23E-139	ENPP4	9.51E-100			SH3BP5	3.202E-77
RAB5B	1.54E-131	CST6	2.89E-233			IRF2BP2	1.76E-121	SLC12A2	1.3E-187	SYNE2	1.847E-80			MIS18BP1	1.627E-58	ARHGAP3	2.07E-204			TOMM40	5.3E-139	PPT1	9.83E-100			MIDN	6.256E-77
SLC44A2	1.83E-131	GSTP1	4.01E-232			SEC61G	2.01E-121	LIPH	7.82E-187	PLEKHJ1	2.215E-80			C14orf2	2.033E-58	LYN	3.65E-204			ISG15	3.61E-137	MDM2	1.38E-99			PRMT1	7.969E-77
PIGR	1.97E-131	GIPR	9.46E-232			PNKD	6.67E-121	MYO5C	1.23E-183	IRF8	3.762E-80			RP11-347P	3.69E-58	CYP27A1	3.67E-204			CD300LF	3.75E-137	GNAS	2.15E-99			RCN1	1.06E-76
ARHGAP5	2.92E-131	KIAA1841	3.93E-231			ACTN4	5.65E-114	CD38	1.12E-182	TERF2IP	3.851E-80			AREG	1.096E-57	HSP90B1	5.81E-204			DEFB1	4.75E-137	EIF2AK2	1.178E-98			LMO2	1.526E-76
CLDN3	7.07E-131	TRIM29	4.89E-231			BCYRN1	3.36E-99	FAM84B	3.49E-182	DCK	1.599E-79			IRAK3	1.493E-57	SLCO2B1	1.03E-203			UBXN2B	5.88E-136	RHEB	1.315E-98			SERPINE1	1.807E-76
UGCG	8.69E-131	ABI2	1.2E-230			PNRC1	6.249E-85	PLPP3	4.59E-182	IRF3	2.7E-79			LPCAT2	2.828E-57	TUBB6	4.38E-203			SMNDC1	7.59E-136	ZNF706	1.931E-98			SHROOM4	3.142E-76
CRB3	5.28E-130	ATP2A2	5.38E-230			NNMT	1.016E-82	LMO7	1.5E-181	MGEA5	5.392E-79			EPB41L2	3.014E-57	BID	5.65E-203			CLIP4	1.47E-135	PTPN6	2.105E-98			TMEM88	7.046E-76
IER3	1.55E-129	LRRC43	5.65E-230			PHLDA2	3.831E-79	OSMR	3.05E-179	CCDC12	7.035E-79			CLNS1A	5.381E-57	PTPRE	8.04E-203			FN1	1.5E-135	STX11	2.105E-98			ZBTB16	8.038E-76
WEE1	2.94E-129	C20orf96	5.8E-230					NEBL	3.19E-179	SARAF	1.184E-78			USP15	8.717E-57	VCAN	1.08E-202			CASP1	2.63E-135	CFDP1	2.492E-98			ABLIM1	1.801E-75
HDGF	3.57E-129	PLEKHG7	1.12E-229					ERBB3	4.88E-179	NCL	1.634E-78			METRNL	1.074E-56	MT-ND5	2.89E-202			SLC16A10	2.36E-134	DECR1	2.609E-97			MGST2	2.034E-75
IL6ST	5.99E-129	MLLT4	1.21E-229					HSD17B6	1.02E-177	CALM3	3.209E-78			EIF3M	1.196E-56	PABPC4	2.15E-201			AXL	1.72E-131	CLIP4	3.107E-97			EXOC6	2.604E-75

Cluster 1 (airway and alveolar epithelial cells)		Cluster 2 (ciliated cells)		Cluster 3 (type II alveolar cells-1)		Cluster 4 (type II alveolar cells-2)		Cluster 5 (type II alveolar cells-3)		Cluster 6 (immune cells)		Cluster 7 (plasma cells)		Cluster 8 (monocytes-1)		Cluster 9 (monocytes-2)		Cluster 10 (alveolar macrophages-1)		Cluster 11 (alveolar macrophages-2)		Cluster 12 (alveolar macrophages-3)		Cluster 13 (alveolar macrophages-4)		Cluster 14 (mesenchymal cells)	
Gene symbol	Adjusted p values	Gene symbol	Adjusted p values	Gene symbol	Adjusted p values	Gene symbol	Adjusted p values	Gene symbol	Adjusted p values	Gene symbol	Adjusted p values	Gene symbol	Adjusted p values	Gene symbol	Adjusted p values	Gene symbol	Adjusted p values	Gene symbol	Adjusted p values	Gene symbol	Adjusted p values	Gene symbol	Adjusted p values	Gene symbol	Adjusted p values	Gene symbol	Adjusted p values
TSTD1	6.91E-128	CCDC88C	2.59E-229					EDNRB	1.05E-177	CNTRL	3.806E-78			C20orf27	1.933E-56	KCNMA1	2.7E-201			MIR3945H	1.41E-129	SMCO4	4.861E-97			PGM5	4.076E-75
TAGLN2	8.81E-128	MARCKS	5.38E-229					DHCR7	2.96E-177	PPP1R2	6.286E-78			BTK	2.538E-56	OSTF1	1.89E-200			TFRC	1.47E-129	BAX	3.043E-96			WBP5	1.732E-74
CAPN1	2.25E-126	CROCC	9.19E-229					PRTG	1.53E-176	ACTR3	1.241E-77			ARL6IP4	2.938E-56	CLTC	2.63E-200			TBXAS1	4.27E-129	IFNGR1	3.608E-96			KLF4	2.39E-74
RASSF7	3.26E-126	LRRC49	1.91E-228					NNMT	1.97E-175	TUBB	1.312E-77			PSME1	1.395E-55	MMP19	1.9E-199			RHOB	4.18E-128	SLC15A3	4.584E-96			KALRN	4.081E-74
ZDHHC21	9.8E-126	RSPH14	1.14E-227					TSPAN13	3.25E-174	TPR	1.477E-77			MIR4435-2	2.494E-55	SQRDL	6.21E-199			ARRB2	8.7E-128	AIMP1	1.294E-95			ADGRG1	5.483E-74
ANKRD50	1.8E-125	INHBB	3.52E-227					GEM	2.01E-173	GPR183	8.058E-77			MSN	3.807E-55	CRIP1	9.9E-199			DDHD1	1.34E-127	IFIT1	2.136E-95			CDKN1A	5.899E-74
DYNC1H1	2.52E-125	DRC7	7.65E-227					IFT57	7.33E-172	FAM215B	1.037E-76			LAP3	4.011E-55	CLEC2B	1.04E-198			SNTB1	8.41E-127	NDUFA1	2.266E-95			MCF2L	8.207E-74
STARD10	1.12E-124	MAPRE3	4.56E-226					SAMD5	1.08E-171	MFNG	3.988E-76			SAP30	4.603E-55	MYO9B	4.96E-198			ITGB8	2.16E-126	THBD	4.045E-95			HDAC7	1.555E-73
PBX2	1.33E-124	INTU	5.92E-226					SOCS3	1.09E-171	GIMAP1	4.969E-76			EIF3F	6.377E-55	UPP1	8.93E-196			GAS2L3	2.67E-126	AGPS	5.105E-95			DOCK6	2.173E-73
MAST4	3.19E-124	SEPW1	8.46E-226					SLC7A2	2.83E-171	EIF5A	7.778E-76			HNRNPM	1.06E-54	ARL6IP5	4.45E-195			C5AR1	5.01E-126	POLD4	5.776E-95			ACTN1	4.903E-72
BPIFB1	1.07E-123	DUSP18	8.79E-225					MAGI3	6.4E-171	RBM3	1.408E-75			SUPT4H1	2.006E-54	APBB1IP	6.45E-195			EREG	4.42E-124	TUBB	6.162E-95			CFH	5.45E-72
GOLGA2	1.24E-123	ARX	1.55E-224					FADS1	5.38E-170	C19orf25	1.729E-75			ID2	3.958E-54	PRMT2	1.55E-194			HEATR5B	4.44E-123	PFDN1	1.948E-94			AOC3	9.417E-72
P4HB	1.65E-123	SHROOM3	9.5E-223					PLS3	4.06E-169	HINT1	2.436E-75			HLA-DQB1	5.547E-54	PLEKHB2	1.31E-193			EGR2	1.8E-122	TMEM261	2.276E-94			RP11-382A	1.072E-71
ARL4C	2.22E-123	SLC22A4	1.24E-221					PDP2	4.07E-168	KIF20B	4.853E-75			IER2	6.644E-54	CSK	1.34E-193			DNASE2B	6.26E-122	PSME4	5.842E-94			S100A13	1.172E-71
USP22	2.34E-122	ARHGAP3	2.76E-221					TXLNG	5.53E-167	RORA	1.639E-74			HLA-DQB2	7.804E-54	RGCC	1.46E-193			DUSP1	1.02E-120	ARHGAP1	6.136E-94			PIK3C2B	1.247E-71
DYNLL2	7.15E-122	ICA1L	2.39E-220					SLC6A20	1.58E-166	NAP1L4	1.725E-74			TRMT1	1.271E-53	DOCK2	1.79E-192			LCP2	5.46E-120	NT5C3A	9.948E-94			MFGE8	1.268E-71
ACSL5	1.47E-121	BCAS1	3.17E-219					FOLR1	6.47E-165	RBM39	4.583E-74			IDH2	1.423E-53	CAMK1	3.14E-191			LY86	2.74E-119	MTIF3	1.154E-93			IER3	2.962E-71
EVA1A	1.68E-121	C22orf23	8.44E-219					EPHA2	1.58E-164	CARHSP1	5.38E-74			DAZAP2	1.533E-53	ABHD12	9.62E-191			IFIT3	4.14E-119	POR	3.093E-93			MARCKS	5.576E-71
C16orf89	1.75E-121	ST6GALN	1.49E-218					AK1	4.73E-163	ANKRD36	1.02E-73			TSC22D3	1.55E-53	SLC15A3	1.28E-190			TLR4	7.02E-119	SIDT2	3.482E-93			GIMAP1	7.426E-71
STK17A	1.84E-121	TCTN2	2.9E-218					S100A14	1.21E-162	ILF3	1.496E-73			EPSTI1	3.132E-53	ADGRE5	3.13E-190			CAPG	1.79E-118	LMO4	5.621E-93			LBH	2.127E-70
PPP2CB	8.43E-121	LRRC73	9.31E-218					EFNA1	2.13E-162	MT-ND4L	1.898E-73			CHD1	3.176E-53	PTMA	1.47E-189			CLEC2B	2.26E-118	MRPL1	1.187E-92			SRP14	2.221E-70
RHOC	2.27E-120	CYP4B1	2.34E-217					NPNT	4.06E-162	IL10RA	3.718E-73			XRN2	3.874E-53	RHOC	3.14E-189			ARHGAP1	3.24E-118	CKLF	1.46E-92			GADD45B	7.198E-70
CTDSP2	6.1E-120	DENND6B	7.34E-217					SIK1	1.47E-161	GYPC	5.149E-73			MTHFD2	8.673E-53	CEBPB	3.93E-189			IFI16	3.91E-118	HBEGF	1.938E-92			RALGAPA	2.09E-69
LRP10	9.57E-120	DEGS2	1.55E-216					NHSL2	3.19E-161	H3F3B	5.919E-73			RB1	1.163E-52	GIMAP4	4.89E-189			FPR1	2.35E-117	PLEKHA2	1.978E-92			PHLDB2	3.429E-69
WWTR1	4.71E-119	PIP	2.15E-215					RNF128	7.75E-161	HMGA1	7.127E-73			APRT	1.297E-52	TAP1	6.06E-189			PTGER4	2.6E-117	NSRP1	4.517E-92			MEF2C	4.483E-69
C5orf38	5.48E-119	WFDC2	9.6E-215					EXPH5	4.73E-159	CHMP7	9.919E-73			LAMTOR2	1.368E-52	MSN	8.06E-189			ZRSR2	1.11E-116	SLC16A6	1.765E-91			PTTG1IP	1.845E-68
CFLAR	2.7E-118	CCDC148	1.19E-214					CASD1	1.91E-158	IRF1	1.443E-72			ROCK1	1.441E-52	TNFAIP2	9.08E-189			CECR1	1.48E-116	TMBIM1	1.794E-91			INHBB	2.247E-68
WBP5	3.49E-118	CRIP2	4.76E-214					WWC2	7.6E-158	C19orf66	1.665E-72			TGFB1	1.615E-52	ATP6V0D1	1.14E-188			EMP3	3.63E-116	CYBB	2.878E-91			ZFYVE21	3.06E-68
ASPH	9.32E-118	GSTA2	5.2E-214					SUSD2	2.27E-157	EPC1	2.38E-72			PICALM	1.938E-52	LDHA	2.95E-188			CYBB	1.05E-115	SP100	3.17E-91			NKTR	4.737E-68
ARAP2	7.58E-117	SPATA6	1.58E-212					ESAM	4.05E-157	N4BP2L2	4.647E-72			RBPJ	4.008E-52	MYADM	7.2E-188			AKR1C3	1.52E-115	NCOR1	3.229E-91			APOL3	5.235E-68
ATP2A2	1.03E-116	PRMT8	3.96E-212					MAP9	1.2E-156	RP11-386I	6.711E-72			HLA-DRB5	8.007E-52	PSME2	9.5E-188			LY6E	7.75E-115	STX12	3.371E-91			EPB41L2	1.15E-67
WDR6	1.06E-116	HSPH1	9.09E-211					SLC40A1	1.57E-156	DNAJC9	6.952E-72			NUDT1	2.038E-51	ITGB1BP1	1.17E-187			SEMA3C	1.07E-114	MCL1	1.417E-90			ACTG1	1.447E-67
CDKN2B	2.1E-116	DZIP1L	7.77E-210					TOX	9.92E-155	HMGN2	1.31E-71			LAMTOR1	2.045E-51	P2RX7	2.05E-187			PTPRC	1.4E-113	IRF8	3.984E-90			PLS3	2.357E-67
CCDC6	1.12E-115	KIF6	1.13E-209					SCGB3A1	3.81E-154	LUC7L3	1.326E-71			SLC43A2	3.313E-51	RAPGEF1	3.03E-187			VMO1	3.46E-112	CHCHD10	1.172E-89			MAP3K6	3.159E-67
VPS13A	1.29E-114	SERPINI2	1.55E-209					RGS16	9E-154	ITGAE	1.995E-71			SYK	5.133E-51	PSMC1	3.75E-187			RNF130	5.35E-111	RBPJ	1.409E-89			PHACTR2	3.394E-67
KIF1C	1.47E-114	TTC16	9.02E-209					PLEKHA5	3.25E-152	TSC22D4	2.531E-71			TMEM167	5.273E-51	UBE2D1	6.31E-187			OAS1	7.71E-110	ZSCAN16-	2.845E-89			MXRA7	4.601E-67
KCNQ1OT	1.5E-114	ARMC2	4.18E-208					SHROOM3	5.6E-152	PFDN5	3.12E-71			C7orf73	5.679E-51	CD36	5.31E-186			GNA13	3.72E-108	IER5	4.961E-88			MYO1C	4.884E-67
LTBP3	2.31E-114	TTLL7	5.65E-208					NEDD4L	3.27E-149	ZCCHC11	3.98E-71			HIGD2A	8.924E-51	SNX6	1.64E-185			CRIP1	4.36E-107	RHOG	7.908E-88			SEMA6B	5.246E-67
MACF1	7.37E-114	HS3ST1	9.87E-208					TMC5	3.34E-149	MEAF6	5.479E-71			COMMD6	1.278E-50	TPI1	7E-185			CLEC4E	3.73E-106	VRK2	3.196E-87			LRRC1	6.476E-67
IFITM2	1.55E-113	AC009502.	1.63E-207					F3	1.01E-148	ERAP2	1.184E-70			PIM1	1.282E-50	YWHAG	1.11E-184			CRTAM	9.98E-105	MFGE8	4.346E-87			CX3CL1	7.225E-67
CD47	5.7E-113	LINC01091	1.69E-207					AGR2	1.4E-147	FKBP11	1.459E-70			CYTIP	4.173E-49	PAK2	1.39E-184			KYNU	2.03E-104	TMED5	9.477E-87			SCARF1	1.291E-66
PPA1	6.68E-113	RHPN1	2.53E-207					CLDN12	1.77E-146	PHF14	1.804E-70			EIF3L	6.424E-49	ATP6V1C1	4.91E-184			THBD	1.58E-103	ZYX	5.671E-86			GADD45A	1.815E-66
AP2B1	1.26E-112	WBSCR27	4.67E-207					CCND2	3.43E-146	APBB1IP	5.103E-70			MEF2A	1.005E-48	SHTN1	1.01E-183			PECAM1	2.14E-102	PLEKHB2	1.376E-85			DOCK4	2.777E-66
LY6E	1.83E-112	C5orf42	1.61E-205					VEGFA	1.17E-141	FYB	6.956E-70			RBP7	1.935E-48	RNASET2	1.63E-183			HSPA1A	6.47E-102	GPI	1.543E-85			USP53	4.38E-66
AHCYL2	2.27E-112	CALM2	3.23E-205					NFIB	2.19E-140	BCL11A	1.245E-69			MGAT1	2.776E-48	DOCK4	2.25E-182			KLHL6	2.12E-101	NUP214	1.651E-85			NDRG2	4.901E-66
FAM63B	6.73E-112	RP11-60L3	1.09E-204					MAGI1	4.4E-139	CYTH4	1.41E-69			CMTM7	1.151E-47	DRAP1	1.01E-181			SAMSN1	7.516E-94	SMS	4.8E-85			FHL1	1.431E-65
STT3B	7.28E-112	CH17-340N	1.14E-204					GDF15	2.58E-135	RNASET2	1.572E-69			PLSCR1	1.329E-47	SNX3	3E-181			HERC5	6.776E-93	IFIT2	5.151E-85			ANGPT2	1.508E-65
TUG1	9.9E-112	FAM65B	1.19E-204					LIFR	1.84E-134	NAP1L1	2.14E-69			FIBP	1.464E-47	EHBP1L1	3.72E-181			MS4A4A	9.72E-93	SFT2D1	8.479E-85			TRIB2	1.584E-65
ERRFI1	1.12E-111	SIX4	1.02E-203					GKN2	6.41E-133	METTL12	7.955E-68			EIF5A	2.102E-47	DNMT1	5.93E-181			IFI44L	1.198E-85	GCA	9.522E-85			SERPINB6	3.28E-65
TRIP6	2.47E-111	PGM2L1	1.48E-203					ABLIM1	6.05E-130	QRSL1	1.517E-67			PGK1	2.143E-47	SYK	8.33E-180			GPR65	2.116E-82	BST2	1.247E-84			FKBP9	4.778E-65
EPS8	3.06E-111	PDLIM4	1.83E-203					ENAH	2.95E-129	PDE3B	4.918E-67			PDE4B	2.659E-47	SH2B3	8.69E-180			GMFG	1.393E-76	DOCK8	4.043E-84			CDC37	5.724E-65
TSPAN3	3.62E-111	HSPBP1	1.31E-202					SYNE2	3.18E-129	RPS13	5.912E-67			FCGR3A	6.404E-47	GLMP	1.31E-179			OTUD1	5.535E-75	UBE2R2	1.029E-83			ARHGEF1	3.485E-64
SLC22A31	5.18E-111	CEP290	2.81E-202					NGFRAP1	3.41E-128	BTN3A2	6.314E-67			AIM1	9.51E-47	RAB1A	1.59E-179			FCGR3A	1.328E-69	BTF3L4	1.159E-83			JAG1	5.048E-64
PRSS23	2.13E-110	RP11-395B	3.33E-202					CLDN4	5.23E-128	MKI67	1.216E-66			MYCBP2	3.267E-46	ABHD2	3.33E-179			LIPA	8.321E-68	NEXN	2.068E-83			STARD3	1.227E-63
GOLGB1	8.37E-110	FAM47E	4.1E-202					KRT18	9.33E-128	RASGRP1	1.715E-66			CD99	4.556E-46	SLC20A1	6.5E-179			MARCH1	1.481E-65	ADGRE5	5.444E-83			FGD5	3.511E-63
TRIM8	8.44E-110	CTGF	9.91E-202					SORBS2	1.86E-125	ATF7IP	2.225E-66			CLEC2B	7.103E-46	TAPBP	6.9E-179			JAML	3.186E-65	PDK4	5.572E-83			GNG12	4.766E-63
BCL2L1	1.09E-109	CCDC24	1.51E-201					SFN	2.56E-122	NASP	2.75E-66			SRSF3	7.492E-46	TMEM176	3.47E-176			KCTD12	1.273E-61	ARPC1B	1.157E-82			PTPRM	5.175E-63
TPM4	1.53E-109	SMKR1	2.56E-201					RNASE4	1.39E-121	ANAPC16	3.128E-66			IDH3G	8.777E-46	ZNF706	9.58E-176			PLAUR	3.725E-56	DDHD1	2.482E-82			RDX	5.429E-63
SPTAN1	2.2E-109	IFT46	4.44E-201					HES1	3.3E-116	PSME2	4.254E-66			NAPRT	1.473E-45	NINJ1	1.36E-175			HLA-DMB	3.101E-52	BNIP2	3.325E-82			SPNS2	1.729E-62
SEL1L3	2.54E-109	CLMN	5.95E-201					SFTA1P	3.47E-113	HIST2H2B	5.245E-66			CELF2	1.606E-45	FNBP1	3.01E-175			MNDA	1.604E-49	LACTB	4.388E-82			RAMP1	1.856E-62
ANXA2	6.42E-109	RP11-446N	8.26E-201					AKR1C1	8.16E-112	POLR2J3	5.967E-66			BRK1	1.672E-45	RAB5C	1.72E-174			IGSF6	1.666E-47	CLEC2B	1.762E-81			CCDC68	2.587E-62
SNHG19	1.12E-108	CLDN4	9.14E-201					CCND1	2.71E-110	PIP4K2A	7.791E-66			PARL	1.735E-45	PSMB9	3.18E-174			RBP4	1.496E-46	PHACTR1	1.093E-80			SUN2	2.643E-62
SCAI	5.89E-108	ZNF273	1.18E-200					AQP1	1.19E-108	TOMM7	1.032E-65			CALM2	3.303E-45	PRNP	2.51E-173			RETN	3.536E-44	FCF1	5.917E-80			LPP	3.054E-62
MPRIP	1.57E-107	HSPB11	3.1E-200					COL8A1	2.38E-107	ARHGAP2	1.861E-65			FUS	3.377E-45	SLC2A3	1.53E-172			XIST	2.758E-43	IFI27	1.419E-79			MAPK3	4.154E-62
SAA1	1.6E-107	C1orf141	4.23E-200					SMIM22	3.76E-101	C5orf56	6.297E-65			MTDH	8.347E-45	OSBPL8	2.29E-171			LST1	4.827E-36	RGS19	1.117E-78			SLC39A14	4.984E-62
CLPTM1L	2.34E-107	CYB561	9.31E-199					BCYRN1	1.483E-91	SFXN1	1.498E-64			SAT1	1.044E-44	TALDO1	4.98E-171					FDX1	1.119E-78			CDC42BP	7.1E-62
AK3	3.36E-107	UBAC1	1.43E-198					GJA1	1.089E-85	ANP32B	1.499E-64			GADD45B	1.595E-44	CD40	2.56E-170					PNPLA6	2.22E-78			TTC28	8.772E-62
WWC2	7.7E-107	RBM38	1.02E-197					ID1	3.604E-85	CSK	4.821E-64			HNRNPA2	2.559E-44	PGD	3.71E-170					CMIP	2.627E-78			GAS6	9.606E-62
ALCAM	2.83E-106	AZIN1	1.69E-197					CYP4B1	1.757E-82	FAM107B	9.084E-64			BST1	1.111E-43	CXXC5	9.7E-170					GAA	2.629E-77			KANK2	2.48E-61
SPINK5	3.83E-106	WDR90	1.94E-197					MT1E	2.024E-79	ARHGEF6	3.173E-63			PTEN	3.219E-43	ITGB1	1.26E-169					RTN3	1.017E-76			SLC38A2	4.246E-61
PLLP	6.59E-106	IGF2BP2	2.01E-197					GPRC5A	7.242E-78	SLA	5.699E-63			ENO1	3.497E-43	BCKDK	1.71E-169					RAB13	1.127E-76			ZCCHC24	4.756E-61
MGAT4B	9.35E-106	TP53INP1	2.72E-197					ELF3	1.819E-72	RASA2	7.944E-63			FPR3	4.675E-43	TDP2	6.37E-169					KYNU	1.903E-76			BCAM	5.145E-61
MYOF	9.99E-106	USP2	1.21E-196					PDLIM3	5.51E-72	GAPT	3.876E-62			TAP1	1.638E-42	PSMD14	7.75E-169					NCKAP1L	6.141E-76			SOCS2	5.694E-61
TMOD3	1.33E-105	CEP83	6.32E-196					SCGB1A1	2.716E-70	CYFIP2	6.532E-62			ABRACL	3.876E-41	TAGLN2	3E-168					EFHD2	1.045E-75			ABLIM3	8.565E-61
EPB41L5	2.35E-105	CCDC67	6.46E-196					WFDC2	1.115E-67	IDH2	8.852E-62			EIF3G	3.893E-41	IDH1	1.21E-167					CD58	2.864E-75			PDLIM7	9.879E-61
FAM210B	6.69E-105	ENKD1	7.58E-196							VOPP1	9.335E-62			WARS	4.224E-41	CD58	2.75E-167					ARRB2	3.301E-75			NQO1	1.195E-60
MKNK2	2.24E-104	EFCAB2	9.86E-196							LAT2	9.336E-62			TRAPPC1	4.549E-41	QKI	3.72E-167					ATP6V1B2	6.36E-75			ACTN4	1.788E-60
CLDN7	4.85E-104	TACSTD2	2.06E-195							CHST12	2.745E-61			ARL6IP5	5.076E-41	VOPP1	5.08E-167					TBXAS1	1.95E-74			PRKCH	2.674E-60

Cluster 1 (airway and alveolar epithelial cells)		Cluster 2 (ciliated cells)		Cluster 3 (type II alveolar cells-1)		Cluster 4 (type II alveolar cells-2)		Cluster 5 (type II alveolar cells-3)	
Gene symbol	Adjusted p values	Gene symbol	Adjusted p values	Gene symbol	Adjusted p values	Gene symbol	Adjusted p values	Gene symbol	Adjusted p values
VPS13C	6.69E-104	SEC14L4	3.2E-194						
CLTC	8.79E-104	IQCE	8.56E-194						
LPCAT1	1.09E-103	CCDC157	1.28E-193						
HMGN3	2.08E-103	CYB5D1	1.43E-193						
SEPT2	2.38E-103	CKB	8.96E-193						
ATP11A	2.99E-103	NME7	2.21E-192						
CCDC84	4.65E-103	PLXNB1	7.97E-192						
SPINK1	6.82E-103	IFT43	7.98E-192						
DMBT1	9.91E-103	USP43	2.1E-191						
PRRC2B	1.02E-102	DPY19L2	2.63E-191						
PRDX2	1.08E-102	CFAP61	9.21E-191						
C12orf49	1.7E-102	ENO4	1.14E-190						
IL33	2.05E-102	DGKH	3.13E-190						
TOR1AIP2	4.88E-102	UCKL1-AS	4.05E-190						
FOSL2	1.23E-101	NRAV	1.55E-189						
SAA2	6.55E-101	C15orf65	1.64E-189						
AKR1C1	7.09E-101	MEIG1	1.94E-189						
LDLR	1.17E-100	NEK1	1.24E-188						
ITGAV	1.73E-100	DNAH1	1.92E-188						
ZNF431	3.02E-100	PCDH17	8.89E-188						
MGLL	4.86E-100	RSPH3	1.75E-187						
NFE2L2	1.14E-99	PTGES3	3.21E-186						
MIDN	2.4E-99	C11orf74	1.11E-185						
LIMD1	2.59E-99	RP5-1092A	2.03E-185						
WSB1	3.55E-99	IL7	2.64E-185						
NEXN	7.57E-99	EFCAB6	4.57E-185						
MUC5B	1.067E-98	PZP	5.46E-185						
TMEM43	6.735E-98	MYCBP	9.9E-185						
BAG1	2.519E-97	CTA-363E(	1.63E-184						
DUOXA1	2.695E-96	FAM179B	2.39E-184						
ADD1	5.569E-96	ALS2CR12	4.06E-184						
COL4A1	2.966E-95	CELSR1	7.74E-184						
PHACTR2	3.621E-95	RPGR	8.61E-184						
FAM107B	5.259E-95	CCNA1	9.37E-184						
GLCCI1	6.663E-95	DYNC2LI1	1.03E-183						
TIA1	4.287E-94	SYNGAP1	2.28E-183						
CRK	1.634E-93	PRR15	6.56E-183						
PDXDC1	4.103E-93	NTN1	1.08E-182						
MGP	4.398E-93	DALRD3	2.79E-182						
APLP2	2.05E-92	ANKFN1	6.17E-182						
IRF2BPL	1.696E-91	CCDC13	1.06E-181						
SEC61A1	1.78E-91	DMD	1.11E-181						
SLC39A8	4.435E-91	TTC30B	2.81E-180						
UBL3	1.629E-89	TSTD1	8.52E-180						
HDLBP	1.709E-89	SMIM6	4.55E-179						
HSBP1L1	1.801E-89	MORN1	6.09E-179						
CYBRD1	3.137E-89	LRRC56	2.11E-178						
STARD7	4.588E-89	NR2F1	3.65E-178						
DENND3	1.528E-88	GNA14	4.11E-178						
PARP14	1.137E-87	FBXO36	9.94E-178						
FADS3	4.278E-87	ZNF487	1.3E-177						
DHRS3	1.392E-86	LEKR1	1.97E-177						
S100A6	2.959E-86	HES1	3.83E-177						
METTL7A	3.664E-86	ACKR4	3.9E-177						
ANKRD11	5.11E-86	MORF4L2	4.11E-177						
H1F0	8.747E-86	PPP1R36	1.23E-176						
MT1X	1.454E-84	AKAP6	2.98E-176						
DDX17	1.897E-83	ATP9A	3.44E-176						
CLTB	2.717E-83	CEP41	3.47E-176						
LIMA1	2.782E-83	FOXA1	2.35E-175						
PHLDA2	5.673E-83	SERTAD4	5.35E-175						
GPM6A	1.241E-82	FUZ	7.04E-175						
ANKRD36(	2.138E-82	DPY30	1.27E-174						
FERMT2	2.776E-82	ANKMY1	2.68E-174						
STOM	7.718E-82	MT-ND3	3.15E-174						
RRAS	1.36E-81	LRP11	5.2E-174						
CD46	3.816E-81	FAM83H-A	6.08E-174						
CISD1	1.57E-80	ZFHX2	7.74E-173						
FLNA	1.754E-80	PKIB	3.1E-172						
MACC1	4.464E-80	C6orf52	1.19E-171						
SNHG25	5.114E-80	BMPR1B	1.32E-171						
MT-CO3	7.096E-80	RHPN2	1.97E-171						
DLC1	2.909E-79	ZNF474	1.36E-170						
ACTG1	3.369E-79	GAS8	1.92E-170						
GDF15	9.08E-79	SORBS2	3.25E-170						
QSOX1	9.945E-79	IRX3	7.19E-170						
PTTG1IP	3.253E-78	GTF2I	7.15E-169						
MT-ND6	3.466E-77	CTD-2270I	1.58E-167						

Cluster 6 (immune cells)		Cluster 7 (plasma cells)		Cluster 8 (monocytes-1)		Cluster 9 (monocytes-2)	
Gene symbol	Adjusted p values	Gene symbol	Adjusted p values	Gene symbol	Adjusted p values	Gene symbol	Adjusted p values
REL	9.32E-61			NMI	1.359E-40	IQGAP1	5.29E-167
ARID5A	1.162E-60			GBP1	2.671E-40	MTDH	6.89E-167
PTPN22	1.173E-60			BLOC1S1	2.999E-40	DBNL	9.37E-167
GPR65	1.52E-60			HADHA	3.156E-40	ABI1	6.22E-166
KIAA0101	1.557E-60			LITAF	4.378E-40	EPSTI1	6.42E-166
ARL4C	1.721E-60			CLEC12A	4.63E-40	CD82	6.86E-166
SAMD9	7.143E-60			HMOX1	6.64E-40	CALM2	2.96E-165
CLIC1	1.306E-59			HMGA1	1.063E-39	VMA21	3.3E-165
RGS10	5.963E-59			TES	1.131E-39	PLA2G15	3.6E-165
CDKN1B	9.047E-59			TPM4	1.759E-37	PICALM	8.17E-164
SENP7	9.095E-59			SPP1	2.558E-37	TCN2	1.16E-163
PIM1	6.567E-58			ARL4C	3.472E-37	CIR1	1.18E-163
ABI3	1.131E-57			RAB32	3.512E-37	SDSL	1.74E-163
TAP1	2.036E-57			STAT1	1.214E-36	UBE2J1	8.05E-163
BLNK	7.072E-57			IL18	1.971E-36	TMEM70	2.52E-162
LCP1	9.641E-57			PILRA	2.056E-36	ARHGDIA	8.82E-162
EIF3K	1.083E-56			PSMB3	2.688E-36	PQLC3	2.41E-161
CYB561A3	4.464E-56			HLA-DMA	8.684E-36	ID2	1.08E-160
TMPO	2.003E-55			SGK1	2.433E-35	C20orf24	2.25E-160
SEMA4D	2.368E-55			TNFAIP3	4E-35	VMP1	2.33E-160
CNOT6L	2.794E-55			TUBA1B	7.309E-35	TCEB1	2.92E-160
RGS2	3.797E-55			CSF3R	9.435E-35	TGFB1	7.13E-160
SLC38A1	6.134E-55			TNFSF10	2.384E-34	RNF213	2.94E-159
PRKCH	6.897E-55			BTG1	4.781E-34	CCDC109E	9.7E-159
BST2	7.716E-55			TKT	8.392E-34	ATF5	1.39E-158
COMMD6	1.29E-54			STXBP2	4.01E-33	MYOF	1.99E-158
LBR	1.949E-54			GPR34	6.28E-33	ANXA1	3.18E-158
DAPP1	2.652E-54			PLBD1	3.97E-28	C7orf73	3.99E-158
CCDC88C	5.576E-54			AXL	1.07E-27	PSMD7	4.03E-158
PSIP1	6.254E-54			NR4A2	1.131E-27	MIR4435-2	1.46E-157
SLFN5	9.181E-54			CREM	1.684E-27	UNC93B1	4.09E-157
ITGB3BP	1.584E-53			YWHAH	1.038E-25	RAC1	2.41E-155
C10orf54	1.859E-53			THBS1	4.738E-25	RAB13	3.59E-154
SNHG25	6.707E-53			GSN	7.133E-25	GBP5	2.84E-153
FCRL5	1.333E-52			CD302	6.303E-24	VAMP8	9.47E-153
TCF4	1.619E-52			IL1B	1.384E-23	CPQ	1.68E-152
ARID5B	3.07E-52			CCL2	6.468E-15	B3GNT2	4.78E-152
CCDC109E	4.95E-52			IFITM3	1.257E-10	LPAR6	2.87E-151
OFD1	1.907E-51			IL7R	6.775E-08	SPCS3	1.03E-150
NUSAP1	4.308E-51					RAB7A	3.24E-150
IER2	7.548E-51					STX4	3.53E-150
PDE4B	1.296E-49					PDIA3	4.99E-150
SLC25A6	4.27E-47					FDX1	8.69E-150
MZB1	9.215E-47					GBP2	1.7E-149
IGHG2	2.238E-46					IL13RA1	1.19E-148
AIM1	2.614E-46					MITF	1.06E-147
RP11-467L	5.108E-45					RNH1	1.13E-146
IGHG1	1.718E-44					PLEC	2.35E-146
OST4	8.816E-43					PDIA4	5.64E-145
SRGN	5.907E-41					NCF1	7.56E-145
ACTG1	1.31E-39					P4HB	9.66E-145
PLAC8	3.404E-39					TMEM50A	1.58E-144
JCHAIN	3.997E-36					IGF2R	4.14E-144
CCL4	1.728E-33					IRAK1	1.17E-143
HBB	2.944E-33					IFNAR1	1.54E-141
BASP1	1.605E-25					IER3	1.46E-140
IGHG3	3.385E-25					SRSF2	2.39E-140
IGHG4	6.25E-18					PRDX3	1.04E-137
S100A4	1.741E-08					ALDOA	7.64E-136
						MT-ATP8	2.48E-135
						GTF3A	2.56E-135
						CYP1B1	4.23E-135
						PIM1	5.02E-135
						FLOT1	1.02E-134
						RBMS1	1.3E-132
						CPM	6.03E-132
						AP2M1	1.32E-130
						SPRED1	4.78E-129
						NCOA4	9.87E-129
						CAPNS1	1.1E-127
						NUMB	3.79E-124
						TSPAN4	6.55E-123
						CSF1	1.56E-122
						UQCRC1	5.98E-120
						ARL4C	8.21E-120
						SLC43A2	4.83E-119
						BLOC1S1	3.36E-118
						LTA4H	4.02E-117

Cluster 10 (alveolar macrophages-1)		Cluster 11 (alveolar macrophages-2)		Cluster 12 (alveolar macrophages-3)		Cluster 13 (alveolar macrophages-4)		Cluster 14 (mesenchymal cells)	
Gene symbol	Adjusted p values	Gene symbol	Adjusted p values	Gene symbol	Adjusted p values	Gene symbol	Adjusted p values	Gene symbol	Adjusted p values
				GRB2	2.917E-74			RPS8	4.762E-60
				CXCL8	3.736E-74			PHLDB1	1.047E-59
				MYD88	1.436E-73			BACE2	1.489E-59
				HSPA1B	7.424E-73			ITPRIP	1.494E-59
				MCOLN1	2.296E-72			CTTNBP2N	1.77E-59
				TUBA1B	2.815E-71			LRRC8A	3.507E-59
				BCAP31	3.826E-68			BCR	3.096E-58
				PIK3AP1	2.004E-66			UGCG	3.461E-58
				CCL20	1.118E-65			TMSB10	6.74E-58
				HCK	4.477E-64			ARID5A	1.268E-57
				NMB	7.167E-63			DUSP23	2.206E-57
				FOLR3	1.096E-62			PEA15	2.39E-57
				EVL	2.827E-61			LAMB1	3.104E-57
				HLA-DQA1	5.721E-44			RBP1	3.206E-57
				TGM2	1.657E-27			RPS27A	3.285E-57
								CHN1	3.462E-57
								NASP	7.954E-57
								TANC1	1.575E-56
								PIEZO1	1.807E-56
								IL4R	2.16E-56
								H3F3B	3.712E-56
								HTRA1	4.239E-56
								GSTM3	1.484E-55
								NEXN	3.06E-55
								IL15RA	5.192E-55
								ACKR3	5.415E-54
								LXN	6.139E-54
								DAAM1	7.325E-54
								FOXP1	1.053E-53
								FOS	1.349E-53
								MAF	1.53E-53
								GAPDH	2.236E-53
								SORBS2	6.421E-53
								SMAD9	1.05E-52
								PPM1F	2.163E-52
								TMEM2	3.386E-52
								PLCG1	4.382E-52
								MIER2	4.728E-52
								TRIOBP	5.357E-52
								APBB2	8.045E-52
								DDIT4	1.139E-51
								MAGI1	3.021E-51
								IL32	3.582E-51
								OLFM1	4.186E-51
								IGF1	7.944E-51
								IER2	1.027E-50
								GPM6B	1.368E-50
								RPL13A	1.862E-50
								TCEAL4	2.537E-50
								SERTAD1	4.656E-50
								TUBB6	1.142E-49
								ASAP1	1.537E-49
								NOTCH1	2.201E-49
								MOB2	3.113E-49
								TNS1	3.766E-49
								SESN3	4.946E-49
								NCKAP1	9.529E-49
								HRH1	2.52E-48
								ARL2	8.35E-48
								FGFR1	9.269E-48
								IGFBP6	1.163E-47
								STAB1	1.175E-47
								HIF1A	1.48E-47
								MDK	3.044E-47
								MAP7D3	4.518E-47
								HHEX	5.658E-47
								WASF2	7.005E-47
								GALNT18	7.244E-47
								ITM2B	8.816E-47
								TPST2	9.618E-47
								ENAH	1.087E-46
								ITPR1	1.188E-46
								PIR	1.301E-46
								C4orf32	1.951E-46
								DUSP5	2.514E-46
								C1orf54	4.209E-46
								BCL3	4.262E-46
								YES1	5.091E-46

Cluster 1 (airway and alveolar epithelial cells)		Cluster 2 (ciliated cells)		Cluster 3 (type II alveolar cells-1)		Cluster 4 (type II alveolar cells-2)		Cluster 5 (type II alveolar cells-3)		Cluster 6 (immune cells)		Cluster 7 (plasma cells)		Cluster 8 (monocytes-1)		Cluster 9 (monocytes-2)		Cluster 10 (alveolar macrophages-1)		Cluster 11 (alveolar macrophages-2)		Cluster 12 (alveolar macrophages-3)		Cluster 13 (alveolar macrophages-4)		Cluster 14 (mesenchymal cells)	
**Gene symbol**	**Adjusted p values**	**Gene symbol**	**Adjusted p values**	**Gene symbol**	**Adjusted p values**	**Gene symbol**	**Adjusted p values**	**Gene symbol**	**Adjusted p values**	**Gene symbol**	**Adjusted p values**	**Gene symbol**	**Adjusted p values**	**Gene symbol**	**Adjusted p values**	**Gene symbol**	**Adjusted p values**	**Gene symbol**	**Adjusted p values**	**Gene symbol**	**Adjusted p values**	**Gene symbol**	**Adjusted p values**	**Gene symbol**	**Adjusted p values**	**Gene symbol**	**Adjusted p values**
MYH9	1.016E-76	FSD1L	1.77E-167													GTF3C6	2.36E-116									FILIP1L	5.604E-46
ITGB1	1.137E-76	HAGHL	2.96E-167													RBM47	2.93E-116									C12orf57	6.231E-46
NCOA7	1.207E-76	CNIH2	4.48E-167													ATP5G3	3.94E-116									FRYL	2.213E-45
NEBL	1.25E-76	LAMC2	1.13E-166													TIMM8B	2.68E-114									RP11-553L	3.921E-45
WNK1	1.45E-76	KCNJ16	3.78E-166													LAMTOR2	4.14E-114									CEP68	5.061E-45
RAB11B	1.561E-76	FSTL1	4E-166													CD9	4.31E-114									CD109	5.127E-45
MRPL33	2.894E-76	GLIS3	4.95E-166													HNRNPM	4.46E-112									HOTAIRM	6.289E-45
HSPB1	8.342E-76	ZKSCAN1	6.13E-166													ATP5B	1.99E-111									CDK17	9.536E-45
VMP1	6.597E-75	IFT140	8.73E-166													TXNRD1	9.21E-109									FRY	1.059E-44
LUC7L3	2.376E-74	STRBP	1.76E-165													RAN	8.58E-107									BCAR1	1.757E-44
MMP24-AS	3.027E-74	TMC4	3.9E-165													MT-ND4L	2.73E-106									ENDOD1	2.209E-44
TAPBP	7.764E-74	CLHC1	3.93E-164													DEK	4.57E-105									LEPROT	5.612E-44
IDS	4.532E-73	GCLM	8.43E-164													SDF2L1	5.96E-105									PDLIM4	6.111E-44
MZT2B	2.474E-72	NAT14	3.98E-163													TMED9	3.25E-102									SEPT7	6.231E-44
MT-ND4L	3.051E-72	CBY1	2.31E-162													TUBA1C	5.301E-97									PVRL2	2.435E-43
EID1	4.707E-72	FAM27C	5.26E-162													FNIP2	1.495E-94									SUN1	3.064E-43
SESTD1	9.245E-72	LZTFL1	5.9E-162													CCL4L2	4.211E-93									RPS18	4.274E-43
AHNAK	7.142E-71	ERICH5	6.75E-162													PSMB3	2.715E-90									DUSP1	6.788E-43
UNC13D	5.071E-69	ANKRD26	1.2E-161													ANXA4	9.706E-90									DSTN	3.466E-42
EIF4G2	3.951E-68	IGF1R	1.28E-161													RDX	2.139E-89									PTMS	3.586E-42
ADRB2	3.191E-67	PPP1R16A	4.49E-161													ALCAM	2.181E-89									PCGF2	3.9E-42
HMGN1	5.414E-66	WDR34	1.81E-160													FUCA1	1.15E-87									CCL2	6.16E-42
RRBP1	5.81E-66	WDR19	5.11E-160													RP11-386I1	6.089E-67									MAP3K11	1.325E-41
CAPNS1	2.448E-65	B9D2	8.04E-160													MMP7	1.039E-64									CDKN1C	2.408E-41
PTPN1	3.342E-65	PRKAR1A	4.81E-159													MT1G	1.05E-61									TLE1	9.688E-41
CFL2	1.109E-64	RUVBL1	1.61E-158													LMNA	1.462E-61									ASRGL1	1.074E-40
TGFBR2	1.226E-64	DDR1	4.25E-158													MT1X	1.359E-16									RPL32	1.648E-40
PPDPF	2.604E-64	NUCB2	4.76E-158																							RBFOX2	1.768E-40
REXO2	4.941E-62	ZDHHC1	2.2E-157																							HSPA1A	2.945E-40
GLS	7.423E-61	TTC21B	1.74E-156																							PXN	2.954E-40
PDXK	2.661E-60	SLC22A23	5.06E-156																							C9orf3	3.641E-40
BCYRN1	1.612E-57	WDR35	2.11E-155																							GATA6	4.271E-40
DDIT4	1.565E-56	LRIG1	6.06E-155																							SH3D19	7.359E-40
HCFC1R1	1.324E-55	RUVBL2	6.18E-155																							SLC44A2	8.5E-40
DPYSL2	3.475E-55	GABPB1-A	7.9E-155																							EHD4	9.886E-40
SPRYD7	1.261E-54	EPB41L4B	9.82E-155																							ARID5B	1.03E-39
CXCL1	6.229E-54	TJP3	1.09E-154																							CMIP	1.075E-39
DUSP23	4.403E-53	UBXN11	2.08E-154																							NRP1	1.786E-39
FAM105A	1.06E-51	CALML4	2.13E-154																							EPB41L4A	2.202E-39
PDIA3	3.834E-50	CCDC64B	2.97E-154																							LRCH1	3.867E-39
RPL3	1.597E-48	KLHL32	7.26E-154																							IFITM1	6.552E-39
C14orf132	3.231E-47	ELK3	1.55E-153																							MAFF	6.574E-39
CLDN18	1.295E-43	CEP97	1.94E-153																							ARHGEF1	6.911E-39
C4BPA	1.149E-39	KIF3B	2.76E-153																							GABARAP	9.894E-39
TAGLN	1.146E-38	IRF6	1.05E-152																							VGLL4	2.126E-38
HNRNPA1	9.545E-38	CD59	3.15E-152																							PXDC1	2.681E-38
HPCAL1	1.654E-34	SRCIN1	3.77E-152																							SBDS	4.775E-38
		GOLM1	3.85E-152																							ROCK2	5.412E-38
		MAPK10	3.9E-152																							BHLHE40	5.619E-38
		PITPNM1	3.95E-152																							DPYSL2	8.157E-38
		ALMS1	7.29E-152																							MAP4	1.431E-37
		NSUN7	7.77E-152																							UBC	1.814E-37
		RCAN3	1.7E-151																							FAM114A1	1.846E-37
		RP11-242D	1.76E-151																							CYP1B1	1.928E-37
		CTD-2015H	2.38E-151																							EGR1	3.352E-37
		CFH	4.08E-151																							CCNL1	5.362E-37
		COQ4	9.94E-151																							NR4A1	7.57E-37
		HSPA4L	2.44E-150																							VCL	9.435E-37
		NEGR1	8.03E-150																							RHOB	9.668E-37
		KIAA1671	1.84E-149																							KLF7	1.385E-36
		KB-1562D1	4.5E-149																							GNAI2	1.593E-36
		BASP1	9.5E-149																							PTK2	1.769E-36
		GLB1L2	1.22E-148																							EIF4A1	5.758E-36
		PGAP1	1.71E-148																							AFF1	6.852E-36
		APP	2.14E-148																							KMT2E	7.753E-36
		SAA1	4.65E-148																							ETS2	8.29E-36
		ZMYND12	9.52E-148																							TMCC3	1.303E-35
		PTRH1	3.15E-147																							CTTN	1.63E-35
		ACYP1	4.12E-147																							ITGAV	1.802E-35
		SHANK2	5.61E-147																							RGS3	1.997E-35
		SPATA6L	1.47E-146																							ASS1	2.101E-35
		PBX1	1.57E-146																							FAM118A	2.629E-35
		CLSTN1	1.85E-146																							BTF3	4.044E-35
		MYH14	1.92E-146																							RNF144B	4.981E-35
		PART1	1.93E-146																							GIT1	6.463E-35
		AZIN1-AS1	2.05E-146																							TPD52L1	1.081E-34
		LXN	2.56E-146																							CBX6	1.434E-34
		SYNE1	5.48E-146																							DIXDC1	1.559E-34

Cluster 1 (airway and alveolar epithelial cells)		Cluster 2 (ciliated cells)		Cluster 3 (type II alveolar cells-1)		Cluster 4 (type II alveolar cells-2)		Cluster 5 (type II alveolar cells-3)		Cluster 6 (immune cells)		Cluster 7 (plasma cells)		Cluster 8 (monocytes-1)		Cluster 9 (monocytes-2)		Cluster 10 (alveolar macrophages-1)		Cluster 11 (alveolar macrophages-2)		Cluster 12 (alveolar macrophages-3)		Cluster 13 (alveolar macrophages-4)		Cluster 14 (mesenchymal cells)	
Gene symbol	Adjusted p values	Gene symbol	Adjusted p values	Gene symbol	Adjusted p values	Gene symbol	Adjusted p values	Gene symbol	Adjusted p values	Gene symbol	Adjusted p values	Gene symbol	Adjusted p values	Gene symbol	Adjusted p values	Gene symbol	Adjusted p values	Gene symbol	Adjusted p values	Gene symbol	Adjusted p values	Gene symbol	Adjusted p values	Gene symbol	Adjusted p values	Gene symbol	Adjusted p values
		KNDC1	9.97E-146																							LPAR6	1.805E-34
		MYEF2	1.17E-145																							ANO6	5.047E-34
		SEC14L3	2E-145																							RBMS1	7.177E-34
		DSTN	4.22E-145																							HSPA1B	9.331E-34
		NFIB	4.56E-145																							COL8A1	9.435E-34
		SOX4	2.02E-143																							PROCR	1.852E-33
		SUN1	2.35E-143																							SNHG7	4.092E-33
		PPOX	1.03E-142																							PLEC	5.526E-33
		KIAA1456	1.55E-142																							SLFN5	7.991E-33
		SOD1	2.24E-142																							TMEM109	1.009E-32
		FAM104B	9.39E-142																							ZBTB38	1.226E-32
		STPG1	1.02E-141																							FGF7	1.641E-32
		PSCA	1.37E-141																							MCTP1	4.104E-32
		TTBK2	2.68E-141																							LTBP3	4.37E-32
		ARL3	2.84E-141																							KLF10	7.628E-32
		STK36	3.82E-141																							GBP4	8.467E-32
		MDM1	6.51E-141																							MRFAP1	1.313E-31
		AGR2	7.4E-141																							ODF2L	1.413E-31
		TMEM123	7.43E-141																							PCSK7	4.575E-31
		TRIM56	9.27E-141																							SERINC3	5.531E-31
		KIF3A	1.87E-140																							ADD1	5.832E-31
		SCGB3A1	2.12E-140																							CIRBP	6.176E-31
		TDRP	2.52E-140																							SOD2	6.544E-31
		TMEM107	1.02E-139																							NAA10	8.438E-31
		DUSP14	1.05E-139																							MGAT4A	1.057E-30
		PCAT19	1.54E-139																							GPM6A	1.222E-30
		DNAL4	1.56E-139																							IGFBP2	1.619E-30
		DTX3	1.6E-139																							MAP4K4	2.15E-30
		UBE3D	2.46E-139																							RGL2	2.258E-30
		LMLN	3.24E-139																							KLF3	2.828E-30
		CATIP	5.09E-139																							DDX17	2.938E-30
		PLEKHA5	5.2E-139																							UAP1	4.441E-30
		NUDT4	6.24E-139																							FOXO3	7.254E-30
		KIAA0556	4.69E-138																							FBXO21	7.559E-30
		GCC2	9.75E-138																							MAST4	9.888E-30
		FXYD1	1.86E-137																							UTRN	1.039E-29
		ODF2	2.1E-137																							IGF1R	1.06E-29
		HSD11B1L	2.21E-137																							ELMO1	1.371E-29
		KIF27	2.31E-137																							ZFP36L2	1.527E-29
		ZBED5-AS	3.45E-137																							ATRX	2.394E-29
		NQO1	4.04E-136																							SRSF5	2.443E-29
		ZNF664	6.16E-136																							TRIM56	3.467E-29
		CRNDE	1.22E-135																							ZNF532	3.555E-29
		PLXNB2	1.24E-135																							BAG3	6.567E-29
		DFNB31	1.63E-135																							PPP1R12B	7.084E-29
		ABCA5	6.21E-135																							ITGB1BP1	7.298E-29
		CNTRL	6.28E-135																							PTP4A3	7.797E-29
		GPC3	8.5E-135																							ATN1	1.158E-28
		TTLL5	4.19E-134																							SEPN1	1.345E-28
		REEP1	5.34E-134																							MXD4	1.438E-28
		ENPP5	1.16E-133																							VAMP2	3.033E-28
		CLDN1	1.19E-133																							SH3BGRL2	4.042E-28
		BAIAP2L1	6.51E-133																							RNF115	4.944E-28
		SCD5	7.16E-133																							AMOTL2	5.265E-28
		SOX9	1.87E-132																							HYAL1	9.388E-28
		CDC14A	9.24E-132																							NFE2L1	1.02E-27
		CEP162	1.09E-131																							CYB5R3	1.032E-27
		DUSP4	2.2E-131																							MBNL2	1.21E-27
		FBXW9	2.2E-131																							MYL12A	1.227E-27
		ANKRD65	2.66E-131																							AC013461.	1.541E-27
		KIAA1324	3.27E-131																							FN1	1.646E-27
		ERBB2	6.11E-131																							NFYB	1.677E-27
		DUSP19	1.08E-130																							PNP	2.006E-27
		ZDHHC11	4.72E-130																							PSIP1	2.046E-27
		CFAP36	6.58E-130																							AAMDC	2.183E-27
		ZSCAN18	6.97E-130																							SON	2.688E-27
		APPL2	8.82E-130																							HNRNPA1	2.938E-27
		ADAM12	9.69E-130																							LATS2	4.393E-27
		SSBP3	1.43E-129																							HIP1	4.946E-27
		BCAS3	2.07E-129																							SERPINF1	5.199E-27
		FLNB	2.3E-129																							NAMPT	5.205E-27
		PPP1R14C	4.46E-129																							RRAS	8.083E-27
		H2AFJ	4.71E-129																							OAZ2	9.709E-27
		SLC25A29	1.02E-128																							PKD2	1.123E-26
		TMEM154	2.31E-128																							ZBTB20	1.141E-26
		LRWD1	4.05E-128																							BTG2	1.415E-26
		DNAJC10	6.74E-128																							C10orf128	1.672E-26
		CCDC190	1.06E-127																							ST6GAL1	2.386E-26

Cluster 1 (airway and alveolar epithelial cells)		Cluster 2 (ciliated cells)		Cluster 3 (type II alveolar cells-1)		Cluster 4 (type II alveolar cells-2)		Cluster 5 (type II alveolar cells-3)		Cluster 6 (immune cells)		Cluster 7 (plasma cells)		Cluster 8 (monocytes-1)		Cluster 9 (monocytes-2)		Cluster 10 (alveolar macrophages-1)		Cluster 11 (alveolar macrophages-2)		Cluster 12 (alveolar macrophages-3)		Cluster 13 (alveolar macrophages-4)		Cluster 14 (mesenchymal cells)	
Gene symbol	Adjusted p values	Gene symbol	Adjusted p values	Gene symbol	Adjusted p values	Gene symbol	Adjusted p values	Gene symbol	Adjusted p values	Gene symbol	Adjusted p values	Gene symbol	Adjusted p values	Gene symbol	Adjusted p values	Gene symbol	Adjusted p values	Gene symbol	Adjusted p values	Gene symbol	Adjusted p values	Gene symbol	Adjusted p values	Gene symbol	Adjusted p values	Gene symbol	Adjusted p values
		UBE2H	2.18E-127																							CPD	3.325E-26
		C8orf34	4.17E-127																							GLTSCR2	3.692E-26
		REC8	4.34E-127																							FNBP1L	4.981E-26
		DICER1-A	5.41E-127																							RPS6	6.88E-26
		HMGN5	7.11E-127																							VAMP3	8.894E-26
		LBH	1.02E-126																							SLC26A2	1.054E-25
		ZC2HC1A	1.31E-126																							LAPTM4A	1.154E-25
		GLB1L	3.45E-126																							CFL2	1.388E-25
		FAM13A	4.34E-126																							CDC25B	1.389E-25
		CCDC138	4.36E-126																							ANXA6	1.406E-25
		AKNA	4.7E-126																							CFI	1.498E-25
		PHTF1	6.48E-126																							SMURF2	1.59E-25
		LINC00094	1.16E-125																							FMO2	2.291E-25
		ARFGEF3	1.62E-125																							NMT2	2.417E-25
		CARS	1.69E-125																							MAT2A	2.548E-25
		GRAMD3	3.53E-125																							NME4	1.141E-24
		TP53TG1	4.21E-125																							TSPAN4	1.448E-24
		ANKRD54	6.02E-125																							FAM198B	1.596E-24
		AK8	6.33E-125																							MESDC1	1.66E-24
		APOD	6.48E-125																							SERBP1	1.691E-24
		LCN2	1.95E-124																							SSH1	2.373E-24
		RAMP1	3.63E-124																							MLLT4	4.033E-24
		ABCC5	8.55E-124																							OSMR	5.441E-24
		SAA2	1.31E-123																							LINC00657	5.628E-24
		COPRS	1.5E-123																							NARF	7.303E-24
		IFT80	4.56E-123																							SLC20A1	8.148E-24
		SYTL1	5.32E-123																							NOSIP	1.643E-23
		EPPK1	6.24E-123																							SEPT2	1.875E-23
		WEE1	1.41E-122																							RERE	2.488E-23
		MAPK8IP1	3.42E-122																							MYL6	3.126E-23
		SSBP2	1.58E-121																							PARVA	4.235E-23
		TP53BP1	1.68E-121																							HERC2	4.867E-23
		NFIA	4.15E-121																							FLOT1	9.37E-23
		ARL6	8.38E-121																							PDLIM2	9.865E-23
		TMEM45B	1.45E-120																							RABAC1	1.132E-22
		ZC2HC1C	1.65E-120																							RBM39	1.389E-22
		FAIM	1.81E-120																							SLC2A3	1.68E-22
		SLC4A8	5.47E-120																							TMOD3	2.419E-22
		ABO	1.29E-119																							CDC42EP3	2.607E-22
		TMEM98	1.71E-119																							RBM17	3.97E-22
		CRISPLD1	5.35E-119																							PIK3R1	7.061E-22
		KRT8	1.03E-118																							GNA11	7.148E-22
		MFSD6	1.44E-118																							CRBN	7.936E-22
		LPAR3	1.87E-118																							KAT6A	1.121E-21
		CCNO	5.32E-118																							GSDMD	1.431E-21
		DHX40	1.56E-117																							ADD3	1.5E-21
		CIPC	1.57E-117																							MSN	1.939E-21
		COBL	2.13E-117																							MYL12B	2.255E-21
		FOXC1	3.51E-117																							C5orf24	2.368E-21
		BCYRN1	6.51E-117																							FAM63B	2.864E-21
		MUC16	9.43E-117																							SKAP2	2.958E-21
		CMTM4	1.04E-116																							FIS1	3.018E-21
		LRRC61	1.91E-116																							RAB13	5.724E-21
		PLEKHB1	4.49E-116																							N4BP2L2	5.959E-21
		ATXN7L3	6.72E-116																							WDR60	6.765E-21
		C5orf15	1.07E-115																							HIPK3	8.483E-21
		PIGR	1.23E-115																							PEAK1	1.383E-20
		SPTBN1	2.72E-115																							MMP14	1.73E-20
		MAGI1	4.04E-115																							PPP1R15A	2.899E-20
		P4HA2	5.85E-115																							FUS	2.964E-20
		ARHGAP1	5.95E-115																							SFPQ	3.379E-20
		C14orf79	8.56E-115																							LUC7L3	3.66E-20
		OSBPL3	1.06E-114																							MED13L	3.699E-20
		OXTR	3.38E-114																							MEF2A	3.81E-20
		FZD3	3.39E-114																							GYPC	4.87E-20
		DLGAP1-A	4.14E-114																							CRK	6.144E-20
		ANKRD18	4.29E-114																							CLTB	8.661E-20
		CXXC4	5.02E-114																							JMJD1C	9.84E-20
		ABHD2	6.31E-114																							PRDX2	1.12E-19
		ORAI2	8.08E-114																							FAM127A	1.232E-19
		CCDC8	1.21E-113																							SNAP23	1.719E-19
		CEP19	1.39E-113																							CCNL2	2.017E-19
		NR2F2	2.44E-113																							NUDT4	3.32E-19
		PHIP	4.05E-113																							IRF2BPL	4E-19
		EML1	4.56E-113																							FAM3C	7.697E-19
		U47924.27	5.55E-113																							SLC25A37	8.753E-19
		CDH1	5.99E-113																							PPP1R12A	1.579E-18
		FBXL2	2.5E-112																							PITPNB	1.842E-18

Cluster 1 (airway and alveolar epithelial cells)		Cluster 2 (ciliated cells)		Cluster 3 (type II alveolar cells-1)		Cluster 4 (type II alveolar cells-2)		Cluster 5 (type II alveolar cells-3)		Cluster 6 (immune cells)		Cluster 7 (plasma cells)		Cluster 8 (monocytes-1)		Cluster 9 (monocytes-2)		Cluster 10 (alveolar macrophages-1)		Cluster 11 (alveolar macrophages-2)		Cluster 12 (alveolar macrophages-3)		Cluster 13 (alveolar macrophages-4)		Cluster 14 (mesenchymal cells)	
Gene symbol	Adjusted p values	Gene symbol	Adjusted p values	Gene symbol	Adjusted p values	Gene symbol	Adjusted p values	Gene symbol	Adjusted p values	Gene symbol	Adjusted p values	Gene symbol	Adjusted p values	Gene symbol	Adjusted p values	Gene symbol	Adjusted p values	Gene symbol	Adjusted p values	Gene symbol	Adjusted p values	Gene symbol	Adjusted p values	Gene symbol	Adjusted p values	Gene symbol	Adjusted p values
		EXPH5	4.12E-112																							SF3B1	2.124E-18
		ANKRD37	6.05E-112																							SF1	2.449E-18
		CES4A	7.64E-112																							PHF14	2.534E-18
		LRBA	7.92E-112																							GINM1	2.796E-18
		SNHG10	8.2E-112																							TAGLN2	4.362E-18
		IL20RA	1.04E-111																							RFK	9.853E-18
		CERS4	1.1E-111																							WARS	1.048E-17
		LRRC45	1.56E-111																							PPP1CB	1.272E-17
		ST5	5.49E-111																							PNRC1	1.424E-17
		WLS	8.65E-111																							LRRC8C	1.512E-17
		EPS8L2	1.15E-109																							EDNRB	1.735E-17
		NAT1	1.44E-109																							CAMSAP2	2.861E-17
		ERP27	2.08E-109																							METTL7A	4.737E-17
		ZNHIT2	3.38E-109																							SHISA5	4.749E-17
		MID1	3.56E-109																							TUG1	5.571E-17
		FAM221A	4.76E-109																							SRSF3	8.872E-17
		TTC30A	7.6E-109																							ABL2	1.267E-16
		STX2	2.08E-108																							PNISR	1.615E-16
		NEIL1	2.47E-108																							RCAN1	1.681E-16
		YWHAE	6.69E-108																							TNRC6B	1.811E-16
		PCNXL4	2.46E-107																							PAPSS2	1.833E-16
		SYTL2	3.31E-107																							LMNA	4.475E-16
		MTSS1L	3.67E-107																							RBMS2	7.383E-16
		COL28A1	5.78E-107																							JADE1	1.293E-15
		RARRES2	6.97E-107																							RGCC	1.335E-15
		GRAMD1C	8.42E-107																							GLG1	1.546E-15
		WDR31	9.99E-107																							REV3L	3.577E-15
		KIAA1522	6.99E-106																							SERINC1	3.628E-15
		AES	7.65E-106																							NRP2	3.651E-15
		CRY2	1.34E-105																							RPS4Y1	4.571E-15
		IFT122	1.54E-105																							ID2	7.114E-15
		ANKRD42	2.37E-105																							SLC25A25	7.195E-15
		IGSF10	2.69E-105																							LYST	7.402E-15
		DDX17	3.33E-105																							MYCBP2	7.822E-15
		MT1E	5.16E-105																							PHLDA1	1.016E-14
		SATB1	5.43E-105																							CLK1	1.104E-14
		WDR92	9E-105																							LINC00152	1.127E-14
		BBS4	9.3E-105																							PLXND1	1.91E-14
		AMPD3	1.09E-104																							AAK1	2.35E-14
		YBX3	1.21E-104																							HNRNPM	3.503E-14
		PVRL4	1.34E-104																							TLN1	9.988E-14
		RARRES1	2.26E-104																							SYPL1	1.059E-13
		CASC4	2.33E-104																							SSFA2	1.109E-13
		TSPYL4	3.34E-104																							TIMP2	1.137E-13
		SPPL2B	5.3E-104																							FGD5-AS1	2.016E-13
		BBS5	1.26E-103																							NFAT5	9.325E-13
		HDLBP	1.98E-103																							CLEC2B	1.156E-12
		KCTD1	4.78E-103																							PPIC	1.203E-12
		SCAMP4	8.62E-103																							PPP3CA	1.775E-12
		GPM6B	9.09E-103																							SRSF7	2.583E-12
		EPCAM	9.29E-103																							MPZL2	6.427E-12
		ACAP1	1.14E-102																							PGRMC2	7.204E-12
		ERGIC3	1.61E-102																							SLK	3.078E-11
		SYTL3	1.64E-102																							MARCH6	6.799E-11
		COLCA2	1.64E-102																							JUN	1.35E-10
		ZNF3	1.67E-102																							SLC4A7	3.123E-10
		CASZ1	2.89E-102																							G3BP1	7.038E-10
		UGDH	4.65E-102																							PON2	1.177E-09
		ITGA2	7.65E-102																							FOSB	2.324E-09
		ULK4	1.05E-101																							CXCL2	5.347E-09
		CTD-2186M	1.63E-101																							NFE2L2	8.04E-09
		PKD1	4.35E-101																							KCNQ1OT	3.575E-07
		LRRC27	8.54E-101																								
		MBOAT2	1.37E-100																								
		STIM2	3.17E-100																								
		TRPV4	1.2E-99																								
		LRP2BP	2.18E-99																								
		IFT74	2.31E-99																								
		PRUNE2	6.28E-99																								
		ADRB1	6.85E-99																								
		ATXN2	7.99E-99																								
		FAM188B	1.016E-98																								
		AP4M1	1.414E-98																								
		FAT1	1.507E-98																								
		KIAA0232	1.821E-98																								
		TMEM254	3.2E-98																								
		WDR27	4.435E-98																								
		SLFN5	4.7E-98																								

Cluster 1 (airway and alveolar epithelial cells)		Cluster 2 (ciliated cells)		Cluster 3 (type II alveolar cells-1)		Cluster 4 (type II alveolar cells-2)		Cluster 5 (type II alveolar cells-3)		Cluster 6 (immune cells)		Cluster 7 (plasma cells)		Cluster 8 (monocytes-1)		Cluster 9 (monocytes-2)		Cluster 10 (alveolar macrophages-1)		Cluster 11 (alveolar macrophages-2)		Cluster 12 (alveolar macrophages-3)		Cluster 13 (alveolar macrophages-4)		Cluster 14 (mesenchymal cells)	
**Gene symbol**	**Adjusted p values**	**Gene symbol**	**Adjusted p values**	**Gene symbol**	**Adjusted p values**	**Gene symbol**	**Adjusted p values**	**Gene symbol**	**Adjusted p values**	**Gene symbol**	**Adjusted p values**	**Gene symbol**	**Adjusted p values**	**Gene symbol**	**Adjusted p values**	**Gene symbol**	**Adjusted p values**	**Gene symbol**	**Adjusted p values**	**Gene symbol**	**Adjusted p values**	**Gene symbol**	**Adjusted p values**	**Gene symbol**	**Adjusted p values**	**Gene symbol**	**Adjusted p values**
		APBB1	5.575E-98																								
		C10orf95	6.085E-98																								
		TMF1	1.025E-97																								
		KIFAP3	1.117E-97																								
		C5orf45	2.275E-97																								
		TEAD1	2.582E-97																								
		MLXIP	3.886E-97																								
		FAM84A	5.843E-97																								
		MKS1	1.123E-96																								
		POU2AF1	1.958E-96																								
		SLC35A3	2.251E-96																								
		DCDC2	2.816E-96																								
		PRRG4	2.893E-96																								
		C11orf63	3.595E-96																								
		NEDD4L	7.509E-96																								
		TNRC6B	1.919E-95																								
		SLC35A2	4.581E-95																								
		SMYD2	8.065E-95																								
		ZNF599	9.674E-95																								
		ANAPC4	1.669E-94																								
		MEIS2	1.853E-94																								
		HSD17B13	4.909E-94																								
		NCALD	5.453E-94																								
		KIAA0825	6.04E-94																								
		GALNS	7.656E-94																								
		PRR7	1.01E-93																								
		SERPINF1	1.428E-93																								
		DIXDC1	1.572E-93																								
		MZF1-AS1	2.081E-93																								
		NET1	2.143E-93																								
		LINC00240	8.313E-93																								
		ZNF688	8.708E-93																								
		PER2	9.772E-93																								
		SPATS2L	6.521E-92																								
		CXorf57	6.936E-92																								
		ITGB8	8.29E-92																								
		PAQR7	1.192E-91																								
		KRT19	1.794E-91																								
		LMO7	2.22E-91																								
		TULP3	4.238E-91																								
		PAWR	7.41E-91																								
		NAV2	1.188E-90																								
		ARHGAP3	1.309E-90																								
		MIPEP	4.179E-90																								
		FDXR	4.352E-90																								
		NPIPA1	4.865E-90																								
		FGFR1OP	4.993E-90																								
		KANSL1L	1.617E-89																								
		EPB41L4A	1.978E-89																								
		RASSF9	2.374E-89																								
		EVA1C	2.543E-89																								
		PWWP2B	3.451E-89																								
		RNF32	4.447E-89																								
		CDH26	6.774E-89																								
		GNA11	1.15E-88																								
		KCTD12	1.197E-88																								
		RHOU	1.876E-88																								
		TMEM68	2.075E-88																								
		CTA-293F1	2.191E-88																								
		SPATA7	2.22E-88																								
		UBL3	3.114E-88																								
		MISP	3.438E-88																								
		WDPCP	7.271E-88																								
		GOLGA6L	8.495E-88																								
		KIAA0895	1.3E-87																								
		ACOT11	1.303E-87																								
		MARCH6	1.765E-87																								
		CEP170B	4.625E-87																								
		MAGIX	5.096E-87																								
		MUC12	5.928E-87																								
		DAAM1	7.257E-87																								
		MSH3	8.625E-87																								
		RP11-798M	1.187E-86																								
		OCEL1	1.419E-86																								
		GULP1	1.563E-86																								
		BTBD3	1.768E-86																								
		GCLC	2.237E-86																								
		MT-CO2	4.027E-86																								

Cluster 1 (airway and alveolar epithelial cells)		Cluster 2 (ciliated cells)		Cluster 3 (type II alveolar cells-1)		Cluster 4 (type II alveolar cells-2)		Cluster 5 (type II alveolar cells-3)		Cluster 6 (immune cells)		Cluster 7 (plasma cells)		Cluster 8 (monocytes-1)		Cluster 9 (monocytes-2)		Cluster 10 (alveolar macrophages-1)		Cluster 11 (alveolar macrophages-2)		Cluster 12 (alveolar macrophages-3)		Cluster 13 (alveolar macrophages-4)		Cluster 14 (mesenchymal cells)	
Gene symbol	Adjusted p values	Gene symbol	Adjusted p values	Gene symbol	Adjusted p values	Gene symbol	Adjusted p values	Gene symbol	Adjusted p values	Gene symbol	Adjusted p values	Gene symbol	Adjusted p values	Gene symbol	Adjusted p values	Gene symbol	Adjusted p values	Gene symbol	Adjusted p values	Gene symbol	Adjusted p values	Gene symbol	Adjusted p values	Gene symbol	Adjusted p values	Gene symbol	Adjusted p values
		LINC00467	4.128E-86																								
		UGCG	4.545E-86																								
		ABCD3	4.67E-86																								
		HDGF	4.826E-86																								
		NEK4	6.567E-86																								
		CAST	7.595E-86																								
		PPP4R3B	1.854E-85																								
		CAP2	2.183E-85																								
		FMO2	9.293E-85																								
		CGN	2.212E-84																								
		CMIP	2.851E-84																								
		CETN3	3.72E-84																								
		CCDC125	4.075E-84																								
		CCDC66	4.479E-84																								
		GLT8D1	5.217E-84																								
		MARVELD	5.995E-84																								
		EPN2	6.688E-84																								
		CEP78	9.419E-84																								
		GTF2IRD2	8.069E-83																								
		PAIP2B	8.911E-83																								
		C11orf49	9.402E-83																								
		PLS1	1.602E-82																								
		PIAS3	1.902E-82																								
		CFAP20	2.097E-82																								
		FZD6	2.22E-82																								
		TTC5	2.977E-82																								
		RB1CC1	3.012E-82																								
		DNAAF5	4.846E-82																								
		UBN2	5.183E-82																								
		FOCAD	6.072E-82																								
		IFI27	6.329E-82																								
		ETNK1	6.356E-82																								
		TC2N	1.105E-81																								
		PGRMC1	2.792E-81																								
		SCPEP1	2.981E-81																								
		RPA3	4.867E-81																								
		TMEM173	4.87E-81																								
		USP51	4.984E-81																								
		C19orf33	5.27E-81																								
		XPNPEP3	5.561E-81																								
		TMPRSS3	7.805E-81																								
		RUNDC1	9.538E-81																								
		ZC3H6	1.791E-80																								
		BCL2L1	2.145E-80																								
		WDR73	2.465E-80																								
		GAS6	3.212E-80																								
		KIAA1217	3.311E-80																								
		CEP89	3.549E-80																								
		TAX1BP1	5.389E-80																								
		ADGRG1	5.669E-80																								
		BBS9	8.08E-80																								
		ADCK4	8.844E-80																								
		F11R	2.169E-79																								
		ANKRD36	2.991E-79																								
		AC062017.	3.035E-79																								
		PSMC3IP	3.038E-79																								
		MIR99AHC	3.691E-79																								
		FOXP2	4.824E-79																								
		XRRA1	6.627E-79																								
		DNAJC16	6.731E-79																								
		AHSA1	8.925E-79																								
		PSD3	9.032E-79																								
		ERBB4	9.054E-79																								
		NBL1	9.349E-79																								
		KLK11	1.196E-78																								
		HIST1H2B	1.375E-78																								
		PROSER3	1.593E-78																								
		CAPN2	1.69E-78																								
		PNMA1	2.038E-78																								
		TMEM59	2.566E-78																								
		MT-ND4	2.725E-78																								
		BAIAP2	3.017E-78																								
		BPIFB1	5.849E-78																								
		TTC12	7.829E-78																								
		CEP164	2.16E-77																								
		CRIM1	3.738E-77																								
		RAD9A	5.369E-77																								
		GNG12	1.284E-76																								

Cluster 1 (airway and alveolar epithelial cells)		Cluster 2 (ciliated cells)		Cluster 3 (type II alveolar cells-1)		Cluster 4 (type II alveolar cells-2)		Cluster 5 (type II alveolar cells-3)		Cluster 6 (immune cells)		Cluster 7 (plasma cells)		Cluster 8 (monocytes-1)		Cluster 9 (monocytes-2)		Cluster 10 (alveolar macrophages-1)		Cluster 11 (alveolar macrophages-2)		Cluster 12 (alveolar macrophages-3)		Cluster 13 (alveolar macrophages-4)		Cluster 14 (mesenchymal cells)	
Gene symbol	Adjusted p values	Gene symbol	Adjusted p values	Gene symbol	Adjusted p values	Gene symbol	Adjusted p values	Gene symbol	Adjusted p values	Gene symbol	Adjusted p values	Gene symbol	Adjusted p values	Gene symbol	Adjusted p values	Gene symbol	Adjusted p values	Gene symbol	Adjusted p values	Gene symbol	Adjusted p values	Gene symbol	Adjusted p values	Gene symbol	Adjusted p values	Gene symbol	Adjusted p values
		AHSA2	1.797E-76																								
		EPHX2	1.872E-76																								
		KCNE1	3.597E-76																								
		FAM60A	3.601E-76																								
		NLRP1	3.754E-76																								
		SUDS3	4.839E-76																								
		ATF7IP2	6.871E-76																								
		DDAH1	7.268E-76																								
		WWC1	7.879E-76																								
		AGBL5	8.978E-76																								
		BCL7A	9.994E-76																								
		TRAF4	1.825E-75																								
		LENG8	3.182E-75																								
		H3F3B	3.379E-75																								
		ALCAM	3.535E-75																								
		SMPD2	7.902E-75																								
		RALGPS2	8.754E-75																								
		KATNB1	8.945E-75																								
		SRGAP1	1.351E-74																								
		BBIP1	1.975E-74																								
		MEAF6	3.38E-74																								
		ZFP36L1	3.562E-74																								
		POFUT2	2.037E-73																								
		ANKZF1	2.716E-73																								
		PTOV1	2.975E-73																								
		ARSD	4.748E-73																								
		RAB11FIP4	4.826E-73																								
		GFOD2	5.54E-73																								
		EFCAB11	6.047E-73																								
		TMEM14B	6.477E-73																								
		RP11-792A	7.763E-73																								
		ZNF33B	1.192E-72																								
		HECTD1	2.028E-72																								
		APITD1	2.995E-72																								
		CTBP2	3.311E-72																								
		HNRNPF	4.976E-72																								
		CTTN	5.131E-72																								
		ERBB3	1.206E-71																								
		INTS10	1.21E-71																								
		HIPK3	1.242E-71																								
		FARP1	1.501E-71																								
		MNAT1	1.673E-71																								
		REPIN1	1.681E-71																								
		UNC93B1	1.916E-71																								
		CHCHD6	2.55E-71																								
		MTA1	2.905E-71																								
		ADPRHL2	3.051E-71																								
		TOB1	3.892E-71																								
		HMGB3	5.926E-71																								
		LLGL2	6.577E-71																								
		VPS35	1.137E-70																								
		FAM63B	2.053E-70																								
		CFDP1	2.293E-70																								
		WDTC1	3.947E-70																								
		PALLD	5.124E-70																								
		PLEKHA4	5.607E-70																								
		PXN	7.751E-70																								
		OTUD4	1.155E-69																								
		TTC9	1.175E-69																								
		ZDHHC11B	1.39E-69																								
		CBX5	4.562E-69																								
		SLC25A14	5.226E-69																								
		MPC2	6.73E-69																								
		PCDH7	7.006E-69																								
		APOO	8.136E-69																								
		WDR13	9.332E-69																								
		SORD	9.743E-69																								
		PSIP1	1.347E-68																								
		TMBIM6	1.385E-68																								
		ZDHHC13	1.784E-68																								
		STYXL1	2.551E-68																								
		PAPOLA	4.198E-68																								
		BACE2	4.542E-68																								
		ZNF609	6.268E-68																								
		TRMT1L	9.858E-68																								
		MED25	1.907E-67																								
		SMIM8	6.869E-67																								
		JAG1	9.552E-67																								

Cluster 1 (airway and alveolar epithelial cells)		Cluster 2 (ciliated cells)		Cluster 3 (type II alveolar cells-1)		Cluster 4 (type II alveolar cells-2)		Cluster 5 (type II alveolar cells-3)		Cluster 6 (immune cells)		Cluster 7 (plasma cells)		Cluster 8 (monocytes-1)		Cluster 9 (monocytes-2)		Cluster 10 (alveolar macrophages-1)		Cluster 11 (alveolar macrophages-2)		Cluster 12 (alveolar macrophages-3)		Cluster 13 (alveolar macrophages-4)		Cluster 14 (mesenchymal cells)	
Gene symbol	Adjusted p values	Gene symbol	Adjusted p values	Gene symbol	Adjusted p values	Gene symbol	Adjusted p values	Gene symbol	Adjusted p values	Gene symbol	Adjusted p values	Gene symbol	Adjusted p values	Gene symbol	Adjusted p values	Gene symbol	Adjusted p values	Gene symbol	Adjusted p values	Gene symbol	Adjusted p values	Gene symbol	Adjusted p values	Gene symbol	Adjusted p values	Gene symbol	Adjusted p values
		TBL1XR1	1.079E-66																								
		NELFE	3.088E-66																								
		KIF2A	3.21E-66																								
		PRKAR2A	3.374E-66																								
		FAM206A	3.448E-66																								
		NDFIP2	4.609E-66																								
		ITGA3	5.826E-66																								
		CRIP1	6.203E-66																								
		SLC20A2	1.043E-65																								
		CDC16	1.716E-65																								
		ABHD11	2.512E-65																								
		SMIM19	2.811E-65																								
		RASSF6	2.974E-65																								
		SLC12A7	3.102E-65																								
		VEZF1	3.689E-65																								
		IQCB1	4.967E-65																								
		FCF1	1.123E-64																								
		TJP2	1.395E-64																								
		KTN1	1.855E-64																								
		RHOBTB3	3.269E-64																								
		ZBTB10	3.85E-64																								
		ANXA4	6.228E-64																								
		HACD4	7.729E-64																								
		SQLE	2.07E-63																								
		ZNF264	3.22E-63																								
		MZF1	3.259E-63																								
		DNAAF2	3.62E-63																								
		NAB1	4.123E-63																								
		GOLPH3L	4.479E-63																								
		TRAK1	5.04E-63																								
		MT1F	5.302E-63																								
		TOMM34	6.036E-63																								
		SMIM14	9.165E-63																								
		DYRK2	1.212E-62																								
		SARAF	1.742E-62																								
		KLF5	2.339E-62																								
		ZBTB18	3.514E-62																								
		CSNK1E	3.632E-62																								
		ANK3	4.318E-62																								
		PPL	6.83E-62																								
		LIPH	7.678E-62																								
		EHF	7.774E-62																								
		GK5	1.059E-61																								
		C9orf72	1.112E-61																								
		C1orf228	1.179E-61																								
		MTF1	1.712E-61																								
		GALNT3	1.718E-61																								
		SCIN	1.899E-61																								
		MT-ND6	2.761E-61																								
		PHC1	3.122E-61																								
		MGP	8.744E-61																								
		MPZL3	8.87E-61																								
		REV3L	1.129E-60																								
		MIDN	1.226E-60																								
		GFPT1	1.321E-60																								
		ECI2	1.373E-60																								
		COG7	1.625E-60																								
		NR2F6	2.117E-60																								
		FAM149A	2.806E-60																								
		KDM1B	2.815E-60																								
		SNX7	3.661E-60																								
		KLHDC10	4.029E-60																								
		GFM2	4.253E-60																								
		ST6GALN[illegible]	5.173E-60																								
		THADA	5.358E-60																								
		CUEDC1	2.362E-59																								
		ARHGEF1[illegible]	2.432E-59																								
		PPP4R1	3.161E-59																								
		EPHA2	3.203E-59																								
		CES2	3.872E-59																								
		SLCO3A1	5.679E-59																								
		IKZF2	7.284E-59																								
		GAS7	8.266E-59																								
		VGLL4	8.365E-59																								
		GRAMD2	1.108E-58																								
		MUM1	1.287E-58																								
		EFEMP1	1.423E-58																								
		WDR45B	1.475E-58																								

Cluster 1 (airway and alveolar epithelial cells)		Cluster 2 (ciliated cells)		Cluster 3 (type II alveolar cells-1)		Cluster 4 (type II alveolar cells-2)		Cluster 5 (type II alveolar cells-3)		Cluster 6 (immune cells)		Cluster 7 (plasma cells)		Cluster 8 (monocytes-1)		Cluster 9 (monocytes-2)		Cluster 10 (alveolar macrophages-1)		Cluster 11 (alveolar macrophages-2)		Cluster 12 (alveolar macrophages-3)		Cluster 13 (alveolar macrophages-4)		Cluster 14 (mesenchymal cells)	
Gene symbol	Adjusted p values	Gene symbol	Adjusted p values	Gene symbol	Adjusted p values	Gene symbol	Adjusted p values	Gene symbol	Adjusted p values	Gene symbol	Adjusted p values	Gene symbol	Adjusted p values	Gene symbol	Adjusted p values	Gene symbol	Adjusted p values	Gene symbol	Adjusted p values	Gene symbol	Adjusted p values	Gene symbol	Adjusted p values	Gene symbol	Adjusted p values	Gene symbol	Adjusted p values
		RERE	1.665E-58																								
		MAP7	2.934E-58																								
		FAM174B	3.509E-58																								
		SIPA1L3	5.849E-58																								
		AC159540.	6.075E-58																								
		AGRN	6.371E-58																								
		EVI5	6.475E-58																								
		EP400	9.375E-58																								
		INADL	1.077E-57																								
		PPP2CB	1.145E-57																								
		TM9SF2	1.185E-57																								
		INSR	1.485E-57																								
		SH3BP4	2.737E-57																								
		LYPLA2	3.779E-57																								
		MT-ND2	3.791E-57																								
		GOLGB1	4.123E-57																								
		RIC3	5.427E-57																								
		MDK	6.191E-57																								
		TSPAN3	7.165E-57																								
		HOOK1	7.966E-57																								
		WHSC1L1	9.434E-57																								
		TTLL3	1.158E-56																								
		ZBTB44	1.74E-56																								
		H3F3A	2.143E-56																								
		TRIM37	3.163E-56																								
		RBM43	3.164E-56																								
		LAMA5	3.907E-56																								
		PKD2	7.31E-56																								
		PUM2	7.833E-56																								
		OXR1	1.123E-55																								
		ADD3	1.207E-55																								
		TMEM30B	1.227E-55																								
		LINC00969	1.343E-55																								
		LYRM7	1.502E-55																								
		MTURN	2.822E-55																								
		LY75	2.976E-55																								
		TSC22D1	3.763E-55																								
		ZNF704	5.905E-55																								
		RPP38	5.94E-55																								
		GALK2	6.375E-55																								
		CSNK1A1	8.056E-55																								
		PAPSS1	9.011E-55																								
		RALGAPA	1.048E-54																								
		FRK	1.268E-54																								
		NFE2L2	1.401E-54																								
		SS18	1.654E-54																								
		LIMK2	1.727E-54																								
		SGPL1	2.446E-54																								
		ZNF33A	2.828E-54																								
		ZNF395	3.133E-54																								
		VRK3	6.018E-54																								
		TUBGCP2	6.588E-54																								
		IRAK1BP1	9.304E-54																								
		MLLT1	9.553E-54																								
		STK11IP	1.121E-53																								
		RAB3IP	1.248E-53																								
		SLC7A2	1.702E-53																								
		FAM172A	1.725E-53																								
		IFT52	1.985E-53																								
		SLAIN2	2.207E-53																								
		TMEM50B	2.332E-53																								
		OFD1	3.038E-53																								
		STAM2	3.46E-53																								
		ATP8B1	3.911E-53																								
		RPS6KA5	4.31E-53																								
		UAP1	4.608E-53																								
		ERF	5.084E-53																								
		TBCA	6.662E-53																								
		IFT20	7.079E-53																								
		DHRS4-AS	1.84E-52																								
		CAMSAP1	2.197E-52																								
		ULK2	2.275E-52																								
		WFS1	2.537E-52																								
		B3GNT7	3.531E-52																								
		JUP	4.159E-52																								
		HCG18	4.457E-52																								
		GMCL1	7.633E-52																								
		STEAP3	7.705E-52																								

Cluster 1 (airway and alveolar epithelial cells)		Cluster 2 (ciliated cells)		Cluster 3 (type II alveolar cells-1)		Cluster 4 (type II alveolar cells-2)		Cluster 5 (type II alveolar cells-3)		Cluster 6 (immune cells)		Cluster 7 (plasma cells)		Cluster 8 (monocytes-1)		Cluster 9 (monocytes-2)		Cluster 10 (alveolar macrophages-1)		Cluster 11 (alveolar macrophages-2)		Cluster 12 (alveolar macrophages-3)		Cluster 13 (alveolar macrophages-4)		Cluster 14 (mesenchymal cells)	
**Gene symbol**	**Adjusted p values**	**Gene symbol**	**Adjusted p values**	**Gene symbol**	**Adjusted p values**	**Gene symbol**	**Adjusted p values**	**Gene symbol**	**Adjusted p values**	**Gene symbol**	**Adjusted p values**	**Gene symbol**	**Adjusted p values**	**Gene symbol**	**Adjusted p values**	**Gene symbol**	**Adjusted p values**	**Gene symbol**	**Adjusted p values**	**Gene symbol**	**Adjusted p values**	**Gene symbol**	**Adjusted p values**	**Gene symbol**	**Adjusted p values**	**Gene symbol**	**Adjusted p values**
		MAP3K13	8.555E-52																								
		ERICH2	8.577E-52																								
		EPS8	9.455E-52																								
		TMTC3	1.035E-51																								
		MCAT	1.738E-51																								
		MT-ND5	2.064E-51																								
		GPBP1	3.101E-51																								
		SEC14L1	5.685E-51																								
		S100P	7.606E-51																								
		SLC40A1	7.656E-51																								
		HSP90AB1	8.914E-51																								
		MAL2	1.131E-50																								
		ACVR1B	1.586E-50																								
		JADE1	1.753E-50																								
		NT5DC1	1.783E-50																								
		ATP2C2	2.116E-50																								
		DTX2	2.231E-50																								
		ALKBH5	2.261E-50																								
		DIDO1	3.437E-50																								
		MTMR2	5.122E-50																								
		TRMT10A	5.257E-50																								
		ERLIN2	6.751E-50																								
		BDH1	8.501E-50																								
		SH3RF1	9.787E-50																								
		SCGB3A2	1.042E-49																								
		TMCO3	1.337E-49																								
		SEMA3C	1.685E-49																								
		DNAJB2	1.851E-49																								
		KRT18	4.267E-49																								
		C21orf2	4.66E-49																								
		UCP2	7.625E-49																								
		EPHX1	7.67E-49																								
		TPM1	8.577E-49																								
		KPNA5	8.705E-49																								
		ESYT2	9.936E-49																								
		CFLAR	2.202E-48																								
		B3GNT5	2.68E-48																								
		CXADR	2.717E-48																								
		PARD3	3.435E-48																								
		ICMT	4.063E-48																								
		LAMB3	8.287E-48																								
		ASXL2	8.65E-48																								
		IRX2	1.038E-47																								
		CD2AP	1.728E-47																								
		MYO1D	1.735E-47																								
		BANF1	2.405E-47																								
		TTC3	2.944E-47																								
		ZMAT1	5.708E-47																								
		RSRP1	6.904E-47																								
		CELF1	9.709E-47																								
		RP11-532F	1.171E-46																								
		LCOR	1.34E-46																								
		CDKN1A	1.429E-46																								
		EIF4G3	1.551E-46																								
		GBP3	2.184E-46																								
		SERPINB6	2.198E-46																								
		RBM23	2.211E-46																								
		MT-ATP6	2.716E-46																								
		CCDC186	3.365E-46																								
		ZNF516	3.886E-46																								
		NCKAP1	4.027E-46																								
		TUSC2	4.64E-46																								
		HINT1	4.81E-46																								
		NDUFC1	6.98E-46																								
		LIMCH1	1.423E-45																								
		MYO10	1.463E-45																								
		SON	1.469E-45																								
		TPRG1L	1.532E-45																								
		PRKCE	1.601E-45																								
		PPP1CB	2.066E-45																								
		PPP1R7	2.107E-45																								
		KLHL28	2.999E-45																								
		ABLIM1	3.911E-45																								
		POLD2	4.493E-45																								
		DCAF6	5.348E-45																								
		FILIP1	5.699E-45																								
		CACHD1	6.991E-45																								
		CTNND1	7.199E-45																								

Cluster 1 (airway and alveolar epithelial cells)		Cluster 2 (ciliated cells)		Cluster 3 (type II alveolar cells-1)		Cluster 4 (type II alveolar cells-2)		Cluster 5 (type II alveolar cells-3)		Cluster 6 (immune cells)		Cluster 7 (plasma cells)		Cluster 8 (monocytes-1)		Cluster 9 (monocytes-2)		Cluster 10 (alveolar macrophages-1)		Cluster 11 (alveolar macrophages-2)		Cluster 12 (alveolar macrophages-3)		Cluster 13 (alveolar macrophages-4)		Cluster 14 (mesenchymal cells)	
**Gene symbol**	**Adjusted p values**	**Gene symbol**	**Adjusted p values**	**Gene symbol**	**Adjusted p values**	**Gene symbol**	**Adjusted p values**	**Gene symbol**	**Adjusted p values**	**Gene symbol**	**Adjusted p values**	**Gene symbol**	**Adjusted p values**	**Gene symbol**	**Adjusted p values**	**Gene symbol**	**Adjusted p values**	**Gene symbol**	**Adjusted p values**	**Gene symbol**	**Adjusted p values**	**Gene symbol**	**Adjusted p values**	**Gene symbol**	**Adjusted p values**	**Gene symbol**	**Adjusted p values**
		KMT5B	1.006E-44																								
		SPINT2	1.051E-44																								
		SIGIRR	1.221E-44																								
		FAM120B	1.373E-44																								
		VMP1	1.383E-44																								
		SAMD4B	1.641E-44																								
		OCIAD2	1.656E-44																								
		CD46	2.398E-44																								
		STOM	2.753E-44																								
		MBTPS1	3.002E-44																								
		CSNK1G1	3.462E-44																								
		RNF6	3.599E-44																								
		SMARCA2	3.905E-44																								
		SESN1	3.982E-44																								
		CLIP1	5.276E-44																								
		GAPVD1	5.561E-44																								
		MAML2	9.928E-44																								
		NCBP3	1.872E-43																								
		CCDC69	1.965E-43																								
		ST14	2.323E-43																								
		PDCD4	2.92E-43																								
		SFXN3	3.492E-43																								
		EBLN3	4.466E-43																								
		ISCA2	5.772E-43																								
		MAP1LC3A	5.805E-43																								
		RAB28	6.907E-43																								
		GLYR1	1.234E-42																								
		VPS13C	1.539E-42																								
		MAP4K4	1.693E-42																								
		ITGB3BP	1.724E-42																								
		SCO2	2.178E-42																								
		SLC25A4	2.319E-42																								
		RALGDS	3.408E-42																								
		ZNF652	6.227E-42																								
		WIPI1	7.877E-42																								
		MBNL2	9.185E-42																								
		RAB11FIP1	9.297E-42																								
		SREK1	1.315E-41																								
		ARHGAP5	1.809E-41																								
		ARFIP2	2.224E-41																								
		BNIP3	2.315E-41																								
		IKZF4	2.872E-41																								
		SPATA13	3.005E-41																								
		TMEM131	5.821E-41																								
		BPTF	7.035E-41																								
		ACSS1	7.038E-41																								
		NFATC3	8.268E-41																								
		RCN1	8.726E-41																								
		AHI1	1.684E-40																								
		NGRN	1.953E-40																								
		PARVA	2.703E-40																								
		C4orf48	2.922E-40																								
		LINC00657	3.538E-40																								
		RNF19A	4.843E-40																								
		IDS	5.158E-40																								
		VDAC3	5.437E-40																								
		MDM2	5.531E-40																								
		SEPT9	5.611E-40																								
		KCNQ1OT	5.758E-40																								
		MCU	5.809E-40																								
		USP54	7.684E-40																								
		HIST1H1C	7.991E-40																								
		OCLN	8.351E-40																								
		TCTN3	1.532E-39																								
		RP11-140K	1.943E-39																								
		RARRES3	2.037E-39																								
		CSTF3	2.075E-39																								
		SLC39A6	2.188E-39																								
		IRF2BPL	2.212E-39																								
		MAST4	2.849E-39																								
		MYO1E	2.954E-39																								
		TOP2B	3.614E-39																								
		GOLGA2	3.694E-39																								
		PAK1	4.847E-39																								
		ZMYND8	5.278E-39																								
		ABHD17B	6.221E-39																								
		TMEM14A	7.31E-39																								
		INTS3	1.316E-38																								

Cluster 1 (airway and alveolar epithelial cells)		Cluster 2 (ciliated cells)		Cluster 3 (type II alveolar cells-1)		Cluster 4 (type II alveolar cells-2)		Cluster 5 (type II alveolar cells-3)		Cluster 6 (immune cells)		Cluster 7 (plasma cells)		Cluster 8 (monocytes-1)		Cluster 9 (monocytes-2)		Cluster 10 (alveolar macrophages-1)		Cluster 11 (alveolar macrophages-2)		Cluster 12 (alveolar macrophages-3)		Cluster 13 (alveolar macrophages-4)		Cluster 14 (mesenchymal cells)	
Gene symbol	Adjusted p values	Gene symbol	Adjusted p values	Gene symbol	Adjusted p values	Gene symbol	Adjusted p values	Gene symbol	Adjusted p values	Gene symbol	Adjusted p values	Gene symbol	Adjusted p values	Gene symbol	Adjusted p values	Gene symbol	Adjusted p values	Gene symbol	Adjusted p values	Gene symbol	Adjusted p values	Gene symbol	Adjusted p values	Gene symbol	Adjusted p values	Gene symbol	Adjusted p values
		SETD5	1.438E-38																								
		NUDT3	1.443E-38																								
		HEBP2	1.632E-38																								
		SPTAN1	2.06E-38																								
		NDUFAB1	2.719E-38																								
		DAPP1	3.223E-38																								
		PRRC2B	3.319E-38																								
		FOXO3	6.784E-38																								
		FOLR1	1.179E-37																								
		FAM214A	1.23E-37																								
		IDH2	1.276E-37																								
		ANAPC5	1.333E-37																								
		MAZ	1.959E-37																								
		ATP6V1D	2.511E-37																								
		JOSD2	2.894E-37																								
		THUMPD3	3.041E-37																								
		UBL5	3.232E-37																								
		RBM6	3.273E-37																								
		AKIRIN1	4.364E-37																								
		YLPM1	1.052E-36																								
		ANKRD13I	1.198E-36																								
		TRNAU1A	1.395E-36																								
		CFAP97	1.709E-36																								
		DDIT4	1.84E-36																								
		IARS2	2.054E-36																								
		CNOT11	2.529E-36																								
		SRSF10	3.722E-36																								
		HIPK2	4.401E-36																								
		RRM2B	4.56E-36																								
		FAM120A	5.353E-36																								
		CTNNA1	5.814E-36																								
		SURF2	7.655E-36																								
		KMT2A	7.764E-36																								
		PABPN1	8.04E-36																								
		RGS12	8.351E-36																								
		NAP1L4	8.375E-36																								
		MACF1	9.107E-36																								
		MORF4L1	1.124E-35																								
		SETD2	1.215E-35																								
		KDM3B	1.822E-35																								
		MACC1	2.427E-35																								
		CHD6	3.041E-35																								
		MRPL23	3.156E-35																								
		DSG2	3.387E-35																								
		CLCN3	4.194E-35																								
		BORCS7	4.616E-35																								
		CCDC28A	4.732E-35																								
		TRIP11	5.284E-35																								
		CCND1	9.537E-35																								
		FOXA2	1.084E-34																								
		NUDT14	1.168E-34																								
		NBR1	1.21E-34																								
		MAGED2	1.222E-34																								
		ZNF397	1.682E-34																								
		TPGS2	1.876E-34																								
		CCDC82	2.019E-34																								
		ILF3-AS1	2.093E-34																								
		CD38	2.62E-34																								
		CES1	4.752E-34																								
		PPID	6.09E-34																								
		ZNF428	7.432E-34																								
		USP10	1.037E-33																								
		GOLGA8B	1.118E-33																								
		FAM107B	1.206E-33																								
		STK38	1.384E-33																								
		TUG1	1.776E-33																								
		MAN1A2	1.882E-33																								
		PHACTR4	2.317E-33																								
		PSMD10	2.897E-33																								
		ANXA11	3.034E-33																								
		SMAD2	3.539E-33																								
		TOPORS-A	5.666E-33																								
		GSE1	8.617E-33																								
		ELOF1	1.08E-32																								
		RABGAP1I	1.106E-32																								
		GALC	1.163E-32																								
		ZCCHC11	1.17E-32																								
		GADD45G	1.303E-32																								

Cluster 1 (airway and alveolar epithelial cells)		Cluster 2 (ciliated cells)		Cluster 3 (type II alveolar cells-1)		Cluster 4 (type II alveolar cells-2)		Cluster 5 (type II alveolar cells-3)		Cluster 6 (immune cells)		Cluster 7 (plasma cells)		Cluster 8 (monocytes-1)		Cluster 9 (monocytes-2)		Cluster 10 (alveolar macrophages-1)		Cluster 11 (alveolar macrophages-2)		Cluster 12 (alveolar macrophages-3)		Cluster 13 (alveolar macrophages-4)		Cluster 14 (mesenchymal cells)	
Gene symbol	Adjusted p values	Gene symbol	Adjusted p values	Gene symbol	Adjusted p values	Gene symbol	Adjusted p values	Gene symbol	Adjusted p values	Gene symbol	Adjusted p values	Gene symbol	Adjusted p values	Gene symbol	Adjusted p values	Gene symbol	Adjusted p values	Gene symbol	Adjusted p values	Gene symbol	Adjusted p values	Gene symbol	Adjusted p values	Gene symbol	Adjusted p values	Gene symbol	Adjusted p values
		BLCAP	1.333E-32																								
		RASAL2	1.436E-32																								
		DDB1	2.292E-32																								
		DYNC1LI2	2.453E-32																								
		ECHDC2	2.549E-32																								
		PREPL	2.657E-32																								
		DDX42	2.962E-32																								
		PRR34-AS1	3.091E-32																								
		USP22	7.422E-32																								
		MT-ND4L	7.478E-32																								
		ANAPC16	8.104E-32																								
		UTRN	9.914E-32																								
		MGMT	1.042E-31																								
		PRPS1	1.068E-31																								
		KPNA6	1.188E-31																								
		TMX4	1.764E-31																								
		COA3	1.911E-31																								
		DNAJB6	2.49E-31																								
		LMBRD1	2.633E-31																								
		KIAA0319L	2.665E-31																								
		SSRP1	2.871E-31																								
		PPDPF	4.614E-31																								
		BCLAF1	5.901E-31																								
		GBAS	6.936E-31																								
		NF1	8.698E-31																								
		CTAGE5	8.896E-31																								
		ZBED5	1.053E-30																								
		SMARCA4	1.254E-30																								
		SNRNP200	2.574E-30																								
		DDX6	2.764E-30																								
		KPNA3	4.901E-30																								
		MT-CYB	5.453E-30																								
		CIRBP	8.005E-30																								
		FOXP1	8.207E-30																								
		LRRCC1	1.081E-29																								
		DDX3Y	1.546E-29																								
		MED31	1.648E-29																								
		AKR7A2	2.481E-29																								
		FBXO22	2.573E-29																								
		NFIC	3.274E-29																								
		SLC25A36	3.547E-29																								
		XPO1	3.722E-29																								
		USP11	3.805E-29																								
		GTF2H5	4.173E-29																								
		ZNF106	7.358E-29																								
		STX16	7.585E-29																								
		SYAP1	9.901E-29																								
		C19orf70	1.717E-28																								
		NCOA7	1.725E-28																								
		SGMS2	1.806E-28																								
		BAD	2.284E-28																								
		PNKD	2.579E-28																								
		MRPL42	2.792E-28																								
		ZFHX3	3.218E-28																								
		ENPP4	3.978E-28																								
		MMP24-AS	5.56E-28																								
		RBM5	5.696E-28																								
		CPD	7.624E-28																								
		EMB	1.01E-27																								
		AKR1C1	1.12E-27																								
		ACTN4	1.434E-27																								
		WBSCR22	1.53E-27																								
		STAT3	2.231E-27																								
		FAM8A1	2.315E-27																								
		GDF15	3.659E-27																								
		OTUD6B-A	5.117E-27																								
		CHCHD5	5.842E-27																								
		CXXC5	7.025E-27																								
		SRP9	1.071E-26																								
		IL13RA1	1.084E-26																								
		RRAGA	1.115E-26																								
		CIR1	1.137E-26																								
		ATP5I	1.381E-26																								
		PRDM2	1.411E-26																								
		MRFAP1L1	1.414E-26																								
		C10orf11	4.446E-26																								
		UPF3A	4.96E-26																								
		CHD4	5.018E-26																								

Cluster 1 (airway and alveolar epithelial cells)		Cluster 2 (ciliated cells)		Cluster 3 (type II alveolar cells-1)		Cluster 4 (type II alveolar cells-2)		Cluster 5 (type II alveolar cells-3)		Cluster 6 (immune cells)		Cluster 7 (plasma cells)		Cluster 8 (monocytes-1)		Cluster 9 (monocytes-2)		Cluster 10 (alveolar macrophages-1)		Cluster 11 (alveolar macrophages-2)		Cluster 12 (alveolar macrophages-3)		Cluster 13 (alveolar macrophages-4)		Cluster 14 (mesenchymal cells)	
Gene symbol	Adjusted p values	Gene symbol	Adjusted p values	Gene symbol	Adjusted p values	Gene symbol	Adjusted p values	Gene symbol	Adjusted p values	Gene symbol	Adjusted p values	Gene symbol	Adjusted p values	Gene symbol	Adjusted p values	Gene symbol	Adjusted p values	Gene symbol	Adjusted p values	Gene symbol	Adjusted p values	Gene symbol	Adjusted p values	Gene symbol	Adjusted p values	Gene symbol	Adjusted p values
		ENOSF1	5.939E-26																								
		CNOT1	6.701E-26																								
		PRPF4B	9.078E-26																								
		PRMT2	1.12E-25																								
		HMGN2	1.126E-25																								
		PDIA4	1.148E-25																								
		SFPQ	1.246E-25																								
		ARGLU1	1.921E-25																								
		SOCS6	2.684E-25																								
		ATP13A3	2.977E-25																								
		PRKAA1	3.993E-25																								
		SIK1	4.788E-25																								
		CAND1	5.801E-25																								
		PNISR	8.201E-25																								
		MRPL40	9.429E-25																								
		LUC7L3	1E-24																								
		PDCD6	1.06E-24																								
		NUMA1	1.112E-24																								
		TLE4	1.323E-24																								
		PURA	1.46E-24																								
		EIF2AK1	1.883E-24																								
		UBA1	4.136E-24																								
		IRF2BP2	4.996E-24																								
		PIN1	5.356E-24																								
		EWSR1	1.191E-23																								
		SF3B2	4.441E-23																								
		PFDN6	4.828E-23																								
		ZBTB4	5.176E-23																								
		GSN	6.019E-23																								
		UHMK1	7.878E-23																								
		ROGDI	1.332E-22																								
		SKIV2L2	2.451E-22																								
		C4orf3	5.302E-22																								
		PUM1	5.333E-22																								
		PROS1	5.906E-22																								
		ST3GAL6	8.991E-22																								
		LONP2	1.087E-21																								
		ANKRD11	1.934E-21																								
		LYRM2	2.401E-21																								
		LRP10	3.222E-21																								
		TRIM44	3.605E-21																								
		VCP	3.621E-21																								
		HSPE1	3.919E-21																								
		PTBP3	4.019E-21																								
		AFTPH	5.188E-21																								
		CD164	7.312E-21																								
		RAD50	7.785E-21																								
		H1FX	8.458E-21																								
		TNKS2	8.898E-21																								
		TPR	1.04E-20																								
		FXR1	1.502E-20																								
		HNRNPM	1.525E-20																								
		HMGN1	1.626E-20																								
		SPG20	1.719E-20																								
		PHACTR2	3.832E-20																								
		SIVA1	4.049E-20																								
		ZCRB1	4.183E-20																								
		ZBTB7A	4.794E-20																								
		DYNLRB1	4.989E-20																								
		MYO6	5.884E-20																								
		RBM47	1.009E-19																								
		SHFM1	1.433E-19																								
		NDUFA5	1.704E-19																								
		SLC30A9	2.547E-19																								
		PDE4DIP	3.126E-19																								
		SF1	3.379E-19																								
		RAB11B	3.38E-19																								
		RP11-160E	3.516E-19																								
		HADH	3.681E-19																								
		TGIF1	4.632E-19																								
		KRT10	6.666E-19																								
		ASH1L	7.571E-19																								
		SYPL1	1.02E-18																								
		CPEB4	1.292E-18																								
		KHDRBS1	1.31E-18																								
		TUFM	1.439E-18																								
		PSMB5	1.874E-18																								
		PTTG1IP	3.892E-18																								

Cluster 1 (airway and alveolar epithelial cells)		Cluster 2 (ciliated cells)		Cluster 3 (type II alveolar cells-1)		Cluster 4 (type II alveolar cells-2)		Cluster 5 (type II alveolar cells-3)		Cluster 6 (immune cells)		Cluster 7 (plasma cells)		Cluster 8 (monocytes-1)		Cluster 9 (monocytes-2)		Cluster 10 (alveolar macrophages-1)		Cluster 11 (alveolar macrophages-2)		Cluster 12 (alveolar macrophages-3)		Cluster 13 (alveolar macrophages-4)		Cluster 14 (mesenchymal cells)	
Gene symbol	Adjusted p values	Gene symbol	Adjusted p values	Gene symbol	Adjusted p values	Gene symbol	Adjusted p values	Gene symbol	Adjusted p values	Gene symbol	Adjusted p values	Gene symbol	Adjusted p values	Gene symbol	Adjusted p values	Gene symbol	Adjusted p values	Gene symbol	Adjusted p values	Gene symbol	Adjusted p values	Gene symbol	Adjusted p values	Gene symbol	Adjusted p values	Gene symbol	Adjusted p values
		HNRNPUL	4.627E-18																								
		POLR2J3	8.927E-18																								
		PTRHD1	1.238E-17																								
		DDX5	1.477E-17																								
		WASF2	1.733E-17																								
		KIF5B	4.249E-17																								
		N4BP2L2	7.195E-17																								
		DNAJA1	7.981E-17																								
		MRPL41	2.447E-16																								
		SKP1	6.97E-16																								
		BUD31	1.45E-15																								
		MRPL57	1.557E-15																								
		ERH	6.914E-15																								
		UPF2	9.628E-15																								
		SMDT1	1.087E-14																								
		SRRM2	3.554E-14																								
		HSPD1	5.188E-14																								
		THRAP3	6.243E-14																								
		RUNX1	8.286E-14																								
		NDUFB7	1.555E-13																								
		PDK4	4.702E-13																								
		ROMO1	7.191E-13																								
		SSB	1.89E-12																								
		NPTN	1.901E-12																								
		CANX	2.129E-12																								
		CXCL1	9.015E-12																								
		SLTM	7.606E-11																								
		HSPB1	7.559E-10																								
		BTG2	9.589E-07																								
		EGR1	8.245E-06																								
		RHOB	1.135E-05																								

Supplemental Table III. Signature genes of the airway and alveolar epithelial cell populations[1] in human lung of control donors and IPF patients

Cluster 1 (basal cells)		Cluster 2 ($MUC5B^{+}$ club cells)		Cluster 3 ($SCGB3A2^{high}$ club cells)		Cluster 4 (type II alveolar cells)		Cluster 5 (type I alveolar cells-1)		Cluster 6 (type I alveolar cells-2)		Cluster 7 (type I alveolar cells-3)	
**Gene symbol**	**Adjusted p values**	**Gene symbol**	**Adjusted p values**	**Gene symbol**	**Adjusted p values**	**Gene symbol**	**Adjusted p values**	**Gene symbol**	**Adjusted p values**	**Gene symbol**	**Adjusted p values**	**Gene symbol**	**Adjusted p values**
S100A2	5.09E-230	BPIFB1	1.663E-92	SCGB3A2	9.99E-126	HLA-B	5.16E-180	AREG	5.936E-32	EMP2	2.28E-129	AGER	8.23E-144
KRT15	4.61E-219	SCGB3A1	2.431E-80	PIGR	6.95E-102	IGHG4	5.7E-159	NFKBIA	4.545E-25	AGER	2.11E-124	CAV1	1.98E-117
KRT5	2.59E-189	SAA1	4.229E-69	SFTPB	1.897E-86	SERPINA1	4.22E-158	HOPX	1.029E-18	CAV1	6.22E-100	RTKN2	1.69E-116
KRT17	2.01E-180	SAA2	1.022E-61	CYB5A	6.665E-85	IGKC	2.12E-157	SFTPC	1.104E-18	HOPX	6.818E-77	HOPX	8.77E-113
RPLP1	1.09E-173	WFDC2	1.771E-61	MGP	1.906E-79	CD74	8.85E-148	CXCL2	6.101E-18	RAB11FIP1	1.944E-73	SPOCK2	1.23E-109
RPL3	1.4E-151	MUC5B	4.55E-57	AGR3	7.459E-63	SLC34A2	5.31E-144	ZFP36	8.639E-18	CLDN18	9.286E-67	GGT1	1.32E-107
RPS18	7.72E-139	TMEM45A	2.156E-56	SCNN1B	1.423E-62	LAMP3	1.76E-140	XIST	6.963E-16	SPOCK2	2.009E-65	EMP2	2.398E-98
PTPRZ1	3.98E-136	LCN2	3.815E-55	SCGB1A1	6.459E-55	HLA-A	1.69E-139	CCND2	9.06E-16	KLF6	4.302E-59	KRT7	1.839E-96
RPS2	2.13E-126	LTF	8.858E-48	FABP5	1.009E-53	IGHG3	1.12E-137	TM4SF1	1.189E-14	RTKN2	5.586E-59	TSPAN13	1.009E-90
RPL10A	3.98E-126	CP	8.769E-41	MET	4.123E-53	NAPSA	4.7E-132	C4BPA	2.756E-14	DSTN	4.953E-55	SCEL	1.557E-87
RPL13A	3.81E-123	TSPAN8	1.573E-39	CLIC6	1.079E-50	HLA-DRA	5.57E-124	DBI	1.395E-13	SFTA2	2.221E-52	TIMP3	1.846E-87
IGFBP6	5.76E-123	SCGB1A1	5.599E-39	ARFGEF3	1.666E-47	NPC2	4.12E-122	NEAT1	7.556E-13	SCEL	1.39E-50	CAV2	7.145E-76
RPS8	1.81E-121	PIGR	1.188E-36	KIAA1324	2.339E-42	SFTPB	7.28E-112	TTN	2.334E-12	ANKRD29	1.048E-44	MYL9	1.543E-74
RPS6	5.14E-120	RARRES1	7.108E-36	ALDH1A1	7.206E-39	IGLC2	2.42E-111	SFTA2	3.021E-12	NUPR1	1.119E-44	NDRG1	3.617E-73
MMP1	2.11E-117	RHOV	1E-34	SCGB3A1	2.029E-37	AQP1	4.06E-109	FTL	1.17E-11	CAV2	1.12E-42	RAB11FIP1	5.219E-72
RPL41	3.15E-116	PLEKHS1	1.526E-34	CXCL17	9.986E-37	HLA-DPA1	6.84E-106	THBS1	2.146E-11	SEPP1	2.496E-42	NTM	7.06E-72
RPS4X	1.02E-114	PROM1	1.277E-29	MMP7	2.614E-36	CTSH	3.59E-105	PEBP4	2.548E-11	MYL9	4.948E-42	GGTLC1	1.454E-70
AQP3	4.32E-112	MSMB	2.66E-29	GRHL1	9.833E-33	DMBT1	1.116E-97	DUSP6	8.955E-11	ARHGEF2(	6.176E-42	S100A4	1.554E-70
COL17A1	7.24E-110	XBP1	4.163E-27	RNASE1	7.741E-32	NRGN	1.83E-97	PGC	1.444E-10	CLIC5	5.83E-41	LIMCH1	2.598E-70
RPS17	5.63E-107	AGR2	2.946E-26	C16orf89	9.209E-31	IGHG1	1.908E-97	GKN2	1.079E-09	COL4A2	5.72E-39	DENND3	1.446E-69
ALDH3A1	1.04E-105	TFF3	2.319E-24	SLPI	1.019E-30	HLA-C	4.912E-91	JUND	2.455E-09	PEBP4	1.684E-37	LAMA3	1.931E-69
TP63	1.2E-103	SLPI	4.904E-23	FAM129A	6.633E-30	C1S	9.057E-90	FGGY	2.612E-09	TSPAN13	3.846E-37	GPRC5A	4.187E-69
TRIM29	1.82E-102	RP11-355F	1.637E-22	FAM46C	2.943E-29	ETV1	1.038E-88	ADAMTS9	4.987E-09	PLA2G1B	5.644E-37	WFS1	5.912E-68
RPS9	6.69E-102	BPIFA1	1.797E-22	INHBB	1.271E-28	ADGRF1	1.054E-87	FOS	7.321E-09	LMO7	2.128E-36	ANKRD29	9.093E-68
RPS27	1.97E-100	ZG16B	3.441E-22	WFDC2	2.173E-28	HLA-DPB1	5.419E-86	GNAS	1.494E-08	NTM	2.5E-36	SFTA2	2.962E-64
RPL13	4.47E-100	CXCL1	6.268E-21	MUC4	1.544E-27	B2M	1.613E-82	HSD17B6	2.346E-08	ANOS1	5.585E-36	DAPK2	4.587E-64
SYT8	5E-100	GSTA1	2.093E-20	CRACR2B	1.782E-27	SERPIND1	3.549E-82	PDLIM3	2.53E-08	LDLR	3.024E-35	FAM105A	1.637E-62
RPL11	8.23E-100	GALNT6	5.064E-19	HLA-DRB5	6.024E-27	CXCL14	5.232E-81	GDF15	1.111E-07	CD55	6.582E-35	ITLN2	8.026E-61
GNB2L1	3.89E-99	MDK	2.436E-18	STEAP4	2.415E-26	SFTPA1	1.241E-80	ATP6V0E1	2.172E-07	SLC39A8	1.004E-34	ALCAM	2.077E-60
RPL10	1.697E-98	CYP2F1	3.117E-15	ERP27	9.972E-26	C1R	8.462E-80	LIMS1	2.755E-07	CYR61	1.009E-34	POLR2L	3.497E-60
RPS5	5.406E-98	TGM2	4.429E-15	CLU	3.051E-25	SLC5A8	1.626E-79	MGST1	2.966E-07	MAP2	2.714E-34	CST6	1.956E-59
RPS15	1.32E-97	ADAM28	1.106E-14	FMO2	2.624E-22	C3	7.466E-79	SDR16C5	3.011E-07	TACC2	1.972E-33	HPCAL1	2.882E-59
RPL4	1.414E-97	CAPN13	1.528E-14	HMGN3	3.249E-21	CHI3L1	9.019E-79	S100A10	4.002E-07	GPM6A	5.606E-33	SEMA3B	5.121E-59
RPS7	1.529E-97	CXCL6	2.033E-14	HNMT	5.827E-20	LRRK2	1.885E-78	PLA2G1B	4.503E-07	WWC2	6.153E-33	DUSP1	5.168E-59
RPL15	7.394E-97	LINC01207	4.352E-14	KLK11	7.59E-20	CXCL17	7.984E-77	MYL12B	4.72E-07	KRT7	1.082E-32	TNNC1	5.896E-58
RPL8	3.05E-96	TMC5	2.065E-13	NFIA	4.539E-19	DRAM1	8.731E-77	UQCRQ	4.766E-07	GJA1	1.211E-32	ANXA3	4.495E-57
BTF3	8.285E-95	CLU	6.017E-13	SLC4A5	5.779E-19	PARM1	7.355E-75	ATP5EP2	5.595E-07	FAM105A	3.613E-32	UNC13D	7.942E-57
RPS24	1.317E-94	ANPEP	6.077E-13	RP11-160E	2.696E-18	SOD2	1.604E-74	ITGB6	9.761E-07	DUSP1	4.783E-32	MYRF	1.446E-56
RPL32	9.383E-94	ANKRD36(	9.48E-13	TSC22D1	9.789E-18	MT-ND1	3.08E-74	CD63	1.088E-06	MSMO1	1.047E-31	SUSD2	2.288E-56
HNRNPA1	1.062E-93	PSCA	1.479E-12	PAG1	1.6E-17	AHCYL2	3.136E-71	TMEM97	1.388E-06	HPCAL1	1.553E-31	ABCA7	2.236E-54
RPL7	1.539E-92	KDR	3.408E-12	PVRL4	2.453E-17	NEAT1	3.487E-71	CALM1	1.625E-06	PLAC8	1.932E-31	ACTB	4.341E-54
RPS19	1.832E-92	ERN2	1.202E-11	S100A13	5.568E-17	PSAP	2.477E-70	CLDN18	2.026E-06	NEXN	9.433E-31	GOLGA8B	5.842E-54
DSC3	5.67E-92	C3	3.622E-11	LTA4H	2.014E-16	FTL	4.069E-69	BMP2	2.403E-06	VEGFA	1.44E-30	TMSB4X	6.547E-54
COL7A1	1.56E-90	CTSC	5.022E-11	LGALS3BP	1.165E-15	SFTPA2	1.034E-68	ANXA5	2.867E-06	DBI	1.634E-30	SPTBN1	1.723E-53
RPL29	1.723E-89	HS3ST1	5.385E-11	CP	1.233E-15	SFTPC	1.968E-68	SNX25	4.114E-06	S100A4	4.349E-30	SLC39A8	2.674E-53
RPL14	4.144E-89	KLK11	6.519E-11	ANPEP	4.084E-15	ALPL	1.297E-67	ZNF385B	5.465E-06	COL12A1	6.226E-30	MYL12A	2.715E-53
RPL19	6.037E-89	SAA4	6.958E-11	CYP2A6	4.691E-15	DPP4	2.739E-66	BCAT1	5.499E-06	WFS1	2.006E-29	COL12A1	3.911E-53
IL33	2.265E-88	S100A9	1.093E-10	AQP5	5.374E-15	TXNRD1	3.544E-64	KRT8	5.774E-06	JUND	2.101E-29	MAP2	2.149E-52
VSNL1	3.682E-88	MARCKSL	3.546E-10	PLCG2	8.952E-15	LCN2	7.066E-64	ODC1	5.815E-06	IDI1	1.781E-28	MYL6	8.392E-52
RPS28	7.856E-87	BMPR1B	4.539E-10	CLDN3	1.598E-14	TSPAN3	2.555E-63	NDUFA4	7.356E-06	COX17	3.341E-28	RGCC	2.496E-51
MT1X	3.334E-86	DUSP4	1.66E-09	PLPP2	1.818E-14	IGLC3	3.614E-63	DESI2	1.303E-05	CLIC3	4.538E-28	CD151	5.942E-51
RPL28	7.106E-86	ATP12A	2.018E-09	CD74	2.517E-14	LPCAT1	1.551E-61	SFTPD	2.193E-05	MYRF	8.533E-28	LMO7	1.285E-50
RPL18A	8.405E-86	VMO1	2.411E-09	SLC22A3	7.284E-14	CTSB	8.361E-61	ANXA3	2.272E-05	FAM177A1	9.641E-28	SFTA1P	2.755E-50
RPL27A	6.469E-83	DUSP23	2.414E-09	GSN	7.326E-14	SPINK1	9.578E-61	PEG10	2.766E-05	HMGCS1	1.267E-27	FLNA	1.363E-49
RPS23	2.396E-82	AQP5	2.998E-09	PTPN13	7.573E-14	RNF145	1.363E-59	AKAP13	2.786E-05	TNNC1	8.446E-27	AHNAK	4.102E-49
RPS27A	1.105E-81	HP	4.099E-09	EID1	1.239E-13	SLC6A14	8.141E-57	CKS2	3.911E-05	ATP5E	1.812E-26	UBC	9.371E-49
RPS3A	1.819E-80	PLK2	4.231E-09	IGLC3	1.455E-13	LGMN	1.812E-56	TXN	4.861E-05	GPRC5A	2.121E-26	CLIC3	1.702E-48
RPL12	1.216E-79	KYNU	4.406E-09	TMEM45A	1.55E-13	ABCA3	2.09E-56	TMSB4X	6.373E-05	QKI	5.014E-26	GSTP1	4.554E-48
MEG3	1.421E-79	TNFAIP2	7.327E-09	WWTR1	1.609E-13	TGM2	1.098E-55	ACAT2	6.841E-05	DAPK2	1.233E-25	ARHGEF2(	5.172E-48
SLC25A6	1.559E-79	GRAMD3	9.738E-09	ARHGDIB	3.338E-13	RASGRF1	3.92E-55	PMEPA1	8.136E-05	COX7A2	1.355E-25	PAPSS2	1.255E-47
RPL34	1.72E-78	FAM3D	1.014E-08	SNHG19	7.637E-13	SMPDL3A	1.148E-54	TUBA1B	0.0001149	COL8A1	1.395E-25	FSTL3	3.467E-47
RPS14	2.931E-78	TSPAN1	1.973E-08	APOD	1.208E-12	MUC1	4.818E-54	COL1A1	0.0001227	COL4A4	1.868E-25	COL4A3	3.666E-47
RPS3	4.595E-78	CPD	4.412E-08	ELF3	3.112E-12	TNIP3	6.703E-54	WIF1	0.0001269	LAMA3	3.055E-25	KLF6	4.911E-47
RPL26	7.619E-78	PLPP2	7.291E-08	GSTA1	3.177E-12	CD24	6.702E-53	TPM1	0.0001942	CFLAR	3.908E-25	ANKRD1	1.229E-46
RPL6	1.964E-77	GGT5	1.649E-07	NT5E	3.989E-12	CALR	1.222E-52	BMP1	0.0002018	LIMCH1	5.656E-25	PCYOX1	7.38E-46

Cluster 1 (basal cells)		Cluster 2 ($MUC5B^+$ club cells)		Cluster 3 ($SCGB3A2^{high}$ club cells)		Cluster 4 (type II alveolar cells)		Cluster 5 (type I alveolar cells-1)		Cluster 6 (type I alveolar cells-2)		Cluster 7 (type I alveolar cells-3)	
Gene symbol	Adjusted p values	Gene symbol	Adjusted p values	Gene symbol	Adjusted p values	Gene symbol	Adjusted p values	Gene symbol	Adjusted p values	Gene symbol	Adjusted p values	Gene symbol	Adjusted p values
GAPDH	2.182E-77	CD82	2.21E-07	SMIM22	4.064E-12	HLA-DRB1	2.217E-52	KLF6	0.0002214	TAGLN	1.56E-24	CFL2	7.438E-46
PKP1	2.543E-75	CXCL8	3.947E-07	HLA-DMA	4.384E-12	MICAL2	4.022E-52	RTN4	0.0002703	COL4A1	6.031E-24	GPM6A	8.521E-46
TNNI2	5.577E-75	INSR	4.78E-07	MRPS25	5.093E-12	PTP4A3	7.871E-51	LYZ	0.0002844	NDUFA4	1.324E-23	NUPR1	1.501E-45
NPM1	3.729E-74	FCGBP	9.032E-07	CYP1B1	6.923E-12	HIF1A	2.122E-50	CA2	0.0003172	AHNAK	1.453E-23	CYBRD1	2.082E-45
LPAR6	2.201E-71	NPDC1	1.061E-06	PDCD4	8.564E-12	RAB27B	3.313E-49	NPC2	0.0003619	SFTPC	2.486E-23	C19orf33	1.579E-44
RPS21	6.331E-71	MS4A8	1.14E-06	CAPN8	9.554E-12	MT-CO3	1.335E-48	FOSB	0.0003912	COL4A3	2.729E-23	COL4A2	2.355E-44
TNS4	7.929E-71	TMEM176/	1.31E-06	HSD17B13	1.198E-11	MT-ATP6	1.826E-48	CDH2	0.0004627	NECAB1	2.83E-23	NEBL	3.69E-44
RPL35A	9.173E-71	LY6E	1.796E-06	FOLR1	1.256E-11	CITED2	2.803E-48	S100A8	0.0004673	SDR16C5	4.884E-23	CD55	1.875E-43
RPS10	1.788E-70	S100P	2.18E-06	NOTCH3	1.827E-11	SMC4	2.864E-48	ARPC2	0.0007127	ANXA3	4.915E-23	MYO1C	2.933E-43
NGFR	4.513E-70	LINC00342	3.806E-06	SNHG25	3.172E-11	AGL	4.554E-48	FEM1C	0.0009826	VCL	5.835E-23	HIST1H1C	4.546E-43
ITGB4	1.964E-69	CYBA	8.647E-06	CDK14	7.097E-11	FBP1	1.786E-47	WSB1	0.0012905	C14orf132	1.043E-22	TIMP2	8.314E-43
RPL5	6.36E-69	CFH	1.424E-05	CTSE	7.911E-11	HLA-E	2.363E-47	DPYSL2	0.0014121	MS4A15	1.297E-22	USMG5	1.364E-41
PERP	7.3E-69	IRAK3	2.708E-05	MACROD2	8.8E-11	COLEC12	2.721E-47	CDKN1A	0.0020907	RGCC	1.333E-22	NEDD4L	1.717E-41
CHST9	2.068E-68	SERPINF1	3.207E-05	TTC3	9.211E-11	FN1	1.745E-46	CITED2	0.0021742	JUN	1.476E-22	ADIRF	1.836E-41
RPL36A	5.659E-68	TNFSF10	3.533E-05	RUNX2	3.446E-10	PDZK1IP1	4.706E-46	NNMT	0.0027083	ANKRD1	1.674E-22	C14orf132	1.926E-41
RPL36	1.179E-67	GLUL	4.875E-05	EZR	8.243E-10	HMCN1	6.683E-46	GALNT10	0.0029055	SESTD1	2.145E-22	TACC2	1.814E-39
NACA	4.363E-67	PODXL	5.208E-05	MSI2	8.92E-10	HLA-DOA	7.33E-46	CDC42EP3	0.003321	NCKAP5	2.456E-22	GAS6	3.334E-39
RPS25	4.601E-66	MUC4	6.614E-05	GRAMD3	1.142E-09	FCGR2A	9.768E-46	CPM	0.003906	NGFRAP1	2.717E-22	UPK3B	5.625E-39
RPL39	6.42E-66	IGLC2	6.742E-05	MS4A8	1.342E-09	C5	2.258E-45	PTGS2	0.004651	PEBP1	3.41E-22	COL4A1	7.088E-39
IGFBP7	1.473E-65	CYP24A1	7.337E-05	WNT7B	2.35E-09	MID1IP1	6.062E-45	NFKBIZ	0.0048423	SQLE	6.917E-22	MMP23B	7.636E-39
RPL35	2.375E-65	IGFBP3	9.859E-05	DNAH5	4.371E-09	ATP11A	1.091E-44	MACC1	0.0049909	DENND3	8.506E-22	TNS1	9.126E-39
RPL21	3.769E-64	DDIT4	0.0001089	PRSS12	5.394E-09	FGG	3.778E-44	SCD	0.0075918	CPB2	1.207E-21	DSTN	1.118E-38
RPL18	5.167E-64	CX3CL1	0.0001122	ABCC5	6.832E-09	EPS8	5.318E-44	RHOB	0.0108414	PAPSS2	1.228E-21	S100A6	1.186E-38
EEF1A1	9.631E-64	SORD	0.0001535	AADAC	1.399E-08	RASGRP1	1.597E-43	MALL	0.0123144	SLC1A1	1.392E-21	RNASE1	1.385E-38
SERPINF1	6.462E-63	FUT2	0.0001769	ZNF608	1.46E-08	LAP3	2.808E-43	MSLN	0.0123729	RTN4	4.421E-21	ID3	2.088E-38
RPL30	9.397E-63	CLDN10	0.0001798	ATL2	1.698E-08	SFTPD	5.291E-43	C11orf96	0.0141736	FSTL3	1.485E-20	ABCA1	6.503E-38
IGFBP2	2.08E-62	CHST9	0.0001991	MT-ND4L	2.23E-08	TFPI	5.911E-43	KRT18	0.0167226	SYT15	1.955E-20	UBL3	8.809E-38
GPX2	2.661E-62	LY6D	0.0002467	RUNX1	3.688E-08	C2	2E-42	MBIP	0.0203828	MYL6	5.232E-20	SLC1A1	1.005E-37
SERPINB5	3.551E-62	TSHZ2	0.000306	HLA-DRB1	4.82E-08	MT-ATP8	2.886E-42	ICAM1	0.0210542	ZNF385B	5.94E-20	TTLL7	1.122E-37
RPL7A	1.6E-61	VSTM2L	0.0003869	GOLM1	6.454E-08	APOE	3.668E-42	GEM	0.0247275	ATP5EP2	7.027E-20	PARD6B	5.28E-37
RPL23A	1.717E-61	HNMT	0.0012059	S100A14	1.09E-07	ST3GAL1	1.341E-41	EMC4	0.0319336	C1orf198	7.983E-20	HEG1	2.167E-36
RPL31	3.477E-61	CCNO	0.0012101	HSD17B11	1.613E-07	AASS	2.957E-41	FAM133B	0.0354786	GGTLC1	8.957E-20	TMEM160	2.321E-36
LIMA1	6.303E-61	AZGP1	0.0013073	CTSC	2.931E-07	SCD	5.071E-41	SQLE	0.0375439	RP1-78O14	9.458E-20	SESTD1	2.358E-35
LGALS7B	1.524E-60	B3GNT3	0.0014169	TRIM2	4.063E-07	P4HB	5.471E-41	PTRF	0.0421602	TGFBR2	1.177E-19	HMHA1	4.003E-35
DDIT4	2.406E-60	MMP7	0.0015359	NR4A1	4.928E-07	SLC22A31	8.384E-41	JUN	0.0476976	COX6C	1.506E-19	ADRB2	1.403E-34
ITGA2	2.881E-60	NAALADL	0.0016997	EPHX1	5.635E-07	SEZ6L2	5.245E-40			HSD17B6	1.761E-19	NPNT	2.443E-34
SNCG	7.222E-60	STEAP4	0.002588	CAPS	6.112E-07	HTRA1	6.373E-40			TTN	1.848E-19	MS4A15	1.845E-33
NSG1	1.595E-59	HLA-DRB5	0.0030635	GGT5	1.025E-06	GALNT1	6.544E-40			PEAK1	3.023E-19	PPP2R5A	3.289E-33
RPL37A	1.779E-59	FAM46C	0.0031519	MUC1	1.154E-06	NELL1	1.201E-39			CCDC141	4.363E-19	BLVRB	5.597E-33
RPS13	4.761E-58	IGHG3	0.0046008	SOX4	1.403E-06	CTSD	1.282E-39			SPTBN1	6.375E-19	NGFRAP1	3.078E-32
RPSA	5.775E-58	AKR1C1	0.0054433	IRX3	1.413E-06	CTSE	1.455E-39			PGC	7.101E-19	HSPG2	3.718E-32
ADH7	5.906E-58	SPDEF	0.0075731	MDK	1.872E-06	ATP10B	1.528E-39			ATF4	8.697E-19	NCKAP5	3.811E-32
TPPP3	1.565E-57	SIX1	0.0082492	EGR1	2.826E-06	GABRB2	1.259E-38			PLLP	1.144E-18	KRT19	3.88E-32
RPLP2	1.368E-55	RUNX2	0.0089835	BMPR1B	3.408E-06	SPP1	1.547E-38			TES	1.306E-18	ABCA8	4.182E-32
CD109	5.19E-55	CFB	0.01008	STT3B	4.916E-06	CCL18	1.604E-38			C12orf49	1.517E-18	CSRP1	5.105E-32
RPS15A	1.047E-54	KLK10	0.0124123	VIM	5.103E-06	HSD17B11	3.205E-38			MYL12A	1.785E-18	TACSTD2	1.016E-31
FHL2	1.113E-54	UAP1	0.0124307	DSP	5.734E-06	MTUS1	4.887E-38			ROR1	3.214E-18	OTUD1	1.026E-31
LINC01503	1.366E-53	SLC15A2	0.0124501	ST6GALN/	7.032E-06	HLA-DQA2	5.26E-38			TIMP2	5.732E-18	AKAP11	1.033E-31
FAT2	3.794E-53	NDRG2	0.0132532	SOX2	7.148E-06	RCAN2	5.579E-38			FAM167A	6.115E-18	STARD7	1.048E-31
RHOC	4.372E-53	ST6GALN/	0.0154778	SPINK5	7.406E-06	SNX30	6.324E-38			BLVRB	9.106E-18	CEACAM6	2.908E-31
FBLN1	1.957E-52	FMO2	0.0215146	LYNX1	7.635E-06	HLA-F	1.772E-37			JCHAIN	1.37E-17	EPB41L5	7.233E-31
PFDN5	3.851E-52	CYP4X1	0.0230676	IDH1	8.202E-06	MT-CYB	1.79E-37			CEACAM6	1.795E-17	TGFBR2	1.536E-30
CSTA	2.322E-51	SAMHD1	0.026929	VMP1	9.918E-06	LYZ	1.829E-37			HBEGF	2.204E-17	DLC1	1.603E-30
VAV3	4.994E-51	GOLM1	0.0325505	HLA-DQB1	2.619E-05	DHRS7	3.187E-37			PRKCZ	2.519E-17	ABI3BP	1.982E-30
ARL4D	8.985E-51	SLC4A7	0.0334743	SCPEP1	2.978E-05	TMBIM6	6.106E-37			TMSB4X	2.993E-17	ATOH8	2.011E-30
S100A11	2.982E-50	SLC44A4	0.0336872	PABPC1	4.688E-05	C8orf4	6.643E-37			ADIRF	2.999E-17	ATP5E	2.732E-30
IFI16	3.162E-50	EHF	0.0367817	LY6E	4.735E-05	RNF141	1.334E-36			EPB41L5	3.392E-17	CYR61	4.595E-30
GPR87	4.935E-50	LGALS9	0.0370939	SDC4	0.000119	ADGRF5	1.879E-36			USMG5	7.164E-17	LIN7A	5.274E-30
RGS12	3.033E-49	SUSD4	0.0375619	SLC22A31	0.0001191	HLA-G	4.372E-36			TXN	9.262E-17	NDST1	5.673E-30
MT2A	3.625E-49			KLK10	0.000183	BCAT1	4.597E-36			CYBRD1	9.904E-17	WWC2	5.745E-30
LGALS7	5.642E-49			ST8SIA4	0.0002035	KCNJ2	4.671E-36			CTGF	1.285E-16	MYO1B	5.811E-30
EEF1D	7.854E-49			ATP1B1	0.0002122	SAT1	5.414E-36			PTRF	1.735E-16	PDPN	6.47E-30
RP11-44F2	3.996E-48			IGFBP2	0.000282	C4BPA	6.722E-36			INSIG1	2.209E-16	SBDS	1.966E-29
EEF1B2	4.974E-48			ZFP36L1	0.000566	VSIG1	1.701E-35			ASAH1	3.205E-16	RP11-496I9	2.457E-29
TRPC6	5.41E-48			TRIM22	0.0005678	MALL	3.048E-35			DLC1	4.331E-16	FAM107B	2.519E-29
RPL9	1.37E-46			CYBA	0.0018106	DHCR24	9.805E-35			TMEM245	6.029E-16	CTNNBIP1	3.211E-29
PALMD	2.651E-46			MT-ND6	0.0038959	PCSK2	1.469E-34			IDS	6.055E-16	PLLP	7.529E-29

Cluster 1 (basal cells)		Cluster 2 (MUC5B$^+$ club cells)		Cluster 3 (SCGB3A2^{high} club cells)		Cluster 4 (type II alveolar cells)		Cluster 5 (type I alveolar cells-1)		Cluster 6 (type I alveolar cells-2)		Cluster 7 (type I alveolar cells-3)	
**Gene symbol**	**Adjusted p values**	**Gene symbol**	**Adjusted p values**	**Gene symbol**	**Adjusted p values**	**Gene symbol**	**Adjusted p values**	**Gene symbol**	**Adjusted p values**	**Gene symbol**	**Adjusted p values**	**Gene symbol**	**Adjusted p values**
KCNN4	3.14E-46			SERPINF1	0.0048932	REL	1.472E-34			STX11	6.342E-16	CLDN18	1E-28
LTBP4	5.518E-46			FNIP2	0.0051119	ARRDC3	3.336E-34			XIST	7.287E-16	EFEMP1	1.324E-28
FAU	6.483E-46			IGLC2	0.0162751	DAPP1	2.714E-33			TIMP3	8.471E-16	IFI27	2.195E-28
RPL37	9.812E-46					SERPINB1	1.221E-32			OTUD1	8.482E-16	S100A10	3.484E-28
SRGAP3	1.822E-44					TPT1	1.304E-32			BMP2	1.075E-15	EHD2	4.625E-28
PLCH2	5.343E-44					TOP1	1.387E-32			CADM1	1.155E-15	EPCAM	6.234E-28
ELN	1.374E-43					SYNE1	1.456E-32			TM4SF1	1.279E-15	FXYD3	7.685E-28
SUGCT	1.789E-43					HMOX1	6.374E-32			NEBL	1.537E-15	RNH1	9.494E-28
TNC	1.407E-42					STXBP1	1.209E-31			RAB32	1.896E-15	CYSTM1	1.052E-27
GLTSCR2	4.347E-42					FNIP2	1.355E-31			TMEM97	3.761E-15	PKDCC	1.201E-27
RPS16	6.167E-42					CLDN2	2.116E-31			CYP4B1	3.822E-15	MYL12B	1.482E-27
RPS4Y1	6.336E-42					SCTR	2.427E-31			SPRYD7	1.105E-14	MAP7D1	1.796E-27
SNAI2	1.204E-41					HSP90B1	5.502E-31			PIP5K1B	1.161E-14	COL8A1	2.065E-27
ZNF385A	2.166E-41					CSF3R	6.147E-31			AK1	1.237E-14	GJA1	2.183E-27
MDFI	2.823E-40					RPN2	7.943E-31			ESRRA	1.249E-14	SNX22	3.395E-27
ISYNA1	5.259E-40					KCNJ15	8.119E-31			CST6	1.506E-14	IGFBP7	3.437E-27
CEMIP	3.849E-39					HLA-DMA	1.668E-30			EPB41L3	1.749E-14	FADS3	4.255E-27
CCDC80	4.66E-39					C15orf48	2.034E-30			DCXR	1.775E-14	EDN1	5.483E-27
TMPRSS4	4.891E-39					AP1S2	5.534E-30			HEG1	1.852E-14	AMOTL2	5.483E-27
SNCA	6.485E-39					MYO6	8.126E-30			CDKL5	1.927E-14	EGLN2	5.565E-27
FXYD3	1.229E-38					MYO1G	1.044E-29			MAGI1	1.993E-14	RABAC1	6.309E-27
RPS12	1.347E-38					AC079630.	1.172E-29			GLS	2.951E-14	RARRES3	6.842E-27
SOX2	2.039E-38					SLC39A8	2.361E-29			LSS	4.375E-14	METRNL	9.075E-27
TINAGL1	6.575E-38					VWA1	7.018E-29			ACTG1	5.596E-14	TSTD1	1.002E-26
RASSF6	9.896E-38					GRN	7.325E-29			MLLT4	5.703E-14	CAPN2	1.219E-26
PTMA	1.214E-37					FAM20A	7.852E-29			HACD1	7.156E-14	CFLAR	1.841E-26
EEF2	4.476E-36					WARS	7.924E-29			ZNF431	7.811E-14	SEMA5A	1.904E-26
GCLC	1.163E-35					HLA-DQA1	9.413E-29			PLA2G16	8.51E-14	ABLIM1	2.003E-26
FAM118A	1.888E-35					RFTN1	5.332E-28			SDPR	8.751E-14	LAMB2	3.015E-26
TCF4	1.907E-35					TMEM163	6.909E-28			MGLL	9.361E-14	C1orf198	3.246E-26
EIF3E	3.638E-35					SLCO2A1	1.17E-27			TTLL7	1.064E-13	ALDH3B1	3.268E-26
ZNF90	4.464E-35					HK2	1.445E-27			ABLIM1	1.217E-13	IL18	3.395E-26
DKK3	5.821E-35					PON2	2.436E-27			TACC1	1.428E-13	DST	4.515E-26
SPRY1	6.646E-35					MT-ND2	2.485E-27			OSGIN2	1.634E-13	AAMDC	4.541E-26
UNC5B-AS	7.95E-35					SEL1L3	2.566E-27			SNX22	1.882E-13	C1orf116	4.656E-26
EYA2	2.433E-34					CTSS	3.43E-27			P3H2	1.986E-13	SLC40A1	4.835E-26
FGFR3	5.395E-34					C8orf34-AS	6.299E-27			HCFC1R1	2.46E-13	RDX	4.972E-26
SPOCK3	1.349E-33					RP11-1143	6.638E-27			LIMS2	2.715E-13	NDUFB7	5.448E-26
SULF2	1.656E-33					QSOX1	6.799E-27			HMGCR	2.837E-13	BRI3	5.71E-26
TMEM237	4.383E-33					GNAS	7.823E-27			BRI3	3.063E-13	SULT1A1	1.115E-25
IKBIP	1.194E-32					CTSZ	9.068E-27			TMOD3	3.134E-13	SPINT1	2.067E-25
A4GALT	1.228E-32					FAM210B	1.147E-26			EHD2	3.158E-13	MGLL	2.189E-25
SH3BGRL3	3.43E-32					DUSP6	1.514E-26			CYSTM1	3.186E-13	ACTG1	2.705E-25
TSKU	6.147E-32					PLD3	4.387E-26			ALCAM	3.37E-13	PROS1	4.325E-25
ITGB8	6.25E-32					OLFM1	1.099E-25			MARC2	3.42E-13	ALDH3A2	4.436E-25
FBXO32	9.239E-32					STAT1	1.517E-25			PKDCC	3.484E-13	SYNPO	4.731E-25
HES1	9.646E-32					GPNMB	3.736E-25			S100A6	4.753E-13	TNFRSF12.	4.734E-25
NBEAL1	2.147E-31					ADGRE5	3.781E-25			ACTB	5.632E-13	TMEM125	5.746E-25
KCTD1	1.128E-30					SGK1	4.522E-25			PEG10	6.66E-13	LINC00152	8.206E-25
TSC22D1	1.179E-30					BACE2	5.076E-25			POLR2L	6.804E-13	RRAS	8.56E-25
CAPS	1.312E-30					HLA-DQB1	1.77E-24			FERMT2	7.924E-13	EIF1	2.119E-24
SOX9	1.501E-30					ANKRD50	3.417E-24			AKAP13	1.041E-12	CARHSP1	2.512E-24
RPL24	2.564E-30					PPP2R5C	4.149E-24			MRPS36	1.449E-12	AGTPBP1	3.144E-24
RPS29	3.863E-30					TMEM164	1.222E-23			HHIP	1.858E-12	GRK5	3.51E-24
CLDN1	6.43E-30					TYMP	1.789E-23			CXADR	1.957E-12	RARRES2	5.138E-24
CYP24A1	9.912E-30					ROS1	2.192E-23			PTPN1	2.522E-12	PRSS8	6.35E-24
RPL22	2.685E-29					SIAE	2.873E-23			SLCO4C1	2.663E-12	PNPLA2	7.534E-24
DST	1.266E-28					CPM	3.061E-23			CA2	2.978E-12	CH17-360D	8.313E-24
PAX9	1.992E-28					APOL6	3.927E-23			ACAT2	2.981E-12	LGALS1	2.235E-23
SYTL1	2.036E-28					MFSD2A	4.354E-23			LINC00891	2.998E-12	IL32	2.853E-23
GAMT	9.353E-28					TRAM1	4.423E-23			MSLN	3.199E-12	PRDX1	4.002E-23
JAG2	1.154E-27					ASPH	5.405E-23			SBDS	3.829E-12	IRX2	4.318E-23
RPL22L1	1.168E-27					ITGB6	7.716E-23			STX2	4.119E-12	MPC1	4.353E-23
TP53TG1	1.5E-27					MUC21	9.063E-23			ADRB2	4.199E-12	RHOBTB3	5.636E-23
LTB4R	1.723E-27					NNMT	9.131E-23			ATP1B3	5.237E-12	FIS1	9.402E-23
RPLP0	3.516E-27					PSMD14	1.126E-22			RDX	5.788E-12	CHP1	1.001E-22
ITM2B	6.398E-27					TPP1	1.363E-22			RRAS	7.035E-12	VAMP8	1.157E-22

Cluster 1 (basal cells)		Cluster 2 (MUC5B^{+} club cells)		Cluster 3 (SCGB3A2^{high} club cells)		Cluster 4 (type II alveolar cells)		Cluster 5 (type I alveolar cells-1)		Cluster 6 (type I alveolar cells-2)		Cluster 7 (type I alveolar cells-3)	
Gene symbol	Adjusted p values	Gene symbol	Adjusted p values	Gene symbol	Adjusted p values	Gene symbol	Adjusted p values	Gene symbol	Adjusted p values	Gene symbol	Adjusted p values	Gene symbol	Adjusted p values
AR	8.997E-27					FGL2	2.98E-22			CFL2	7.382E-12	CAPNS1	1.18E-22
ETS2	2.552E-26					NDNF	3.837E-22			UNC13D	7.599E-12	MIR4435-2	1.407E-22
UBA52	3.19E-26					DPYSL2	4.614E-22			ZBED2	9.565E-12	MLLT4	1.468E-22
CLCA2	4.208E-26					ST6GAL1	4.657E-22			LL22NC03-	1.336E-11	CKB	1.658E-22
RPL38	4.909E-26					RRBP1	5.171E-22			NDRG1	1.451E-11	MRPL14	1.942E-22
RHBDL1	1.485E-25					ACSS2	5.178E-22			PHLDB2	1.86E-11	RAB32	2.355E-22
LPCAT2	2.289E-25					ST8SIA4	6.849E-22			SSFA2	2.199E-11	PRKCZ	2.467E-22
POSTN	2.918E-25					PGK1	9.008E-22			MYL12B	2.404E-11	LLGL2	3.827E-22
GPC1	3.077E-25					IFI6	9.138E-22			ID4	2.445E-11	FAM63B	4.106E-22
RP11-1275I	5.076E-25					SFN	1.838E-21			PCYOX1	2.876E-11	HEBP1	4.18E-22
PCDH7	5.359E-25					BCYRN1	1.944E-21			CLIC2	3.231E-11	ST6GALN/	5.235E-22
CIRBP	7.469E-25					TLR2	2.007E-21			CD47	3.382E-11	VEGFA	5.271E-22
PVRL1	1.207E-24					CYBB	2.467E-21			EDN1	6.925E-11	ARAP2	5.908E-22
RAB7B	1.344E-24					EMB	5.49E-21			CD9	7.188E-11	PTPN21	7.441E-22
MT1E	2.803E-24					CADM1	7.12E-21			PXDC1	7.996E-11	QKI	9.238E-22
KRT6A	2.819E-24					WIF1	1.142E-20			NDUFB2	9.103E-11	CRYAB	9.9E-22
COMMD6	2.998E-24					APOC1	1.16E-20			SCD	9.414E-11	C5orf38	1.012E-21
BASP1	1.342E-23					RND1	2.134E-20			LGALS3	1.067E-10	PLS3	1.062E-21
UQCRH	2.856E-23					ACTR2	2.157E-20			APLP2	1.118E-10	INF2	1.404E-21
HSPA4L	4.794E-23					ARPC1B	2.456E-20			SLC2A3	1.283E-10	MAP1LC3/	3.362E-21
FERMT1	7.094E-23					SECISBP2I	3.391E-20			CGNL1	1.971E-10	PLCXD2	3.458E-21
MARCKS	1.596E-22					ITGB2	3.624E-20			UBC	2.042E-10	HBEGF	3.798E-21
KRT19	3.415E-22					TIMP1	4.032E-20			ELOVL5	2.267E-10	EPS8L2	4.216E-21
DGKH	4.643E-22					GDE1	4.283E-20			EBP	2.635E-10	DYNLRB1	4.219E-21
BCL11A	7.387E-22					PABPC4	4.781E-20			SFTPD	2.684E-10	FMO2	4.82E-21
HMGN1	9.287E-22					C4orf48	4.783E-20			TJP2	2.985E-10	FLNB	4.958E-21
NACA2	1.021E-21					LDHA	5.153E-20			MGST1	3.09E-10	DDT	4.996E-21
TPM2	1.345E-21					REEP3	5.486E-20			MITF	3.236E-10	FOLR1	1.103E-20
EIF3F	1.608E-21					CLTC	5.662E-20			PFKFB2	3.483E-10	APLP2	1.153E-20
PAQR7	1.65E-21					CD63	5.863E-20			CYP51A1	3.843E-10	PLEKHJ1	1.375E-20
EIF3K	1.975E-21					TMEM173	9.163E-20			CACNA2D	4.469E-10	ACADL	1.478E-20
ID1	2.285E-21					TNFSF10	1.814E-19			SEMA5A	5.088E-10	LAMA5	1.551E-20
H3F3B	5.567E-21					SLC6A20	1.954E-19			CAPN2	6.909E-10	NEXN	1.593E-20
SFN	5.888E-21					SRGN	2.236E-19			CAT	7.151E-10	ID4	1.639E-20
G0S2	9.387E-21					TYROBP	2.611E-19			NPNT	7.449E-10	CCDC107	1.701E-20
SNRPD2	9.494E-21					TANC2	3.202E-19			APBB2	8.214E-10	C12orf49	1.888E-20
ATP5G2	9.741E-21					TRIM8	3.964E-19			UBL3	1.001E-09	AATK	2.071E-20
RPL27	1.13E-20					CFAP221	8.14E-19			FGGY	1.015E-09	H3F3B	2.391E-20
IFITM3	1.169E-20					MT-ND4	8.148E-19			NRG1	1.058E-09	MRPL55	2.758E-20
NDUFA8	2.475E-20					GLS	1.055E-18			KPNA3	1.116E-09	TBC1D2	3.037E-20
PLP2	2.52E-20					LYN	1.13E-18			FASN	1.202E-09	SPTSSA	3.293E-20
RP11-12G1	2.986E-20					ZMAT3	1.329E-18			CGN	1.22E-09	NDUFA3	3.589E-20
TPBG	3.717E-20					AGR2	1.516E-18			TUBA1A	1.396E-09	TMEM141	4.004E-20
DCN	4.001E-20					ACAD8	2.524E-18			FOS	1.447E-09	MTCH1	5.379E-20
TNFRSF21	8.266E-20					C16orf89	2.577E-18			CRYAB	1.453E-09	RBP1	8.042E-20
THOC3	9.319E-20					LPL	2.706E-18			MLPH	1.635E-09	CLIC5	9.618E-20
PYCARD	1.241E-19					PDIA3	2.712E-18			NR2F6	2.052E-09	ZFYVE9	1.262E-19
FAF1	1.244E-19					LCP1	3.505E-18			THOC2	2.334E-09	SCAI	1.289E-19
DUSP23	1.39E-19					HSPA8	4.163E-18			EPDR1	2.434E-09	VSIG2	1.308E-19
RP11-160E	1.56E-19					SLC46A2	4.497E-18			THSD7A	2.717E-09	UTRN	1.344E-19
LY6E	1.582E-19					MLEC	5.471E-18			IFI27	2.941E-09	RASSF7	2.013E-19
PDLIM1	1.759E-19					TAPBP	6.043E-18			PLS3	3.007E-09	NXN	3.38E-19
ST6GALN/	1.807E-19					CD44	6.357E-18			ABCA7	3.054E-09	COX17	3.411E-19
CALD1	2.138E-19					SEC11C	6.529E-18			PDGFA	3.665E-09	FAM167A	4.147E-19
TUSC3	2.557E-19					HLA-DMB	8.12E-18			ICAM1	4.412E-09	ID1	4.76E-19
TIMP1	4.322E-19					MDM2	1.3E-17			ARL5A	4.423E-09	ANOS1	5.009E-19
RAB4A	4.698E-19					CASK	2.37E-17			DPYSL2	5.465E-09	STX7	5.682E-19
EIF3L	6.124E-19					ACO1	2.697E-17			RNF128	6.036E-09	SPRYD7	6.884E-19
PHLDA3	7.018E-19					ALOX15B	2.739E-17			NFKBIA	6.121E-09	PPFIBP1	8.479E-19
S100A14	1.457E-18					GNS	2.94E-17			ATP1A1	6.122E-09	PDXK	9.736E-19
NT5C	1.5E-18					ACSL4	3.123E-17			LAPTM4B	6.261E-09	NDUFB2	9.857E-19
PDLIM4	1.571E-18					LMAN1	3.971E-17			AQP4	7.065E-09	EFNA1	1.018E-18
TMA7	1.829E-18					EML4	6.185E-17			LRRC16A	7.151E-09	MEAF6	1.396E-18
SERBP1	2.655E-18					TMC5	1.109E-16			RHOA	7.494E-09	CADM1	2.142E-18
C6orf48	8.567E-18					RRM2B	1.351E-16			ATF3	7.553E-09	SPARC	2.155E-18
CYBA	9.689E-18					SLC16A7	1.522E-16			EGFR	7.734E-09	EHD1	2.184E-18
CLSTN1	1.447E-17					TAP1	1.54E-16			DNAJB1	8.157E-09	METTL7A	2.29E-18

Cluster 1 (basal cells)		Cluster 2 (MUC5B^{+} club cells)		Cluster 3 (SCGB3A2^{high} club cells)		Cluster 4 (type II alveolar cells)		Cluster 5 (type I alveolar cells-1)		Cluster 6 (type I alveolar cells-2)		Cluster 7 (type I alveolar cells-3)	
**Gene symbol**	**Adjusted p values**	**Gene symbol**	**Adjusted p values**	**Gene symbol**	**Adjusted p values**	**Gene symbol**	**Adjusted p values**	**Gene symbol**	**Adjusted p values**	**Gene symbol**	**Adjusted p values**	**Gene symbol**	**Adjusted p values**
BLCAP	1.813E-17					STEAP4	1.67E-16			USP13	8.236E-09	SDPR	2.447E-18
EPB41L4A-	3.883E-17					SQRDL	1.73E-16			ESAM	8.484E-09	DAPK3	2.66E-18
GLIS3	4.145E-17					TBC1D24	1.742E-16			NEDD9	8.652E-09	CACNA2D	4.161E-18
BEST1	4.738E-17					SLC44A4	2.89E-16			CCDC107	1.095E-08	TMEM109	4.201E-18
C9orf3	5.626E-17					DAB2	3.378E-16			LIMD1	1.127E-08	CD47	4.749E-18
PIK3R1	6.539E-17					CTSA	4.041E-16			FZD5	1.146E-08	F11R	5.676E-18
IMPDH2	7.036E-17					BCL6	4.717E-16			UTRN	1.154E-08	TMSB10	6.095E-18
ADH1C	7.361E-17					FASN	4.806E-16			CDKN2B	1.318E-08	SSFA2	6.142E-18
NUDT4	1.271E-16					FUT8	5.118E-16			FAM63B	2.221E-08	HINT2	6.261E-18
SRSF5	1.545E-16					GGCX	5.4E-16			CD151	2.313E-08	NEDD8	6.989E-18
SIX1	2.853E-16					C20orf24	5.651E-16			TACSTD2	3.095E-08	GABARAP	7.32E-18
COL4A5	3.363E-16					RBPMS	6.257E-16			SPOP	3.354E-08	TES	9.383E-18
ITGA6	3.422E-16					NFIX	6.961E-16			DDAH1	3.397E-08	TSPAN4	1.054E-17
COX4I1	3.643E-16					CASP4	7.405E-16			LLGL2	3.4E-08	PTTG1IP	1.456E-17
BCAM	6.737E-16					ATP9A	7.777E-16			SLPI	4.284E-08	TAGLN2	1.548E-17
MMP14	7.727E-16					SLC12A2	8.967E-16			SPRED1	4.478E-08	SLC25A24	1.613E-17
NOTCH1	8.966E-16					PDIA4	9.487E-16			FDPS	4.698E-08	PLAC8	2.226E-17
GPC3	1.008E-15					SOD3	1.355E-15			PTPRM	4.73E-08	PEF1	2.739E-17
HIGD2A	1.526E-15					PAPPA	1.369E-15			DHCR24	5.207E-08	HSPA1B	2.822E-17
CDH3	1.639E-15					ACVR1B	1.396E-15			ABI3BP	5.349E-08	LAMTOR2	3.132E-17
SUMO2	1.656E-15					PARP14	1.412E-15			DESI2	6.338E-08	SCUBE1	4.069E-17
GOLGA8A	1.748E-15					PAM	1.791E-15			PARD6B	6.916E-08	FLCN	5.162E-17
LRP1	2.947E-15					HDGF	1.875E-15			MMP28	7.213E-08	MRPL41	5.425E-17
FBL	3.485E-15					FCER1G	1.902E-15			CCSER2	7.593E-08	MPP5	5.447E-17
SSBP2	4.952E-15					RBM47	2.151E-15			PRDX5	8.083E-08	CCDC84	6.691E-17
PROM2	5.418E-15					HSD17B12	2.325E-15			FMO5	1.012E-07	COX6A1	7.77E-17
TMEM123	5.458E-15					MTDH	2.384E-15			PTPN21	1.15E-07	PLA2G16	8.254E-17
SERPINB1	5.727E-15					CANX	2.878E-15			VEPH1	1.195E-07	IL17RE	9.731E-17
TSPAN1	7.076E-15					CD46	3.746E-15			MYH9	1.438E-07	TJP1	1.061E-16
IGKC	9.881E-15					C6orf62	4.104E-15			METRNL	1.511E-07	CALM2	1.089E-16
CFH	1.133E-14					NTN4	4.675E-15			RASSF7	1.539E-07	SLC2A3	1.093E-16
AC090498.	1.566E-14					SLC31A1	5.223E-15			PGRMC2	1.541E-07	COX6C	1.143E-16
CLNS1A	1.618E-14					ANXA4	7.261E-15			ZFP36	1.607E-07	FAM177A1	1.416E-16
EIF4A2	1.999E-14					RPN1	7.415E-15			PLA2G10	1.806E-07	LGALS3	1.453E-16
RAB34	2.061E-14					MBOAT7	7.626E-15			HSPB1	2.253E-07	LAMB3	1.628E-16
HINT1	2.731E-14					PHLDA1	8.37E-15			TMED5	2.345E-07	NRBP2	1.647E-16
SNHG8	3.629E-14					UCP2	1.18E-14			MICA	2.452E-07	WASF3	1.783E-16
PDCD4	1.747E-13					NFKB1	1.509E-14			SCNN1A	2.493E-07	HSPA1A	2.116E-16
MT-ND4L	1.839E-13					CREB3L1	2.041E-14			PPFIBP1	2.501E-07	TTC37	2.133E-16
TRIAP1	2.513E-13					ZDHHC9	2.047E-14			CHPT1	2.604E-07	PHACTR2	2.223E-16
BLOC1S2	2.555E-13					PDIA6	2.453E-14			DUSP3	2.685E-07	PLEKHA1	2.571E-16
XPR1	3.203E-13					SORBS1	2.502E-14			TEAD1	3.014E-07	PHLDB2	3.125E-16
ALDH7A1	3.244E-13					NCOA4	4.591E-14			FHL1	3.373E-07	WNT9A	3.771E-16
GSTK1	4.948E-13					SLFN5	4.88E-14			CEBPD	3.561E-07	ANKS1A	3.854E-16
EIF3D	7.337E-13					KIAA1324L	5.672E-14			SLC25A4	3.591E-07	SBSPON	4.886E-16
EIF3G	1.206E-12					BTG3	6.005E-14			BMP1	3.675E-07	RPS19BP1	5.952E-16
YBX1	1.313E-12					METAP2	6.32E-14			ATP5J2	4.508E-07	C1orf167	7.46E-16
RHOD	2.002E-12					ATP6V1A	6.383E-14			SDCBP	4.858E-07	SERINC1	7.623E-16
DSP	2.149E-12					EIF2S2	6.654E-14			SNAP23	6.694E-07	DNM2	8.613E-16
SORL1	3.65E-12					CMB9-22P	7.459E-14			OCLN	7.06E-07	C10orf54	8.996E-16
LAMB3	4.866E-12					AIF1	8.867E-14			PLXNA1	7.162E-07	CRK	9.167E-16
WIPI1	5.922E-12					GAREM1	1.115E-13			C5orf24	7.541E-07	IFI27L1	9.772E-16
LMO4	6.49E-12					RNF213	1.134E-13			FADS3	7.668E-07	C15orf52	1.039E-15
NDFIP2	7.003E-12					COTL1	1.979E-13			PRKAR1A	7.754E-07	UBTD1	1.092E-15
PLXNB1	1.017E-11					NUCB2	2.345E-13			GOLPH3	8.453E-07	CELF2	1.468E-15
SEPW1	1.332E-11					TRIM2	2.769E-13			MVD	1.03E-06	PER1	1.533E-15
LSM2	1.534E-11					MSN	2.913E-13			CUX1	1.036E-06	MPRIP	1.571E-15
AHCY	1.803E-11					TMPRSS2	2.962E-13			TIMM44	1.081E-06	MVB12B	2.03E-15
PRNP	1.819E-11					SUSD6	3.089E-13			CPEB2	1.119E-06	PPP1R12C	2.746E-15
IER3	2.158E-11					CPD	3.338E-13			FAM174B	1.211E-06	USP54	2.757E-15
UBE2E3	2.187E-11					CNIH1	3.583E-13			AMOTL2	1.221E-06	LINC00162	2.883E-15
LSM6	2.274E-11					ZDHHC21	3.934E-13			NFATC3	1.265E-06	BDNF	2.975E-15
TNFRSF19	2.606E-11					FTH1	4.045E-13			MUC15	1.317E-06	DAB2IP	3.062E-15
FAM213A	7.525E-11					RDH11	4.403E-13			FAM46B	1.378E-06	WBP2	3.112E-15
RMDN2	8.364E-11					CDH3	4.587E-13			SEMA3B	1.648E-06	TMEM139	3.156E-15
OCIAD2	9.158E-11					TMEM63B	5.217E-13			SLC27A3	1.968E-06	GNAQ	3.507E-15
CD99	9.303E-11					XBP1	7.179E-13			DST	1.99E-06	MATN3	3.971E-15

Cluster 1 (basal cells)		Cluster 2 (MUC5B$^+$ club cells)		Cluster 3 (SCGB3A2^{high} club cells)		Cluster 4 (type II alveolar cells)		Cluster 5 (type I alveolar cells-1)		Cluster 6 (type I alveolar cells-2)		Cluster 7 (type I alveolar cells-3)	
**Gene symbol**	**Adjusted p values**	**Gene symbol**	**Adjusted p values**	**Gene symbol**	**Adjusted p values**	**Gene symbol**	**Adjusted p values**	**Gene symbol**	**Adjusted p values**	**Gene symbol**	**Adjusted p values**	**Gene symbol**	**Adjusted p values**
RSL1D1	1.278E-10					IGFBP4	8.287E-13			CEBPG	2.047E-06	CCND3	4.054E-15
GLUL	1.285E-10					GK	1.098E-12			C4orf3	2.693E-06	MMP24-AS	5.001E-15
HN1	1.301E-10					EIF4E2	1.203E-12			SPTSSA	3.079E-06	ACOX1	5.192E-15
EIF2A	1.415E-10					MAGT1	1.262E-12			LAMC1	3.353E-06	PTPRF	6.141E-15
ZFAS1	1.971E-10					POR	1.403E-12			ZC3H12C	3.503E-06	FAM127A	6.203E-15
TXNIP	2.203E-10					EMC10	1.415E-12			CKB	3.604E-06	RBMS3	7.584E-15
METTL9	2.626E-10					HSPA5	1.452E-12			RHOF	3.97E-06	KRT18	7.628E-15
GPX4	2.794E-10					CEP70	1.68E-12			ARPC2	4.045E-06	MRPL57	8.366E-15
HOOK2	2.823E-10					NRP2	1.891E-12			ERGIC1	4.322E-06	PTPN1	8.529E-15
SCPEP1	3.416E-10					ENO1	1.915E-12			PDP2	4.351E-06	PDCD6IP	9.632E-15
S100A16	4.302E-10					CCDC69	2.131E-12			DDIT3	4.357E-06	NDUFAF3	1.014E-14
TAX1BP3	4.711E-10					COL1A1	2.533E-12			ALDH3B1	5.173E-06	PDLIM2	1.038E-14
BRK1	6.529E-10					ARL6IP5	2.984E-12			FDFT1	5.493E-06	ITGA3	1.106E-14
APP	7.407E-10					RAB5B	3.432E-12			GNAS	5.791E-06	FARP1	1.26E-14
RSRP1	8.687E-10					RDH10	3.47E-12			MAL2	5.828E-06	CCNY	1.265E-14
HMGB3	1.062E-09					GPX1	4.578E-12			S100A10	5.934E-06	KPNA3	1.319E-14
PCNP	1.208E-09					SAR1A	1.662E-11			ZNF493	5.981E-06	MPP3	1.541E-14
C17orf89	1.237E-09					WWC3	1.762E-11			SHTN1	6.061E-06	SEPT10	1.989E-14
TMEM173	1.288E-09					SEPW1	1.833E-11			SUSD2	6.1E-06	KCNT2	2.023E-14
NME1	1.312E-09					SURF4	2.019E-11			ATP2B4	6.303E-06	MRPS36	2.124E-14
SEPT9	1.352E-09					CKS2	2.196E-11			JADE1	6.423E-06	PRDX5	2.212E-14
ST13	2.245E-09					ANP32E	2.644E-11			CDH1	7.7E-06	TALDO1	2.374E-14
UQCRB	2.318E-09					CMTM6	2.845E-11			RASSF8	7.848E-06	MGAT3	2.621E-14
SERPINB3	2.627E-09					GBP3	3.53E-11			SH3BP5	7.964E-06	DUSP3	3.041E-14
UQCRC1	2.878E-09					LRP10	3.558E-11			PDXK	8.752E-06	NDUFA2	3.055E-14
NHP2	3.032E-09					GABARAP	3.782E-11			FILIP1	9.35E-06	ISG20	3.073E-14
TGIF1	3.918E-09					FAM120A	4.11E-11			SGCE	9.923E-06	SELENBP1	3.196E-14
SUMO1	4.787E-09					PPP1R12B	4.603E-11			CRIP1	1.093E-05	NDUFS7	3.247E-14
TMEM134	5.967E-09					PTP4A2	5.196E-11			STX3	1.108E-05	DNAJB2	3.324E-14
IGBP1	6.46E-09					SMIM14	5.878E-11			DUSP18	1.126E-05	EPB41L3	4.075E-14
EPPK1	7.34E-09					ATP1B1	6.208E-11			LATS2	1.145E-05	DUOX1	4.126E-14
SHFM1	7.637E-09					HERPUD1	7.601E-11			NCOA7	1.147E-05	LAMC2	4.273E-14
STMN1	1.026E-08					CD38	7.875E-11			TJP1	1.27E-05	TP53BP2	4.664E-14
C9orf16	1.187E-08					NAV2	8.959E-11			ARPC5	1.466E-05	NIPSNAP3	4.702E-14
KRT13	1.469E-08					RPS27L	1.037E-10			C1QB	1.515E-05	SLC19A3	4.756E-14
CLU	1.908E-08					CALU	1.902E-10			GPD1L	1.565E-05	FHL1	4.811E-14
UXT	1.977E-08					ERLEC1	2.198E-10			FHDC1	1.566E-05	ALS2CL	4.822E-14
MZT2A	1.978E-08					LIPA	2.236E-10			RHOBTB2	1.712E-05	PEAK1	5.099E-14
FAM3C	2.758E-08					IL6ST	2.616E-10			ATP13A4	1.712E-05	CCDC12	5.682E-14
BEX4	2.879E-08					HYOU1	2.904E-10			ZNF704	1.859E-05	ZDHHC12	5.933E-14
PLEC	3.66E-08					ARF4	4.167E-10			ITGB1	2.104E-05	ST3GAL4	6.168E-14
EGFR	5.446E-08					TMED2	4.842E-10			TGFBR3	2.112E-05	PFKFB3	6.861E-14
GSTO1	6.196E-08					ITPR2	1.013E-09			BNIP3	2.123E-05	HCFC1R1	7.257E-14
EFNA5	6.503E-08					INTS6	1.667E-09			RPS6KA2	2.194E-05	CACNG4	8.48E-14
BAG1	8.467E-08					KPNB1	1.803E-09			ST3GAL5	2.294E-05	NEDD9	9.099E-14
RPS6KA5	8.927E-08					TMEM30A	1.963E-09			ARPP19	2.478E-05	SMPD1	1.078E-13
PKM	9.182E-08					LAPTM5	3.111E-09			STK17A	2.665E-05	SMYD2	1.105E-13
LDHB	1.155E-07					CDC42EP1	3.244E-09			HMHA1	2.674E-05	GAB1	1.159E-13
CNBP	1.615E-07					BIRC3	3.654E-09			PAM	2.753E-05	MYO1D	1.174E-13
FOXP1	2.626E-07					DNAJC3	7.778E-09			LPIN1	2.793E-05	SCNN1A	1.247E-13
APRT	3.012E-07					DNAJC21	8.096E-09			EHD1	3.285E-05	LEPROT	1.291E-13
SRSF2	3.236E-07					VIM	8.249E-09			SLC3A2	3.301E-05	VKORC1	1.383E-13
WASF2	3.52E-07					SDF2L1	9.932E-09			FAM198B	3.332E-05	TEAD1	1.53E-13
FGFBP1	3.768E-07					IRX3	1.673E-08			CYB5R3	3.433E-05	TOR1AIP2	1.618E-13
EHF	4.195E-07					ACSL5	1.773E-08			PHACTR1	3.53E-05	CAPG	1.674E-13
ERH	4.771E-07					SQSTM1	2.097E-08			TUBB6	3.748E-05	MRPL34	1.752E-13
SYNE2	1.582E-06					GPRC5C	2.656E-08			ACBD3	3.776E-05	JUP	1.851E-13
CD44	3.043E-06					ARL6IP1	4.816E-08			GNAQ	4.08E-05	FGD4	1.923E-13
TXNDC17	4.865E-06					CCDC47	5.486E-08			ABCA3	4.358E-05	KIFC3	1.94E-13
CTSB	5.104E-06					CCDC88A	6.286E-08			ATP5H	4.545E-05	RAB17	2.064E-13
PNISR	5.251E-06					CDKN1A	7.607E-08			SREBF2	5.117E-05	PPP1R15A	2.167E-13
GPX1	5.612E-06					CSTB	8.324E-08			COX7B	5.118E-05	ATP5L	2.241E-13
ZFP36L2	5.665E-06					PSMA7	1.21E-07			PLIN2	6.074E-05	TJP2	2.476E-13
SLC1A5	8.835E-06					PPIB	2.719E-07			GADD45B	6.114E-05	SCAMP2	2.847E-13
SAT1	4.905E-05					GOLGA4	3.181E-07			ADI1	6.143E-05	NOP10	2.907E-13
MIF	8.681E-05					TNFAIP2	3.769E-07			C10orf54	6.201E-05	MYH14	3.283E-13
MYC	9.965E-05					AGPAT2	3.893E-07			ASNS	6.497E-05	LNX2	3.513E-13

Cluster 1 (basal cells)		Cluster 2 ($MUC5B^+$ club cells)		Cluster 3 ($SCGB3A2^{high}$ club cells)		Cluster 4 (type II alveolar cells)		Cluster 5 (type I alveolar cells-1)		Cluster 6 (type I alveolar cells-2)		Cluster 7 (type I alveolar cells-3)	
Gene symbol	Adjusted p values	Gene symbol	Adjusted p values	Gene symbol	Adjusted p values	Gene symbol	Adjusted p values	Gene symbol	Adjusted p values	Gene symbol	Adjusted p values	Gene symbol	Adjusted p values
HSPE1	0.0001175					MAST4	5.833E-07			PARVA	7.6E-05	LINC00493	5.094E-13
NCL	0.0004884					ITGAV	5.958E-07			YWHAH	7.746E-05	C2orf54	5.745E-13
F3	0.000769					OS9	5.991E-07			DUOX1	7.788E-05	LENG8	6.185E-13
IGFBP5	0.001585					ARF1	6.564E-07			SLC25A24	0.0001026	N4BP1	6.592E-13
TPSB2	0.0019956					SLC5A3	1.595E-06			ARHGAP5	0.0001256	COMT	6.622E-13
H2AFZ	0.0025423					DNAJB9	1.664E-06			PROS1	0.0001317	ANO6	6.918E-13
IGHA1	0.006285					GRB2	1.762E-06			ANKRD40	0.0001351	COX5B	7.519E-13
						FAM46A	3.755E-06			MYO1C	0.0001376	CHCHD5	8.298E-13
						VCP	1.124E-05			CPEB4	0.0001483	SEPP1	8.62E-13
						CCL20	0.0061343			ARHGAP3	0.0001646	PIM3	8.693E-13
										MYO1B	0.0001667	MRPL33	9.224E-13
										GAB1	0.0001704	NUMB	9.45E-13
										CORO1C	0.0001724	OTUD7B	9.761E-13
										ACAT1	0.0001881	OSGIN2	1.097E-12
										TNFRSF12	0.0001955	SSNA1	1.124E-12
										HIP1	0.0002044	KLF13	1.245E-12
										SNHG7	0.0002118	MRPL36	1.671E-12
										PIAS1	0.000223	C12orf76	1.731E-12
										SNX24	0.0002755	C10orf67	1.771E-12
										CALM2	0.0002757	AMOTL1	2.151E-12
										HOOK3	0.0002837	NBEAL2	2.219E-12
										TMEM189	0.0002985	PNKD	2.622E-12
										ATP10A	0.0003278	ATG101	2.623E-12
										TMEM41B	0.0003336	P4HA2	2.701E-12
										NBEAL2	0.0003513	PEBP4	3.045E-12
										SFTA1P	0.0003881	RTCB	3.57E-12
										SPAG9	0.0004178	ERGIC1	3.583E-12
										SC5D	0.0004239	COMTD1	3.616E-12
										COBL	0.0004691	RTN3	3.69E-12
										ANKS1A	0.0004782	RP11-65I12	4.006E-12
										MMS19	0.0005052	ANXA8L1	4.184E-12
										ID3	0.0005082	TMEM219	4.221E-12
										RETSAT	0.0005299	RNF144B	4.63E-12
										PACSIN2	0.0005413	JADE1	4.762E-12
										RCAN1	0.0006341	ABHD11-A	5.186E-12
										RBMS3	0.0006511	H2AFJ	5.505E-12
										RORA	0.0006517	TOM1L2	5.764E-12
										PPP2CB	0.0006918	FXYD4	5.832E-12
										C19orf33	0.0007192	SLC25A4	6.333E-12
										PSMA7	0.0007931	FSTL1	6.575E-12
										NUP58	0.000835	ZDHHC7	7.017E-12
										LPIN2	0.0009017	TMEM14C	7.455E-12
										NR3C1	0.0009673	SPTAN1	7.796E-12
										C1orf116	0.0010616	COL4A4	8.609E-12
										GKN2	0.0010702	ILK	8.84E-12
										KANK2	0.0011233	IDS	8.923E-12
										RAB27A	0.0012069	GUCD1	9.976E-12
										CPM	0.0013035	TXNDC15	1.024E-11
										N4BP1	0.0013614	SEMA3E	1.079E-11
										MEGF9	0.0013646	BMPR2	1.083E-11
										SMAD7	0.0014259	ZBTB4	1.118E-11
										DYNC1H1	0.0017198	RAD23A	1.133E-11
										TRIB1	0.0018724	CRB3	1.243E-11
										ANO6	0.0024014	CERS2	1.266E-11
										LRRFIP1	0.0028988	SPSB3	1.362E-11
										EPCAM	0.003026	MAFK	1.458E-11
										PMM1	0.0031784	KANSL3	1.583E-11
										STX7	0.0032299	ANXA2	1.81E-11
										NDUFA6	0.0033634	ARFGAP2	1.848E-11
										FLNA	0.0036757	RNF38	1.988E-11
										TMEM141	0.0042631	TMEM245	2.011E-11
										LAMC2	0.0050661	MIR4458H	2.021E-11
										MIR4435-2	0.0053485	UBE2B	2.057E-11
										PTPN12	0.0053569	PPDPF	2.402E-11
										MAFK	0.0055024	FERMT2	2.426E-11
										RYBP	0.0070517	PRKAB1	2.43E-11
										C19orf70	0.0072846	FAM174B	2.634E-11

Cluster 1 (basal cells)		Cluster 2 (MUC5B$^+$ club cells)		Cluster 3 (SCGB3A2^{high} club cells)		Cluster 4 (type II alveolar cells)		Cluster 5 (type I alveolar cells-1)		Cluster 6 (type I alveolar cells-2)		Cluster 7 (type I alveolar cells-3)	
**Gene symbol**	**Adjusted p values**	**Gene symbol**	**Adjusted p values**	**Gene symbol**	**Adjusted p values**	**Gene symbol**	**Adjusted p values**	**Gene symbol**	**Adjusted p values**	**Gene symbol**	**Adjusted p values**	**Gene symbol**	**Adjusted p values**
										ATP11A	0.0074634	TBCB	2.777E-11
										ILK	0.0075526	CNST	2.798E-11
										ATHL1	0.0078772	PARVA	2.86E-11
										STARD7	0.0082546	PSMB6	2.866E-11
										RNH1	0.0090039	ATP1A1	2.871E-11
										ERRFI1	0.0097779	REPS2	2.956E-11
										MACROD2	0.0109755	F3	2.989E-11
										CPNE3	0.0110782	ATP13A4	3E-11
										CLDN7	0.0130883	KCNMB1	3.116E-11
										CSRNP1	0.0140559	CDKN2B	3.274E-11
										FLRT3	0.0147582	MGAT1	3.287E-11
										TNS1	0.0181002	GGA2	3.473E-11
										KIAA1109	0.0220365	C19orf70	3.511E-11
										KLF2	0.0225268	DOT1L	3.518E-11
										SLC44A1	0.0245805	MFN2	3.634E-11
										EFNA1	0.0322914	LAMP1	3.715E-11
										FAM134B	0.0377845	PAFAH1B1	3.81E-11
												CTGF	4.056E-11
												CUX1	4.259E-11
												ATP5D	4.351E-11
												ARAP1	5.147E-11
												SULT1A2	5.153E-11
												SPRED1	5.206E-11
												ORMDL1	5.473E-11
												ADI1	5.818E-11
												LMTK2	5.947E-11
												SLC5A9	6.265E-11
												VPS28	6.294E-11
												CDH1	6.351E-11
												CHCHD6	7.008E-11
												PTRF	7.246E-11
												GALK1	7.263E-11
												RNF181	7.903E-11
												CLPTM1L	8.354E-11
												CAB39	8.449E-11
												MRPS34	8.505E-11
												PLXNA1	8.685E-11
												MGEA5	8.94E-11
												PMVK	9.409E-11
												LIMD1	9.431E-11
												ASAH1	9.911E-11
												LSM4	9.934E-11
												PEBP1	9.989E-11
												CXADR	1.004E-10
												SORT1	1.019E-10
												ECHDC1	1.044E-10
												SNX14	1.049E-10
												GPRIN2	1.061E-10
												BCAM	1.119E-10
												MYEOV2	1.172E-10
												SERTM1	1.225E-10
												PRDX6	1.319E-10
												NRG1	1.381E-10
												CDV3	1.425E-10
												KANK2	1.437E-10
												ATP5I	1.508E-10
												DHRS4L2	1.536E-10
												VAMP2	1.541E-10
												DYNC1H1	1.566E-10
												FAXDC2	1.588E-10
												SLC44A2	1.696E-10
												FAM198B	1.708E-10
												SLC6A4	1.821E-10
												HYAL1	1.876E-10
												MPPE1	1.981E-10
												STX11	2.017E-10
												TSPAN12	2.162E-10

Cluster 1 (basal cells)		Cluster 2 (MUC5B^{+} club cells)		Cluster 3 (SCGB3A2^{high} club cells)		Cluster 4 (type II alveolar cells)		Cluster 5 (type I alveolar cells-1)		Cluster 6 (type I alveolar cells-2)		Cluster 7 (type I alveolar cells-3)	
**Gene symbol**	**Adjusted p values**	**Gene symbol**	**Adjusted p values**	**Gene symbol**	**Adjusted p values**	**Gene symbol**	**Adjusted p values**	**Gene symbol**	**Adjusted p values**	**Gene symbol**	**Adjusted p values**	**Gene symbol**	**Adjusted p values**
												ANKRD11	2.183E-10
												SNX24	2.209E-10
												CDKL5	2.318E-10
												GRAMD1A	2.332E-10
												STK17B	2.358E-10
												GALC	2.471E-10
												PXDC1	2.625E-10
												EPAS1	2.63E-10
												SDC1	2.654E-10
												MYO9A	2.68E-10
												MYO5B	2.699E-10
												DUSP8	2.701E-10
												TLN1	2.74E-10
												HAGH	2.77E-10
												SH2D3A	2.781E-10
												SRGAP2C	3.161E-10
												SMAD4	3.245E-10
												CDC16	3.826E-10
												RAB25	3.85E-10
												REXO2	3.988E-10
												PRKCDBP	4.115E-10
												FAM189A2	4.458E-10
												C19orf60	4.506E-10
												FNBP1L	4.808E-10
												AMN	5.228E-10
												RNF41	5.256E-10
												GCNT2	5.441E-10
												TMTC2	5.955E-10
												RANBP17	6.05E-10
												SNAP23	6.072E-10
												EVI5	6.076E-10
												NHSL1	6.283E-10
												SHTN1	6.459E-10
												HRCT1	6.467E-10
												INADL	6.577E-10
												SGCE	6.784E-10
												VEPH1	6.83E-10
												RP1-78O14	7.332E-10
												PPP2CB	7.332E-10
												CSNK1D	8.116E-10
												GIPC1	8.151E-10
												ATP5J2	8.762E-10
												PDE4B	8.913E-10
												MRPL54	9.184E-10
												ACTR1A	9.196E-10
												AK1	9.241E-10
												RORA	9.366E-10
												PSMB5	9.434E-10
												GADD45GI	1.024E-09
												ACAT1	1.044E-09
												ZNF330	1.071E-09
												KLC4	1.086E-09
												LRRC1	1.124E-09
												GATA6-AS	1.135E-09
												N4BP2L2	1.149E-09
												STAT6	1.294E-09
												FBXO7	1.329E-09
												TMOD3	1.365E-09
												NDUFB10	1.444E-09
												RAB9A	1.499E-09
												PLEKHO1	1.582E-09
												PVRL2	1.639E-09
												PIN1	1.649E-09
												ERBB2	1.651E-09
												VEGFB	1.669E-09
												LINC01290	1.698E-09
												STXBP6	1.816E-09

Cluster 1 (basal cells)		Cluster 2 (MUC5B$^+$ club cells)		Cluster 3 (SCGB3A2^{high} club cells)		Cluster 4 (type II alveolar cells)		Cluster 5 (type I alveolar cells-1)		Cluster 6 (type I alveolar cells-2)		Cluster 7 (type I alveolar cells-3)	
Gene symbol	Adjusted p values	Gene symbol	Adjusted p values	Gene symbol	Adjusted p values	Gene symbol	Adjusted p values	Gene symbol	Adjusted p values	Gene symbol	Adjusted p values	Gene symbol	Adjusted p values
												ARHGEF2	1.886E-09
												ARL8A	1.896E-09
												UBL5	1.905E-09
												C7orf50	1.956E-09
												ITGB1	1.991E-09
												ZC2HC1A	2.004E-09
												CTBP1	2.069E-09
												CRY2	2.172E-09
												FAM174A	2.385E-09
												TUG1	2.447E-09
												ABTB1	2.464E-09
												STX12	2.464E-09
												ZNF451	2.698E-09
												LMBRD1	2.981E-09
												SH3GLB2	3.195E-09
												RBMS1	3.394E-09
												ACAA1	3.421E-09
												SCARB2	3.529E-09
												AGRN	3.566E-09
												WBP5	3.703E-09
												ORMDL2	3.717E-09
												RASSF8	3.81E-09
												MSN	3.869E-09
												MARK3	4.194E-09
												MKNK2	4.342E-09
												ANXA11	4.508E-09
												IARS2	4.63E-09
												DPYSL2	4.997E-09
												TMEM2	5.329E-09
												COL18A1	5.647E-09
												LINC00116	5.793E-09
												DNAJC5	5.807E-09
												IL6R	5.953E-09
												TPPP	6.356E-09
												U47924.27	6.475E-09
												DERA	6.983E-09
												PVR	7.567E-09
												YPEL3	7.866E-09
												CGN	7.898E-09
												NME3	7.932E-09
												FKBP9	8.076E-09
												EIF1B	8.391E-09
												TNKS1BP1	8.548E-09
												POGK	8.873E-09
												CCDC85A	9.266E-09
												ADAM9	9.488E-09
												SUMO3	9.546E-09
												RCOR1	9.606E-09
												FLII	1.002E-08
												FZD6	1.037E-08
												FNTA	1.06E-08
												MRPL47	1.098E-08
												MIR22HG	1.118E-08
												WTIP	1.141E-08
												SDC2	1.171E-08
												DDAH2	1.18E-08
												WNT3A	1.253E-08
												CREBL2	1.258E-08
												CLUH	1.281E-08
												HSBP1L1	1.296E-08
												RAB6A	1.303E-08
												ATP5O	1.338E-08
												ARPC5	1.358E-08
												MARC2	1.398E-08
												PXDN	1.498E-08
												PTRH1	1.588E-08
												SPSB2	1.615E-08

Cluster 1 (basal cells)		Cluster 2 (MUC5B$^+$ club cells)		Cluster 3 (SCGB3A2^{high} club cells)		Cluster 4 (type II alveolar cells)		Cluster 5 (type I alveolar cells-1)		Cluster 6 (type I alveolar cells-2)		Cluster 7 (type I alveolar cells-3)	
Gene symbol	Adjusted p values	Gene symbol	Adjusted p values	Gene symbol	Adjusted p values	Gene symbol	Adjusted p values	Gene symbol	Adjusted p values	Gene symbol	Adjusted p values	Gene symbol	Adjusted p values
												RHOBTB2	1.685E-08
												ADPRH	1.716E-08
												LIMS2	1.726E-08
												DOCK4	1.741E-08
												C4BPA	1.802E-08
												CHPT1	1.875E-08
												PKP3	1.974E-08
												C1orf168	2.04E-08
												INPPL1	2.042E-08
												ATHL1	2.06E-08
												NDUFAB1	2.093E-08
												RIMKLA	2.167E-08
												ACADVL	2.298E-08
												DDRGK1	2.35E-08
												TTC7A	2.459E-08
												DCTN4	2.48E-08
												RER1	2.486E-08
												PLEKHB2	2.519E-08
												B3GNT2	2.552E-08
												TMEM129	2.571E-08
												NCBP2-AS	2.649E-08
												PGM1	2.818E-08
												NAA60	2.921E-08
												CTIF	3.019E-08
												ANG	3.052E-08
												ATP6V1E1	3.056E-08
												SIK2	3.092E-08
												TNS3	3.104E-08
												FZD5	3.21E-08
												TPRG1L	3.214E-08
												COBL	3.271E-08
												PDK4	3.287E-08
												INPP5K	3.343E-08
												TMEM259	3.356E-08
												GSDMD	3.363E-08
												TMEM43	3.396E-08
												REEP5	3.443E-08
												CORO1C	3.501E-08
												MARVELD	3.546E-08
												TRMT112	3.552E-08
												MRFAP1	3.632E-08
												ATP5B	3.643E-08
												FAM134C	3.7E-08
												NDUFA12	3.811E-08
												CARD16	3.865E-08
												TUFM	3.911E-08
												UNC13B	3.928E-08
												FKBP8	3.958E-08
												YEATS2	4.341E-08
												CBR1	4.442E-08
												SFTA3	4.891E-08
												PIEZO1	4.971E-08
												NDUFA4	5.072E-08
												PRKAR1A	5.137E-08
												PNPLA6	5.164E-08
												RHOA	5.388E-08
												NR2C1	5.475E-08
												RNF128	5.598E-08
												PAPSS1	5.86E-08
												CCDC6	5.936E-08
												EBLN3	6.037E-08
												ERRFI1	6.1E-08
												MRPS21	6.515E-08
												SACM1L	6.625E-08
												SUMF2	6.711E-08
												MT-ND4L	6.834E-08
												NOSTRIN	7.26E-08

Cluster 1 (basal cells)		Cluster 2 (MUC5B^{+} club cells)		Cluster 3 (SCGB3A2^{high} club cells)		Cluster 4 (type II alveolar cells)		Cluster 5 (type I alveolar cells-1)		Cluster 6 (type I alveolar cells-2)		Cluster 7 (type I alveolar cells-3)	
Gene symbol	Adjusted p values	Gene symbol	Adjusted p values	Gene symbol	Adjusted p values	Gene symbol	Adjusted p values	Gene symbol	Adjusted p values	Gene symbol	Adjusted p values	Gene symbol	Adjusted p values
												FAM168B	7.445E-08
												GK5	7.557E-08
												RASAL2	7.87E-08
												ARHGAP5	8.007E-08
												APBB2	8.118E-08
												RBCK1	8.275E-08
												RP4-568C1	8.42E-08
												BLOC1S1	8.831E-08
												PHACTR1	8.886E-08
												COX6B1	9.154E-08
												WDR13	9.176E-08
												MVP	9.267E-08
												STX3	9.308E-08
												NDUFB9	9.421E-08
												MISP	9.464E-08
												SPG7	9.627E-08
												RHOU	1.016E-07
												CCDC64B	1.038E-07
												LGALS8	1.04E-07
												BAG5	1.052E-07
												PAM	1.069E-07
												NDUFA1	1.079E-07
												RNF19A	1.152E-07
												PPP1R13L	1.162E-07
												C6orf1	1.196E-07
												PXN	1.208E-07
												CRIP2	1.271E-07
												RBMS2	1.314E-07
												GOLPH3	1.317E-07
												CD81	1.321E-07
												ZNF706	1.331E-07
												RETSAT	1.356E-07
												ETF1	1.377E-07
												ADH5	1.383E-07
												ANXA5	1.424E-07
												GALNT5	1.428E-07
												TRAPPC1	1.446E-07
												ZC3H14	1.454E-07
												SPAG9	1.468E-07
												FBXW5	1.499E-07
												MBTPS1	1.514E-07
												TPM4	1.572E-07
												DAPK1	1.608E-07
												RAD21	1.611E-07
												USP25	1.636E-07
												DAD1	1.646E-07
												RBX1	1.69E-07
												CAT	1.911E-07
												SP100	1.982E-07
												UXS1	2.007E-07
												KIF1B	2.052E-07
												EVA1A	2.061E-07
												IFI35	2.135E-07
												SEMA6D	2.186E-07
												CDIPT	2.252E-07
												SLC35A3	2.257E-07
												CHMP5	2.352E-07
												JOSD2	2.379E-07
												LAMTOR4	2.44E-07
												CTNNA1	2.443E-07
												COX7A2	2.494E-07
												DNAJB4	2.812E-07
												PLCD1	2.93E-07
												MICA	2.951E-07
												GNB1	3.192E-07
												TUFT1	3.223E-07
												ZDHHC20	3.34E-07

Cluster 1 (basal cells)		Cluster 2 (MUC5B+ club cells)		Cluster 3 (SCGB3A2high club cells)		Cluster 4 (type II alveolar cells)		Cluster 5 (type I alveolar cells-1)		Cluster 6 (type I alveolar cells-2)		Cluster 7 (type I alveolar cells-3)	
**Gene symbol**	**Adjusted p values**	**Gene symbol**	**Adjusted p values**	**Gene symbol**	**Adjusted p values**	**Gene symbol**	**Adjusted p values**	**Gene symbol**	**Adjusted p values**	**Gene symbol**	**Adjusted p values**	**Gene symbol**	**Adjusted p values**
												BAIAP2	3.383E-07
												ABI2	3.388E-07
												SLC26A2	3.415E-07
												ERBB3	3.442E-07
												RNF8	3.665E-07
												AQP4	3.728E-07
												PLEKHH2	3.738E-07
												SCOC	3.861E-07
												ACOT8	3.889E-07
												PRR34-AS[illegible]	3.969E-07
												SYNE2	3.989E-07
												LCAT	4.31E-07
												MGST3	4.355E-07
												PET100	4.369E-07
												ARHGAP2[illegible]	4.575E-07
												LATS2	4.712E-07
												SEC13	4.767E-07
												TMEM41B	4.844E-07
												ABHD14B	4.85E-07
												PRR15L	4.95E-07
												USP33	4.981E-07
												MAP4	5.014E-07
												MSLN	5.057E-07
												TXNDC11	5.148E-07
												FAM219B	5.204E-07
												ACOT9	5.221E-07
												KLF9	5.268E-07
												GNG5	5.275E-07
												DGCR2	5.31E-07
												CASZ1	5.332E-07
												RRAGC	5.343E-07
												TRIM28	5.479E-07
												HMGCL	5.481E-07
												MRPL52	5.73E-07
												PQLC1	5.856E-07
												CYHR1	6.023E-07
												NISCH	6.13E-07
												VCL	6.289E-07
												ACTR1B	6.364E-07
												ZNHIT1	6.676E-07
												SNHG18	7.73E-07
												TSPAN7	7.884E-07
												ROR1	8.705E-07
												ZBTB38	8.927E-07
												DMKN	9.019E-07
												PHF10	9.519E-07
												DEGS1	9.663E-07
												SMIM4	9.984E-07
												RAP1A	1.014E-06
												THRA	1.123E-06
												RHOF	1.255E-06
												CTD-2626[illegible]	1.322E-06
												TIMM13	1.353E-06
												SPRED2	1.45E-06
												MOAP1	1.453E-06
												GABARAP	1.497E-06
												NDNF	1.502E-06
												NDUFS6	1.507E-06
												MMP28	1.514E-06
												MED10	1.531E-06
												DIAPH3	1.582E-06
												MGAT4B	1.649E-06
												LIPH	1.711E-06
												ATF4	1.712E-06
												CDC42BPE[illegible]	1.716E-06
												PPIA	1.723E-06
												FOXA2	1.732E-06

Cluster 1 (basal cells)		Cluster 2 (MUC5B^{+} club cells)		Cluster 3 (SCGB3A2^{high} club cells)		Cluster 4 (type II alveolar cells)		Cluster 5 (type I alveolar cells-1)		Cluster 6 (type I alveolar cells-2)		Cluster 7 (type I alveolar cells-3)	
Gene symbol	Adjusted p values	Gene symbol	Adjusted p values	Gene symbol	Adjusted p values	Gene symbol	Adjusted p values	Gene symbol	Adjusted p values	Gene symbol	Adjusted p values	Gene symbol	Adjusted p values
												SDHA	1.775E-06
												ATP2C2	1.925E-06
												SOD1	2.044E-06
												UBA1	2.09E-06
												ORMDL3	2.207E-06
												TAGLN	2.312E-06
												MXI1	2.398E-06
												ANXA1	2.473E-06
												THUMPD1	2.509E-06
												CTNND1	2.557E-06
												AVPI1	2.562E-06
												ACSS1	2.702E-06
												RPS6KA1	2.998E-06
												SNX21	3.004E-06
												DESI2	3.031E-06
												ICAM1	3.271E-06
												DLGAP1-A	3.386E-06
												AGAP3	3.399E-06
												RIN2	3.491E-06
												SREBF2	3.526E-06
												PMF1	3.544E-06
												FZD8	3.604E-06
												GNG12	3.616E-06
												ITFG1	3.632E-06
												AJUBA	3.704E-06
												SLC39A13	3.805E-06
												TBC1D9	3.852E-06
												GATA6	3.987E-06
												VSIG10	4.087E-06
												FAM46B	4.099E-06
												SCO2	4.109E-06
												C16orf13	4.135E-06
												TNFAIP1	4.237E-06
												WNK1	4.293E-06
												GLRX	4.363E-06
												STUB1	4.731E-06
												C5orf56	5.225E-06
												FOSL2	5.29E-06
												MAPK1IP1	5.328E-06
												DCTN2	5.401E-06
												APC	5.711E-06
												FAM96B	5.811E-06
												C21orf2	6.1E-06
												BNIP2	6.238E-06
												SGMS1	6.284E-06
												FCHO2	6.376E-06
												EXOSC6	6.57E-06
												TPRN	6.694E-06
												RP1-151F1	6.719E-06
												VPS37B	7.058E-06
												DDX24	7.241E-06
												SHROOM3	7.3E-06
												SRRM2	7.951E-06
												ISG15	8.398E-06
												IFNGR1	8.495E-06
												MARCH6	8.525E-06
												RICTOR	9.002E-06
												PPP4R1	9.189E-06
												DNASE2	9.203E-06
												WDR34	9.728E-06
												JOSD1	9.802E-06
												TNIP1	1.012E-05
												ZNF106	1.039E-05
												NAA38	1.058E-05
												PAXIP1-AS	1.071E-05
												SNX1	1.107E-05
												ARHGAP4	1.24E-05

Cluster 1 (basal cells)		Cluster 2 (MUC5B^{+} club cells)		Cluster 3 (SCGB3A2^{high} club cells)		Cluster 4 (type II alveolar cells)		Cluster 5 (type I alveolar cells-1)		Cluster 6 (type I alveolar cells-2)		Cluster 7 (type I alveolar cells-3)	
Gene symbol	Adjusted p values	Gene symbol	Adjusted p values	Gene symbol	Adjusted p values	Gene symbol	Adjusted p values	Gene symbol	Adjusted p values	Gene symbol	Adjusted p values	Gene symbol	Adjusted p values
												NFATC3	1.254E-05
												H1FX	1.288E-05
												RP11-347P	1.312E-05
												FAM200B	1.318E-05
												CPEB4	1.333E-05
												RAB3IP	1.35E-05
												RIPK4	1.35E-05
												BLVRA	1.368E-05
												SLC39A1	1.369E-05
												SFTPD	1.383E-05
												MEGF9	1.391E-05
												MYH9	1.392E-05
												ATP6AP1	1.43E-05
												KIAA1109	1.474E-05
												DNAJB6	1.484E-05
												CMTM4	1.53E-05
												CIRBP	1.538E-05
												NDUFA13	1.573E-05
												SMG9	1.579E-05
												FOXJ3	1.614E-05
												POLR2J3	1.718E-05
												PEG10	1.758E-05
												BCR	1.919E-05
												AES	1.94E-05
												PLCE1	2.006E-05
												MAPK3	2.115E-05
												WNT7A	2.153E-05
												ECHDC3	2.168E-05
												TUBB6	2.25E-05
												MOSPD2	2.255E-05
												CISD1	2.26E-05
												ZNF83	2.489E-05
												ARL5A	2.496E-05
												DENND5A	2.559E-05
												TCEB2	2.635E-05
												DDX58	2.676E-05
												HELZ	2.69E-05
												DENND6A	2.735E-05
												TMEM63B	2.765E-05
												CRYZ	2.768E-05
												HCFC2	2.834E-05
												ACTR10	2.926E-05
												CLIP4	2.984E-05
												UBE2H	3.079E-05
												LRP10	3.079E-05
												CAMK2G	3.084E-05
												PRRG4	3.085E-05
												GPR108	3.106E-05
												DYNC1I2	3.246E-05
												PIK3IP1	3.27E-05
												EIF6	3.379E-05
												SAMD4A	3.404E-05
												ATXN2	3.442E-05
												CYTH2	3.541E-05
												CNKSR1	3.62E-05
												OCLN	3.623E-05
												PTDSS1	3.73E-05
												EDIL3	3.806E-05
												ARRB1	3.942E-05
												STX2	4.009E-05
												FBXL3	4.239E-05
												YPEL5	4.737E-05
												ADCY6	4.962E-05
												CHCHD1	5.073E-05
												B3GALNT1	5.287E-05
												RASSF1	5.314E-05
												PIM1	5.374E-05

Cluster 1 (basal cells)		Cluster 2 ($MUC5B^+$ club cells)		Cluster 3 ($SCGB3A2^{high}$ club cells)		Cluster 4 (type II alveolar cells)		Cluster 5 (type I alveolar cells-1)		Cluster 6 (type I alveolar cells-2)		Cluster 7 (type I alveolar cells-3)	
**Gene symbol**	**Adjusted p values**	**Gene symbol**	**Adjusted p values**	**Gene symbol**	**Adjusted p values**	**Gene symbol**	**Adjusted p values**	**Gene symbol**	**Adjusted p values**	**Gene symbol**	**Adjusted p values**	**Gene symbol**	**Adjusted p values**
												CTTN	5.493E-05
												STK17A	5.602E-05
												ATF3	5.66E-05
												C4orf3	5.842E-05
												XPO4	5.91E-05
												FILIP1	6.191E-05
												SLC19A1	6.506E-05
												TMEM37	6.608E-05
												FAM109A	6.623E-05
												HIRIP3	6.772E-05
												CYLD	6.808E-05
												PINK1	7.053E-05
												ARHGAP2[illegible]	7.069E-05
												BCL2L1	7.076E-05
												ANKRD13[illegible]	7.309E-05
												SUN2	7.609E-05
												TMEM98	7.668E-05
												B4GAT1	8.273E-05
												WDR11	8.636E-05
												PPL	9.717E-05
												MMP24	0.0001008
												TUBA4A	0.0001011
												OSGIN1	0.0001085
												B2M	0.0001131
												NDUFB4	0.000115
												HACD1	0.0001197
												LBH	0.0001262
												CLIC2	0.0001377
												ACSF2	0.0001529
												HTATIP2	0.0001535
												KAT2B	0.000174
												EFR3B	0.0001788
												PPM1K	0.000182
												PARP14	0.0001843
												DDHD2	0.0001846
												DNAJB1	0.0001967
												YWHAH	0.0002214
												CRIP1	0.0002296
												NPEPL1	0.0002298
												MAPK1	0.0002406
												SHANK2	0.0002467
												HDAC7	0.0002534
												CLDN7	0.0002814
												PLXNB2	0.0002851
												SERPING1	0.0002877
												NELFB	0.0003166
												MEF2D	0.0003205
												APEH	0.0003366
												HOMER1	0.0003409
												ATL3	0.0003409
												SMAD3	0.0003674
												PSME1	0.0003729
												TRIM23	0.0003791
												PMM1	0.0003829
												ATP1B3	0.0003829
												NINJ2	0.0004239
												ZFHX3	0.0004474
												ARSJ	0.0004638
												NBN	0.0004803
												DUOXA1	0.0005061
												SARAF	0.0005238
												SMAD9	0.0005529
												AKR1C3	0.00056
												CLDN4	0.0005995
												TOMM7	0.0006019
												LPCAT3	0.000607
												GRB7	0.0006418

Cluster 1 (basal cells)		Cluster 2 (MUC5B$^+$ club cells)		Cluster 3 (SCGB3A2^{high} club cells)		Cluster 4 (type II alveolar cells)		Cluster 5 (type I alveolar cells-1)		Cluster 6 (type I alveolar cells-2)		Cluster 7 (type I alveolar cells-3)	
**Gene symbol**	**Adjusted p values**	**Gene symbol**	**Adjusted p values**	**Gene symbol**	**Adjusted p values**	**Gene symbol**	**Adjusted p values**	**Gene symbol**	**Adjusted p values**	**Gene symbol**	**Adjusted p values**	**Gene symbol**	**Adjusted p values**
												PPP1R13B	0.0006595
												PKN1	0.0006725
												NDUFA11	0.0006958
												SLC3A2	0.0007024
												ULK2	0.0007259
												ZNF704	0.0007433
												ANXA4	0.0008747
												SGK223	0.0009369
												TRPM4	0.0009551
												TMCC1	0.0011368
												TUBA1A	0.001147
												TPRA1	0.0012284
												LGALS3BP	0.0012496
												SLC15A2	0.0014434
												WWC1	0.001573
												FLRT3	0.00159
												LRP5	0.0016608
												ATP11A	0.0018659
												TINAGL1	0.0020094
												AC008268.	0.0020176
												DYNLL1	0.002041
												RP11-172H	0.0022419
												TMEM246	0.0025205
												MAL2	0.0027509
												IGSF8	0.0030766
												ZC3H12C	0.0036675
												RAD17	0.003978
												MAGI3	0.0043121
												NCOA7	0.0044826
												RAB23	0.0045859
												THSD7A	0.0048821
												JUN	0.0050128
												DHCR24	0.0054381
												TMEM30A	0.0062157
												DNAJC15	0.0077335
												FAM134B	0.0090781
												COX7C	0.0094686
												MUC15	0.0101737
												C3orf58	0.0114927
												LTBP4	0.0137477
												TGFB2	0.0155468
												SCD5	0.0158337
												ADCY9	0.0165919
												VAPA	0.0184377
												COX8A	0.0200808
												CD9	0.0214565
												TMBIM1	0.0216465
												SESN1	0.0220776
												CHCHD2	0.0255336
												SMAD7	0.0420655

1The airway and alveolar epithelial cells were from the cluster 1 in Figure 1A&C

Supplement Figure Legends

Supplemental Figure 1. Expression of the canonical markers of the different cell populations from **A.** control and **B.** IPF human lungs in the t-SNE plots. Basal cell – KRT15; ciliated cell – DNAI1; type I alveolar cells – ABCA3; type II alveolar cells – HOPX; T cells – CD8A; mast cells – KIT; plasma cells – CD27; monocytes – ITGAM; alveolar macrophages – PPARG; endothelial cells – PECAM1; and fibroblasts – COL14A1.

Supplemental Figure 2. Unsupervised t-SNE clustering of the single cells from cluster 1 (Figure 1A, C) in each individual. *Top* – controls, n=4; *bottom* – IPF, n=4.

Supplemental Figure 3. Airway and alveolar epithelial cell populations in controls *vs.* IPF. **A.** Violin plots of basal cell marker KRT5, type II alveolar cell markers SFTPC and ABCA3 and type I alveolar cell markers AGER and HOPX in cell populations from control (C) and IPF (I) donors identified in Figure 2A and Supplemental Figure 2. Note that sub-cluster 4 was not detected (ND) in control cell populations. **B.** Relative size of epithelial cell populations (shown in panel **A.**) in controls (*left*) vs. IPF (*right*). p values are shown in the figure.

Supplemental Figure 4. Gene ontology analysis of the dysregulated genes in the $SCGB3A2^{high}$ club cells of IPF patients. **A.** Top 10 functional categories of the down-regulated genes in $SCGB3A2^{high}$ club cells of IPF patients. **B.** Top 10 functional categories of the up-regulated genes in $SCGB3A2^{high}$ club cells of IPF patients. The analysis was performed on https://david.ncifcrf.gov/.

Supplemental Figure 5. Slingshot analysis of the single cells from IPF patients. The single cells were from Figure 2A (right side). Two lineages were identified. Lineage 1: (1) basal cells -> (3) $SCGB1A1^{+}SCGB3A2^{high}$ club cells -> (2) $SCGB1A1^{+}$MUC5B+ club cells. Lineage 2: (1) basal cells -> (3) $SCGB1A1^{+}SCGB3A2^{high}$ club cells -> (4) intermediate AT -> (5) intermediate AT -> (6) intermediate AT -> (7) AT1.

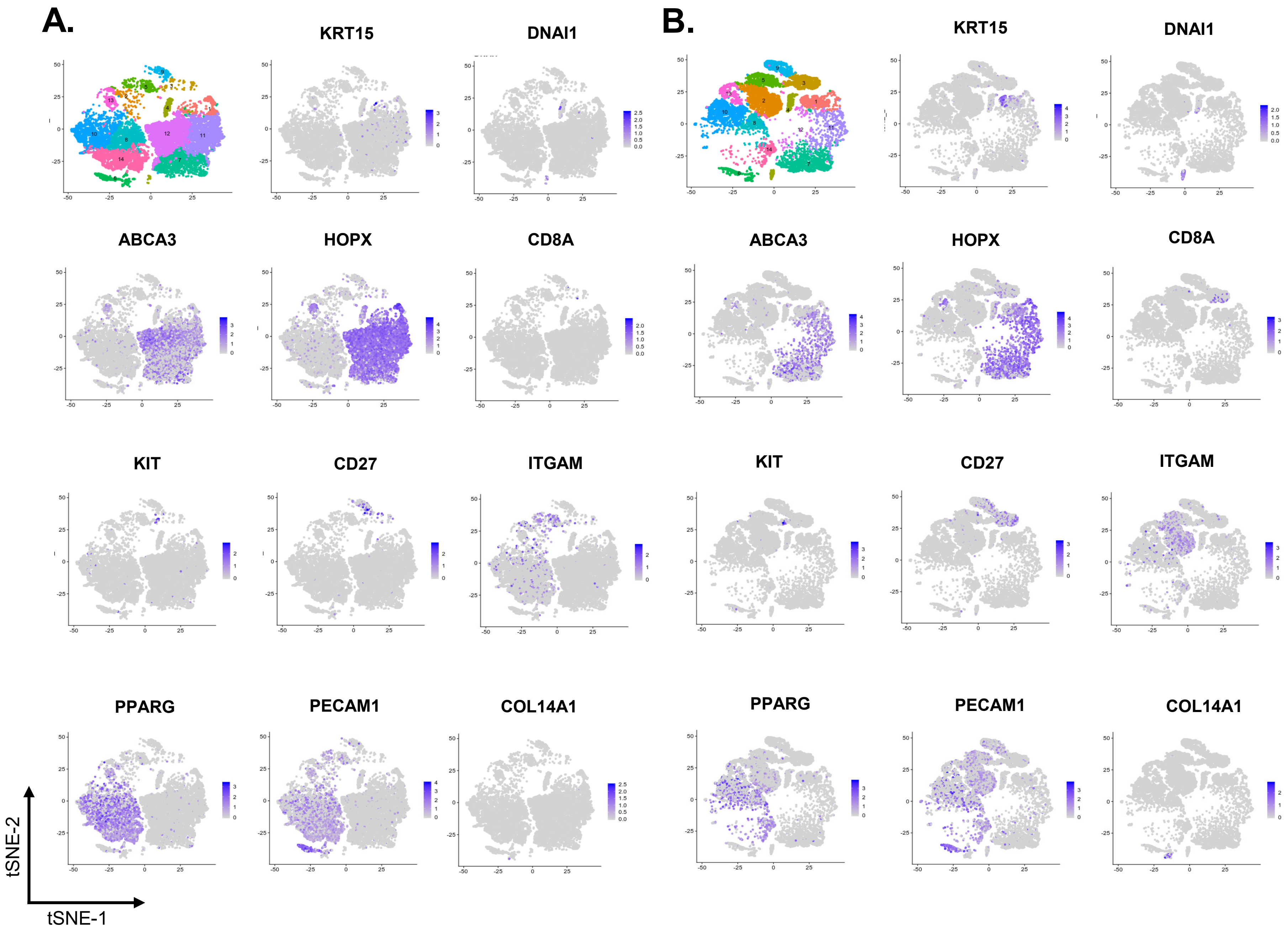

Supplemental Figure 1

Supplemental Figure 2

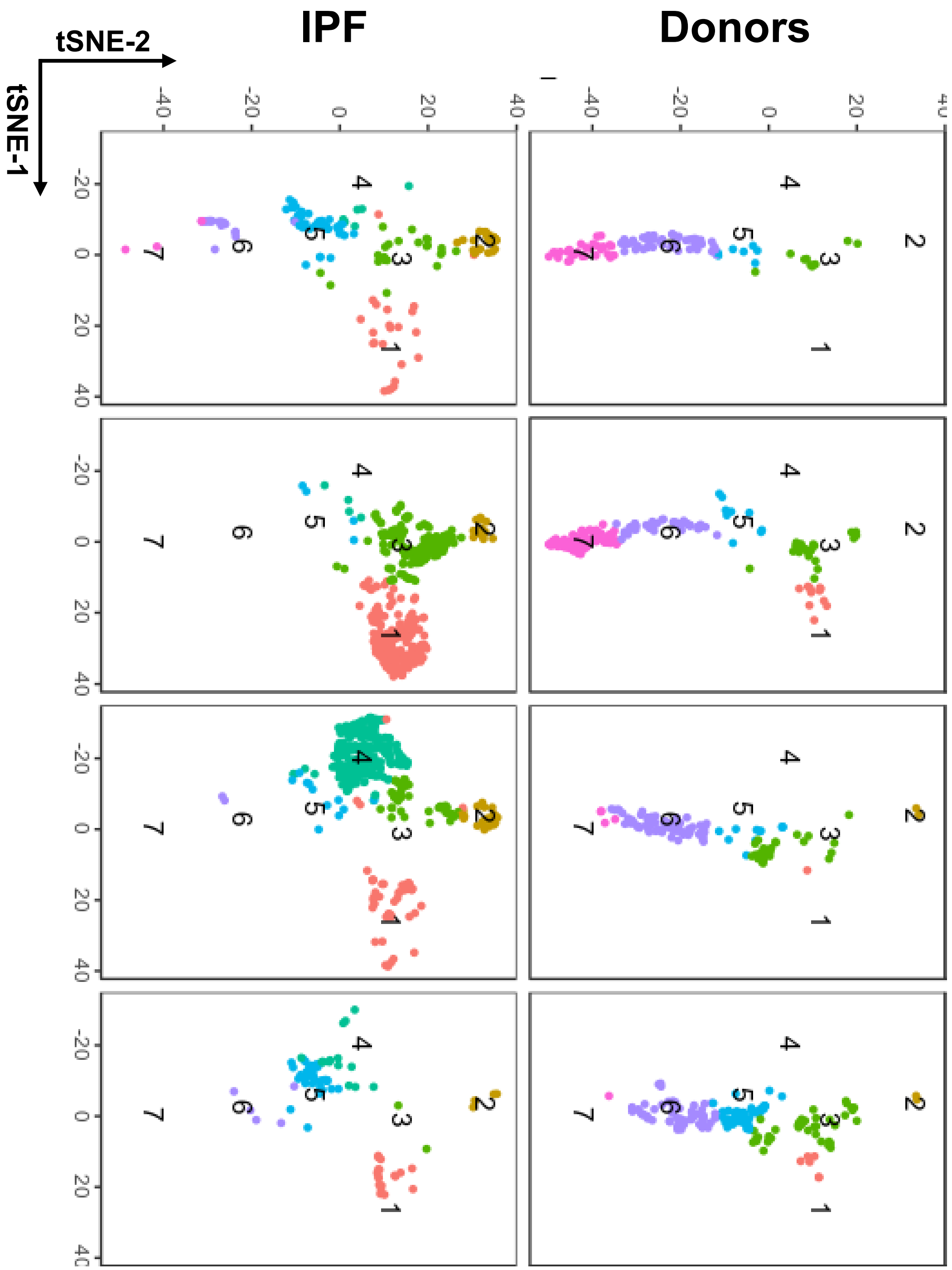

Supplemental Figure 3

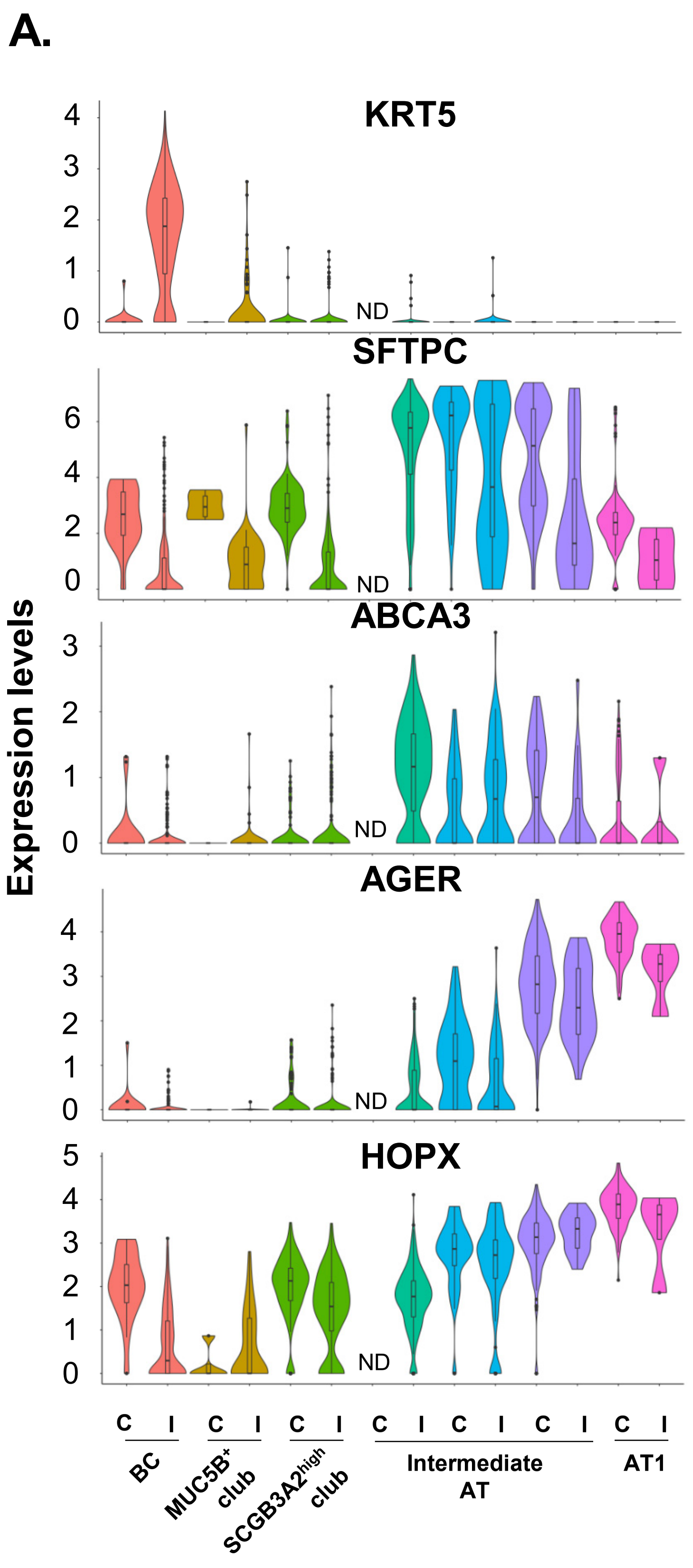

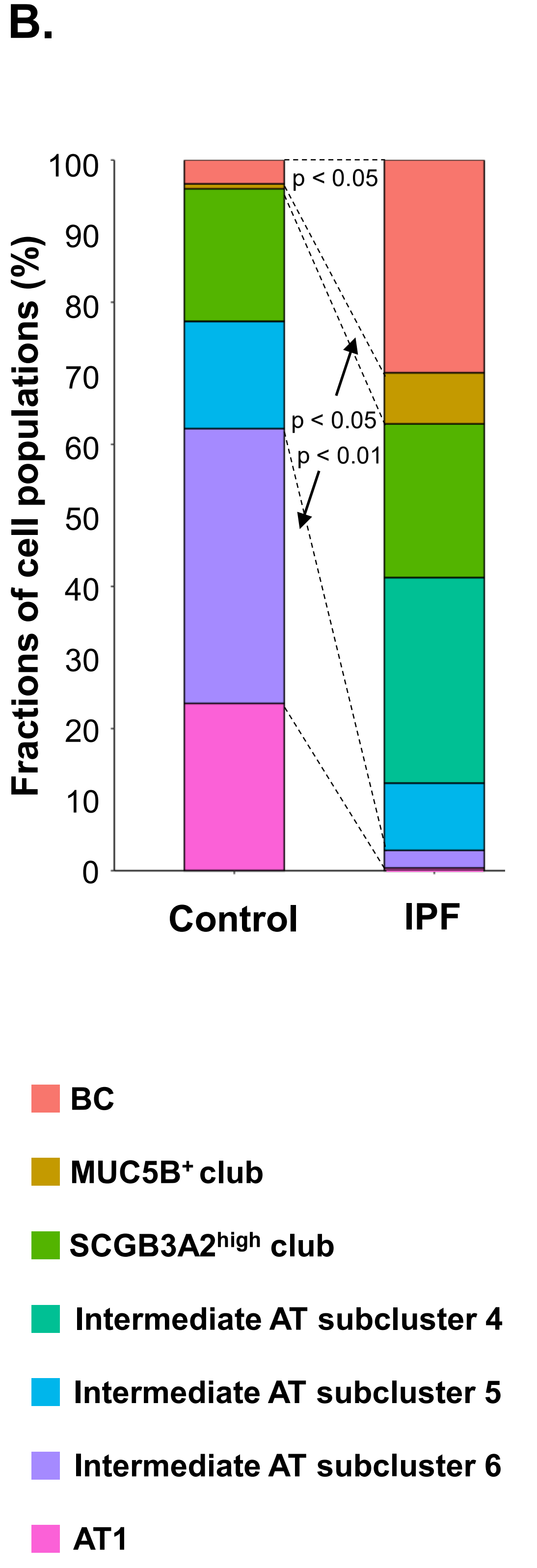

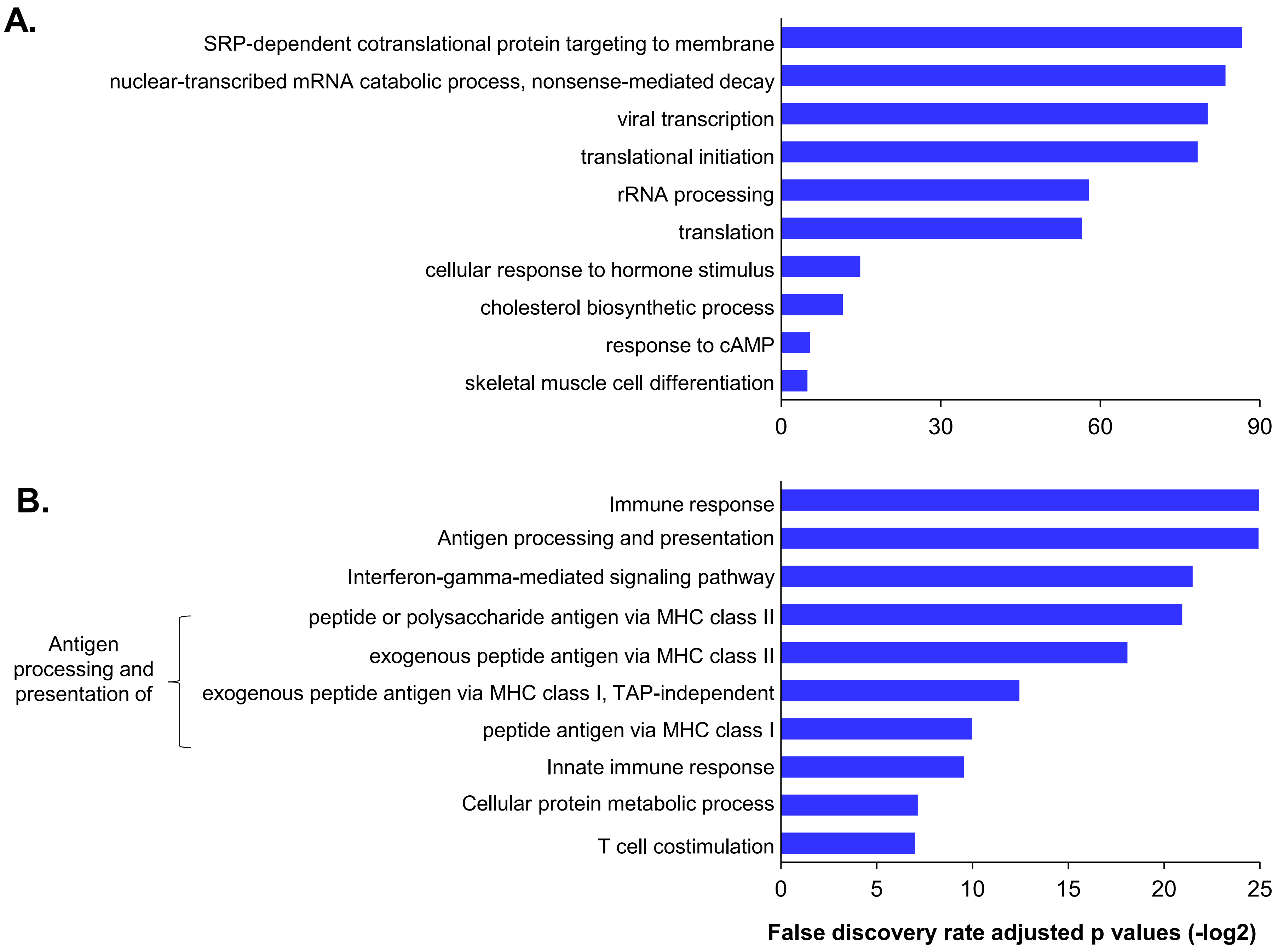

Supplemental Figure 4

Supplemental Figure 5

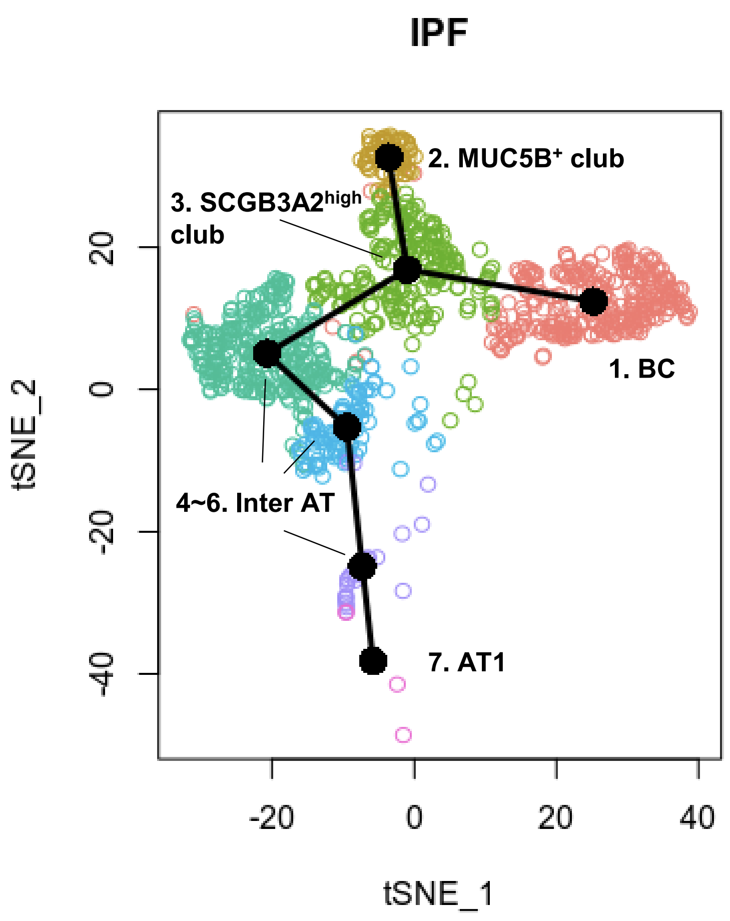

Supplement: S1 File — (PDF) [file pone.0237529.s001.pdf]
